# Spoken disagreement is more constructive than written disagreement

**DOI:** 10.1038/s41467-026-71669-5

**Published:** 2026-04-27

**Authors:** Burint Bevis, Juliana Schroeder, Michael Yeomans

**Affiliations:** 1 Department of Management and Entrepreneurship, Imperial Business School, Imperial College London, London, UK; 2 Department of Management and Organizations, Haas School of Business, University of California at Berkeley, Berkeley, CA USA

**Keywords:** Communication, Human behaviour

## Abstract

Amid rising global polarization, finding ways to disagree constructively is vital. This paper examines whether the medium of disagreement—spoken or written—shapes conversation outcomes. A series of randomized experiments (*N* = 1,576 conversation partners who had 1,842 conversations and 1,432 observers of those conversations) suggests that spoken conversations with a disagreeing counterpart lead to greater understanding, lower conflict, more favorable impressions of one’s counterpart, and greater attitude alignment than written ones. Speech also fosters more conversational receptiveness—cues in language that signal openness to opposing viewpoints—which partly mediates the effects of medium on these constructive disagreement outcomes. The conversation medium further moderates the association between language and outcomes: receptiveness is a stronger predictor of constructive disagreement in writing than in speech, suggesting people use less receptive language in the very medium in which it may be most effective (i.e., the written medium). A final study suggests that people may misjudge the effects of medium, wrongly believing spoken (versus written) disagreement will be less constructive and preferring to write to disagreeing counterparts. Despite people’s erroneous beliefs, spoken conversation offers a promising path to disagreeing constructively.

## Abstract (web summary)

Here the authors examine how spoken versus written conversation affects conversational receptiveness, and find that speaking is more constructive during disagreements than writing.

## Introduction

Political and social disagreement has been steadily increasing for decades in America^1,2^. Despite the value of having diverse perspectives for innovation and problem-solving^3–7^, animosity tends to arise between those with strongly opposing views^2,8–11^. People typically resolve disagreements via conversation, wherein counterparts exchange and discuss one another’s viewpoints. But although conversations can promote mutual understanding and bridge divides^12–15^, in times of disagreement, they can also exacerbate conflict^16,17^. This begs the question—how can we make conversations between disagreeing counterparts more constructive?

Prior research has examined many inputs to constructive disagreement, including the beliefs people have about one another and to whom they speak^12,18–23^. But many difficult conversations are important precisely because a specific issue must be discussed with a specific person. The present study examines another aspect of discourse that we suggest plays a key role in fostering constructive disagreement: the conversation medium. Not only does the medium represent a central element of any interaction, it also typically lies within individuals’ control.

In a series of randomized experiments that take into consideration different conversation formats (e.g., duration of conversations, synchronicity) and a range of topic issues and population samples, we show that conversation medium causally influences the constructive nature of disagreements. Specifically, we focus our comparisons on two ubiquitous conversation media—spoken and written. Moreover, we explore several potential reasons for different conversational outcomes when speaking or writing, including different uses of language as a function of medium. In aggregate, our experiments suggest that by opting for spoken over written communication, individuals can foster deeper understanding, reduce perceived conflict, and enhance impressions of disagreeing counterparts.

Communication theorist Marshall McLuhan once wrote: “The medium is the message”^24^. Indeed, media richness theory suggests that “richer” media (e.g., being face-to-face) can result in more effective communication than “leaner” media (e.g., emailing)—yet this theory has been modified and debated extensively since its inception^25–30^. Many questions remain, including what exactly makes a medium “rich” and how richness affects social outcomes, especially in high-stakes conversations such as conflict resolution. Furthermore, the effects of medium are often studied in one-way communication, to isolate either how conversation medium changes the production of linguistic content (e.g., speaking versus writing) or the consumption of content (e.g., listening versus reading)^31–33^. But in natural conversation, people simultaneously produce and consume language together, while constantly updating their beliefs about one another. Little research has studied the combined effects of medium on conversation, particularly in disagreement conversations (for exceptions^34,35^).

In considering different potential outcomes when speaking or writing, several prior findings lead us to hypothesize that spoken conversations will result in more constructive dialogue than written ones. First, people tend to infer communicators’ mental states more accurately and have higher impressions of their mental capacities when they hear communicators compared to reading their same words, suggesting spoken conversations may lead people to understand each other better and perceive each other more positively^33,36–40^. Indeed, speech can accurately convey an experience or intention even without containing semantic content (e.g., via prosodic features like tone of voice), highlighting how effectively it conveys a communicator’s mental states^41,42^. Second, speaking tends to be a more immediate and synchronous medium than writing. The broad range of expressive cues in speech, such as tonal inflexions and back-channeling, allows speakers to convey thoughts and respond to others’ thoughts quickly. For example, in speech, active listening is expressed in turn from non-verbal and verbal cues^43–46^.

But it is still possible that the potential advantages of speech – such as its capacity to authentically and quickly convey mental content – might also exacerbate disagreement, for instance by magnifying negative emotional reactions that individuals may prefer to conceal^16,17,47^. Indeed, speaking tends to be less deliberative than writing; so writing could allow people to carefully craft arguments that mitigate conflict escalation^32^. Further, our own data (see Study 6) finds that an overwhelming majority of people—84%—prefer to write more than speak when they expect to encounter disagreement. Thus, although some prior findings suggest speaking could improve constructive disagreement, the diverging preferences of our own participants and ambiguity of prior research indicate it is still unclear exactly how communication medium affects potentially heated disagreements.

One way in which the medium could affect the course of a disagreement is by changing people’s conversational behavior. In particular, past work has identified conversational receptiveness (i.e., the behavior in conversation that conveys thoughtful consideration of opposing views) as a set of linguistic techniques that build shared understanding and prevent conflict escalation^48–52^. We propose that the medium of conversation shapes counterparts’ linguistic receptiveness, affecting argument dynamics in both speech and writing. Yet, receptiveness may function differently in speech than writing in two ways. First, the linguistic markers of receptiveness may have a larger (or smaller) impact on conversation outcomes when spoken aloud. Because speech allows for nonverbal information, the effect of linguistic information may be blunted or inflected. In other words, receptiveness may be communicated in speech through how one sounds, not just what one says. Second, conversation medium may affect the average level of receptiveness. For example, perhaps when the medium allows for nonverbal information, people express comparatively less receptiveness via their linguistic content. The extra information from nonverbal cues might also promote common ground and empathy, which could instead increase the receptiveness that disagreeing counterparts use with one another. We compare these possibilities here, as part of a larger investigation into the behavioral mechanisms of the effect of conversation medium on constructive disagreement.

To conceptualize what makes a disagreement constructive, we particularly focus on two relevant conversation outcomes: perceived understanding and conflict. Understanding is a ubiquitous facet of conversation, as people typically strive to understand and be understood by their counterparts during their conversations^53^. But in the context of disagreements, partisans persistently misunderstand their opponents’ positions and motives, potentially to their own detriment because having information about the other party (e.g., their interests, options) can lead to more mutually beneficial agreements^23,54–61^. A second aspect of constructive disagreement is experiencing less conflict. Whereas disagreement is a mere misalignment in beliefs, conflict encompasses the emotional and relational consequences of disagreement. Prior research finds that “conflict spirals,” whereby disagreement gradually escalates into incivility over the course of an interaction, leading to negative emotions and mistrust between counterparts, can harm disagreement outcomes^17,47,62^.

In conjunction, then, we suggest that disagreement can be considered constructive when conversation partners achieve relatively higher understanding and experience lower conflict, even if their attitudes do not ultimately align. Understanding and conflict are distinct outcomes; for example, conflict can be minimized by avoiding a conversation entirely, but this will never improve understanding. Of course, there are other conversation outcomes that could be associated with constructive disagreement, but we think that understanding and conflict are the most immediately relevant to the conversation itself^33,63,64^. Still, we measure other possible outcomes in our studies—including changes in communicators’ attitudes, social perceptions and humanization of one’s conversation partner, and conversation enjoyment—and tend to find these outcomes correlate with perceived understanding and conflict.

Our experiments test how conversation medium affects the outcomes of disagreement conversations. In Studies 1 and 2A-C, we randomly assign dyads who disagree with one another to a conversation medium—either speaking or writing—to discuss an issue they disagree about. We identified issues that would be polarizing among our participant samples based on pilot data; topics range from the legalization of drugs to reparations for slavery. In addition to manipulating the conversation medium, Studies 2A-C orthogonally manipulate aspects of conversations that usually covary with medium: their interactivity (Study 2 A), length (Study 2B), and synchronicity (Study 2 C). In so doing, we can test whether these aspects moderate the main effect of the medium and whether they have their own independent effects on constructive disagreement. Finally, Study 3 conceptually replicates the effect of medium observed in earlier studies using three different participant samples outside of the laboratory. In all studies, we measure the proposed primary components of constructive disagreement, perceived understanding and conflict, hypothesizing that speaking will generate more understanding and less conflict than writing. In Study 3, we also measure objective understanding, operationalized as the accuracy of people’s predictions about their conversation partner’s position stance.

We investigate the mechanisms underlying why conversation medium changes disagreement outcomes in several ways. First, we investigate potential moderators of the main effect by manipulating various structural components of conversations that could co-occur with medium (i.e., interactivity, length, and synchronicity in Study 2), as well as measuring individual differences across participants (e.g., age, gender; see Studies 1–3 Pooled Results section). Second, we disentangle the joint effects of the medium on the production of content (speaking vs. writing) and the consumption of content (hearing vs. listening) by manipulating each separately (in Study 4). Third, we use natural language processing to analyze the linguistic choices made during these conversations, to show how medium changes linguistic content, even after controlling for differences in the amount of content (see Study 5).

A final study on people’s lay beliefs (Study 6) suggests that people misunderstand the effect of medium on constructive disagreement. Given that people can typically control not just the language they use in conversation, but also the medium in which that conversation is conducted, they may not choose the optimal medium for constructive disagreement. They may also fail to appreciate the ways in which their linguistic choices and the effects of those choices on their counterparts differ across media. Combined, these results have important consequences for how people in disagreement can get along with each other better.

## Results

We use a common analytical strategy across all studies. We test the effects of condition on our dependent variables using linear regressions estimated with the “multiwayvcov” package in R, which implements multiway clustered standard errors to account for dependence within dyads and sessions where participants have a single conversation (i.e., Studies 1−2). Specifically, we cluster standard errors at the dyad and session levels, controlling for topic fixed effects. In Study 3, where participants have multiple conversations, we also cluster standard errors by individuals. We evaluate statistical significance using two-tailed t-tests. We confirm all our results are robust when additionally controlling for age, gender, and strength of position. To help interpret the non-significant results, we additionally report 95% confidence intervals for these and all other statistical tests. See 10.17605/OSF.IO/C3ZGY for all pre-registrations, and Supplementary Note 1 for a discussion of any deviations from the pre-registrations.

### Study 1: The effects of conversation medium (video-chatting, speaking, and writing) on constructive disagreement

Supporting the hypothesis that spoken conversation leads to more constructive disagreement than written conversation, our Study 1 participants reported higher perceived understanding in the speaking (*M* = 4.95, SD = 0.95) and video-chatting (*M* = 4.79, SD = 1.07) conditions than in the writing condition (*M* = 4.30, SD = 1.25). (Speaking vs. writing: standardized *β* = 0.54, SE = 0.09, *t*(287) = 5.91, *p* <0.001, 95% CI [0.36, 0.72]; Video-chatting vs. writing: standardized *β* = 0.42, SE = 0.04, *t*(287) = 10.2, *p* <0.001, 95% CI [0.33, 0.50]). Reported understanding was also, unexpectedly, higher in the speaking than video-chatting condition (standardized *β* = 0.13, SE = 0.06, *t*(287) = 2.04, *p* = 0.043, 95% CI [0.00, 0.25]). Because our primary interest is in comparing speaking with writing, we aggregated the video-chatting and speaking conditions into a single “spoken conversation” condition and found a main effect of speaking versus writing on perceived understanding (standardized *β* = 0.48, SE = 0.07, *t*(288) = 7.04, *p* <0.001, 95% CI [0.35, 0.62]).

Perceived conflict was lower in the speaking (*M* = 1.51, SD = 1.12) and video-chatting conditions (*M* = 1.41, SD = 1.11) than in the writing condition (*M* = 1.93, SD = 1.19). (Speaking vs. writing: standardized *β* = −0.36, SE = 0.11, *t*(287) = −3.38, *p* = 0.001, 95% CI [-0.57,-0.15]; Video-chatting vs. writing: standardized *β* = −0.47, SE = 0.13, *t*(287) = −3.75, *p* <0.001, 95% CI [−0.71, −0.22]). There was no statistically significant difference in perceived conflict between the speaking and video-chatting conditions, standardized *β* = −0.11, SE = 0.12, *t*(287) = −0.90, *p* = 0.368, 95% CI [−0.35, 0.13]. When we aggregate the video-chatting and speaking conditions into a single “spoken conversation” condition, we find a main effect of speaking versus writing (standardized *β* = −0.41, SE = 0.09, *t*(288) = −4.39, *p* <0.001, 95% CI [−0.59, −0.23]).

**Fig. 1:**
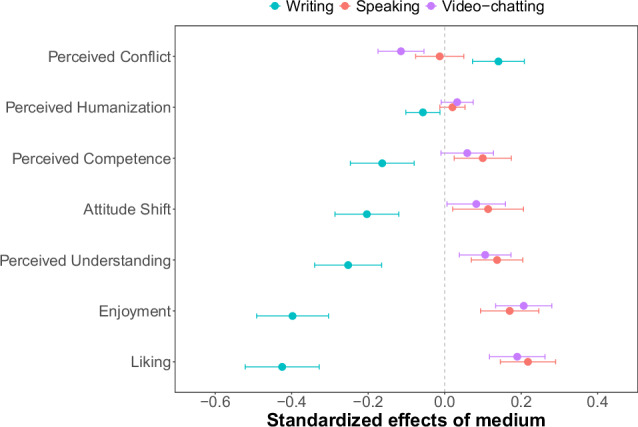
Effects of conversation medium (writing, speaking, or video-chatting) on conversation outcome measures (perceived conflict, perceived humanization and competence of one’s partner, attitude shift, perceived understanding, enjoyment of conversation, and liking of one’s partner) in Study 1. The horizontal axis shows variations in participant attitudes on standardized outcome measures by conversation medium. All data points represent group means, and error bars show the standard errors of the means. The variables are ordered in this plot from the most negative to the most positive effect size. Colors on the chart depict communication medium conditions, where green indicates writing and red indicates speaking. *N* = 292 participants.

Figure 1 plots the effects of conversation medium on perceived understanding, conflict, and other outcome measures (see Supplementary Note 2 for the pooled analysis of all outcome measures across Studies 1–3).

In summary, being randomly assigned to a conversation medium in Study 1 affected both perceived understanding and experienced conflict among pairs who disagreed on relevant, controversial topics. More specifically, disagreeing pairs who spoke to each other felt greater understanding and experienced less conflict than those who wrote to one another. Speaking additionally improved several related outcomes (e.g., higher impressions of one’s partner, greater conversation enjoyment) relative to writing.

We did not observe any improvement in understanding or reduction in conflict among pairs who video-chatted compared with those who spoke together without video. On the contrary, pairs reported slightly less understanding when video-chatting than speaking off-video. Given the additional visual information available when video-chatting, this result could be surprising but must be interpreted with caution. It is possible that the video quality was not optimal; moreover, video conversations are probably less nuanced than face-to-face, in-person conversations, which Study 1 did not test (but see Study 2A).

Several questions remain about our focal effect, such as why spoken media may enhance constructive dissent and whether these results are generalizable to different conversation contexts. We examine these questions further in Study 2.

### Study 2: Robustness across other aspects of conversation structure

Study 2 again tests the effect of conversation medium on constructive disagreement while also manipulating different structures of conversation, specifically the level of interactivity (Study 2A), length (Study 2B), and synchronicity (Study 2C) of the conversation. Our analyses follow the same analytic strategy described in Study 1, clustering standard errors by dyad and session and controlling for topic using fixed effects. In each of Studies 2A, 2B, and 2C, perceived understanding was statistically significantly higher when participants were speaking to each other (2A: *M* = 4.82, SD = 1.06; 2B: *M* = 3.73, SD = 1.02; 2C: *M* = 3.61, SD = 1.04) than when they were writing to each other (2A: *M* = 4.15, SD = 1.29; 2B: *M* = 3.41, SD = 1.17; 2C: *M* = 3.19, SD = 1.19) (2A: standardized *β* = 0.53, SE = 0.19, *t*(366) = 2.83, *p* = 0.005, 95% CI [0.16, 0.90]; 2B: standardized *β* = 0.29, SE = 0.09, *t*(406) = 3.13, *p* = 0.002, 95% CI [0.11, 0.47]; 2C: standardized *β* = 0.33, SE = 0.10, *t*(410) = 3.29, *p* = 0.001, 95% CI [0.13, 0.53]). The effects on perceived conflict were less consistent. Perceived conflict was statistically significantly lower when speaking (*M* = 0.58, SD = 1.03) than writing (*M* = 1.05, SD = 1.30) in Study 2C (standardized *β* = −0.44, SE = 0.12, t(410) = −3.56, p <0.001, 95% CI [−0.68, −0.19]), but not statistically significantly different in Studies 2A and 2B (Study 2A: speaking *M* = 1.31, SD = 1.11; writing *M* = 1.22, SD = 1.21; standardized *β* = 0.05, SE = 0.07, *t*(366) = 0.71, *p* = 0.478, 95% CI [−0.08, 0.18]; Study 2B: speaking *M* = 0.54, SD = 1.15; writing *M* = 0.67, SD = 1.25; standardized *β* = −0.11, SE = 0.18, *t*(406) = −0.57, *p* = 0.566, 95% CI [−0.46, 0.25]).

To interpret the results with greater statistical power, we pooled the data from all three experiments in Study 2, using the same analytic approach but additionally controlling for study number as a fixed effect. We find an overall effect of conversation medium such that understanding was higher when speaking (*M* = 4.02, SD = 1.17) than when writing (*M* = 3.57, SD = 1.28; standardized *β* = 0.38, SE = 0.07, *t*(1186) = 5.60, *p* <0.001, 95% CI [0.24, 0.51]), whereas perceived conflict was lower when speaking (*M* = 0.79, SD = 1.15) than writing (*M* = 0.97, SD = 1.27; standardized *β* = −0.16, SE = 0.07, *t*(1186) = −2.38, *p* = 0.018, 95% CI [−0.30, −0.03]).

**Fig. 2:**
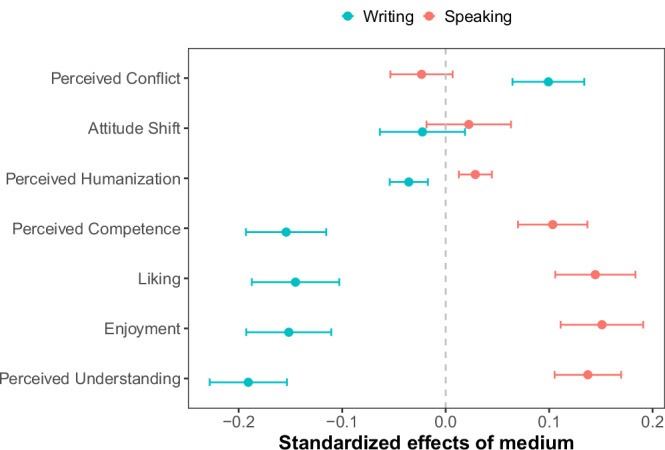
Effect of conversation medium (speaking or writing) on conversation outcome measures (perceived conflict, attitude shift, perceived humanization, competence, and liking of one’s partner, enjoyment of conversation, and perceived understanding) across Studies 2A-C. The horizontal axis shows variations in participant attitudes on standardized outcome measures by conversation medium. All data points represent group means, and error bars show the standard error of the mean. The variables are ordered in this plot from the most negative to the most positive effect size. Colors on the chart depict communication medium conditions where green indicates writing and red indicates speaking. *N* = 1194 participants.

Figure 2 plots the effects of conversation medium on perceived understanding, conflict, and other outcome measures (see Supplementary Note 2 for the pooled analysis of all outcome measures across Studies 1–3).

To test the robustness of the main effect of medium across each conversation structure separately, we used the same regression models as above, but additionally included conversation structure (i.e., the interactivity, length, and synchronicity manipulations) as a main effect alongside the main effect of medium, and added an interaction term between medium and conversation structure. Standard errors were clustered by pair and session, and topic was included as a fixed effect.

For Study 2A, testing the full experimental design of 2 (conversation medium: speaking or writing) × 2 (conversation interactivity: single exchange or multiple exchange), the main effect of medium on perceived understanding was statistically significant (standardized *β* = 0.54, SE = 0.24, *t*(364) = 2.24, *p* = 0.026, 95% CI [0.07, 1.02]) but the main effect of interactivity was not statistically significant (standardized *β* = −0.06, SE = 0.19, *t*(364) = −0.30, *p* = 0.765, 95% CI [–0.42, 0.31]), nor was the interaction between medium and interactivity (standardized *β* = −0.03, SE = 0.20, *t*(364) = −0.18, *p* = 0.860, 95% CI [–0.42, 0.35]). All effects on perceived conflict were not statistically significant: the main effect of medium (standardized *β* = 0.16, SE = 0.14, *t*(364) = 1.18, *p* = 0.237, 95% CI [–0.11, 0.43]), interactivity (standardized *β* = −0.07, SE = 0.22, *t*(364) = −0.31, *p* = 0.754, 95% CI [–0.51, 0.37]), and the interaction between medium and interactivity (standardized *β* = −0.29, SE = 0.27, *t*(364) = −1.04, *p* = 0.299, 95% CI [–0.82, 0.25]).

For Study 2B, testing the full experimental design of 2 (conversation medium: speaking or writing) × 2 (conversation length: short or long), we found a statistically significant main effect of medium on perceived understanding (standardized *β* = 0.36, SE = 0.08, *t*(404) = 4.59, *p* <0.001, 95% CI [0.21, 0.51]), but the main effect of conversation length was not statistically significant (standardized *β* = 0.02, SE = 0.10, *t*(404) = 0.20, *p* = 0.845, 95% CI [–0.22, 0.18]), nor was the interaction between medium and length (*β* = −0.14, SE = 0.21, t(404) = −0.68, *p* = 0.495, 95% CI [−0.56, 0.27]). All effects on perceived conflict were not statistically significant: the main effect of medium (standardized *β* = −0.02, SE = 0.20, t(404) = −0.10, *p* = 0.924, 95% CI [–0.40, 0.37]), length (standardized *β* = 0.26, SE = 0.17, *t*(404) = 1.49 *p* = 0.136, 95% CI [–0.08, 0.60]), and their interaction (*β* = −0.17, SE = 0.25, *t*(404) = −0.69, *p* = 0.488, 95% CI [–0.65, 0.31]).

For Study 2C, testing the full experimental design of 2 (conversation medium: speaking or writing) × 2 (conversation synchronicity: synchronous or asynchronous), the main effect of medium on perceived understanding was statistically significant (standardized *β* = 0.46, SE = 0.10, *t*(408) = 4.80, *p* <0.001, 95% CI [0.27, 0.65]), but there was no statistically significant effect of synchronicity (standardized *β* = −0.09, SE = 0.19, *t*(408) = −0.48, *p* = 0.629, 95% CI [–0.47, 0.28]), nor interaction between medium and synchronicity (standardized *β* = −0.24, SE = 0.25, *t*(408) = −0.96, *p* = 0.336, 95% CI [–0.74, 0.25]). We found a statistically significant effect of medium on perceived conflict (standardized *β* = −0.56, SE = 0.15, *t*(408) = −3.67, *p* <0.001, 95% CI [−0.85, −0.26]), but the effect of synchronicity was not statistically significant (standardized *β* = −0.15, SE = 0.13, *t*(408) = −1.20, *p* = 0.231, 95% CI [–0.40, 0.10]), nor was the interaction between medium and synchronicity (*β* = 0.23, SE = 0.27, *t*(408) = 0.84, *p* = 0.404, 95% CI [–0.30, 0.75]).

As reported above, we did not observe statistically significant interactions between conversation medium and the conversation structure manipulations in Studies 2A-C. However, sensitivity power analyses show that these studies were underpowered to detect the interaction effects of interest. The minimum detectable interaction effects were much larger than the observed estimates (minimum detectable βs: 2A = 1.80, 2B = 1.10, 2C = 1.25; observed βs: 2A = 0.03, 2B = 0.14, 2C = 0.24). As a result, the absence of statistically significant interactions should not be interpreted as evidence that the effect of medium is invariant across conversation structures (see Supplementary Note 3). Accordingly, Studies 2A–C primarily serve a stimulus-sampling function, demonstrating that the main effect of medium on constructive disagreement outcomes replicates across conversational contexts.

### Study 3: Beyond the laboratory

Study 3 employs a field context across three campus sites (University of California, Berkeley; Minnesota State University, Mankato; Arizona State University) wherein we randomly assigned participants to have multiple conversations with different partners (one-on-one) by either writing or speaking. Given the multiple conversations, we estimated the effect of medium on perceived understanding and conflict in a pooled regression, using the combined data collected from all three campus sites. We count each person in each conversation as an observation. We tested our hypotheses using linear regressions and two-sided t-tests, clustering standard errors by individual and dyad, controlling for campus site.

To first conceptually replicate the analyses from those earlier studies, we estimated the effect of medium within a subsample of those pairs who initially disagreed with each other (again defined as position stances that differ by at least three Likert scale points on the pre-survey; this analysis includes *N* = 52 conversations and 104 post-surveys). Our results were consistent with the findings from Studies 1 and 2: Among the initially disagreeing pairs, in the pooled regression across the three campus sites, perceived understanding was higher in spoken (*M* = 4.27, SD = 0.77) than written (*M* = 3.21, SD = 0.98) conversations (standardized *β* = 1.00, SE = 0.16, *t*(100) = 6.38, *p* <0.001, 95% CI = [0.69, 1.30]), whereas perceived conflict was lower in spoken (*M* = 0.22, SD = 1.64) than written (*M* = 1.17, SD = 1.74) conversations (standardized *β* = −0.47, SE = 0.19, *t*(100) = −2.47, *p* = 0.015, 95% CI [−0.83, −0.10]). Following our preregistration, in a robustness analysis we find the effects are still statistically significant when controlling for whether or not participants knew their partner and participants’ age and gender on perceived understanding: standardized *β* = 1.01, SE = 0.14, *t*(96) = 7.12, *p* <0.001, 95% CI [0.73, 1.29]; conflict: standardized *β* = −0.48, SE = 0.17, *t*(96) = −2.79, *p* = 0.006, 95% CI [−0.82, −0.14].

**Fig. 3:**
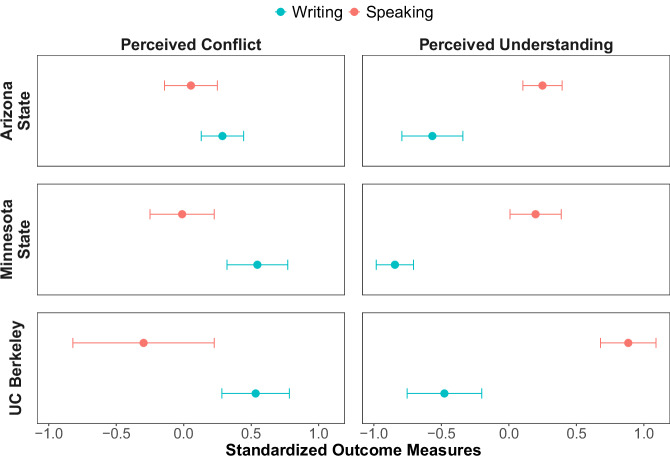
Differences in perceived conflict and understanding in Study 3 by campus site among dyads who initially disagreed. All data points represent group means. Error bars show the standard error of the means. Colors on chart depict communication medium conditions where green indicates writing and red indicates speaking. *N* = 104 post-surveys.

To assess whether the effect of conversation medium on perceived understanding and conflict varied by campus site, we conducted ANOVAs with the interaction term of conversation medium × campus site. The interaction effects were not statistically significant for perceived understanding, *F*(2, 98) = 0.54, *p* = 0.585, or for perceived conflict, *F*(2, 98) = 0.53, *p* = 0.588. See Fig. 3 to visualize the effects of conversation medium on perceived understanding and conflict by campus site among initially disagreeing dyads.

We next sought to test the effect of conversation medium among agreeing dyads only. Unlike the samples in Studies 1 and 2, we had enough pairs who agreed with each other (i.e., who reported their opinions on the same side of the attitude scales in the pre-survey, e.g., both supporting or both opposing the discussed issue) to analyze the treatment effect among these pairs. This analysis includes 127 conversations (254 post-surveys). Using the same regression model structure with standard errors clustered at individual and dyad level, we found again that speaking (versus writing) increased perceived understanding (standardized *β* = 0.47, SE = 0.12, *t*(251) = 3.90, *p* <0.001, 95% CI [0.24, 0.71]) and reduced perceived conflict (standardized *β* = −0.26, SE = 0.12, *t*(251) = −2.23, *p* = 0.027, 95% CI [−0.49, −0.03]).

In two campus sites, we preregistered that we would test whether the initial distance between conversation partners’ attitudes moderated the effect of medium on perceived understanding and conflict (including both agreement and disagreement conversations in this analysis, *N* = 179 conversations, 358 post-surveys). In these analyses, perceived understanding was still higher (standardized *β* = 0.61, SE = 0.09, t(353) = 6.47, *p* <0.001, 95% CI [0.43, 0.80]), and perceived conflict was also lower (standardized *β* = −0.31, SE = 0.10, *t*(353) = −3.10, *p* = 0.002, 95% CI [−0.51, −0.12]) in the speaking than writing conditions. Furthermore, there were no statistically significant interactions between initial attitude difference and medium on perceived understanding (standardized *β* = 0.10, SE = 0.06, *t*(353) = 1.75, *p* = 0.081, 95% CI [−0.01, 0.22]) or perceived conflict (standardized *β* = −0.09, SE = 0.06, *t*(351) = −1.40, *p* = 0.161, 95% CI [−0.22, 0.04]).

Finally, in one of the campus sites (ASU), we were able to test for differences in actual understanding by collecting participants’ beliefs about their partner’s attitude on the discussed topic and comparing it to their partner’s actual attitude (as reported in the post-surveys after the conversation). Specifically, we calculated accuracy by taking the absolute difference between perceived and actual attitudes, wherein lower values reflect a smaller discrepancy and thus better accuracy. This analysis includes pairs who initially agreed, disagreed, and who did not complete a pre-survey (*N* = 142 post-surveys). Testing the effect of medium on this measure of accurate understanding, we found higher accuracy in spoken than written conversations (*β* = −0.58, SE = 0.14, *t*(140) = −4.23, *p* <0.001, 95% CI [−0.85, −0.31]). This result is robust when controlling for whether or not pairs knew each other (*β* = −0.58, SE = 0.14, *t*(139) = −4.23, *p* <0.001, 95% CI [−0.84, −0.31]). A moderation analysis found the effect of medium on accuracy did not significantly vary based on the amount of initial disagreement in the dyad (including only pairs who both completed a pre-survey, *N* = 134 post-surveys; interaction term: *β* = 0.09, SE = 0.09, *t*(130) = 1.02, *p* = 0.312, 95% CI [−0.09, 0.27]).

In summary, Study 3 shows that the effects of medium on perceived understanding and conflict still held when participants wrote or spoke to one another in close physical proximity with their conversation partners (e.g., even when writers were sitting in the same room and could see each other while communicating). There was an effect of medium not only when pairs initially disagreed with each other but also when they agreed. This further reinforces the importance of medium in influencing perceptions of understanding and conflict. We also found that the effect of the medium on perceived understanding is mirrored by an effect on actual understanding, suggesting that subjective understanding may signal objective understanding too. For additional analyses pertaining to the unique elements of Study 3, including effects over time (i.e., conversation round) and effects of campus site, see Supplementary Note 4.

### Studies 1–3 pooled results

In this section, we conduct a deeper investigation into the effects of conversation medium by pooling the datasets from Studies 1–3 together for increased precision and robustness (total *N* = 1,590 post-surveys; including only the initially disagreeing pairs from Study 3 to match the inclusion criteria in Studies 1 and 2).

Using the pooled datasets, we conduct two new sets of analyses. For both analyses, we clustered standard errors by dyad, individual, and session with topic and study number as fixed-effect controls. First, we test the effects of medium on perceived understanding and conflict, followed by the other collected outcome measures. Consistent with prior analyses, speaking increased perceived understanding (standardized *β* = 0.45, SE = 0.07, *t*(1578) = 6.53, *p* <0.001, 95% CI [0.31, 0.58]) and reduced perceived conflict (standardized *β* = −0.23, SE = 0.06, *t*(1578) = −3.66, *p* <0.001, 95% CI [−0.35, −0.11]). Conversation medium also had statistically significant effects across most of the secondary outcome measures we collected. Compared to written conversations, spoken conversations led participants to perceive more humanity (standardized *β* = 0.20, SE = 0.05, *t*(1477) = 3.88, *p* <0.001, 95% CI [0.10, 0.29]) and more competence (standardized *β* = 0.33, SE = 0.06, *t*(1578) = 5.67, *p* <0.001, 95% CI [0.21, 0.44]) in their counterparts. Participants who spoke also reported liking their counterpart more (standardized *β* = 0.27, SE = 0.04, *t*(1578) = 7.05, *p* <0.001, 95% CI [0.19, 0.34]), and had greater enjoyment of the conversation (standardized *β* = 0.37, SE = 0.06, *t*(1477) = 6.33, *p* <0.001, 95% CI [0.26, 0.49]). With respect to attitude change, participants’ attitudes shifted more towards their partner’s position after speaking than writing with their partner (i.e., how much their post-conversation attitude moved toward the other side of the Likert scale from their pre-conversation attitude; *β* = 0.15, SE = 0.06, *t*(1578) = 2.26, *p* = 0.024, 95% CI [0.02, 0.25]). The full regression analyses for each outcome measure are reported in Supplementary Note 2.

Second, we investigate whether the main effect of medium is moderated by either individual differences or other measured pre-conversation variables. In particular, we analyzed a series of potential pre-treatment differences among individuals, including age, gender, personality, ideology, issue strength, and differences in issue position with one’s partner. Personality and ideology data were not collected in Study 3. We find that when controlling for these variables, the difference between the writing and speaking conditions remained similar (and statistically significant) for both perceived understanding (standardized *β* = 0.40, SE = 0.06, *t*(1461) = 6.87, *p* <0.001, 95% CI [0.28, 0.51]) and perceived conflict (standardized *β* = −0.20, SE = 0.06, *t*(1461) = −3.17, *p* = 0.002, 95% CI [−0.33, −0.08]). When interacting these variables with treatment assignment, we find that none of these variables statistically significantly moderate our main effects (all *p*s > 0.05). The regression tables including individual difference variables are shown in Supplementary Note 5.

### Study 4: The effect of the consumed and produced medium on constructive disagreement

Study 4 used the recorded conversations from Study 2A to orthogonally manipulate whether a new set of observers read or listened to a conversation that had been originally spoken or written. Thus, the experimental design was a 2 (the original medium of production: speaking or writing) × 2 (observers’ medium of consumption: listening or reading) between-participants design. We again estimated linear regressions, including fixed effects, to control for observers’ assigned topic. Because the impression measures were collected separately for each observer, we clustered standard errors for those analyses at the dyad level.

First examining the effect of the production medium, we found that observers perceived the original pairs’ understanding to be higher when the conversation had been produced by speaking (*M* = 5.15, SD = 1.21) rather than writing (*M* = 4.45, SD = 1.23; standardized *β* = 0.55, SE = 0.09, *t*(88) = 6.43, *p* <0.001, 95% CI [0.38, 0.72]). Observers perceived conflict to be lower when the conversation was produced by speaking (*M* = 2.49, SD = 1.29) rather than writing (*M* = 2.80, SD = 1.30; standardized *β* = −0.24, SE = 0.08, *t*(88) = 3.01, *p* = 0.003, 95% CI [−0.40, −0.08]).

Next examining the effect of the consumption medium, there was not a statistically significant effect of observers listening to the conversations (*M* = 4.83, SD = 1.31) versus reading the conversations (*M* = 4.72, SD = 1.23) on perceived understanding (standardized *β* = .08, SE = 0.06, *t*(88) = 1.47, *p* = 0.146, 95% CI [−0.03, 0.20), but the listeners perceived the original communicators to have less conflict (*M* = 2.51, SD = 1.25) than did the readers (*M* = 2.79, SD = 1.34; standardized *β* = −0.21, SE = 0.06, *t*(88) = 3.60, *p* <0.001, 95% CI [−0.33, −0.10]).

Finally examining the interaction between production and consumption medium, there were statistically significant interactions on perceived understanding (standardized *β* = 0.32, SE = 0.11, *t*(88) = 3.02, *p* = 0.003, 95% CI [0.11, 0.53]) and conflict (standardized *β* = −0.31, SE = 0.11, *t*(88) = 2.73, *p* = 0.007, 95% CI [−0.54, −0.08]).

Specifically, the medium of consumption had a larger effect when the conversation was originally spoken (understanding: standardized *β* = 0.28, SE = 0.07, *t*(41) = 4.00, *p* <0.001, 95% CI [0.14, 0.42]; conflict: standardized *β* = −0.38, SE = 0.08, *t*(41) = −4.81, *p* <0.001, 95% CI [−0.54, −0.22]) than when the conversation was originally written (understanding: standardized *β* = 0.06, SE = 0.09, *t*(46) = 0.69, *p* = 0.494, 95% CI [−0.11, 0.23]; conflict: standardized *β* = .07, SE = 0.08, *t*(46) = 0.90, *p* = 0.371, 95% CI [−0.09, 0.24]).

**Fig. 4:**
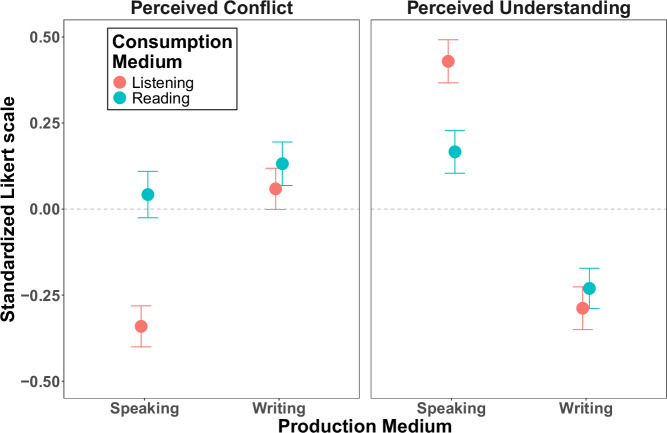
Effect of consumed and produced medium on perceived conflict and understanding in Study 4. Stimuli are the single-exchange conversations in Study 2 A. All data points represent group means. Error bars show the standard error of the means. Colors on chart depict consumption medium conditions where green indicates reading and red indicates listening. *N* = 971 participants.

To better understand the pattern of these interactions, shown in Fig. 4, we conducted two sets of simple effect analysis. First, when conversations were originally spoken, consuming the conversation by listening (rather than reading) led to higher perceived understanding (standardized *β* = 0.28, SE = 0.07, *t*(41) = 4.00, *p* <0.001, 95% CI [0.14, 0.42]) and lower perceived conflict (standardized *β* = −0.38, SE = 0.08, *t*(41) = − 4.81, *p* <0.001, 95% CI [−0.54, −0.22]), but when conversations were originally written, consumption medium did not statistically significantly affect perceived understanding (standardized *β* = 0.06, SE = 0.09, *t*(46) = 0.69, *p* = 0.494, 95% CI [−0.11, 0.23]) or perceived conflict (standardized *β* = 0.07, SE = 0.08, *t*(46) = 0.90, *p* = 0.371, 95% CI [−0.09, 0.24]). Second, when conversations were consumed by listening, those that were originally spoken (vs. written) seemed to show higher understanding (standardized *β* = 0.69, SE = 0.10, *t*(88) = 6.98, *p* <0.001, 95% CI [0.50, 0.89]) and lower perceived conflict (standardized *β* = −0.40, SE = 0.09, *t*(88) = − 4.21, p <0.001, 95% CI [−0.59, −0.21]), and when conversations were consumed by reading, there was also higher understanding for spoken than written conversations (standardized *β* = 0.41, SE = 0.10, *t*(88) = 3.99, *p* <0.001, 95% CI [0.21, 0.62]), but no effect of production medium on perceived conflict (standardized *β* = −0.09, SE = 0.10, *t*(88) = −0.90, *p* = 0.373, 95% CI [−0.29, 0.11]).

For results on all dependent variables, see Supplementary Note 6.

In summary, Study 4 suggests the medium of conversation can influence constructive disagreement through two primary pathways: changing which words a communicator selects when speaking or writing (i.e., the production of semantic content) or changing how even the same words are interpreted when heard or read (i.e., the consumption of semantic content). These results are consistent with prior research, which identifies several dimensions on which the content of spoken vs written language differs (e.g., formality^32^) and suggests that speech can humanize written text^33^.

But extending prior work, we also found statistically significant interactions between the produced and consumed media on constructive disagreement. Whether the consumption medium (listening or reading) had an effect on perceived understanding or conflict depended on how the messages were produced. In particular, spoken statements that were heard by observers (i.e., in the “produced-by-speaking and consumed-by-listening” condition) were deemed to create the most understanding and least conflict. Overall, this pattern of results suggests that conversation medium can affect both how language is produced and consumed, broadly consistent with media richness theory and its updates^25–30^. In the next section of this paper, we investigate in finer detail how conversation content affects perceived understanding and conflict.

### Study 5: Disagreement conversation language analyses

Study 5 sought to specifically analyze the effect of medium on the language in the 699 disagreement conversations recorded in Studies 1 and 2. Speaking is faster than writing, and so spoken conversations naturally contain more words (*M* = 616, SD = 10.0) than written ones (*M* = 190, SD = 3.17; standardized *β* = 1.43, SE = 0.08, *t*(1389) = 18.56, *p* <0.001, 95% CI [1.28, 1.58]). After controlling for word count and topic, we replicate earlier findings wherein perceived understanding is higher in speaking than in writing (standardized *β* = 0.35, SE = 0.09, *t*(1388) = 4.03, p <0.001, 95% CI [0.18, 0.51]) and perceived conflict is lower in speaking than in writing (standardized *β* = −0.32, SE = 0.08, *t*(1388) = −3.85, *p* <0.001, 95% CI [−0.48, −0.16]). Using a mediation test, we also confirm that none of the effects of medium on conversational outcomes were statistically significantly mediated by word count (all *p*s > 0.5). These results, in conjunction with those of Study 2B, where we manipulated conversation length, reassure us that our effects are driven not merely by the amount of conversation, but the actual content of that conversation.

**Fig. 5:**
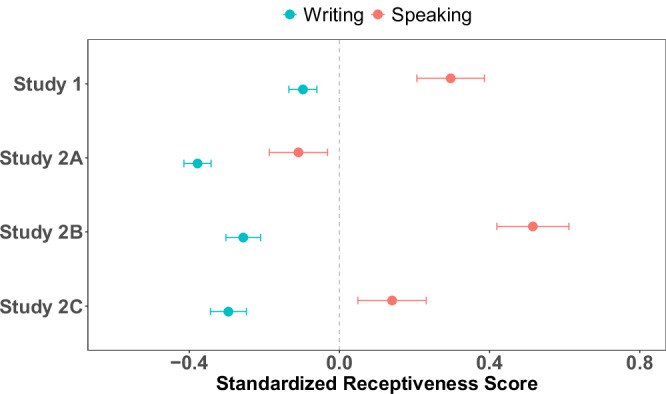
Effect of conversation medium (speaking or writing) on conversational receptiveness in Studies 1 and 2. Receptiveness score was calculated from a pre-trained model of conversational receptiveness directly from the transcribed conversations. All data points represent group means for each cell, and error bars show the standard error of the mean. Colors on the chart depict communication medium conditions, where green indicates writing and red indicates speaking. *N* = 1398 participants.

We found that conversational receptiveness was higher in speaking than writing when controlling for the conversation topic (standardized *β* = 0.46, SE = 0.05, *t*(1389) = 9.15, p <0.001, 95% CI [0.36, 0.56]). The effect is robust when we also control for word count (standardized *β* = 0.20, SE = 0.08, *t*(1388) = 2.58, *p* = 0.010, 95% CI [0.05, 0.35]). We conducted regressions estimating the robustness of the effect of medium on receptiveness in Studies 2A, 2B, and 2C separately and found that in each study, conversational receptiveness was higher in the speaking than writing conditions with and without word count as controls (see Fig. 5 and Supplementary Note 9).

**Fig. 6:**
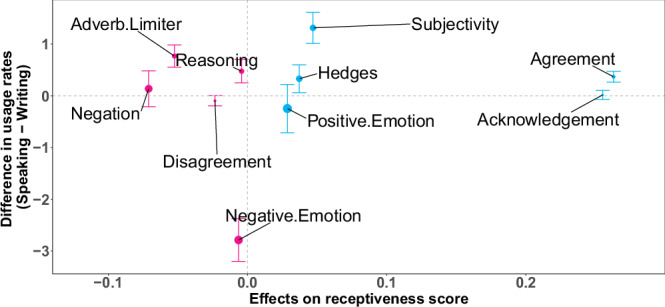
The relative contribution of different receptiveness features to the effect of conversation medium on overall receptiveness in Studies 1–2, as scored by the algorithm. The vertical axis shows the difference in feature usage in speaking relative to writing. The higher on the y-axis, the more that feature is used in speaking than writing. Conversely, the lower on the y-axis, the more that feature is used in writing than speaking. The x-axis shows the estimated effect of each feature on conversational receptiveness. All data points show group means; error bars show standard errors of receptiveness score. The size of the data points shows the total usage rate across all conversations when both speaking and writing. Colors on the chart depict expected effects on receptiveness, where purple indicates expected effects to be negative and blue indicates expected effects to be positive. Expected effects are based on prior study of linguistic features on rated receptiveness scores (Yeomans et al., 2018). *N* = 1398 participants.

Figure 6 shows the average feature usage in writing minus feature usage in speaking. The more a feature is used in speaking than writing, the higher it is on the y-axis of Fig. 6. On the x-axis, the linear coefficients from the pre-trained algorithm are shown; features associated with greater receptiveness are to the right, and those associated with lower receptiveness are to the left.

Three receptiveness features in particular showed either relatively large differences in usage between speaking and writing, or were relatively large predictors of receptiveness score, or both. We found that greater receptiveness scores in speaking were due to relatively greater use of subjectivity phrases (*β* = 1.31, SE = 0.28, *t*(1388) = 4.73, *p* <0.001, 95% CI [0.77, 1.85]) and agreement (*β* = 0.37, SE = 0.12, *t*(1388) = 3.20, *p* = 0.001, 95% CI [0.14, 0.59]), as well as relatively lesser use of negative emotions (*β* = −2.79, SE = 0.37, *t*(1388) = −7.44, *p* <0.001, 95% CI = [−3.52, −2.05]). Other features such as disagreement, acknowledgement, reasoning, and positive emotion did not show statistically significant differences were not used much differently across conditions. And some features actually went in the opposite direction of the general trend—for example, negations and adverb limiters were more common in speaking than writing, even though they reduce receptiveness (see Fig. 6).

**Fig. 7:**
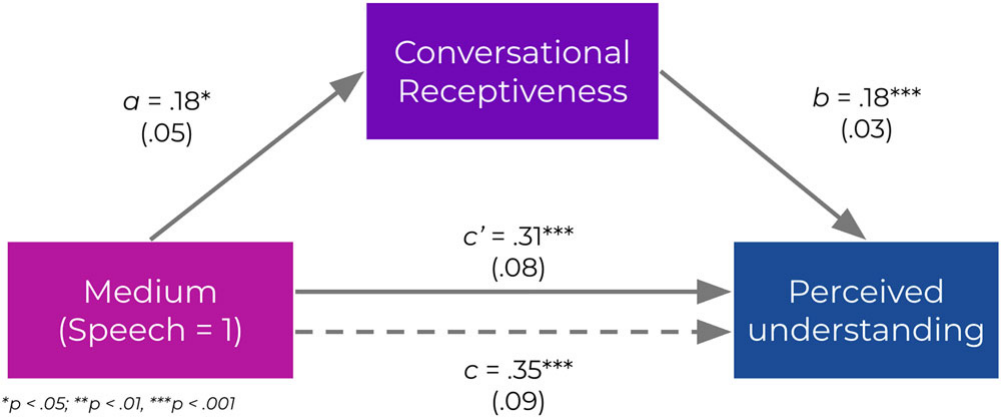
Mediation pathway showing each path effect using data collected in Studies 1 and 2. **a** shows the effect of conversation medium on conversational receptiveness; (**b**) shows the effect of conversational receptiveness on perceived understanding; (**c**) shows the total effect of conversation medium on perceived understanding; and (**c′**) shows the direct effect of conversation medium on perceived understanding. Effects are estimated using ordinary least squares linear regression with topic and study fixed effects. Standard errors are clustered at the dyad and session levels. Statistical tests are two-sided. No adjustments were made for multiple comparisons. Standard errors shown in parentheses. *N* = 1398 participants.

Figure 7 shows the mediation model testing whether receptiveness mediates the effect of medium on perceived understanding with direct (c′), total (c), and mediation pathways (a, b). The (c′) path shows that speaking (vs. writing) directly increases perceived understanding (standardized *β* = 0.31, SE = 0.08, *t*(1387) = 3.72, *p* <0.001, 95% CI [0.15, 0.48]), the (a) path shows that speech contained more receptive language than writing (standardized *β* = 0.18, SE = 0.08, *t*(1388) = 2.58, *p* = 0.010, 95% CI [0.05, 0.35]), the (b) path shows that conversational receptiveness is associated with greater perceived understanding (standardized *β* = 0.18, SE = 0.03, *t*(1388) = 6.14, *p* <0.001, 95% CI [0.12, 0.23]), and the (c) path shows the total effect of condition and language on understanding (standardized *β* = 0.35, SE = 0.09, t(1388) = 4.03, *p* <0.001, 95% CI [0.18, 0.51]). Together, the effect of the communication media on perceived understanding was partly mediated by the use of receptive language, such that the estimated indirect effect of the conversation medium on perceived understanding through receptiveness was 0.037 (*p* = 0.004, 95% CI [0.01, 0.06])–about 10% of the total effect.

We conducted these same analyses for all the common dependent variables. For perceived conflict, we found that the estimated indirect effect of conversation medium through receptiveness was statistically significant: 0.024 (*p* = 0.003, 95% CI: [0.05, 0.10])—about 7% of the total effect. We also observed statistically significant indirect effects for competence, liking, and conversation enjoyment—but not for humanization or attitude shift. Supplementary Note 10 shows the relationship for all pathways for all dependent variables.

**Fig. 8:**
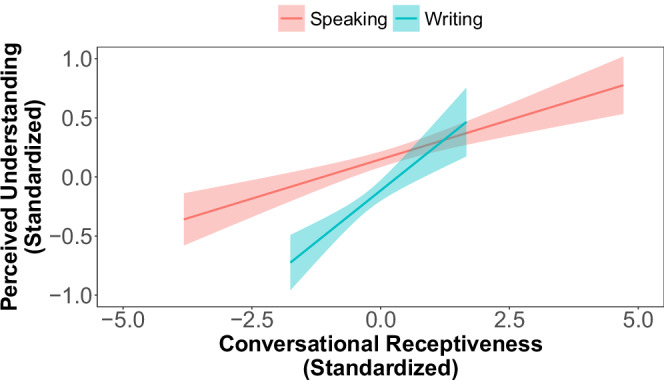
Moderation effect of speaking or writing on the relationship between conversational receptiveness and perceived understanding using data collected in Studies 1 and 2. Effects are estimated using ordinary least squares linear regression with topic and study fixed effects. Standard errors are clustered at the dyad and session levels. Statistical tests are two-sided. No adjustments were made for multiple comparisons. The solid lines show model-predicted mean perceived understanding as a function of conversational receptiveness for speaking and writing at the individual level. The shaded bands represent 95% confidence intervals around the predicted mean. Spoken conversations contain a wider range of receptiveness scores than written conversations, as spoken conversations contain more words. Colors on the chart depict communication medium conditions, where green indicates writing and red indicates speaking. *N* = 1398 participants.

Thus far, our analyses have assumed the effect of conversation medium on receptive language is similar in both speaking and writing. Examining the interaction for perceived understanding, our regression shows a strong relationship between receptive language and perceived understanding in written conversations (standardized *β* = 0.34, SE = 0.07, *t*(648) = 4.99, *p* <0.001, 95% CI [0.20, 0.47]), but a relatively weaker relationship in spoken conversations, albeit still different from zero (standardized *β* = 0.15, SE = 0.03, *t*(730) = 5.21, *p* <0.001, 95% CI [0.09, 0.20]); interaction effect: standardized *β* = −0.23, SE = 0.06, *t*(1386) = 3.60, *p* <0.001, 95% CI [−0.35, −0.10]). Thus, medium moderates the association between conversational receptiveness and perceived understanding (see Fig. 8), suggesting that receptive language use is more closely associated with felt understanding in writing than in speech. Examining the interaction for perceived conflict, our regression shows a strong relationship between receptive language and perceived understanding in written conversations (standardized *β* = −0.34, SE = 0.08, *t*(648) = −4.47, *p* <0.001, 95% CI [−0.49, −0.19]), but a relatively weaker relationship in spoken conversations, albeit still different from zero (standardized *β* = −0.07, SE = 0.03, *t*(730) = −2.64, *p* <0.001, 95% CI [−0.12, −0.02]); interaction effect: standardized *β* = 0.21, SE = 0.08, *t*(1386) = 2.82, *p* = 0.005, 95% CI [0.06, 0.36]). We found similar moderation patterns in the other dependent variables, which are reported in Supplementary Note 11.

Although we focus on differences in receptive language between speaking and writing, there are other potential language constructs that may also differ across conditions. We cannot possibly test them all—indeed, there are seemingly infinite ways to extract language features from text data^65^. However, we focus on three reasonable benchmarks to receptiveness: word count, sentiment, and politeness^66,67^ These benchmark measures are in fact correlated with receptiveness to varying degrees (word count: *r* = 0.27, *p* <0.001, 95% CI [0.22, 0.32]; sentiment: *r* = 0.25, *p* <0.001, 95% CI [0.20, 0.30]; politeness: *r* = 0.18, *p* <0.001, 95% CI [0.13, 0.23]). We conduct all our main analyses while including these three measures as controls, and report the full analyses in Supplementary Note 12. Even controlling for word count, sentiment, and politeness, there remains a positive association between receptiveness and perceived understanding (standardized *β* = 0.15, SE = 0.03, *t*(1382) = 4.60, *p* <0.001, 95% CI [0.08, 0.21]) and a negative association between receptiveness and perceived conflict (standardized *β* = −0.09, SE = 0.03, t(1382) = −3.51, *p* <0.001, 95% CI [−0.14, −0.04]). Furthermore, again controlling for word count, sentiment, and politeness, receptiveness is higher when speaking than writing, albeit not statistically significantly higher (standardized *β* = 0.13, SE = 0.08, *t*(1382) = 1.74, *p* = 0.082, 95% CI [−0.02, 0.28]).

In summary, Study 5’s results indicate that people engage in greater conversational receptiveness when speaking than writing, using more subjectivity phrases and expressing more agreement while also using fewer negative emotions in their language. Receptive language partially mediates the effect of medium on constructive disagreement outcomes (e.g., perceived understanding, conflict), accounting for about 10% of the total effect of medium. The conversation medium further moderated the association between receptive language and constructive outcomes, whereby conversational receptiveness was more tightly associated with constructive disagreement outcomes in written than spoken communication. There are many possible reasons for this result, including that people use less receptive language in writing than speech or that word choices matter more for constructive outcomes in writing than speech. We consider these possibilities further in the discussion.

### Study 6: The expected effects of conversation medium

Our final study examines people’s preferences and predictions about how the conversation medium will affect their disagreement outcomes. We tested the within-subject effects of medium on our outcome measures. In direct opposition to the results observed in Studies 1–3, the participants in Study 6 expected writing to produce less conflict (*M* = 3.79, SD = 0.27) than either speaking (*M* = 4.25, SD = 0.30; standardized *β* = −0.31, SE = 0.07, *t*(402) = −4.56, *p* <0.001, 95% CI [−0.45, −0.18]) or video-chatting (*M* = 4.33, SD = 0.31; *β* = −0.37, SE = 0.07, *t*(402) = −5.36, *p* <0.001, 95% CI [0.23, 0.50]). Participants also expected writing to produce more understanding (*M* = 4.22, SD = 0.30) than video-chatting (*M* = 4.04, SD = 0.29; standardized *β* = 0.13, SE = 0.06, *t*(402) = 2.04, *p* = 0.042, 95% CI [0.01, 0.25]) although not statistically significantly more than speaking (*M* = 4.06, SD = 0.29; standardized *β* = 0.11, SE = 0.06, *t*(402) = 1.81, *p* = 0.071, 95% CI [−0.01, 0.23]). We found no statistically significant differences between video-chatting and speaking for either predicted conflict (standardized *β* = 0.05, SE = 0.07, *t*(402) = 0.80, *p* = 0.426, 95% CI [−0.08, 0.19]) or understanding (standardized *β* = 0.01, SE = 0.06, *t*(402) = 0.23, *p* = 0.816, 95% CI [−0.11, 0.14]). Participants further predicted that speaking (vs. writing) would be less comfortable, more effortful, more awkward, less enjoyable, would make their impressions of their partner more positive, and less likely to shift the other person’s attitudes, all *p*s <0.05, but we did not find statistically significant effects of conversation medium on liking of one’s partner or perceptions of common ground (*p*s > 0.664). See Supplementary Note 13 for all predicted effects of medium, as well as exploratory interaction tests with demographic variables (wherein no statistically significant interaction effects emerged).

Consistent with their predictions, participants expressed a strong preference for communicating via writing (83.9%) rather than via speaking (7.3%) or video-chatting (8.9%; chi-squared(2) = 220, *p* <0.001). There was no statistically significant difference between the proportion of participants who selected speaking from those who selected video-chatting (chi-squared(1) = 0.29, *p* = 0.59).

Overall, Study 6 suggests that people expect written disagreements to yield more constructive outcomes, such as less conflict and more enjoyment, than spoken disagreements. These predictions and preferences stand in stark contrast with the results from our prior experiments, which instead indicate that speaking produces more constructive disagreement (for instance, by lowering perceived conflict, among other outcomes) than writing.

It should be noted that there were several differences between our predictors in this study and our experiencers in other studies: they were collected at different points in time (e.g., after versus before the COVID-19 pandemic for predictors and experiencers, respectively) and also come from different populations (e.g., online workers versus primarily college students for predictors and experiencers, respectively). Furthermore, in this study, predictors considered the conversation medium conditions within-subjects, whereas in previous studies, the medium was experienced in a single, between-subjects condition. Still, the contrast between the results here and our earlier experiments suggests that people may fundamentally misunderstand the role of conversation medium in their disagreement outcomes.

## Discussion

Conversations between opponents can be enormously valuable when they are constructive, with the potential to improve understanding and reduce conflict. What makes a disagreement conversation constructive? The current paper examines the effect of a common and consequential choice in communication, the medium of conversation, on disagreement conversation outcomes. Across a series of experiments, we find that when disagreeing counterparts have spoken compared to written conversations, they report greater understanding and less conflict, as well as higher impressions of each other, more common ground, and greater enjoyment of the conversation. This is in surprising contrast to the expectations of participants in Study 6, who on average preferred to write than speak with a disagreeing counterpart, and believed that spoken conversations would produce less constructive outcomes than written conversations.

Our experiments suggest that linguistic choices may play a role in shaping perceptions of conflict. One possible reason why communicators reported less conflict and greater understanding when speaking than writing is that they tended to use more receptive language. The increase in conversational receptiveness was particularly associated with a greater use of subjectivity phrases and a lower frequency of negative emotion phrases in speech compared to writing. Yet conversational receptiveness was also more strongly associated with constructive outcomes in writing than speech. This moderation result, contrasted with the treatment effect, presents something of a puzzle: Why do people use less receptive language in the very context where it might matter most? Perhaps receptive language has more impact in writing because it is used more rarely. Another possibility is that paralinguistic cues convey their own information about a communicator’s intent, which sometimes conflicts with linguistic cues. This would naturally lessen the relative impact of a communicator’s linguistic cues on constructive disagreement outcomes. We conduct some exploratory analyses of the auditory features of speech in Supplementary Note 14. Either way, future research could investigate this question further.

The current paper provides several theoretical contributions. Perhaps most directly, it extends existing, highly influential communication theories by providing additional insight into how the communication medium influences interpersonal consequences^25,26,30,68,69^. For one, the presence of human voice, via speech – compared to its absence, via writing—appears to be critical for changing conversational outcomes. In contrast, our data suggest that being able to see a communicator may be less critical, or else visual cues may simply be largely redundant with verbal cues in the contexts we examined. In Study 1, communicators reported nearly identical conversation outcomes when assigned to a video-chat conversation compared to an audio-only conversation, and in Study 3, even writers who could see each other reported less understanding and greater conflict than speakers reported. Another insight suggested by our studies is that the conversation medium changes outcomes both by changing how language is produced—whereby communicators make different linguistic choices, such as using more receptive words, when speaking than writing—as well as how it is consumed—whereby even the same words are interpreted more constructively when heard than read (as we show in Study 4 and Study 5).

A related theoretical contribution made by the present research is opening the “black box” of what happens in a conversation, by mapping changes in communicators’ linguistic approaches as a function of medium using state-of-the-art algorithms for analyzing language data, particularly conversational receptiveness. Whereas existing research demonstrates the features of conversational receptiveness and their effects on perceived conflict, our experiments suggest that a commonly faced decision when anticipating interpersonal conflict – the decision to communicate using a speech-based or text-based medium—influences the use of receptive language during the conversation, and ultimately changes perceived understanding and conflict by the end of the conversation^51,52^. We further test exactly which linguistic features are associated with different conversation media and constructive outcomes, for example, finding that spoken conversations use more subjective language and hedges than written conversations (consistent with prior research that speech is more ambiguous than writing)^34^. Thus, we provide insight into how communication medium influences both the level of conversation receptiveness and its association with disagreement outcomes.

There are also practical implications. First, with the proliferation of digital transformation initiatives post COVID-19, virtual communication (working remotely, socializing asynchronously) has become a new normal for many people. Advancements in communication technologies ensure that people have more communication platforms available to use than ever before, eliciting the necessity of selecting just one for a conversation and highlighting the importance of understanding which aspects of a platform affect conversation outcomes. Second, our work helps to expand a growing toolbox of methods to resolve and manage conflict^70,71^. Although a single conversation may not alter attitudes, the long-term benefits of constructive disagreement may lie in fostering a culture where differences are engaged rather than polarized.

Study 6 highlights the importance of conducting this research: our results are not intuitive. Participants who imagined communicating with a disagreeing partner had a stronger preference for writing than speaking to them, and tended to make the wrong predictions about how the medium would affect their conversation; for instance, estimating that there would be more understanding and less conflict in the written than spoken medium, when our experiments found the opposite results. This suggests that people don’t fully grasp how the communication medium affects their conversation outcomes, have trouble simulating a disagreement conversation, and/or do not care about maximizing constructive outcomes in disagreement^72^. People’s miscalibration in predicting disagreement outcomes may be one instantiation of a broader set of miscalibrated social decisions^73,74^. Regardless, the results of the current paper have the potential to be informative for people’s communication decisions in ways they may not expect.

The current findings point to several potential limitations. First, participants across experiments may have had different goals during their conversations; for example, in some conversations, participants’ language and/or tones suggested that they were not really interested in seeking out the other person’s perspectives as much as stating their own^63^. We think these varying goals are true to life, and did not try to control or measure them in the studies. But it is possible that participants’ goals could influence the effect of medium of conversation outcomes. Relatedly, we do not directly compare these conversations (exchanging opinions on controversial topics) to other types of conversations (such as friendly chatting). It is possible that talking produces more understanding than writing, even when the topic is not a controversial one. This limitation also applies to preferences over different media; although the Study 6 participants preferred to write when they expected to disagree with their counterpart, perhaps they would prefer a different medium for another type of conversation. Future research could unpack any potential interaction effects between conversation media, communicators’ goals, the type of conversation, and outcomes.

Second, the level of measured conflict was relatively low across all experiments and conditions (below the scale midpoint), suggesting that we may have missed situations in which conflict would be more pronounced. Indeed, our participants were mostly strangers, perhaps making the stakes lower for the conversation than they would have been with a closer relationship (e.g., family member). Future research could test whether the effect of medium would be different for people who know one another well, or who are disagreeing about something central to their relationship (e.g., about personal or workplace matters). It is possible that our results underestimate the true effects of communication medium on perceived conflict and understanding (e.g., it may be even more important to speak than write when the stakes are higher) or alternatively it is possible that extreme disagreement may be especially difficult to manage in speech. (In our data, we found main effects of conversation partners’ stance strength, but it did not interact with the conversation medium condition; see Supplementary Note 5).

Another limitation of this research is that Studies 1–3 were conducted primarily among college students at university. Although we did conceptually replicate the effects of medium on perceived understanding and conflict at several different American universities (in Study 3), providing somewhat more diversity in our samples, it is still worth testing these effects in other samples (e.g., older individuals who are non-American). It is possible that heterogeneous treatment effects would exist among different non-student populations. For example, perhaps people in some cultures or settings where there is a strong norm of indirectness would benefit less from spoken conversation. Other settings may also have a much wider span of possible disagreement (clashing with identity or cultural concerns) that might influence the treatment effect. We hope the current paper spurs further exploration of the effect of communication medium in all kinds of conversations, involving different people and topics, to better understand the phenomenon.

How can we change polarized, divisive, and harmful disagreements into mutual respect and thoughtful consideration? This is a critical question in an age of increasing polarization and misinformation. The current research points to a subtle but important feature of conversation that can make disagreement more constructive: the medium of conversation. Spoken conversations produce more receptive language, alongside greater perceived understanding and less experienced conflict, than written conversations during disagreement. These findings have implications for how communication technology may shape discourse. Conflict is born not just from disagreement but from the structure of a conversation itself.

## Methods

For all experiments, we have reported all measures, conditions, data exclusions, and sample sizes. All data, stimuli, analysis code, and preregistrations are posted on the Open Science Framework website at 10.17605/OSF.IO/C3ZGY. All studies included a preregistered analysis plan, except for Study 1, 2A, and one site in Study 3; links to the preregistrations are reported in the appropriate “Method” sections below, as well as in Supplementary Note 1. To reduce researcher degrees of freedom even in studies that were not preregistered and make this manuscript more readable, we apply consistent analyses when possible across studies (using the same exclusion criteria, regression modelling strategies, and outcome measures, unless explicitly noted). This led to some deviations in our preregistered analyses, which we report and discuss in Supplementary Note 1.

All studies reported in this manuscript were reviewed and approved by the Committee for Protection of Human Subjects (CPHS) at the University of California, Berkeley. The research was covered under a ten-year approval (Effective Date: March 12, 2018; Expiration Date: March 11, 2028), reviewed on an expedited basis under Categories 6 and 7 of the federal regulations. Approval letters from CPHS can be found: 10.17605/OSF.IO/C3ZGY. The described ethics approval covers all studies reported in the manuscript. We confirm that for all studies, participants provided informed consent prior to participation.

### Study 1: The effects of conversation medium (video-chatting, speaking, and writing) on constructive disagreement

This experiment tests the causal effect of three conversation media—video-chatting, speaking, and writing—on how constructively pairs disagree. Specifically, we selected pairs who reported disagreeing strongly on a controversial topic and randomly assigned them to discuss the topic for ten minutes via a specific conversation medium. We measured their attitudes both before and after the conversation, as well as other conversation experiences and opinions after the conversation. We hypothesized that the medium would affect pairs’ disagreement, such that pairs who spoke to one another (versus wrote to each other) would disagree more constructively: perceiving they understood each other more and experiencing less conflict. We further measured whether pairs would think more highly of each other and be more open to each other’s views. The conversations in Study 1 (and Study 2) were also recorded and transcribed for the purpose of conducting linguistic analyses.

### Participants

We aimed for at least 50 pairs per experimental condition, but over-recruited because we anticipated that not every individual would be able to be matched with someone who strongly disagreed with them. In total, we recruited 421 participants from the laboratory participant pool of the University of California, Berkeley. Participants were recruited individually and paired based on their disagreement regarding a conversation topic. We assumed most participants did not already know their partner, given that the laboratory participant pool contains over 10,000 people and, in other studies which used the same participant pool, fewer than 5% of random pairs reported knowing each other. Of the 421 recruited individuals, 25 (5.9%) could not participate in the study because either they could not be matched with a partner within their session (e.g., an odd number of participants) or were not fluent enough in English. Beyond the 25 individuals who could not continue the study, no other participants left after learning about their assignment to their experimental condition, allaying concerns about participants selecting out of conditions. Of those who participated, we removed data from 104 people (26.3%) from our analysis because they failed to meet our inclusion criteria: both partners had to feel strongly about, and have opposing preferences on, the topic of discussion (for details on these criteria, which were created for consistency across studies, see Supplementary Note 15). Table S20 in Supplementary Note 15 reports attrition at each stage across all studies. Attrition in later studies was lower than in Study 1 because we changed the laboratory matching procedure to be consistent with our analysis inclusion criteria. The final sample for Study 1 was 292 individuals (i.e., 146 dyads; 59.2% Female, 39.4% Male, *M* age = 21.6 years, SD age = 3.8 years) who participated in exchange for $15 each.

### Topic selection

We conducted a pilot survey of 13 topics from a different set of participants drawn from the same laboratory pool in which we intended to run the primary study, to select the most controversial topics to use for the study – specifically, topics on which around half of the participants supported and the other half opposed, and about which participants reported feeling strongly. See Supplementary Methods for details of the pilot survey and its results. Based on the pilot results, we selected three topics for the participants in the main study to discuss, all of which were relevant for the sample of undergraduate university students who would take the main study: changing the legal drinking age from 21 to 17 years old, using genetically modified food in the university cafeteria, and having a race quota for university admissions (see Supplementary Methods for full description of all topics).

### Experiment design

Dyads were randomly assigned at the lab session level to one of three conditions in a between-subjects experimental design: video-chatting, speaking, or writing. Participants in all conditions communicated using the same computer-mediated communication platform, called Skype, which was preloaded on each participant’s computer. We selected this platform because it has video-chatting, audio-only, and text-chat capacities. This experiment was not preregistered.

### Protocol

When participants entered the laboratory, they were seated at a computer station with dividers that prevented them from seeing other participants. Each laboratory session contained up to 20 participants. There were 52 sessions in total.

Participants first consented to participate and completed a private, online pre-survey in which they reported their position (opposed or supporting, on a six-point Likert response scale ranging from −3 to +3) and how strongly they felt about it (0 = not strongly; 1 = somewhat strongly; 2 = very strongly) on each of the three topics. Then, participants completed a private online personality survey, the Big-5 44-item survey, which was intended to serve as a “filler task” so that the research assistants could determine the (disagreeing) pairs for the conversation in the next part of the study^75^.

After participants completed their personality survey, they learned of their assigned partner and topic, which research assistants wrote on a whiteboard at the front of the room. A research assistant read aloud the study instructions to each dyad (see Supplementary Methods for the study instructions) and gave participants 2 min to prepare for their conversation. When the two minutes had passed, the research assistants walked around the room to ensure each participant had successfully logged onto Skype (in the correct conversation medium) and that the screen recordings had started. To focus participants on the topic, the conversation was structured. Each participant first took up to 3 min to make their initial position clear (i.e., up to 6 min total). Then, they were given three more minutes to jointly discuss, for a total of up to 9 min.

Once the conversation was complete, all participants completed a private, online post-survey in which they reported their conversation outcomes. In this and all other studies in this paper, our analyses focus on the post-survey measures most relevant for constructive disagreement: pairs’ perceptions of mutual understanding (2 items, *ɑ* = 0.67: “To what extent do you think your partner understood your position?” and “To what extent do you think you understood your partner’s position?”) answered on a seven-point Likert response scale from 0 (“did not understand at all”) to 6 (“understood extremely well”), and their assessment of conflict (4 items, *ɑ* = 0.76, e.g., “How much conflict did you feel during your conversation with your partner?” answered on a seven-point Likert response scale from 0 (“no conflict at all”) to 6 (“a great deal of conflict”)). The post-survey also measured participants’ perceptions of their conversation partner’s competence, liking of their partner, humanization of their partner, enjoyment of the conversation, experienced common ground, perceived agreement with their partner, actual attitudes on all three topics again, and demographic information. Finally, participants received payment for the study and were debriefed about the study hypotheses. More protocol details and the full text of each survey measure are in the Supplementary Methods.

### Study 2: Robustness across other aspects of conversation structure

Studies 2A-C were designed to test the robustness of the main effect demonstrated in Study 1. Each experiment employed a similar paradigm to the one in Study 1, with participants in a laboratory paired based on strong disagreement, having a conversation via a randomly assigned medium (and other randomly assigned conversation features), and then reporting their experiences using survey methodology. Because all dyads were randomly assigned to either speak or write to one another across the studies, and they each answered many of the same survey measures, we report the combined results below. To examine the robustness of the effect of conversation medium, each study not only manipulated medium but also manipulated one additional feature of conversation structure that typically covaries with medium. Specifically, Study 2A additionally manipulated conversation interactivity (with some dyads discussing topics back-and-forth, whereas others had a single exchange each), Study 2B manipulated conversation length (with some dyads having shorter conversations, whereas others had longer conversations), and Study 2C manipulated conversation synchronicity (with some dyads taking breaks between conversational turns whereas others had no response speed limitations).

### Study 2A participants

In total, we recruited 435 participants from the laboratory participant pool of the University of California, Berkeley. Of that set, 1 person (0.2%) could not participate in the study because they could not be matched with a partner within their session. Of those who participated, 60 (13.8%) were removed from the analysis due to our predetermined exclusion criteria, which were the same criteria described in Study 1 (participants had to feel strongly about the topic of discussion and have opposing preferences on it). Of those who were included in the study and began their conversation with their assigned partner, 4 (1.1%) left part way through the conversation; this attrition did not vary across conditions (chi-squared (4) = 3.31, *p* = 0.361). Our final sample included 370 individuals (185 dyads; 60.5% Female, 39.2% Male, *M* age = 20.82 years, SD age = 3.64 years) who participated in exchange for $15 each.

### Study 2A experiment design

Dyads were randomly assigned at the lab session level to one of four conditions in a 2 (conversation medium: speaking or writing) × 2 (conversation interactivity: single exchange or multiple exchange) between-subjects experimental design. Participants in the writing condition communicated using G-chat for multiple exchanges and Gmail for single exchanges (selected because these are popular texting platforms), whereas participants in the speaking conditions talked in person (face-to-face). This experiment was preregistered at 10.17605/OSF.IO/C3ZGY (see summary of preregistration deviations in Supplementary Note 1).

### Study 2A protocol

We used a similar procedure to the one described in Study 1. First, participants consented to the study and completed a private pre-survey reporting their attitudes on the same three topics as Study 1 regarding the college drinking age, use of GMOs in cafeterias, and undergraduate admission quotas (see full topic descriptions in Supplementary Methods). The experimenters downloaded participants’ pre-survey responses and used them to pair participants based on strong disagreement on a single topic while participants completed a private personality survey.

Next, participants had their conversation. In the single exchange condition, participants in both the speaking and writing conditions were given time to prepare statements on the assigned topic of conversation they would deliver to their partner. To control the total amount of time, speaking participants had about a minute to prepare their statements and three minutes each to deliver the statements verbally, whereas writing participants had almost five minutes to prepare (i.e., write their statements) and two minutes to read each other’s statements (thus taking about seven minutes total in each condition). One person was randomly assigned to deliver their statement first. To reduce interactivity as much as possible in the single exchange condition, both people prepared their statements at the same time, before they had heard or read the other person’s opinions. (However, it is still possible the second person deviated from their prepared remarks after listening to or reading their partner’s opinions.) In the multiple exchanges condition, participants simply discussed their topic either via writing or speaking for six minutes after considering the topic for about a minute.

Last, participants completed a private post-survey in which they reported their experience during the conversation, impressions of the partner, attitudes on all three topics again, and demographic information. They received payment for the study and were debriefed about the study hypotheses. More protocol details and the full text of each survey measure are in the Supplementary Methods.

### Study 2B participants

In total, we recruited 482 participants from the laboratory participant pool of the University of California, Berkeley. Of that set, 25 people (5.0%) could not participate in the study because either they could not be matched with a partner within their session or were not fluent enough in English. Of those who participated, 46 (9.5%) were removed from the analysis due to our exclusion criteria (the same as in Studies 1 and 2A). All those who began their conversation with their assigned partner also finished the conversation and completed the post-conversation survey; there was no post-treatment attrition. Our final sample was 410 individuals (205 dyads; 66.8% Female, 31.7% Male, *M* age = 20.27 years, SD age = 3.02 years) who participated in exchange for $15 each.

### Study 2B experiment design

Dyads were randomly assigned to one of four conditions at the lab session level in a 2 (conversation medium: speaking or writing) × 2 (conversation length: short or long) between-subjects experimental design. The short conversations were 6 min long (3 min shorter than in Study 1), and the longer conversations were 12 min long (twice as long as the shorter conversations, and 3 minutes longer than in Study 1). In this experiment, participants in all conditions communicated using the same platform used in Study 1. This experiment was not preregistered.

### Study 2B protocol

We followed a similar procedure to that in Study 1. First, participants consented to the study and completed a private pre-survey reporting their attitudes on three topics. To increase generalizability, we pre-tested and selected a different set of topics: participants’ opinions about the importance of freedom of speech, slavery reparations, and legalizing drugs (see Supplementary Methods for topic details and the pilot study conducted to select the topics). The experimenters downloaded participants’ pre-survey responses and used them to pair participants based on strong disagreement on a single topic while participants completed a private personality survey.

Next, pairs discussed their topic via the randomly assigned conversation medium for the randomly assigned amount of time.

Last, participants completed a private post-survey in which they reported their experiences during the conversation, impressions of their partner, attitudes on all three topics again, and demographic information. They received payment for the study and were debriefed about the study hypotheses. More protocol details and the full text of each survey measure is in the Supplementary Methods.

### Study 2C participants

In total, we recruited 489 participants from the laboratory participant pool of the University of California, Berkeley. Of that set, 17 people (3.5%) could not participate in the study because either they could not be matched with a partner within their session, were not fluent enough in English, or voluntarily left prior to starting the conversation. Of those who participated, 58 (12.3%) had to be removed from the analysis due to our predetermined exclusion criteria (the same as in Studies 1, 2A, and 2B). All those who began their conversation with their assigned partner finished the conversation and completed the post-conversation survey; there was no post-treatment attrition. Our final sample was 414 individuals (207 dyads; 68.6% Female, 30.2% Male, *M* age = 20.66 years, SD age = 2.68 years) who participated in exchange for $15 each.

### Study 2C experiment design

Dyads were randomly assigned to one of four conditions at the lab session level in a 2 (conversation medium: speaking or writing) × 2 (conversation synchronicity: asynchronous or synchronous) between-subjects experimental design. In this experiment, participants in all conditions communicated using the same platform used in Study 1. This experiment was preregistered at 10.17605/OSF.IO/C3ZGY (see summary of preregistration deviations in Supplementary Note 1).

### Study 2C protocol

We used a similar protocol as described in Study 2B, including the same three topics of conversation. First, participants consented to the study, completed a private pre-survey reporting their attitudes on the three topics, and were matched (while completing a private personality survey) to discuss a single topic with a conversation partner based on strong disagreement.

Next, participants had their conversation. In the synchronous condition, participants were allowed to speak or write to each other for 12 minutes. In the asynchronous condition, participants in both the speaking and writing conditions were given time to speak, write, listen, or read over three rounds. Specifically, in round one, Person A (randomly assigned) was given one minute to speak (i.e., creating an audio message) or write (i.e., creating a chat message), and then Person B had one minute to listen to the audio message or read the chat message. The positions were then swapped so Person B had one minute to speak or write, and then Person A read or listened. Round one took four minutes in total. Rounds two and three were repeats of round one. Thus, participants in all conditions had 12 minutes in total of conversation time.

Last, participants completed a private post-survey in which they reported their experiences during the conversation, impressions of their partner, attitudes on all three topics again, and demographic information. They received payment for the study and were debriefed about the study hypotheses. More protocol details and the full text of each survey measure is in the Supplementary Methods.

### Study 3: Beyond the laboratory

To extend our findings beyond our initial laboratory participant samples, we partnered with an organization called “Bridge USA,” a youth-led non-profit organization that “creates space for high school and college campuses for open discussion between students about political issues”^76^. We conducted in-person “political conversation events” on three different college campuses across the USA. During the events, we randomly assigned attendees to sit at one of two tables that were designated either for spoken or written conversation. Participants had conversations as they normally would at such events, but they were aware they were in a study, making this a “framed field experiment”^77^. To control for many of the possible differences between speaking and writing, we had pairs always sit across from one another with a laptop in front of them, thus occupying similar physical space. We designed this procedure to enhance our internal validity – for example, by ensuring participants would see each other regardless of whether they were typing on a laptop to their partner or speaking above the laptop with them – but note it may also have reduced external validity – for example, by potentially making the experience feel less natural.

There were three other substantive differences in the design of this study that allowed us to extend the results from Studies 1 and 2. First, participants were allowed to have multiple conversations, each with a different counterpart (though every person stayed within their assigned condition of speaking or writing the whole time). Second, each campus used only one topic for discussion, meaning we had a much higher number of dyads who agreed with each other. Instead of excluding agreeing pairs as we did in prior studies, we instead included them and conducted analyses on their survey data. Finally, in one site, we collected additional information about actual understanding whereby each person predicted their counterpart’s true stance on the topic after each conversation as a complement to our measures of perceived understanding in Studies 1 and 2.

### Participants

We conducted this study at three college campuses selected for their easy access (University of California, Berkeley) and political divisiveness (Arizona State University and Minnesota State University, Mankato). At each campus, we selected one controversial topic (as advised by the local Bridge USA chapter’s student leadership) and hosted an event billed as an “evening of political discourse.” Participants were primarily recruited on Eventbrite and various social media platforms; for example, at one site the Eventbrite invited students to: “Join us for an evening of political discourse by talking with people who might have different political opinions than you do.” Participants had to be 18 years of age or older and were required to pay $3 to enter without a valid student ID. Although we conducted the study at three locations, due to the small sample size and the common methodology employed, we combined the data from all three locations into a single dataset for analysis. We recruited 104 participants in total (38 from UC Berkeley, 32 from MNSU, and 34 from ASU). See Supplementary Note 15. We asked participants to fill out a consent form after arrival. Although the event lasted approximately one hour, allowing for multiple conversations, participants could come late or leave early; consequently, the number of conversations per participant varied (*M* = 5.37 conversation post-surveys completed per person, SD = 0.91; total *N* = 425 conversation post-surveys). The demographic composition across the three campus samples was: 41.2% Female, 50.5% Male, *M* age = 20.4 years, SD age = 1.92 years. Not all participants completed pre-surveys or post-surveys (for attrition details see Supplementary Note 15). In the analyses below, we only include the participants: a) who completed pre-surveys, because we used their pre-survey responses to determine whether they initially agreed or disagreed with their partner’s attitudes on the topic of conversation, and b) whose partner completed a post-survey, so that we could analyze both their and their partner’s responses. In total, these analyses contain 179 conversations (358 post-surveys), 52 conversations wherein the pairs initially disagreed and 127 wherein the pairs initially agreed. For robustness analyses with different data samples, see Supplementary Note 4.

### Experiment design

Participants were randomly assigned to one of two conditions, speaking or writing. Writing participants communicated via the same platform used in Study 1, whereas speaking participants talked in person. We preregistered the experiments at two campus locations 10.17605/OSF.IO/C3ZGY. See preregistration deviations in Supplementary Note 1.

### Protocol

Upon entering the event, participants were randomly assigned to either the speaking or writing condition, and directed to the relevant table designated for their experimental condition. Once seated, participants completed a pre-study survey in which they consented to participate and reported their stance on the topic of discussion, the strength of that stance, and demographic information (e.g., age, gender). The topic of conversation varied by the school; participants reported and discussed their opinions about same-sex marriage at UC Berkeley, gun control laws at MNSU, and the US-Mexico border wall at ASU.

Next, participants had a conversation. Their conversation partner was the person who happened to be seated across from them; they were not matched to someone with whom they disagreed as in prior studies. Each participant had a laptop on the table in front of them (on which they had completed the pre-survey). In the speaking condition, participants simply spoke out loud to one another, face-to-face, above the laptop, whereas in the writing condition participants typed to one another on their laptop (but could still see each other above the laptops). Participants were given up to eight minutes for their spoken and written conversations at UC Berkeley and MNSU. At ASU, we varied the timing to provide a more conservative test of our hypothesis, such that written conversation partners had 12 min, whereas spoken conversation partners had six minutes to communicate (using the conversation lengths tested in Study 2B). See Supplementary Methods for all instructions given to participants.

After each conversation, participants completed a post-conversation survey in which they rated their experience in the conversation and with their partner (perceived understanding, conflict, influence on partner’s opinions, enjoyment of conversation, and agreement with partner), impressions of their partner (liking, perceived competence), and their attitudes again on the same scale as in the pre-study survey.

To move participants to their next conversation, we asked the participants seated on one side of the table to move one seat to their right so they faced a new conversation partner. They had their next conversation with that partner and completed their post-survey after their conversation, following the procedure described above. Typically, the event lasted about one hour, and most participants had four or five conversations within that time period (see Supplementary Note 4).

### Measures

Perceived understanding was measured using the same items as in Studies 1-2 (“To what extent did you think your conversation partner understood your opinions”, “To what extent did you think you understood your conversation partner’s opinions”; *ɑ* = 0.78) on a 7-point Likert response scale from 0 (Did not understand at all) to 6 (Understood extremely well). To shorten the survey, perceived conflict was measured using only a single, face-valid item (“How much conflict did you feel during your conversation”) on a 7-point Likert response scale from 0 (No conflict at all) to 6 (A great deal of conflict).

We also collected other measures, including liking of one’s partner, perceived agreement with partner, enjoyment of the conversation, the perceived competence of one’s partner, and the participant’s own attitudes. Some of the other measurement scales varied across campus samples and were not used in the pooled regression analysis. See Supplementary Methods. In particular, for one of the campus sites (ASU), we asked participants to predict their partner’s stance on the topic after their conversation as well as report their own stance on the topic, enabling us to measure the effect of medium on actual understanding: the absolute difference between the prediction of a partner’s stance and the partner’s actual stance on the topic, in the units of the original 7-point scale. Additionally, because the spoken conversations were face-to-face, we do not have complete transcript data for the conversations (recordings were attempted at one location, but the background noise was too disruptive).

### Study 4: The effect of the consumed and produced medium on constructive disagreement

Studies 1–3 showed that people perceive greater understanding and less conflict when speaking than when writing to one another. We propose two broad explanations for these results. First, it could be that the medium affects how a message is consumed—that is, how it is heard or read by the audience. In particular, the same linguistic content (i.e., words) could elicit more constructive reactions when heard than read, due to the paralinguistic cues in a person’s voice (e.g., vocal tone, pacing) revealing subtle inflections of meaning, thoughtfulness, and emotionality. Second, it could also be that the medium affects how a message is produced—that is, which words are selected when speaking or writing^33^. Indeed, speakers tend to produce different types of linguistic content when they talk than when they write, and it could be that these linguistic choices drive more constructive disagreement^32^. We test both possibilities in Study 4.

Specifically, we recruited online workers to read or listen to statements made by communicators in Study 2A. Critically, some of the spoken statements were heard in their original form (via the communicator’s own voice), whereas some were instead read (via a transcription), and some of the written messages were read in their original form (via a text statement), whereas others were heard (via actors’ voices). Subsequently, participants reported their impression of the original communicators from Study 2A and evaluated the conversations as a whole.

### Participants

In total, we aimed to recruit 1000 participants from Amazon Mechanical Turk. 1,072 participants started the survey. Of that set, 9 people did not consent; 63 additional people could not complete the study, and a further 29 failed the attention checks. Although these exclusions were not in the pre-registration, the results are identical regardless of how they are handled, and we exclude these observations for consistency with the other studies. This leaves a final sample of 971 participants (54.0% Female, 45.9% Male; M age = 38.1 years, SD age = 12.2 years). As the stimuli in this study, we use conversations in the single exchange conditions from Study 2A (*N* = 89 original conversations, 42 spoken and 47 written). Each conversation was rated 10.9 times by different participants on average, where each new participant rated both communicators engaged in a single conversation. Attrition did significantly varied across mediums in the production condition (chi-squared (1) = 7.66, *p* = 0.006) but was not statistically significant in the consumption condition (chi-squared (1) = 0.25, *p* = 0.617).

### Experiment design

Participants were randomly assigned to one of four conditions in a 2 (communicators’ medium of production: speaking or writing) × 2 (observers’ medium of consumption: listening or reading) between-subjects experimental design. This experiment was preregistered at 10.17605/OSF.IO/C3ZGY.

### Observed conversations

The conversations that participants observed were drawn from the spoken or written single-exchange conversations in Study 2A, but some were modified for the experimental condition in the following ways.

For the “produced-by-speaking but consumed-by-reading” experimental condition, we transcribed the originally spoken conversations using the transcription guidelines shown in Supplementary Methods. Thus, participants read (i.e., in the consumption medium) conversations that had originally been spoken (i.e., in the production medium). For the “produced-by-writing but consumed-by-listening” experimental condition, we asked research assistants (serving as our voice actors) to read aloud the originally written conversations, using the guidelines shown in Supplementary Methods. Specifically, we asked two women and two men actors to read aloud the gender-matched participant’s writing in pairs, as if they were having a real conversation. Thus, participants listened to (i.e., in the consumption medium) conversations that had originally been written (i.e., in the production medium). For the “produced-by-speaking and consumed-by-listening” experimental condition, we used audio clips of the original spoken conversations. Thus, participants heard conversations that had originally been spoken. Finally, for the “produced-by-writing and consumed-by-reading” experimental condition, we used the original written conversations. Thus, participants read conversations that had originally been written.

### Protocol

After providing consent, participants (i.e., our observers in this study) were asked to state their own position on the three topics that were discussed in Study 2A (i.e., GMOs, drinking age on campus, and race admissions quotas). Participants next read: “For this study, you will [read] / [listen to] a conversation between two [name redacted] undergraduate students who took part in an experiment, Person A and Person B. For the experiment, Person A and Person B were required to discuss a specific topic that they disagreed about.” In the produced-by-speaking but consumed-by-reading condition, participants learned: “[Person A and Person B] were told to share their opinions by each delivering a face-to-face, spoken statement to each other while in a room together. We then transcribed both Person A and Person B’s spoken statements so that you could read them.” In the produced-by-writing but consumed-by-listening condition, participants learned: “[Person A and Person B] were told to share their opinions by each writing a statement and then emailing their statement to the other. We then asked two actors to read Person A and Person B’s written statements aloud and recorded the actors’ readings so that you could listen to them.” In the produced-by-writing and consumed-by-reading condition, participants learned: “[Person A and Person B] were told to share their opinions by each writing a statement and then emailing their statement to the other. The statements you will read are their original written statements.” Finally, in the produced-by-speaking and consumed-by-listening condition, participants learned: “[Person A and Person B] were told to share their opinions by each delivering a face-to-face, spoken statement to each other while in a room together. The statements you will listen to are their original spoken statements.”

In all conditions, we asked participants to “please pay close attention to what Person A and Person B said [wrote].” We also reported the prompt that the original communicators answered and their positions (e.g., “Person A and Person B answered this prompt: Do you oppose or support changing the legal drinking age from 21 to 18? Person A was in favor lowering the legal drinking age and Person B was opposed. Person A delivered the first statement.”). In the listening conditions, we further instructed participants: “Listen to the conversation below carefully. You can start by hitting the ‘play’ button and can pause or rewind as needed.”

### Measures

After reading or listening to a conversation, participants gave one rating to both communicators on perceived understanding and conflict (as well as other aspects of the conversation experience, e.g., perceived responsiveness, enjoyment, agreement and common ground). They also rated their impressions of each person separately using the same items measuring perceived competence, humanisation, and liking from Studies 1 and 2 (see measures in Supplementary Methods). All scales were adapted from Studies 1 and 2 for the perspective of an observer of the conversation. Finally, participants reported their demographic information and provided comments on technical difficulties and other feedback about the study.

### Study 5: Disagreement conversation language analyses

Although Study 4 identified separate mechanisms of the conversation medium on both the production and consumption of conversation, this begs the question: how exactly does medium change production? As data, dialogue is unstructured and high-dimensional, and there could be many different ways in which the language uttered when speaking is different from when writing^78^. One common approach to analyzing text data is to ask human annotators to evaluate the text on several dimensions. Indeed, we conducted such a study (see Supplementary Note 7), asking annotators to rate aspects of communicators’ transcribed spoken or written conversations at different time points during the conversation. The results showed that, at most time points, annotators perceived the spoken conversation partners to have greater understanding, liking, and agreement, and less conflict, than the writing partners – but that this effect varied over time (for full results, see Supplementary Note 7). However, an annotation approach has limitations. Human annotators are not scalable—their methods are not easily applied across datasets. Furthermore, although annotations reveal the outcomes of human judgment, they do not directly connect those outcomes to the elements in the data (i.e. the conversation behavior) that lead to those judgments.

Here, we apply tools from natural language processing to investigate differences in linguistic content across the two media. In particular, we focus on features associated in previous research with conversational receptiveness (i.e., conversation behavior that signals openness to opposing viewpoints, building trust during disagreement)^52^.

### Sample

To increase our statistical power, we combined all of the data from Studies 1 and 2 into a single dataset of 743 dyads (1486 participants). The spoken conversations were transcribed (see Supplementary Methods for full transcription guide). However, due to technical issues with conversation recordings, 44 conversation transcripts had to be excluded in the language analysis (Study 1: 13 transcripts excluded (91.1% retained); Study 2A: 4 transcripts excluded (97.8% retained); Study 2B: 7 transcripts excluded (96.6% retained); Study 2C: 20 transcripts excluded (90.3% retained)). This leaves 699 conversation transcripts from 1398 participants available for language analysis. The conversations from Study 3 were not recorded, so could not be added to this dataset. Although we did not spell-check the written conversations in this analysis, we conducted a follow-up analysis in which we spell-checked the Study 1 written conversations and confirmed they produced the same effects as the non-checked versions (see Supplementary Note 8).

### Feature extraction

From the transcripts, we extracted text features primarily using the politeness R package (version 0.9.4), which uses spaCy version 3.8.7^79^. The package identifies, for any written language, 40 theory-based features that isolate structural and stylistic aspects of linguistic content. For example, the package extracts linguistic features associated with receptiveness, such as Agreement and Acknowledgement, including ones with negative associations, such as Negation. We extract all features from each turn in conversation. In addition, we use the pre-trained receptiveness model from the package to score each participant’s overall receptiveness separately, treating each person’s entire portion of the transcript as a single document^52^.

The receptiveness algorithm focuses on ten discrete features, with independent linear coefficients in the model, allowing us to isolate the unique impact of each of these features. Previous research has found a significant and interpretable relationship between each of the ten features and perceptions of receptiveness. There are five features associated with higher receptiveness scores: acknowledgement (acknowledging others’ views, e.g., “I get that” or “I understand where you are coming from”); agreement (emphasizing agreement or areas of common ground, e.g., “I agree with you on this point”); positive emotions (framing arguments in a positive way, e.g., “This is great”); subjectivity (making clear that a statement is based on a personal viewpoint than an objective fact, e.g., “In my opinion” or “I believe”); and hedges (softening one’s stance via modifiers, thereby reducing the degree of certainty in a statement, e.g., “sometimes” or “maybe”). Conversely, there are five features associated with lower receptiveness scores: negative emotion (framing arguments in a negative way, e.g., “This is terrible”), disagreement (emphasizing areas of difference or disagreement, e.g., “I don’t agree with this”), adverb limiters (adverbs that increase ambiguity, e.g., “merely”, “simply”); negation (phrases that explicitly negate a statement, e.g., “not”, “never”, “no”); and reasoning (phrases used to explain a rationale which can be conflated with defensiveness if overused, e.g., “therefore”, “because”). Differences in conversational receptiveness should reflect the distribution of these ten features across speaking and writing.

### Mediation analysis

To test the possibility that conversational receptiveness might mediate the relationships between medium and perceived understanding, perceived conflict, and other conversation outcomes, we conducted mediation models using the mediation R package, controlling for word count, study number, and topic^80^. All mediations were estimated using nonparametric bootstrapping with 20,000 simulations, which provides robust confidence intervals without relying on normality assumptions.

### Moderation analysis

To test the possibility of whether any associations between conversational receptiveness and constructive disagreement outcomes are moderated by medium, we included an interaction term (medium × receptiveness) in the regression models as described above.

### Study 6: The expected effects of conversation medium

Although we randomly assigned communicators to a conversation medium in Studies 1-3, typically, people can choose which medium they use for a conversation. Here we explore those choices directly, in a vignette study which measured people’s preferences for different media. We wondered whether people have correct lay theories about how conversation medium can affect their disagreement outcomes. In particular, we tested whether people erroneously think that writing could make disagreement more constructive than speaking or video-chatting, as well as whether they tend to prefer writing when they communicate with a disagreeing counterpart.

### Participants

We planned to recruit 200 participants from MTurk. In total, 202 individuals (49.5% male, 48.0% female; *M* age = 40.86, SD = 12.97 years; 23.8% conservative, 55.0% liberal, 21.3% moderate; 49.0% college degree or higher; 72.3% white, 27.7% non-white or mixed race) agreed to participate in exchange for $0.60.

### Experiment design

The experiment had three within-participants conditions: video-chatting, speaking, and writing. The experiment was preregistered at: 10.17605/OSF.IO/C3ZGY.

### Protocol

Participants were told to imagine the following scenario, which we designed to match the actual experimental instructions from Studies 1-2 (see full text in Supplementary Methods): “You sign up to take part in a research study… First, the researchers ask you to complete an online survey about your opinions on three controversial political issues… For each topic, you rate how strongly you support or oppose a particular stance … and how strongly you feel about your opinion… Based on your answers to the survey, the researchers match you with another participant who strongly disagrees with you on one of the political issues…. Next, you will debate this person on the issues on which you disagree.”

Participants then learned the study has three different conditions: the video-chatting condition (“You and the other person will debate the topic over a Skype video call”), the speaking condition (“You and the other person will debate the topic over a Skype phone call (no video)”), and the writing condition (“You and the other person will debate the topic by typing back and forth over Skype’s chat feature”). We used Skype to keep the platform the same across all three conditions, and because it was the actual platform used in Studies 1-2. Finally, participants completed a survey about their preferences and predictions regarding the imagined study.

### Measures

After participants imagined being in each of the three conditions (e.g., “Imagine that you are assigned to have a video call with the other person with whom you strongly disagree”) in randomized order, they then predicted their conversation experience. In particular, participants predicted understanding (two items, *ɑ* = 0.88) and conflict (four items, ɑ = 0.92). Participants also predicted their beliefs about the competence (five items, *ɑ* = 0.95) of the other person and how much they would like the other person after the conversation was over, as well as their own responsiveness, enjoyment, discomfort (two items, *ɑ* = 0.85), awkwardness, effort (three items, ɑ = 0.77), and agreement with the other person (two items, *ɑ* = 0.92). Next, participants reported their preferred medium: “If you could choose, which of the three conditions would you want to be assigned to?” (Video-Chatting Condition: Skype Video Call / Talking Condition: Skype Phone Call (No Video) / Typing Condition: Skype Chat Feature) and why “you want to be in the condition that you selected.” Even though we labeled the experimental conditions video-chatting, speaking, and writing, we thought it would be more intuitive to call the latter two conditions “talking” and “typing” for participants in the survey question. The full text of all dependent measures is reported in Supplementary Methods.

After making predictions about the conversation in all three conversation media conditions (in randomized order) and reporting their preferred medium, participants reported their demographic information (gender, age, race, political orientation, and education).

### Reporting summary

Further information on research design is available in the Nature Portfolio Reporting Summary linked to this article.

## Supplementary information

Supplementary Information

Reporting Summary

Peer Review file

## Data Availability

All data, stimuli, analysis code, and preregistrations are posted on the Open Science Framework website at 10.17605/OSF.IO/C3ZGY. The data shared in this study consist of anonymized datasets.

## Code availability

The analysis scripts for our studies are openly available via 10.17605/OSF.IO/C3ZGY.
