# Supplementary material for: Spoken disagreement is more constructive than written disagreement
Source: Nat Commun. 2026 Apr 27;17:5792. doi: 10.1038/s41467-026-71669-5 (PMC13332060; doi:10.1038/s41467-026-71669-5)
Supplement: Supplementary file 1 — Supplementary Information [file 41467_2026_71669_MOESM1_ESM.pdf]

SPOKEN DISAGREEMENT IS MORE CONSTRUCTIVE THAN WRITTEN DISAGREEMENT

SUPPLEMENTARY INFORMATION

<u>**Table of Contents**</u>

Supplementary Note 1 - Preregistration Links and Summary of Deviations

Supplementary Note 2 - Analysis of Secondary Outcome Measures (Studies 1-3)

Supplementary Note 3 - Analysis of Conversation Structure and Statistical Power in Study 2

Supplementary Note 4 - Additional Analyses from Study 3

Supplementary Note 5 - The Role of Individual Differences (Studies 1-3)

Supplementary Note 6 - Interaction Effects of Consumed and Produced Media (Study 4)

Supplementary Note 7 - Annotation Study: Dividing Conversations into Exchanges

Supplementary Note 8 - Analysis of Spell-Checked Language (Study 1)

Supplementary Note 9 - Effect of Medium on Receptive Language (Studies 1-2)

Supplementary Note 10 - Testing Receptive Language as a Mediator (Studies 1-2)

Supplementary Note 11 - Testing Receptive Language as a Moderator (Studies 1-2)

Supplementary Note 12 - The Effect of Medium on Other Changes in Language (Studies 1-2)

Supplementary Note 13 - Additional Analyses from Study 6

Supplementary Note 14 - Analyses of Acoustic Features of Speech (Studies 1-2)

Supplementary Note 15 - Attrition Across Studies 1-3

Supplementary Methods

- Topic Descriptions Used in Studies 1-3
- Protocol Details for Studies 1-3
- Dependent Measures Across Studies

- Transcription and Reading Guides Used in Study 4
- Materials for Study 6

Supplementary Note 1 - Preregistration Links and Summary of Deviations

Links to each study's public preregistration are provided in Supplementary Table S1 below. Study 3 employed separate preregistrations for each participant sample, except for the sample of participants from the University of California, Berkeley, which was not preregistered. Although our analyses mostly followed these preregistrations, there were certain consistent deviations across the set of studies. We believe these deviations are justifiable, as they improve the precision of the analyses and have no meaningful impact on the results or conclusions of any of our studies. In the interest of transparency, we describe them here. In general, we prioritized consistent analysis across all studies, which makes our analyses easier to read and understand and also constrains our researcher degrees of freedom.

Most notably, the preregistrations only specified an intended sample size, but did not describe any exclusion criteria. Subsequently, we applied a consistent set of exclusion criteria across all studies, which are fully described in Supplementary Note 15. Primarily, we excluded people who did not finish the study (sometimes due to technical difficulties). We also excluded dyads in Studies 1 and 2 who did not adequately strongly disagree with one another - the original intent was to have every pair strongly disagree, but this was sometimes impossible due to participants' self-reported attitudes and when they enrolled in the study (e.g., if a participant did not report disagreeing with anyone else enrolled in their study session).

Several of our analyses in the main text involved pooled samples. Where possible, we report analyses that combine all three participant samples for Study 3, due to low statistical power in each sample individually. Likewise, we also report pooled results across Studies 1-3, to more precisely estimate treatment effects across all outcome measures and to identify any individual differences in effects. In the summary section that uses natural language

processing, we also pool all conversations from Studies 1 and 2 that were recorded and/or transcribed, to increase the precision of our estimated effects.

We did not use ANOVAs to test any treatment effects, opting instead to conduct regression analyses which are easier to interpret and apply consistently across studies. We also declined to report preregistered mediation analyses in which post-conversation scale measures were proposed as a mediator. Although this analytic approach is common, it can be hard to interpret causality as it violates sequential ignorability (Imai et al., 2010), a key assumption of causal inference. Instead, we conduct exploratory analyses in Section 7 of the paper that test whether conversation behavior itself (e.g., use of receptive language) is a mediator of the effects, in essence, extracting the potential mediating variables directly from the conversation behavior.

Finally, the preregistrations sometimes grouped our outcome measures into indexes in different ways. Broadly speaking, we see treatment effects on almost all of our outcomes in almost all studies, so the grouping does not affect the statistical significance of our conclusions. Instead, we chose to group our outcome measures consistently across all studies, for reader clarity and based on tests of internal reliability, which are all in Supplementary Information R.

Supplementary Table S1: Links to Preregistrations

**Study**	**Preregistration Link**	**Preregistration date**
1	not preregistered	
2A	https://doi.org/10.17605/OSF.IO/C3ZGY	18/08/2017
2B	not preregistered	
2C	https://doi.org/10.17605/OSF.IO/C3ZGY	12/04/2019
3 - UC Berkeley sample	not preregistered	
3 - MNSU sample	https://doi.org/10.17605/OSF.IO/C3ZGY	26/02/2026

3 - ASU sample	https://doi.org/10.17605/OSF.IO/C3ZGY	25/03/2019
4	https://doi.org/10.17605/OSF.IO/C3ZGY	28/09/2018
6	https://doi.org/10.17605/OSF.IO/C3ZGY	12/12/2023

Supplementary Note 2 - Analysis of Secondary Outcome Measures (Studies 1-3)

Supplementary Table S2. Linear regression models estimating the effects of conversation medium on secondary dependent variables using data pooled from Studies 1, 2, and 3. All models are estimated using ordinary least squares with standard errors clustered at the dyad, individual, and session levels, and include topic and study fixed effects. Statistical tests are two-sided. No adjustments were made for multiple comparisons. Results indicate that conversation medium has a statistically significant effect across all dependent variables.

	Perceived Understanding	Perceived Conflict	Perceived Humanisation	Perceived Competence	Attitude Shift Towards Partner	Liking	Enjoyment
	(1)	(2)	(3)	(4)	(5)	(6)	(7)
Medium: Speaking	0.446***	-0.227***	0.195***	0.325***	0.15*	0.266***	0.374***
	(0.055)	(0.062)	(0.050)	(0.057)	(0.064)	(0.038)	(0.059)
Topic F/E	YES	YES	YES	YES	YES	YES	YES
Experiment F/E	YES	YES	YES	YES	YES	YES	YES
Observations	1590	1590	1486	1590	1590	1590	1486
Degrees of Freedom	1578	1578	1477	1578	1578	1578	1477
R^2	0.065	0.022	0.012	0.038	0.405	0.604	0.133
Adjusted R^2	0.056	0.012	0.006	0.029	0.401	0.605	0.128

Note: Standard errors shown in parentheses
*p<0.05; **p<0.01; ***p<0.001

Note: Measures on perceived humanisation and enjoyment were not collected in Study 3. Attitude shift toward partner was measured as the change in a participant's stance on their topic of discussion at the end of their conversation towards their partner's stance.

Supplementary Note 3 - Analysis of Conversation Structure and Statistical Power in Study 2

Supplementary Table S3. Regression showing the effects of conversation medium on perceived understanding and perceived conflict in Studies 2A, 2B and 2C. All models are estimated using ordinary least squares with standard errors clustered at the dyad and session levels, controlling for topic fixed effects. Statistical tests are two-sided. No adjustments were made for multiple comparisons.All regressions are clustered at the dyad and session levels, controlling for topic fixed-effects. Results show that the structural differences in conversations we tested (i.e., interactivity, duration, and synchronicity) have no statistically significant effects on perceived understanding or conflict, nor do they interact with the effect of medium on perceived understanding or conflict.

	Perceived Understanding (2A)	Perceived Understanding (2B)	Perceived Understanding (2C)	Perceived Conflict (2A)	Perceived Conflict (2B)	Perceived Conflict (2C)
**Medium**: Speaking	0.539***	0.359*	0.461**	0.164	-0.190	-0.557***
	(0.151)	(0.154)	(0.155)	(0.157)	(0.153)	(0.157)
**Interactivity**: Single exchange	-0.056			-0.070		
	(0.164)			(0.158)		
Speaking*Single exchange	-0.034			-0.285		
	(0.224)			(0.220)		
**Duration**: Long		-0.020			0.260	
		(0.167)			(0.162)	
Speaking*Long		-0.144			-0.171	
		(0.219)			(0.226)	
**Synchronicity**: Asynchronous			-0.093			-0.150
			(0.165)			(0.170)
Speaking*Asynchronous			-0.244			0.226
			(0.218)			(0.208)
Topic F/E	YES	YES	YES	YES	YES	YES
Observations	370	410	414	370	410	414
Degrees of Freedom	364	404	408	364	404	408
R^2	0.071	0.038	0.059	0.021	0.013	0.059
Adjusted R^2	0.058	0.026	0.047	0.007	0.000	0.048

Note: Standard errors shown in parentheses
*p<0.05; **p<0.01; ***p<0.001

Supplementary Figure S1. Effect of conversation medium and structure (single vs multiple exchanges) on perceived conflict and understanding in Study 2A. All data points represent group means. Group means are calculated as simple arithmetic means, rather than estimated coefficients derived from regression models. Error bars show the standard error of the means.

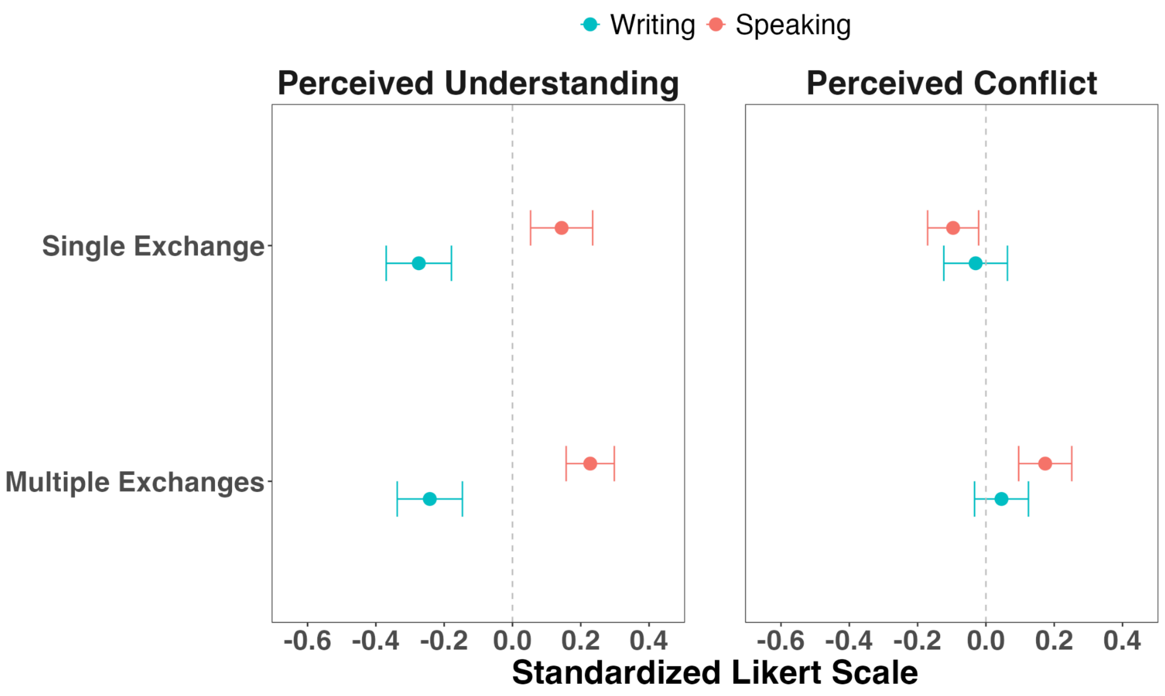

Supplementary Figure S2. Effect of conversation medium and structure (long vs short) on perceived conflict and understanding in Study 2B. All data points represent group means. Group means are calculated as simple arithmetic means, rather than estimated coefficients derived from regression models. Error bars show the standard error of the means.

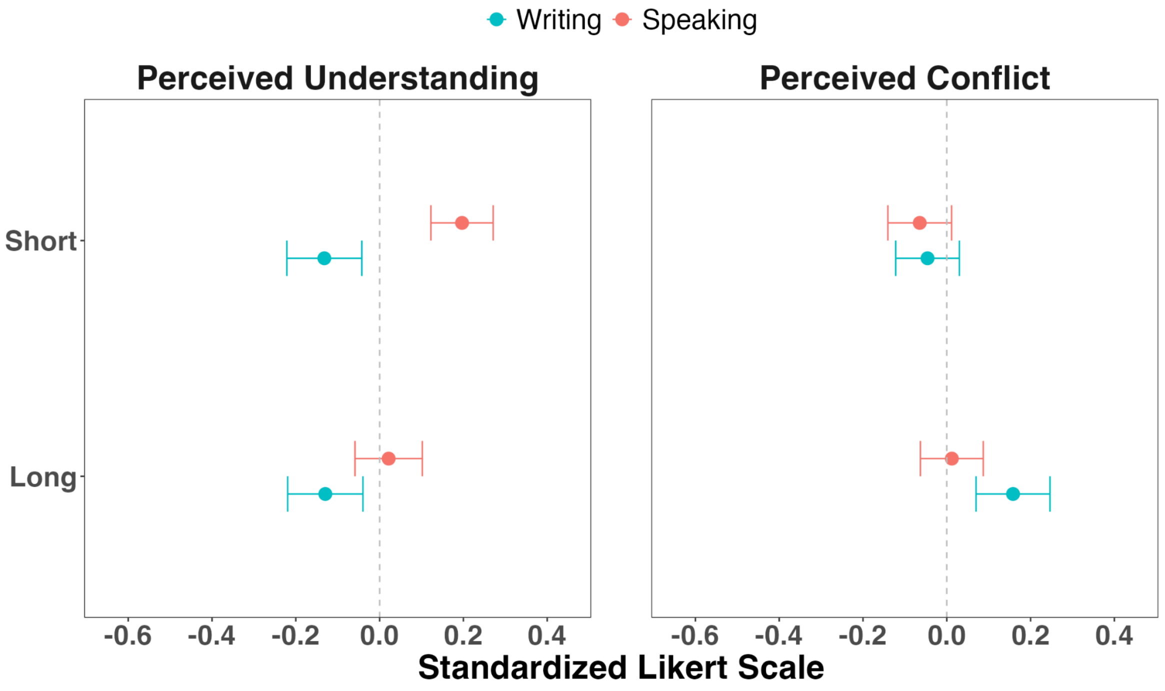

Supplementary Figure S3. Effect of conversation medium and structure (synchronous vs asynchronous) on perceived conflict and understanding in Study 2C. All data points represent group means. Group means are calculated as simple arithmetic means, rather than estimated coefficients derived from regression models. Error bars show the standard error of the means.

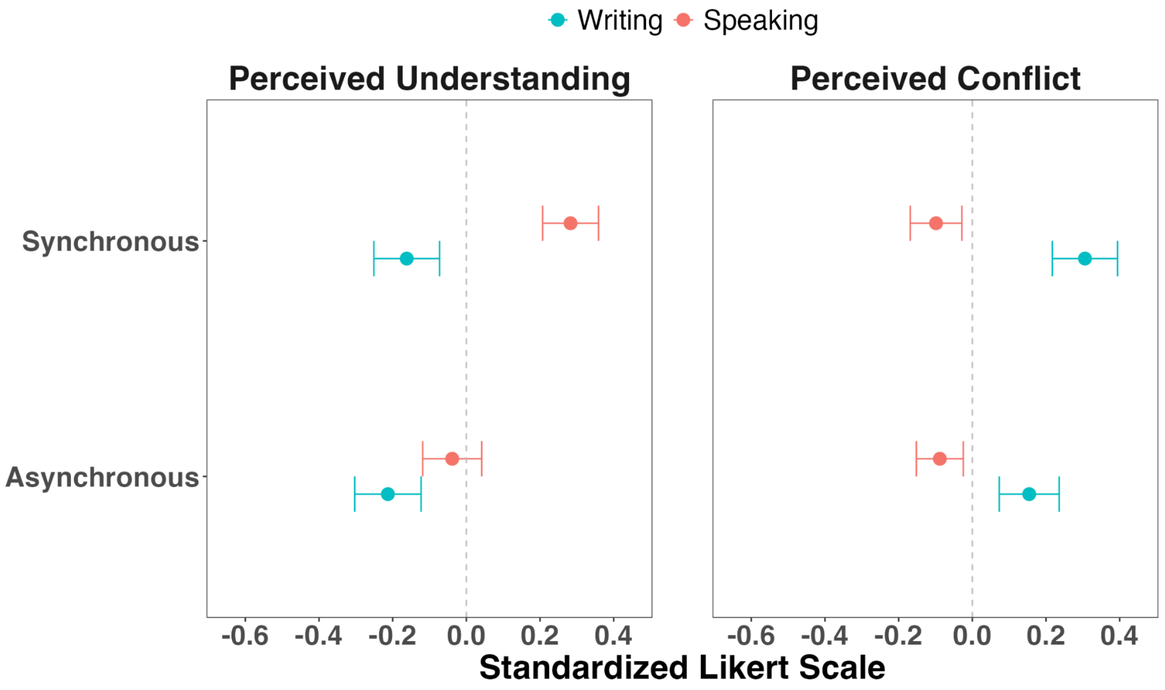

Additional analysis of statistical power. To help interpret the statistically non-significant interaction effects between conversation medium and structure in Studies 2A-C (shown in Figures S2-4 above), we conducted simulation-based power analyses. In each simulation, we held the sample size and predictors constant, and generated new outcome values (e.g., for perceived understanding) by varying the coefficient (β) of the interaction term from −2.0 to +2.0 in increments of 0.05. For each value of β, we ran 1,000 simulations and estimated power as the proportion of simulations in which the interaction term reached statistical significance ($p < .05$), based on repeated sampling of synthetic outcomes.

All three studies were underpowered to detect statistically significant interaction effects. The minimum detectable interaction effects were higher (βs for 2A: 1.80, 2B: 1.10, 2C: 1.25) than our observed interaction effects (βs for 2A: .03, 2B: .14, 2C: .24). We note that it was not always possible for the minimum detectable interaction effects to be simulated with 95% power: Within the range of interaction effect sizes examined in our simulations ($\beta = -2$ to +2), and holding sample size and model structure fixed, the highest achievable power was 96% in Study 2A, 73% in Study 2B, and 89% in Study 2C. In Studies 2B and 2C, power did not reach 95% within this range due to the available sample sizes. Consistent with these results, the rate at which we can reject the null hypotheses for the interaction terms in our studies was low (2A: 5.7%, 2B: 10.9%, 2C: 23.3%), especially compared to the rate at which we can reject the null hypotheses for the main effects of medium (e.g., in Study 2A our analysis suggests we had 99% power to detect the effect of medium). This discrepancy reflects the greater difficulty in detecting interaction terms due to smaller effect sizes and larger standard errors. The code (power_simulations.R) for these analyses are posted on OSF (https://osf.io/k73nq).

Supplementary Note 4 - Additional Analyses from Study 3

Number of conversations. Across all sites, including all 104 participants who had a total of 228 conversations (222 for which we have at least one post-survey), 4 participants had one conversation, 6 participants had two conversations, 7 participants had three conversations, 27 participants had four conversations, 53 participants had five conversations, 3 participants had six conversations, and 4 participants had seven conversations. (Note that having six or seven conversations would only have been possible at the ASU site in the "spoken" condition because the conversations were relatively shorter in this condition.)

Summary of attrition. As can be seen in Supplementary Note 15, the most participant attrition occurred at the UC Berkeley site (i.e., 24 missing post-surveys out of 160 possible, and 7 missing pre-surveys out of 38 participants); in contrast, there was little attrition at the MNSU site (i.e., 7 missing post-surveys out of 154 possible, and no missing pre-surveys out of 32 participants) and almost no attrition at the ASU site (i.e., no missing post-surveys out of 142, only 1 missing pre-survey out of 34 participants). Further examining the pattern of attrition at the UC Berkeley site, we identify two key areas of differential attrition: fewer speakers than writers completed pre-surveys (71% vs. 95%, respectively) and fewer speakers than writers completed post-surveys (76% vs. 94%, respectively). Although we can't know for certain why this happened, a possible reason is that some speaking participants may not have realized they were supposed to be using the laptops in front of them to complete surveys (because speaking in itself does not require the use of a laptop, as compared to writing). Given the differential attrition in the Berkeley sample, we conducted several robustness analyses of the results reported in the main text.

Robustness analyses for the effect of medium on perceived understanding and conflict. First, we re-ran the analyses excluding all of the Berkeley data, separately for the initially disagreeing and agreeing pairs. Without Berkeley data, for disagreeing pairs (n = 84

post-surveys), participants who spoke (vs. wrote) reported more understanding (standardized β = .94, SE = .17, t(81) = 5.49, p < .001, 95 % CI [.60, 1.27]) and non-significantly less conflict (standardized β = -.41, SE = .20, t(81) = -2.01, p = .047, 95% CI [-.81, .01]). These results are similar to those that include the Berkeley data reported in the paper (n = 104 post-surveys; standardized understanding: β = 1.00, SE = .16, t(100) = 6.38, p < .001, 95% CI = [.69, 1.30]; conflict: standardized β = -.47, SE = .19, t(100) = -2.47, p = .015, 95% CI [-.83, -.10]). Without Berkeley data for the agreeing pairs (n = 192 post-surveys), participants who spoke (vs. wrote) again reported more understanding (standardized β = .51, SE = .13, t(189) = 3.80, p < .001, 95 % CI [.24, .77]) and non-significantly less conflict (standardized β = -.21, SE = .14, t(189) = -1.48, p = .141, 95% CI [-.48, .07]). These reports are similar to those that include the Berkeley data reported in the paper with n = 254 post-surveys: understanding (standardizedβ = .47, SE = .12, t(251) = 3.90, p < .001, 95% CI [.24, .71]) and conflict (standardized β = -.26, SE = .12, t(251) = -2.23, p = .027, 95% CI [-.49, -.03]).

Second, we re-ran the analysis to include all of the data we have across all three sites (even for participants who completed a post-survey but not a pre-survey or whose partner did not complete a post-survey; n = 425 post-surveys total). Note that this analysis combines the disagreeing pairs, agreeing pairs, and those pairs for whom initial disagreement or agreement could not be determined because one or both of them did not complete a pre-survey. With all the available post-survey data, we found that perceived understanding was still higher (standardized β = .64, SE = .09, t(422) = 6.85, p < .001, 95% CI [.46, .83]), and perceived conflict lower (standardized β = -.43, SE = .10, t(422) = -4.18, p < .001, 95% CI [-.63, -.23]) in the speaking than writing conditions.

Additional analyses

Effects over time. Since participants had multiple conversations in Study 3, we additionally explored whether the treatment effect varied over time. We did this with an

interaction term between conversation round and medium, controlling for campus site and clustering standard errors at the individual and dyad levels. This analysis includes all disagreeing and agreeing pairs who completed a pre-survey and post-survey during the first four rounds of conversation (final $n = 293$ post-surveys). We restricted the analysis to four rounds because at the ASU site, writers had longer rounds than speakers and only completed four rounds of conversation. We did not find statistically significant associations between conversation round and perceived understanding (standardized $\beta = .02$, $SE = .08$, $t(287) = 0.78$, $p = .777$, 95% CI [-.13, .17]), or an interaction between round and medium on understanding (standardized $\beta = .05$, $SE = .10$, $t(287) = 0.50$, $p = .620$, 95% CI [-.14, .24]). The association between conversation round and perceived conflict was also not statistically significant (standardized $\beta = .01$, $SE = .06$, $t(287) = 0.15$, $p = .883$, 95% CI [-.12, .013]) nor was the interaction between round and medium on conflict (standardized $\beta = .18$, $SE = .10$, $t(287) = 1.75$ $p = .082$, 95% CI [-.04, .38]). See Supplemental Figure S4.

We conducted an additional analysis focusing solely on the first round of conversations. This approach controls for factors such as participants practising their arguments, being influenced by prior discussions, or experiencing fatigue. Analyzing only the first round provides a more comparable analysis to those conducted in Studies 1 and 2, but it substantially restricts our sample size ($n = 84$ post-surveys, including pairs who agreed, disagreed, and whose initial attitudes could not be determined). For only the first round, both main effects were statistically significant (perceived understanding: standardized $\beta = .95$, $SE = .20$, $t(80) = 4.70$, $p < .001$, 95% CI [0.56, 1.35]; perceived conflict: standardized $\beta = -.72$, $SE = .18$, $t(80) = -3.97$, $p < .001$, 95% CI [-1.07, -0.36]).

Supplementary Figure S4. Effect of conversation medium on perceived conflict and understanding in Study 3 by conversation round for the first four rounds of conversation. All

data points represent group means. Group means are calculated as simple arithmetic means, rather than estimated coefficients derived from regression models. Error bars show the standard error of the means.

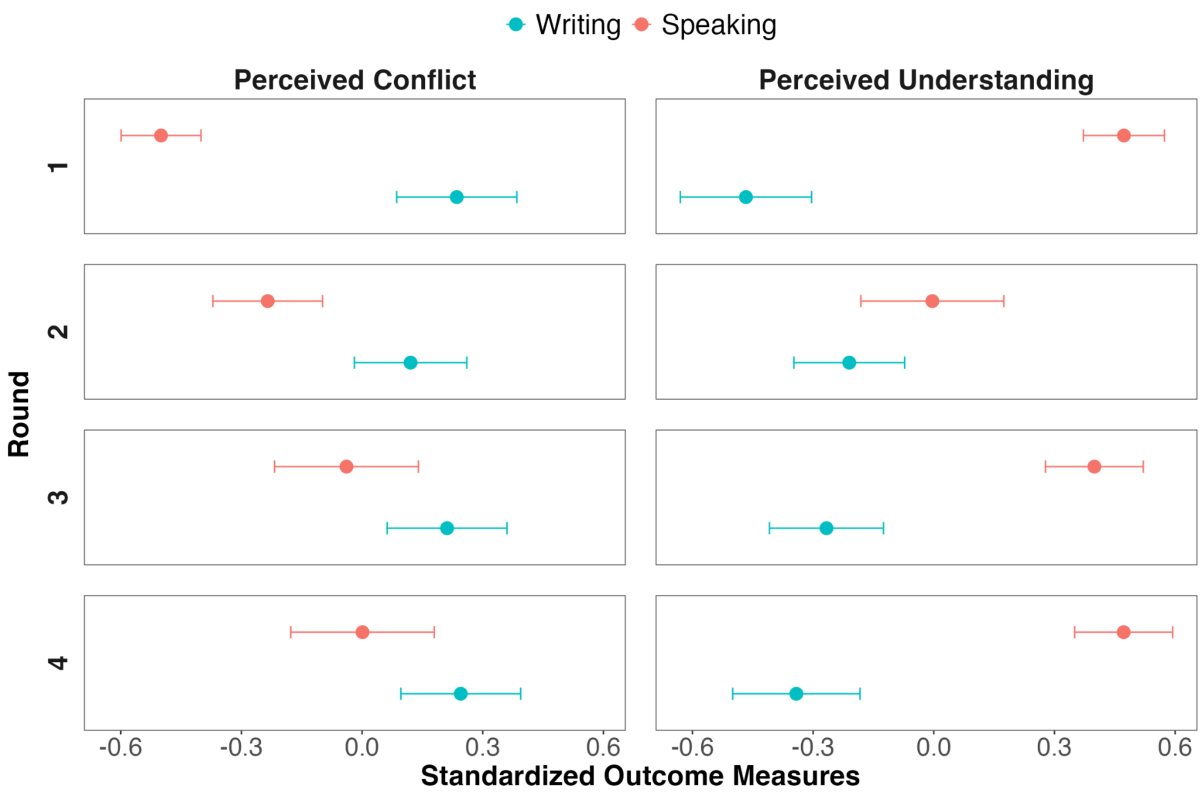

Effects of campus site for initially disagreeing pairs. We also estimated the effect of medium for each campus site among only the initially disagreeing pairs. Across all three sites, we found that speaking significantly increased perceived understanding compared to writing (UC Berkeley: standardized β = 1.36, SE = .34, t(18) = 4.05, p = .001, 95% CI [.70, 2.02]; MNSU: standardized β = 1.04, SE = .23, t(44) = 4.53, p < .001, 95% CI [0.59, 1.49]; ASU: standardized β = .82, SE = .26, t(36) = 3.15, p = .003, 95% CI [.31, 1.32]). We also found that speaking did not statistically significantly reduce perceived conflict compared to writing for each campus site (UC Berkeley: standardized β = -.83, SE = .53, t(18) = -1.58, p = .132, 95% CI [-1.86, .20]; MNSU: standardized β = -.56, SE = .31, t(44) = -1.80, p = .079,

95% CI [-1.16, .05]; ASU: standardized β = -.23, SE = .23, t(36) = -1.01, p = .321, 95% CI [-.69, .22]). See Figure 3 in the main text to visualize these results.

Controlling for initial attitude difference. As an additional robustness check, we re-ran the analysis with initial attitude difference included as a control variable rather than a moderator. To do so, we pooled all post-survey data collected across the three campus sites - including the initially agreeing and disagreeing pairs. As attitude distance is a dyadic level construct, we restrict our analysis to complete dyads where both participants reported their attitudes on topics and completed their post-surveys (n = 358 post-surveys). Speaking maintained its positive effect on perceived understanding (β = .61, SE = .10, t(354) = 6.42, p < .001, 95% CI [.42, .80]) and its negative effect on perceived conflict (β = -.31, SE = .10, t(354) = -3.10, p = .002, 95% CI [-0.51, -.11]).

Supplementary Note 5 - The Role of Individual Differences (Studies 1-3)

Supplementary Table S4. Data pooled from across Studies 1, 2 and 3. Individual level variables include age, gender, big five personality types (openness to experience, conscientiousness, agreeableness, extraversion, and neuroticism), difference in position with one's partner, position strength, and ideology. All regressions are clustered at the dyad, individual, and session levels, controlling for topic and experiment. Age has been centered, whereas other continuous variables such as the personality scales, ideology, position distance and strength were standardized (with a mean of zero and a standard deviation of one). Categories of gender other than male and female were excluded from this analysis. Results show that differences in personality, age, gender, topic position, strength in position and ideology do not significantly affect the relationship between conversation medium and perceived understanding and conflict.

	Perceived Understanding	Perceived Understanding	Perceived Understanding	Perceived Conflict	Perceived Conflict	Perceived Conflict
	(1)	(2)	(3)	(4)	(5)	(6)
Medium: Speaking	0.446***	0.396***	0.403***	-0.227***	-0.204**	-0.157
	(0.068)	(0.058)	(0.085)	(0.062)	(0.064)	(0.089)
Age		-0.005	-0.017		0.007	-0.001
		(0.007)	(0.011)		(0.009)	(0.009)
Gender: Female		-0.066	-0.046		0.091	0.110
		(0.057)	(0.087)		(0.059)	(0.086)
Gender: Other		-0.023	-0.592*		-0.011	0.012
		(0.207)	(0.275)		(0.212)	(0.251)
Agreeableness		0.033	0.064		-0.045	-0.037
		(0.028)	(0.050)		(0.024)	(0.035)
Extroversion		-0.044	-0.095*		0.017	0.043
		(0.030)	(0.046)		(0.028)	(0.046)
Neuroticism		0.018	-0.017		-0.035	-0.042
		(0.033)	(0.052)		(0.025)	(0.043)
Openness		0.013	-0.006		0.002	0.039
		(0.024)	(0.040)		(0.023)	(0.035)
Conscientiousness		0.010	0.012		-0.021	-0.027
		(0.027)	(0.035)		(0.027)	(0.046)
Position difference		-0.007	-0.009		-0.003	0.002
		(0.034)	(0.053)		(0.035)	(0.043)
Position strength		-0.078	-0.046		0.023	-0.057
		(0.051)	(0.058)		(0.045)	(0.061)
Ideology		0.042	0.068		-0.011	-0.052
		(0.029)	(0.042)		(0.025)	(0.042)
Speaking*Age			0.020			0.012
			(0.013)			(0.015)
Speaking*Female			-0.037			-0.043
			(0.119)			(0.106)
Speaking*Other Gender			0.845*			-0.010
			(0.338)			(0.467)
Speaking*Agreeableness			-0.060			-0.019
			(0.060)			(0.047)
Speaking*Extroversion			0.087			-0.048
			(0.060)			(0.050)
Speaking*Neuroticism			0.066			0.018
			(0.064)			(0.055)
Speaking*Openness			0.041			-0.071
			(0.051)			(0.051)
Speaking*Conscientiousness			-0.006			0.013
			(0.052)			(0.061)
Speaking*Position difference			0.002			-0.008
			(0.062)			(0.058)
Speaking*Position strength			-0.065			0.141
			(0.078)			(0.066)
Speaking*Ideology			-0.043			0.078
			(0.055)			(0.057)
Topic F/E	YES	YES	YES	YES	YES	YES
Experiment F/E	YES	YES	YES	YES	YES	YES
Observations	1590	1481	1481	1590	1481	1481
Degrees of Freedom	1578	1461	1450	1578	1461	1450
R^2	0.065	0.061	0.068	0.022	0.019	0.025
Adjusted R^2	0.059	0.049	0.049	0.015	0.007	0.005

Note: Standard errors shown in parentheses
*p<0.05; **p<0.01; ***p<0.001

Note: Personality and ideology measures were collected only in Studies 1 and 2.

Supplementary Note 6 - Interaction Effects of Consumed and Produced Media

Supplementary Table S5. Regression showing the main and interaction effects of consumed and produced media on the primary dependent measures collected in Study 4. All models are estimated using ordinary least squares linear regression at the conversation level. Statistical tests are two-sided, with 95 percent confidence intervals based on a normal approximation. No adjustments were made for multiple comparisons. Results show that the produced medium has an effect on perceived understanding, humanization, agreement, responsiveness, and common ground. In contrast, the consumption medium has no statistically significant effects when accounting for interaction effects. However, there were statistically significant main effects reported in Study 4 for consumption on perceived conflict. Interaction effects are significant for perceived understanding, conflict, agreement, responsiveness, enjoyment, and common ground.

	Perceived Understanding	Perceived Conflict	Perceived Humanisation	Perceived Competence	Agreement	Perceived Responsiveness	Liking	Enjoyment	Common Ground
	(1)	(2)	(3)	(4)	(5)	(6)	(7)	(8)	(9)
Generate: Audio	0.397***	-0.090	-0.200*	-0.171	0.558***	0.833***	-0.170	0.118	1.081***
	(0.100)	(0.100)	(0.099)	(0.106)	(0.135)	(0.174)	(0.127)	(0.113)	(0.182)
Consume: Audio	-0.058	-0.073	0.097	0.001	-0.133	-0.182	-0.085	-0.055	-0.088
	(0.084)	(0.080)	(0.082)	(0.069)	(0.071)	(0.132)	(0.086)	(0.095)	(0.097)
Produced*Consumption (Audio)	0.320**	-0.310**	0.049	0.063	0.354***	0.410*	0.174	0.352**	0.279*
	(0.106)	(0.113)	(0.122)	(0.118)	(0.098)	(0.190)	(0.145)	(0.133)	(0.139)
Observations	971	971	971	971	971	971	971	971	971
Degrees of Freedom	88	88	88	88	88	88	88	88	88
R^2	0.085	0.033	0.012	0.006	0.134	0.119	0.002	0.027	0.156
Adjusted R^2	0.082	0.030	0.009	0.003	0.134	0.117	0.001	0.024	0.154

Note: Parentheses show standard errors
*p<0.05; **p<0.01; ***p<0.001

Supplementary Note 7 - Annotation Study: Dividing Conversations into Exchanges

Although Study 4 suggested that the content of conversations changes as a result of the medium in which they occur, it did not reveal the nature of those changes. In the annotation study, we make a first attempt to provide greater insight into what is happening within the conversations themselves. To do this, we recruited a new set of online workers (i.e., annotators) to evaluate each conversation (all shown via text - either the communicators' original writing or a transcription of their original speech) on four aspects: how much the pairs appeared to understand, have conflict with, agree with, and like each other. We also divided the conversations into discrete exchanges, which were evaluated separately, so that we could analyze the time course of these conversations.

We hypothesized that the annotators would perceive spoken and written conversations to differ in their levels of conflict, understanding, agreement, and liking (such that spoken conversations show less conflict but more understanding, agreement, and liking) across the exchanges. We explored how early these observed differences would emerge in the conversation. We also anticipated that there would be an effect of the duration of conversation, such that conflict would reduce, but understanding, agreement, and liking would increase over time. Finally, we explored whether the spoken and written conversation would follow similar trajectories of change (in perceived understanding, conflict, agreement, and liking) throughout the conversation.

Methods

We preregistered the study design, hypotheses, and analyses here: https://aspredicted.org/KKG_FUO

Participants. In total, we recruited 475 participants. All participants consented, but 14 did not finish the study, leaving a final sample of 461 participants (44.3% Female, Mage = 36.4, SDage = 10.7). Attrition did not vary between the speaking and writing conditions

(chi-squared (1) = 1.45, p = .23). On average, each conversation exchange was rated by 5.1 participants.

Protocol. We told participants that they would review a conversation that took place in a laboratory experiment at the [university name redacted], where each participant in the conversation was asked to discuss a specific political topic about which they disagreed. Similar to previous studies, we asked participants to state their own position and how strongly they felt about controversial speakers on campus, reparations for slavery and legalizing drugs - the same topics given to participants in Study 2B, from where the conversations were drawn (i.e., the 12-minute conversations, n = 89 conversations, 45 spoken and 44 written). We then randomly assigned each participant to read one conversation. Although participants were blind to who the participants in the conversations were, we told them whether the conversation was a spoken or written conversation.

Each participant rated one conversation, and each conversation was divided into a series of exchanges - discrete topics that subdivided the natural flow of the discussion. This allowed us to measure how perceptions of the conversation changed as it progressed. Participants read the exchanges one at a time, in order, from their assigned conversation. After reading each exchange, participants rated the level of understanding, conflict, agreement, and liking of the original communicators. Finally, we asked participants to provide demographic information.

Measures. After reading each exchange, participants completed four items, each measuring their perceptions of understanding, conflict, agreement, and liking in the exchange (see measures in Supplementary Note 15). For example, to measure perceived conflict, participants rated: "How much conflict occurred between the pair in Exchange X" (1 = no conflict, 7 = a lot of conflict).

Assigning exchange groups. We preregistered an analysis plan which involved aggregating the conversational exchanges into five stages: the initial exchange ("start"), the first middle exchange ("middle 1"), the second middle exchange ("middle 2"), the third middle exchange ("middle 3"), and the final exchange ("end").

Two trained annotators divided the conversations into discrete exchanges - stretches of turns that focused on one substantive point, where both participants contributed at least one turn discussing a single argument or belief. Each transcript was segmented into exchanges by one RA and checked by the other, with disagreements resolved via discussion. The RA segmented the transcripts into the first exchange, the last exchange, and three "middle" exchanges (middle 1, middle 2, and middle 3). The below shows how the middle exchanges (applied to conversations that contained between 3 and 15 exchanges) were coded using the following preregistered aggregation scheme:

- 15 exchanges: exchanges 2-5 will be "middle 1", exchanges 6-10 will be "middle 2", and exchanges 11-14 will be "middle 3"
- 14 exchanges: exchanges 2-5 will be "middle 1", exchanges 6-9 will be "middle 2", and exchanges 10-13 will be "middle 3"
- 13 exchanges: exchanges 2-5 will be "middle 1", exchanges 6-8 will be "middle 2", and exchanges 9-12 will be "middle 3"
- 12 exchanges: exchanges 2-4 will be "middle 1", exchanges 5-8 will be "middle 2", and exchanges 9-11 will be "middle 3"
- 11 exchanges: exchanges 2-4 will be "middle 1", exchanges 5-8 will be "middle 2", and exchanges 9-10 will be "middle 3"
- 10 exchanges: exchanges 2-4 will be "middle 1", exchanges 5-6 will be "middle 2", and exchanges 7-9 will be "middle 3"

- 9 exchanges: exchanges 2-3 will be “middle 1”, exchanges 4-6 will be “middle 2”, and exchanges 7-8 will be “middle 3”
- 8 exchanges: exchanges 2-3 will be “middle 1”, exchanges 4-5 will be “middle 2”, and exchanges 6-7 will be “middle 3”
- 7 exchanges: exchanges 2-3 will be “middle 1”, exchange 4 will be “middle 2”, and exchanges 5-6 will be “middle 3”
- 6 exchanges: exchange 2 will be “middle 1”, exchanges 3-4 will be “middle 2”, and exchange 5 will be “middle 3”
- 5 exchanges: exchange 2 will be “middle 1”, exchange 3 will be “middle 2”, and exchange 4 will be “middle 3”
- 4 exchanges: exchange 2 will be “middle 1”, exchange 3 will be “middle 2”, and exchange 3 will be “middle 3”
- 3 exchanges: exchange 2 will be middle 1, 2, and 3

Figure S5 shows the distribution of the number of exchanges across all 89 conversations used in the annotation study. An outlier conversation with 18 exchanges was excluded from analysis.

Supplementary Figure S5. Distribution of number of exchanges in a conversation. The x-axis represents the total number of exchanges in a conversation, and the y-axis represents the number of conversation pairs with that exchange count.

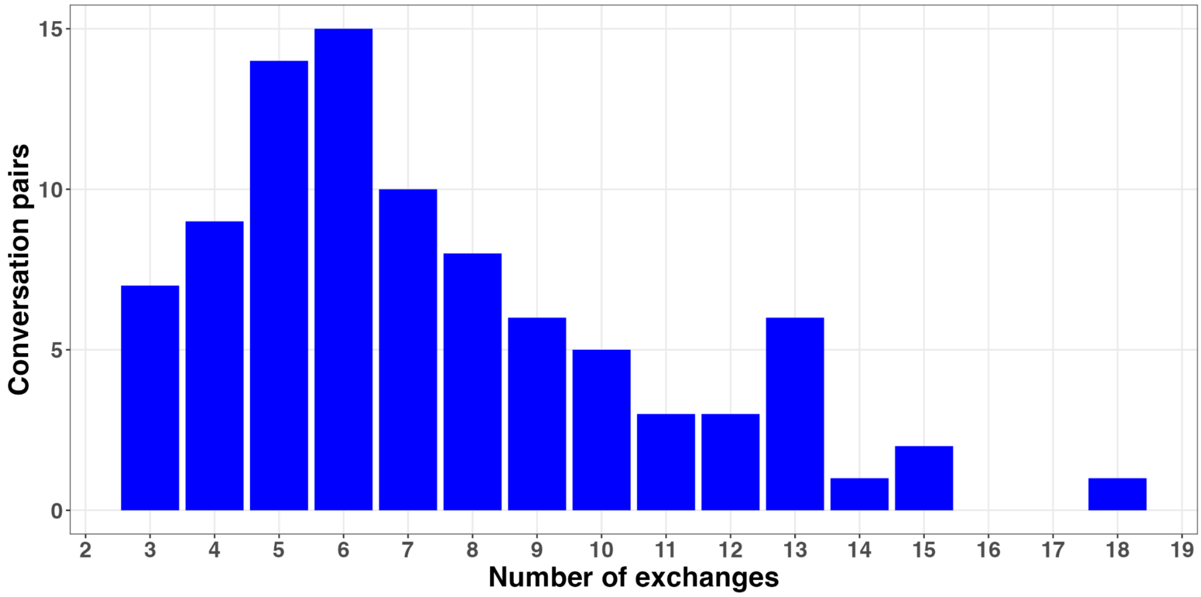

Results

We regressed medium on each dependent variable at the exchange level, controlling for topic and clustering by dyad and session. We tested whether perceptions of understanding, conflict, agreement, and liking changed throughout the conversation. Figure S6 shows the time trends for each variable across the five stages. The patterns suggest that the effect of condition over time varied considerably across stages.

For the very first exchange, during which pairs introduced themselves and, more rarely, started to discuss the topic of disagreement, observers perceived the spoken conversation partners to have slightly *lower* understanding (Speaking: M = 4.65, SD = 1.51; Writing: M = 4.98, SD = 1.55; standardized $\beta = -.21$, SE = .14, t(441) = 1.54, p = .125), higher conflict (Speech: M = 2.37, SD = 1.62; Writing: M = 1.92, SD = 1.55; standardized $\beta = .29$, SE = .11, t(441) = 2.72, p = .007), directionally lower liking (Speaking: M = 4.56, SD = 1.14; Writing: M = 4.76, SD = 1.13; standardized $\beta = -.14$, SE = .12, t(441) = 1.14, p = .255), and less agreement (Speaking: M = 4.18, SD = 1.66; Writing: M = 4.62, SD = 1.67; standardized $\beta = -.25$, SE = .17, t(441) = 1.53, p = .127) than the written conversation partners. Notably, these patterns are the opposite of the usual results; a potential reason is that

speakers were more likely to start discussing their disagreement in the very first exchange than writers.

However, in the subsequent exchanges (i.e., middle 1, middle 2, middle 3, and the ending exchange), we found that the effects flipped and were consistent with the interactants' own ratings. In those later stages, perceived understanding (Speaking: M = 5.28, SD = 1.40; Writing: M = 4.89, SD = 1.64; standardized $\beta = .35$, SE = .10, $t(2346) = 3.37$, $p = .001$) was higher in speaking than writing, perceived conflict was lower in speaking (M = 2.49, SD = 1.59) than writing (M = 2.99, SD = 1.84; standardized $\beta = -.37$, SE = .09, $t(2346) = 4.10$, $p < .001$). Liking was higher in speaking than writing (Speaking: M = 5.09, SD = 1.22; Writing: M = 4.78, SD = 1.27; standardized $\beta = .35$, SE = .10, $t(2346) = 3.53$, $p < .001$). Lastly, agreement was higher in speaking than in writing (Speaking: M = 4.93, SD = 1.51; Writing: M = 4.33, SD = 1.79; standardized $\beta = .48$, SE = .12, $t(2346) = 3.99$, $p < .001$).

Supplementary Figure S6. Annotation Study observers' ratings of conversation exchanges for the spoken and written long conversations (from Study 2B) for the first exchange ("start"), the middle exchanges ("middle 1, 2, and 3") and the last exchange ("end"). The horizontal axis shows how outcome measures change as the conversation progresses over time for speech and text. The vertical axis shows observers' Likert scale ratings for perceived agreement, conflict, liking, and understanding in the conversation. All data points represent group means at each exchange stage. Group means are calculated as simple arithmetic means, rather than estimated coefficients derived from regression models. Consequently, these means do not account for control variables, such as word count. Error bars show the standard error of the mean.

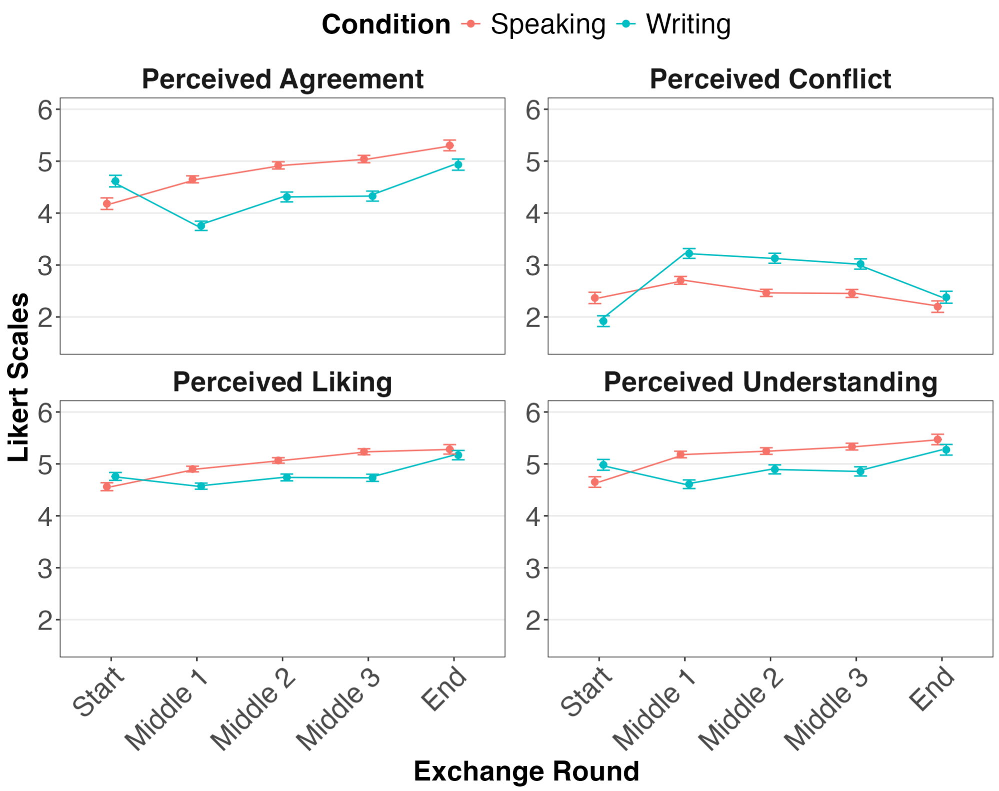

Supplementary Table S6. Randomly selected samples of conversation exchanges across speaking and writing conditions for each exchange category.

	**Examples of Spoken Exchanges**
Start	[1] "We're talking about reparations, right? Yeah. Okay. Cool. Do you want to go first? I don't know. I think we could just be like, do you agree or not first. Oh, wow it got really loud. Oh yeah. It did. Oh wait. Let me turn it down. Yeah, so I opposed. Oh, well I agreed."<br><br>[2] "Okay, Hello. Okay. Hi, I'm Phoebe. Very nice -- yeah -- talking to you. Okay. So do you oppose or support requiring government reparations for slavery? Yeah, I support it. How about you? I oppose it. Okay. So shall I give the reasons I support it and then you can tell me the reason why you don't support it and we can go from there? Should we just go one reason by one reason or just... Yeah, we can go one reason by one reason. Just so we can have more of a conversation. Yeah sure. Okay."<br><br>[3] "Alright. How's your day going? Not too bad. How about yours? Really slow. That's why I'm here. Alright. Do you want to start?"
Middle 1	[1] "Basically, my stance is that we should not legalize drugs because I don't think legalizing drugs would cause -- would solve the underlying problems causing epidemics of addiction. I think certainly with drugs like marijuana or LSD or drugs where their effect is minimal or their effect is not as bad as the enforcement actions are, I think those kinds of

	drugs it would make sense to legalize, but drugs like heroin, methamphetamines, oxycodone pills, I don't think legalizing them would solve the underlying problems like the heroin epidemic. A large contributor to that was overprescription of opioids by doctors and pharmaceutical companies pushing opioid prescription for illnesses that wouldn't really be helped by opioids, and methamphetamines and oxycodone is usually an epidemic in rural areas that used to be in the former manufacturing belt and that's caused by poverty and economic anxiety. So I don't think legalizing those types of drugs would really help anyone. Yeah, I totally agree actually that it doesn't necessarily solve the underlying problems of addiction and also I totally agree that the underlying causes do include a lot of the things that you just listed. My perspective I guess is that it's not necessarily -- the goal is not necessarily to solve addiction and completely solve the opioid epidemic because I think that that's a really complicated issue and there's a lot of different realms that we need to work in to make that happen. But it's about a few things, first of which I think would be harm reduction and I think that there's multiple ways to go about reducing the issue -- like reducing the number of people that overdose on these kinds of drugs that would get into diff -- scary situations because they're under the influence of these kinds of drugs. But I think that this is one option of doing so and that to legalize these drugs would make it easier for people or less frightening for people to seek help. It would also potentially -- I mean, ideally there would be a lot of money that could go out of the incarceration system, which I just think is colossally unjust and oppressive and ultimately shouldn't exist at all. And I mean my -- I mean I think that we shouldn't have prisons, but if there are going to be them, I think that to divert people from that system would be -- like as many people as we possibly can -- would be positive. I also think that legalizing a lot of these drugs would potentially have the effect of you know, prosecuting less people of color and incarcerating less people of color, which is, I mean drug prosecution is just a weapon of oppression against these communities. And I think that if we were to eliminate that -- and then there's also the fact that these drugs would be ideally more regulated and safer and potentially less -- obviously they're not going to be, they're not -- ideally no one would be using these hard drugs, but if they are going to be, which we're not going to stop everyone from using anytime soon -- so to accept the fact that they're going to be and to make sure that they're doing it in the safest way possible, I think is a good idea."<br><br>[2] "I think there's a lot of issues with drugs. I think making weed legal was a good call, but I think certain drugs like heroin and opium and meth and whatever currently should not be illegal because there's no point in making it easier to access because there's just so many issues that arise from them. So I mean while people can access it, I think we'd have more problems if it was easier accessible, like guns for instance. I guess that makes sense. But I know I think that drugs, making them illegal doesn't necessarily make them harder to access. I feel like if you definitely, if you wanted a drug you can definitely, it's definitely pretty easy to go out and find it."<br><br>[3] "Yeah. So why do you support it? There's a lot of reasons. I guess primarily to me it doesn't make sense to jail someone for something that doesn't affect other people. So it's kind of like alcohol and weed are legalized 'cause it's not like you're affecting anyone else by doing drugs. Of course your choices can affect others, but just on principle alone, doing a drug only affects you. And then beyond that drug use is really racialized and disproportionately affects minority groups, and criminalization is a waste of money and it doesn't really ever help the addicts. What are your thoughts? So I oppose it because of the simple reason that if you legalize drugs in the US then more people will start taking it who were fearful of taking it and then it would already be more prevalent, and I think maybe even the death would increase because more people will have more free will to take the drugs and they'll start taking it on a more frequent basis and it will become hard to control and there won't be any limits or boundaries to how many drugs people are taking. So, I thought that it's not a good idea to legalize drugs in the US."
Middle 2	[1] "Yeah. People are just not willing to change or to be different. Mm, yeah that's a good point. I feel that's a good point that's relevant. Because people are kind of already so set in the way that the system is, I think most people probably already think that this is just the way that it should be or this is just the way that the world is and so I feel that also kind

	of complicates things."<br><br>[2] "Yeah. Yeah. And then actually the last -- about the -- I didn't quite catch the last thing you said about as far as the supply... could you repeat that possibly? Oh, I meant -- this is more in the -- for heroin, I think the heroin supply is more to do with doctors overprescribing opioids and Pharma pushing for opioids. But with things like methamphetamines and oxycodone, these -- methamphetamine is a rural problem. It's manufactured at home in mostly rural areas. So if you still prosecuted the manufacturers of methamphetamines, the poor people that are preyed on by the suppliers, I think they would get some benefit if you prosecuted the suppliers and things like oxycodone pills which are pushed by a lot of -- especially a lot of rural doctors, especially in Florida who provide oxy and other pills in large quantities to suppliers who then go out to rural communities and push pills onto the poor and the rural communities. If you prosecuted the suppliers and the doctors providing those pills, I think it would reduce the supply of drugs in the market and I think it would be ultimately more beneficial. Yeah, I totally understand that. I guess that -- I guess the -- I guess what's interesting is the way -- okay, I guess the regulations that would come with this legalization are kind of unknown to us, we're just talking about broad legalization and I guess I don't -- I think there's definitely some incarnations of that that I would think are problematic and I would hope that -- and it's complicated, so that's why I have, I do have semi-mixed feelings about this issue because I think that setting up the rules for a world in which -- or a country in which these drugs are legal, could be complicated. And ideally it would, there -- I don't really know what this would look like and maybe this is kind of idealistic -- but the preying upon populations who are susceptible to this kind of addiction in rural areas, like you're saying, would somehow be regulated. I'm not exactly, that's the thing, I'm not exactly sure what that would look like -- I guess the marijuana industry, it would be like legit dispensaries."<br><br>[3] "Because it is kind of-- it's not super, I guess, recent? Or I mean, I guess, it is recent because it was last year, but not recently as in, I guess, a few months ago or within 2018? Yeah, but what kind of -- It still goes on today, I'm just, you know, just kind of ignorant about it. Right, yeah, I'm not too up to date on current events and stuff, but I don't know, I just feel like in general, I'd want to allow almost -- or everyone to come speak, who wants to. I think the campus should be a program -- like a platform."
Middle 3	[1] "But see, we legalized it and there's, I don't know, I think you're still gonna lose some, but you're also gonna win some because can you imagine people smuggling alcohol during the prohibition era? They decided to legalize it. So I think there are pros that come along to legalizing something that's bad because they track it. Definitely. Yeah. I mean as you were saying, if you had to buy marijuana from someone who's selling it legally, you can track. I think you brought up a point of tracking how much -- who buys how much and all that."<br><br>[2] "Yeah. That's something that I actually wrote down in my notes was also, \"how would we regulate what they spend this money on,\" because if we are giving them money to help mend some of the negative feelings about slavery, it shouldn't just be, \"Oh here's money. Go ahead buy whatever you want with it.\" So yeah, I definitely agree with you on there should be some sort of regulation if that's a thing. So I think actually we agree on this point-- Yeah. --rather than disagree. So there definitely should be something government need to do for these communities and then money is one of the element that needs to be regulated, while there are some elements that government can do to help. Yeah?"<br><br>[3] "It's not safe if it's legal either. I mean if it's legal then the government -- or not government but companies -- could regulate it, or you know how you regulate food?"
End	[1] "Alright, well I guess we're done, but thank you. Yeah, for sure. Alright."

	[2] "I think most people in the US, they would have insurance. I know that a lot of people don't have insurance but the majority of people still have insurance and that would cover like maybe not all of the people who use drugs, but many of them at least. I highly doubt that because the people -- majority of my family don't have health insurance and majority of the people that I know that are addicted to drugs, including people who are alcoholics that need support, also don't have health insurance. So I'm pretty, pretty sure that majority of the US does not have health insurance or if they do, they can't really afford their premiums to pay for health insurance. So it would depend if insurance would be covered or if healing for drug addicts would be covered. It had to be covered on a basic level, which would be extremely expensive for the government because -- and insurance companies-- because becoming sober from any drug, even alcohol, takes a lifetime because it's an everyday process. So as the other thing, I don't really think the government will really be happy with that because one, we could get a patient who's addicted to any kind of drug, whether it's alcohol, heroin, cocaine, that kind of addiction lasts an entire lifetime. It doesn't end after a couple of years, five or 10 years, you could relapse after 20 years of not having a certain drug. And this again, I have experienced, both my parents are alcoholic-- were alcoholics when they were younger and had been sober for 40 years. They had a friend who was sober for 50 years and one day they just slipped up and had a drink and they relapsed and they ended up dying within a month and a half and it had been 50 years of them not having a drink. So it's something that seems a really expensive kind of system just to legalize."<br><br>[3] "Yeah, we should always strive for that perfect world, honestly. Yeah. But, I think, I'm not sure I have anything else to say about it. I really don't think so either."
	**Examples of Written Exchanges**
Start	[1] "Hello! Hi! Not really sure what to do haha"<br><br>[2] "Hello! Hi I'll just write out the question here for the sake of it: Do you oppose or support government reparations for slavery?"<br><br>[3] "hello Hi"
Middle 1	[1] "what do you think about government reparations for slavery?? Person B: I would say that I'm opposed to offering reparations for slavery What do you think? Person A: for the most part, i would say that i'm for offering reparations but i'm not super strong on my stance i don't feel like i know enough about it to feel very strongly about it why are you opposed?"<br><br>[2] "Person B: What is your opinion on the topic Person A: I think that the government should provide reparations for slavery Person B: I chose to mildly disagree with the subject, but only because I was unsure of how the government would plan to do this"<br><br>[3] "Person A: I was in support for the legalization of drugs in the US what are your thoughts? Person B: Well, I'm totally disagree with that I don't think we should legalize drugs in the US"
Middle 2	[1] "I haven't seen the numbers on legal vs. illegal marijuana in California, but it would be interesting to look at it after the legalization. So US Govt. ends up spending a good amount in this anyway"<br><br>[2] "Person B: The question might even boil down to is everyone actually entitled to their own opinion Esp when it's just hateful and disrespectful... Really hard question to answer Person A: for sure. I just prefer to keep an open mind, and a space for everyone to share their opinions. Frankly Berkeley is very liberal, so much that it's a bubble and I think it's good for the students to see that other opinions exist even if we know they are wrong"

	[3] "Person A: I agree that if the speaker specifically is threatening to physically harm someone, they should not be allowed to come. But, from my knowledge, that was not the case in either cancellation. If anything, it was outside forces, and or protesters that created the violence/physical harm Person B: That's a good point, but oftentimes these events are not only events in a small room on campus that no one notices, rather the University has to spend money, from everyone's tuition as well, to provide space for these big-name speakers I know that hate-speech is a buzzword these days, but if we look at history, the effects of even a small gathering can snowball into much greater catastrophes if the ideas said aloud and shared and built on are full of hate That's why I think that if there is public outcry, which you're right, should definitely be peaceful, then the University should reconsider"
Middle 3	[1] "Person B: For example, my ex's best friend is a huge fan of meth and she gets violent Person A: But would she benefit from being branded as a criminal? Person B: I'm not sure why, I don't know enough I'm not saying she should be branded as a criminal necessarily. But i don't think it shold be made readily available to her i just don't think the government should endorse it"<br><br>[2] "Person A: Because not only does it acknowledge and help the black community, it helps to mold public thought on the issue which is equally if not more important Person B: But that is something that can't be changed Agreed If the government is taking a concrete stance on something, then that helps formulate a citizen's mind Person A: Yes, the time that has passed and the loss/destruction of records is hard We agree"<br><br>[3] "Person A: I see what you are saying, but I don't think that legalization would suddenly create a population of addicts There are examples of other countries in the world that have legalized drugs, such as Portugal, and their country is great Very low crime and HIV rates Person B: Ideally, government could help those who got addicted. But in the reality, it wouldn't be so easy.."
End	[1] "Person B: im glad we had this talk Person A: Yupp Person B: i will take some of ur ideas and reflect on them more"<br><br>[2] "The main reason why I want drugs to be legalized is because the enforcement and incarceration of individuals who use/traffick drugs is highly racialized And statistically from othercountries who have legalized drugs,the rates of addiction and drug use actually go down what do you mean? highly racialized as in targeting certain races? Byeee poor black and latino communities are highly targeted hah bye ahh yes they are"<br><br>[3] "oh nice! hope youre enjoying cal Thank you though! Nice talking to you You too!"

Although Studies 1-3 showed that perceived understanding was significantly higher in speech than in writing. In contrast, perceived conflict was significantly lower, the annotation study indicates that understanding and conflict are not perceived uniformly across the duration of a conversation. Instead, we found that, in pairs' initial exchange, written conversations were in fact perceived to contain less conflict and more understanding,

agreement, and liking than spoken conversations. However, after the first exchange, written conversations contained more conflict and less understanding, agreement, and liking than the spoken conversations. In the main text, we investigate which aspects of the language used in spoken and written conversations lead to more constructive disagreement outcomes (see Language Analyses section).

Supplementary Note 8 -Analysis of Spell-Checked Language (Study 1)

To test the impact of spell-checking, the effects of receptiveness (Table S7) on perceived understanding and conflict and the effect of medium on receptiveness (Table S8) were re-evaluated using spell-checked transcripts and written conversations from Study 1. For all regressions, we control for topic, clustering standard errors by dyad and session. The results indicate that using a spell-checked transcript does not alter the estimated effect of receptiveness or medium. Note that the effects are weaker than those reported in the main text, which pool data from Studies 1 and 2, due to using the smaller sample size of just Study 1.

Supplemental Table S7. The effects of receptiveness on perceived understanding and conflict for the original and spell-checked conversations in Study 1. All models use ordinary least squares linear regression with two-sided tests. Topic fixed effects are included, and standard errors are clustered at the dyad and session levels. No adjustments were made for multiple comparisons.

	Perceived Understanding	Perceived Understanding	Perceived Conflict	Perceived Conflict
	(1)	(2)	(3)	(4)
Receptiveness	0.239***		-0.105	
	(0.058)		(0.055)	
Receptiveness (spell checked)		0.234 ***		-0.107*
		(0.056)		(0.054)
Topic F/E	YES	YES	YES	YES
Experiment F/E	YES	YES	YES	YES
Observations	266	266	266	266
Degrees of Freedom	262	262	262	262
R^2	0.082	0.079	0.016	0.019
Adjusted R^2	0.071	0.069	0.004	0.005

Note: Parentheses show standard errors
*p<0.05; **p<0.01; ***p<0.001

Supplementary Table S8. The effect of medium on receptiveness for the original and spell-checked conversations in Study 1. All models use ordinary least squares linear regression with two-sided tests. Topic fixed effects are included, and standard errors are clustered at the dyad and session levels. No adjustments were made for multiple comparisons.

	Receptiveness	Receptiveness	Receptiveness (Spell checked)	Receptiveness (Spell checked)
	(1)	(2)	(3)	(4)
Medium: Speaking	0.439***	0.197	0.446***	0.195
	(0.086)	(0.135)	(0.084)	(0.131)
Word Count		0.158		0.251
		(0.102)		(0.094)
Topic F/E	YES	YES	YES	YES
Experiment F/E	YES	YES	YES	YES
Observations	266	266	266	266
Degrees of Freedom	262	261	262	262
R^2	0.067	0.078	0.067	0.079
Adjusted R^2	0.056	0.064	0.056	0.065

Note: Parentheses show standard errors
*p<0.05; **p<0.01; ***p<0.001

Supplementary Note 9 - Effect of Medium on Receptive Language (Studies 1-2)

We test the effects of conversation medium on receptiveness in data from Studies 1 and 2. For all regressions, we control for topic and word count, clustering standard errors by dyad and session. In Study 2B, the length of the conversations was manipulated such that total word count varied more drastically between conditions. Results in Table S9 show that controlling for word count reduces the effect of medium on conversational receptiveness across Studies 1 and 2.

Supplementary Table S9. The effects of conversation medium and word count on receptiveness in Studies 1 and 2. All models are estimated using ordinary least squares linear regression with two-sided tests. Topic and word count are included as controls, and standard errors are clustered at the dyad and session levels. No adjustments were made for multiple comparisons.

	Dependent variable: Conversational Receptiveness							
	Study 1	Study 1	Study 2A	Study 2A	Study 2B	Study 2B	Study 2C	Study 2C
	(1)	(2)	(3)	(4)	(5)	(6)	(7)	(8)
Medium: Speaking	0.359***	0.203	0.255***	0.389**	0.360***	0.106	0.657***	0.480**
	(0.090)	(0.139)	(0.097)	(0.160)	(0.131)	(0.154)	(0.079)	(0.139)
Word count		0.163**		0.073		0.335**		0.147*
		(0.104)		(0.101)		(0.122)		(0.068)
Topic F/E	YES	YES	YES	YES	YES	YES	YES	YES
Observations	292	266	370	362	410	396	414	374
Degrees of Freedom	288	262	366	357	406	391	410	369
R^2	0.054	0.078	.074	0.080	0.034	0.088	0.130	0.149
Adjusted R^2	0.044	0.064	0.066	0.069	0.027	0.079	0.124	0.140

Note: Standard errors shown in parentheses
*p<0.05; **p<0.01; ***p<0.001

Supplementary Note 10 - Testing Receptive Language as a Mediator (Studies 1-2)

We examine whether receptiveness mediates the effect of conversation medium for our primary dependent variables across all data pooled from Studies 1 and 2. For all regressions, standard errors are clustered by dyad and session, controlling for topic, study, and word count. Each path denotes a facet of the mediation model where (a) is the effect of medium on conversational receptiveness, (b) is the association between conversational receptiveness and each dependent variable, (c) is the total effect of medium on each dependent variable, and (c') is the direct effect of medium on each dependent variable. Note that the effect of medium on conversational receptiveness, path (a), is the same for all dependent variables. The estimated indirect effect reflects the extent to which the effect of the conversation medium on each dependent variable operates through conversational receptiveness, as estimated via nonparametric bootstrapping with 20,000 simulations using the R mediation package.

Supplementary Table S10. Estimated effects of the mediation pathways across primary dependent variables using data pooled from Studies 1 and 2. Brackets show the corresponding 95% confidence interval. All models are estimated using ordinary least squares linear regression with two-sided tests. Topic, study, and word count are included as controls, and standard errors are clustered at the dyad and session levels. No adjustments were made for multiple comparisons.

Dependent Variable	a	b	c	c'	Indirect Effect
**Perceived Understanding**	.18 *	.18 ***	.35***	.31 ***	.04 ** [.01, .07]
**Perceived Conflict**	.18 *	-.12 ***	-.32 ***	-.30 ***	-.02 ** [-.05, .01]
**Perceived Humanisation**	.18 *	.04	.10	.09	.01 [-.005, .02]
**Perceived**	.18 *	.13 ***	.16^	.14	.03 **

**Competence**					[.01, .05]
**Attitude Shift Toward Partner**	.18 *	.05 *	-.03	-.04	.01 * [.002, .03]
**Liking**	.18 *	.11 ***	.22 **	.20 *	.02 ** [.006, .04]
**Enjoyment**	.19 *	.11 ***	.24 **	.22 *	.02 ** [.005, .04]

Note: ^ $p < .1$; * $p < 0.05$; ** $p < 0.01$, *** $p < 0.001$

Supplementary Note 11 - Testing Receptive Language as a Moderator (Studies 1-2)

We test for potential interactions between the conversation medium and conversation receptiveness on our primary dependent variables across data pooled from Studies 1 and 2. For all regressions, we control for topic and study, clustering standard errors by dyad and session. Results in Table S11 show that a) receptiveness is a strong predictor of all dependent variables except for perceived humanisation and attitude shift, and b) receptiveness moderates the relationships between medium and perceived understanding, conflict, competence, and liking. In Table S12, we find similar results when also controlling for word count.

Supplementary Table S11. The effects of conversation medium, conversational receptiveness, and their interaction on the primary dependent variables in data pooled from Studies 1 and 2. All models are estimated using ordinary least squares linear regression with two-sided tests. Topic and study fixed effects are included, and standard errors are clustered at the dyad and session levels. No adjustments were made for multiple comparisons.

	Perceived Understanding	Perceived Conflict	Perceived Humanisation	Perceived Competence	Attitude Shift Towards Partner	Liking	Enjoyment
	(1)	(2)	(3)	(4)	(5)	(6)	(7)
Medium: Speaking	0.301***	-0.131*	0.173**	0.224***	0.045	0.292***	0.307***
	(0.063)	(0.065)	(0.052)	(0.060)	(0.042)	(0.060)	(0.060)
Receptiveness	0.341***	-0.282***	0.073	0.313***	0.075	0.262***	0.225*
	(0.060)	(0.068)	(0.070)	(0.061)	(0.050)	(0.071)	(0.077)
Speaking*Receptiveness	-0.216***	0.220**	-0.045	-0.228***	-0.027	-0.191*	-0.143
	(0.060)	(0.071)	(0.076)	(0.074)	(0.028)	(0.075)	(0.079)
Topic F/E	YES	YES	YES	YES	YES	YES	YES
Experiment F/E	YES	YES	YES	YES	YES	YES	YES
Observations	1486	1486	1486	1486	1486	1486	1486
Degrees of Freedom	1475	1475	1475	1475	1475	1475	1475
R^2	0.081	0.028	0.013	0.053	0.419	0.214	0.145
Adjusted R^2	0.075	0.022	0.007	0.047	0.414	0.209	0.140

Note: Parentheses show standard errors
*p<0.05; **p<0.01; ***p<0.001

Supplementary Table S12. The effects of conversation medium, conversational receptiveness, their interaction, and the word count on the primary dependent variables in data pooled from Studies 1 and 2. All models are estimated using ordinary least squares linear regression with two-sided tests. Topic and study fixed effects are included, and standard errors are clustered at the dyad and session levels. No adjustments were made for multiple comparisons.

	Perceived Understanding	Perceived Conflict	Perceived Humanisation	Perceived Competence	Attitude Shift Towards Partner	Liking	Enjoyment
	(1)	(2)	(3)	(4)	(5)	(6)	(7)
Medium: Speaking	0.276**	-0.261**	0.085	0.097	-0.044	0.163	0.198*
	(0.085)	(0.083)	(0.086)	(0.094)	(0.058)	(0.084)	(0.091)
Receptiveness	0.353***	-0.288***	0.069	0.321***	0.071	0.279**	0.233**
	(0.063)	(0.068)	(0.072)	(0.063)	(0.049)	(0.075)	(0.081)
Speaking*Receptiveness	-0.218***	0.209**	-0.040	-0.251	-0.025	-0.207**	-0.154
	(0.060)	(0.082)	(0.078)	(0.077)	(0.058)	(0.079)	(0.082)
Word count	0.006	0.101**	0.055	0.082	0.060	0.086*	0.072
	(0.033)	(0.029)	(0.044)	(0.045)	(0.024)	(0.035)	(0.040)
Topic F/E	YES	YES	YES	YES	YES	YES	YES
Experiment F/E	YES	YES	YES	YES	YES	YES	YES
Observations	1398	1398	1398	1486	1486	1486	1398
Degrees of Freedom	1386	1386	1386	1386	1386	1386	1386
R^2	0.083	0.032	0.016	0.057	0.431	0.215	0.142
Adjusted R^2	0.075	0.024	0.008	0.049	0.427	0.209	0.135

Note: Parentheses show standard errors
*p<0.05; **p<0.01; ***p<0.001

Supplementary Note 12 - The Effect of Medium on Other Changes in Language (Studies 1-2)

We test the effects of conversation medium on receptiveness, sentiment, word count, and politeness across data pooled from Studies 1 and 2. For all regressions, we control for topic and study, clustering standard errors by dyad and session. Across all regressions, the conversation medium (speaking or writing) significantly affects receptiveness, sentiment, word count and politeness (see Table S13). Politeness was calculated using a pre-trained model (Danescu-Niculescu-Mizil et al., 2013). In Table S14, receptiveness is still significantly correlated with perceived understanding and conflict, whether additional controls are included or excluded.

Supplementary Table S13. The effect of medium on receptiveness, sentiment, word count, and politeness using data in Studies 1 and 2. All models are estimated using ordinary least squares linear regression with two-sided tests. Topic and study fixed effects are included, and standard errors are clustered at the dyad and session levels. No adjustments were made for multiple comparisons.

	Receptiveness<br>(1)	Sentiment<br>(2)	Word count<br>(3)	Politeness<br>(4)
Medium: Speaking	0.430***	0.578***	1.431***	0.458***
	(0.052)	(0.060)	(0.077)	(0.051)
Topic F/E	YES	YES	YES	YES
Experiment F/E	YES	YES	YES	YES
Observations	1486	1394	1398	1486
Degrees of Freedom	1477	1385	1389	1477
R^2	0.081	0.135	0.556	0.095
Adjusted R^2	0.076	0.130	0.554	0.090

Note: Parentheses show standard errors
*p<0.05; **p<0.01; ***p<0.001

Supplementary Table S14. Associations between receptiveness, sentiment, word count, and politeness on our primary dependent variables using data in Studies 1 and 2. All models are estimated using ordinary least squares linear regression with two-sided tests. Topic and study fixed effects are included, and standard errors are clustered at the dyad and session levels. No adjustments were made for multiple comparisons.

	Perceived Understanding	Perceived Understanding	Perceived Understanding	Perceived Conflict	Perceived Conflict	Perceived Conflict
	(1)	(2)	(3)	(4)	(5)	(6)
Receptiveness	0.193***		0.139***	-0.111***		-0.091**
	(0.025)		(0.031)	(0.022)		(0.026)
Sentiment		0.120***	0.098**		-0.127***	-0.112***
		(0.029)	(0.030)		(0.031)	(0.032)
Word count		0.155***	0.114***		-0.011	0.015
		(0.030)	(0.027)		(0.028)	(0.029)
Politeness		0.086**	0.051		-0.029	-0.006
		(0.026)	(0.026)		(0.025)	(0.027)
Topic F/E	YES	YES	YES	YES	YES	YES
Experiment F/E	YES	YES	YES	YES	YES	YES
Observations	1486	1394	1394	1486	1394	1394
Degrees of Freedom	1477	1383	1382	1477	1383	1382
R^2	0.049	0.060	0.077	0.016	0.021	0.027
Adjusted R^2	0.044	0.054	0.069	0.011	0.013	0.020

Note: Parentheses show standard errors
*p<0.05; **p<0.01; ***p<0.001

Supplementary Note 13 - Additional Analyses from Study 6

Supplementary Table S15. Within-participant ratings showing the effect of communication medium on secondary dependent variables (excluding perceived conflict and perceived understanding). Models are estimated using linear mixed-effects regression implemented in the lme4 R package, with participant-level random effects to account for repeated measures. Statistical tests are two-sided. No adjustments were made for multiple comparisons. Video chatting and speaking are combined into a single speaking category. Results show that speaking was expected to be less comforting, more effortful, more awkward, and less enjoyable than writing. There were no significant predictions for partner's competence, likability or the ability to find common ground. There was a small effect on predicted attitude congruence.

	Discomfort	Effort	Awkwardness	Enjoyment	Partner Competence	Partner Liking	Common Ground	Attitude Congruence
	(1)	(2)	(3)	(4)	(5)	(6)	(7)	(8)
Medium: Speaking	-0.616***	0.746***	1.142***	-0.456***	-0.067	-0.059	-0.014	-0.102*
	(0.068)	(0.053)	(0.060)	(0.060)	(0.042)	(0.055)	(0.064)	(0.049)
Observations	606	606	606	606	606	606	606	606

Note: Parentheses show standard errors
*p<0.05; **p<0.01; ***p<0.001

Supplementary Table S16. We explored whether the collected demographic variables (gender, age, race, education, and political ideology) moderated the effect of medium on predictions. Models are estimated using linear mixed-effects regression implemented in the lme4 R package, with participant-level random effects to account for repeated measures. All demographic predictors are mean-centered. Statistical tests are two-sided. No adjustments were made for multiple comparisons. The political ideology is a continuous variable ranging from 1 - 7 where 7 is very conservative and 1 is very liberal. For age, we found an effect on predicted conflict, but did not moderate the effect of medium. For gender, we found that

females predicted less conflict but also did not moderate the effect of medium. No other demographic variables had a significant effect.

	Perceived Understanding	Perceived Understanding	Perceived Conflict	Perceived Conflict
	(1)	(2)	(3)	(4)
Medium: Speaking	-0.119*	-0.135*	0.341***	0.315***
	(0.053)	(0.060)	(0.060)	(0.066)
Age		0.010		0.012*
		(0.005)		(0.005)
Female		0.077		-0.259*
		(0.138)		(0.127)
Ethnic White		-0.055		-0.218
		(0.152)		(0.140)
College Degree		0.017		0.132
		(0.134)		(0.123)
Political Ideology		-0.027		0.040
		(0.058)		(0.053)
Speaking*Age		-0.005		-0.007
		(0.004)		(0.004)
Speaking*Female		-0.014		0.057
		(0.111)		(0.123)
Speaking*White		0.076		0.129
		(0.122)		(0.136)
Speaking*College Degree		0.019		0.093
		(0.108)		(0.120)
Speaking*Political Ideology		-0.044		0.079
		(0.046)		0.052
Observations	606	606	606	606

Note: Parentheses show standard errors
*p<0.05; **p<0.01; ***p<0.001

Supplementary Note 14 - Analyses of Acoustic Features of Speech (Studies 1-2)

To investigate whether aspects of speech influenced our dependent variables, we extracted acoustic features from the speaking conditions in Studies 1, 2A, and 2C using the Librosa library in Python (McFee et al., 2015). We used Librosa because it provides robust and flexible methods for extracting various acoustic features, including state-of-the-art F0tracking (Mauch & Dixon, 2014). We are specifically interested in prosodically relevant acoustic features such as pitch (as approximated by F0), intensity, and tempo, which could play a role in conveying emotion, emphasis, and interpersonal stance.

Speech is a complex, multidimensional signal where temporal and spectral features capture different but complementary aspects of vocal expression. Temporal features such as speech rate, loudness variability, and pitch variation reflect rhythmic patterns and emphasis, whereas spectral features characterize articulation, clarity, and tonal richness. Together, they provide a comprehensive picture of the differences between speeches.

We expect many of these features to be correlated. For example, speech rate and loudness variability may be positively related. Similarly, speech brightness and frequency spread are influenced by high-frequency energy, and speech clarity and crispness may also be correlated, as both contribute to how well-defined speech sounds. However, their relationships are unlikely to be perfectly correlated—one can speak quickly but monotonously or clearly but slowly, making it important to capture each feature independently. Having all these measures allows for a more nuanced interpretation of speech dynamics. Details of all the audio features are briefly described below:

1. **Speech rate** was estimated using the librosa.beat.beat_track() function, which detects the tempo of an audio signal by analyzing onset strength—points where energy changes significantly over time. While tempo is typically measured in beats per minute (BPM) for music, it can also approximate speech rate in conversation, as syllable onsets often follow rhythmic patterns. Because the measure was calculated from the mixed conversational signal, it should be interpreted as an aggregate or conversation-level estimate of speech rate, rather than a precise per-speaker measure. See Ellis (2007).
2. **Loudness variability** was estimated using root mean square (RMS) energy via the librosa.feature.rms() function. This function computes RMS by squaring the amplitude values of the audio waveform, averaging them over a short analysis window, and taking the square root, providing a smooth representation of loudness over time. Unlike peak amplitude, RMS energy captures sustained vocal intensity, making it a more reliable measure of perceived loudness. To quantify variability, the standard deviation of RMS energy is computed across the duration of the audio, reflecting fluctuations in vocal intensity. See McFee et al., (2015).
3. **Pitch variation** was estimated using Librosa's librosa.piptrack() function, which extracts the fundamental frequency (F0) of the speech signal over time. The algorithm computes a spectrogram, identifies frequency peaks, and tracks the dominant frequency contours. By default, piptrack searches across the full frequency range of the spectrogram. To quantify variability, we calculated the standard deviation of the detected F0 values, providing a measure of fluctuations in vocal frequency. While F0 distributions can differ by gender, random assignment produced balanced groups in terms of gender and age, minimizing concerns about systematic bias. See Smith (2022).

4. **Speech crispness** was estimated using zero-crossing rate (ZCR), computed via librosa.feature.zero_crossing_rate(), quantifying how often the audio waveform crosses the zero amplitude axis. See Gouyon et al., (2008).
5. **Speech brightness** was estimated using spectral centroid, computed via librosa.feature.spectral_centroid(), which estimates the center of mass of the sound spectrum, capturing how much energy is concentrated in higher frequencies. See Kapuri & Davy (2007).
6. **Frequency spread** was estimated using spectral bandwidth, computed via librosa.feature.spectral_bandwidth(), which quantifies the range of frequencies present in the speech signal by measuring how widely frequencies are distributed around the spectral centroid. See Kapuri & Davy (2007).
7. **Speech clarity** was estimated using spectral contrast, computed via librosa.feature.spectral_contrast(), which captures the difference in energy between peaks (strong frequencies) and valleys (weaker frequencies) across different frequency bands. See Jiang et al., (2002).

To account for differences in how conversations were recorded across studies, we aggregated all audio features to the dyadic level for consistency. In Study 1 and Study 2B, recordings were collected at the dyad level, where both speakers were recorded in a single recording. In Study 2A, conversations in the multiple exchanges condition were recorded as a single file per dyad, whereas in the single exchange condition, each participant's speech was recorded separately. In Study 2C, recordings in the asynchronous condition were captured separately for each conversational turn, whereas the synchronous condition was recorded at the dyad level. Aggregating to the dyad level ensured consistency in speech feature comparisons across studies, given that most audio recordings contained audio features for

both speakers. We then pooled the data from Studies 1 and 2 and merged participants' survey ratings (on perceived understanding, conflict, competence, humanization, attitude shift, liking, and enjoyment) from our existing dataset.

We were able to find the corresponding audio or audiovisual recordings for all the transcriptions we analyzed in our Language Analyses section (see the number reported in Table S20) except for one recording missing from Study 1. Thus, we extracted auditory features using Librosa in Python on the following recordings: 85 recordings from Study 1, 89 from Study 2A, 98 from Study 2B, and 97 from Study 2C. We pooled these recordings for analysis ($n = 369$). See Table S17 for descriptive statistics.

We conducted exploratory analyses to identify similarities in audio features using Pearson correlations. See Table S18. As expected, we found that loudness variability was correlated with pitch variability, and the spectral features (speech crispness, speech brightness, frequency spread, and speech clarity) were strongly correlated with one another. Speech rate was uncorrelated with all other audio features.

We modeled the effects of each audio feature on all dependent variables at dyad level, controlling for topic and study number, and clustering standard errors by session. The results are reported in Table S19. Speech rate (computed at the conversation level, which may include overlapping talk) had a statistically significant, positive association with perceived understanding (standardized $\beta = .01$, $SE = .01$, $t(350) = 2.17$, $p = .031$), but a negative association with perceived competence (standardized $\beta = -.01$, $SE = .00$, $t(350) = 2.37$, $p = .018$). Speech rate had a positive association with all our other dependent variables but was only statistically significant for perceived competence (standardized $\beta = .09$, $SE = .00$, $t(350) = 2.12$, $p = .034$) and enjoyment (standardized $\beta = .01$, $SE = .00$, $t(350) = 2.57$, $p = .011$). We found that other audio features did not show statistically significant associations with our dependent variables.

These findings highlight associations between speech rate and perceived understanding, conflict, and impressions of competence. It is possible that faster speech rates may convey confidence and cognitive ability, making arguments appear more well-reasoned. These results suggest promising avenues for testing how specific vocal features could influence constructive disagreement, especially speech rate.

As always, there are limitations. Librosa was developed primarily for music information retrieval, and while many of its algorithms (e.g., pitch tracking, onset detection, tempo estimation) can be applied to speech, they are not optimized for speech-specific characteristics. Variability in recording quality, background noise, and microphone differences across participants and conversations further complicates the consistency of spectral features. Most importantly, because auditory features do not apply to written communication, this study cannot assess how medium differences (spoken vs. written) shape disagreements—the core focus of this research. Writers adjust their style to convey emphasis, intonation, and clarity through word choice, but without knowing how they would have verbalized their responses, we cannot determine how much of these speech features would have been represented in text or omitted entirely.

Supplementary Table S17. Descriptive statistics of the auditory features of spoken conversations in Studies 1 and 2.

Audio Feature	N	Mean	SD	Min	Max
Study 1					
Speech Rate (Beats Per Minute)	85	125.41	11.61	93.99	149.83
Loudness Variability (s.d. of waveform aplitudes)	85	0.04	0.01	0.02	0.05
Pitch Variation (Hz)	85	893.30	62.27	742.20	1,026.07
Speech Crispness (Zero Crossing Rate)	85	0.06	0.01	0.04	0.08
Speech Brightness (Hz)	85	2,707.58	295.75	1,926.01	3,531.48
Frequency Spread (Hz)	85	3,261.80	280.81	2,738.14	4,044.18
Speech Clarity (dB)	85	17.53	0.53	16.25	18.54
Study 2a					
Speech Rate (Beats Per Minute)	89	121.93	13.91	93.75	156.61
Loudness Variability (s.d. of waveform aplitudes)	89	0.08	0.04	0.02	0.17
Pitch Variation (Hz)	89	919.35	69.34	718.03	1,073.71
Speech Crispness (Zero Crossing Rate)	89	0.07	0.03	0.04	0.21
Speech Brightness (Hz)	89	2,674.69	639.57	1,780.84	5,789.12
Frequency Spread (Hz)	89	3,215.17	626.22	1,754.07	5,405.72
Speech Clarity (dB)	89	18.47	0.66	16.84	20.18
Study 2b					
Speech Rate (Beats Per Minute)	98	126.36	12.25	98.45	159.35
Loudness Variability (s.d. of waveform aplitudes)	98	0.05	0.01	0.03	0.06
Pitch Variation (Hz)	98	889.62	59.96	699.67	1,007.95
Speech Crispness (Zero Crossing Rate)	98	0.06	0.01	0.05	0.08
Speech Brightness (Hz)	98	2,797.36	280.82	2,190.81	3,327.01
Frequency Spread (Hz)	98	3,301.66	243.44	2,716.18	3,850.54
Speech Clarity (dB)	98	17.85	0.53	16.76	19.19
Study 2c					
Speech Rate (Beats Per Minute)	97	126.09	10.20	98.68	156.25
Loudness Variability (s.d. of waveform aplitudes)	97	0.09	0.02	0.03	0.13
Pitch Variation (Hz)	97	1,007.81	69.44	796.98	1,134.34
Speech Crispness (Zero Crossing Rate)	97	0.10	0.03	0.05	0.17
Speech Brightness (Hz)	97	2,367.15	685.65	1,316.87	3,783.69
Frequency Spread (Hz)	97	2,342.89	791.87	1,313.49	4,006.06
Speech Clarity (dB)	97	19.93	1.06	16.91	22.07

Supplementary Table S18. Correlations between the auditory features of spoken conversations across Studies 1 and 2. Values report Pearson correlation coefficients with two-sided tests. No adjustments were made for multiple comparisons.

Correlation Matrix of Audio Features

Feature	Speech Rate	Loudness Variability	Pitch Variation	Speech Crispness	Speech Brightness	Frequency Spread	Speech Clarity
Speech Rate							
Loudness Variability	-0.08						
Pitch Variation	0	0.37***					
Speech Crispness	-0.03	0.1	0.41***				
Speech Brightness	-0.01	-0.26***	0.08	-0.24***			
Frequency Spread	-0.01	-0.37***	-0.24***	-0.59***	0.89***		
Speech Clarity	-0.04	0.75***	0.58***	0.31***	-0.12*	-0.4***	

Note: * $p < .05$, ** $p < .01$, ***$p < .001$

Supplementary Table S19. Regression showing the main effects of the extracted auditory features in spoken conversations on survey dependent variables across Studies 1, 2A and 2C. All models are estimated using ordinary least squares linear regression at the dyad level, with topic and study fixed effects included. Statistical tests are two-sided. Standard errors are clustered at the dyad level. No adjustments were made for multiple comparisons. Results show that speech rate is most strongly associated with constructive disagreement outcomes, although the associations were only weakly significant.

	Perceived Understanding	Perceived Conflict	Perceived Competence	Perceived Humanization	Attitude Shift	Liking	Enjoyment
	(1)	(2)	(3)	(4)	(5)	(6)	(7)
Speech Rate	0.010*	-0.010*	0.009*	0.008	0.002	0.004	0.009*
	(0.005)	(0.004)	(0.004)	(0.005)	(0.002)	(0.003)	(0.003)
Loudness Variation	0.342	-0.554	1.604	2.676	-5.062	0.590	7.310*
	(2.407)	(3.762)	(2.96)	(2.833)	(2.642)	(2.484)	(3.196)
Pitch Variation	-0.002	0.000	0.001	-0.001	0.000	0.000	-0.001
	(0.001)	(0.001)	(0.001)	(0.001)	(0.001)	(0.001)	(0.001)
Speech Crispness	1.694	-2.454	6.713	9.950*	-6.722	3.391	10.964*
	(6.907)	(5.903)	(5.018)	(4.347)	(4.363)	(5.131)	(5.247)
Speech Brightness	0.000	0.000	-0.001	0.000	0.001	0.000	0.000
	(0.001)	(0.001)	(0.001)	(0.001)	(0.000)	(0.001)	(0.001)
Frequency Spread	0.000	-0.001	0.000	0.000	0.000	0.000	0.000
	(0.001)	(0.001)	(0.001)	(0.001)	(0.000)	(0.001)	(0.001)
Speech Clarity	0.074	-0.010	-0.115	-0.141	0.097	-0.089	-0.023
	(0.101)	(0.097)	(0.106)	(0.118)	(0.084)	(0.101)	(0.095)
Topic F/E	YES	YES	YES	YES	YES	YES	YES
Experiment F/E	YES	YES	YES	YES	YES	YES	YES
Degrees of Freedom	350	350	350	350	350	350	350
R^2	0.075	0.049	0.105	0.060	0.667	0.386	0.293
Adjusted R^2	0.028	0.000	0.058	0.012	0.650	0.350	0.257

Note: Parentheses show standard errors
*p<0.05; **p<0.01; ***p<0.001

Supplementary Note 15 - Attrition Across Studies 1-3

Supplementary Table S20 outlines the key stages of participant involvement for each data collection sample. In Studies 1, 2a, 2b, and 2c, some participants were dismissed from the study (or, in rare cases, voluntarily exited) before starting their conversations; others were excluded from analysis for not meeting the inclusion criteria. There were two inclusion criteria which we applied consistently across these studies. First, we required each participant to feel relatively strong about their assigned topic of conversation, such that they reported either a 1 (“somewhat strongly”) or 2 (“very strongly”) on the question measuring attitude strength (“How strongly do you feel about your stance?” with the participant selecting one of three Likert scale response options: 0 “not strongly at all”, 1 “somewhat strongly”, or 2 “very strongly”). Second, we required each pair to be composed of individuals with opposing positions on their assigned topic of conversation. Specifically, each topic stance question contained six Likert scale response options, with participants selecting one of the six options: -3, -2, -1, 1, 2 or 3, with -3 labeled as “completely oppose” and 3 labeled as “completely support.” To satisfy the second criterion, partners had to be at least three Likert scale points apart on their topic stance question. Dyads who did not satisfy these criteria - for instance, because one person did not feel strongly enough about the assigned topic,or because they did not disagree enough with each other - were not included in the analysis. The only exception to this was in Study 3, where participants engaged in multiple conversations with multiple partners on the same topic. There, we did not exclude dyads with insufficient disagreement, but instead we analysed them separately.

In general, attrition was around or less than 10% and tended to be largely due to pre-treatment factors. However, an exception was in Study 1, because we used a different matching procedure to determine pairs (based on answers to a unique survey item regarding

the issue at the participant's school); we ended up excluding several pairs from analysis who did not satisfy our later inclusion criteria.

Supplementary Table S20. Study sample at different stages, from recruitment to study completion, and data availability in Studies 1, 2, and 3. Attrition from one stage of each study to the next is reported. Study 3 refers separately to data collected from three different participant sites: University of California, Berkeley ("UC Berkeley"), Minnesota State University, Mankato ("MNSU"), and Arizona State University ("ASU").

**Study**		**Experimental Condition Assignment**				**Total**	**% Attrition**	**Reason for Attrition**
**Study 1**		**Video-Chat**	**Speaking**	**Writing**				
	Total Individuals Who Started Survey					421 people		
	Participants Who Started Conversation	144	124	128		396 people	94.1% (396 of 421)	**Pre-pairing attrition.** Could not participate because: unable to be matched with a partner (odd # of participants in a session), not fluent in English, or voluntarily left prior to the conversation (personal reasons).
	Pairs Who Satisfy Criteria for Analysis	45	54	47		146 pairs (292 people)	73.7% (292 of 396)	**Pair-level exclusions.** Excluded from analysis due to lack of adequately strong disagreement with partner.
	Pairs Who Finished Conversation & Post-Survey	45	54	47		146 pairs (292 people)	100% (292 of 292)	**Post-treatment attrition**. Left after starting the conversation or had technical difficulties with their equipment (e.g., Skype) that prevented completing the conversation.
	Recorded Conversations Included in Language Analysis	40	46	47		133 pairs (266 people)	91.1% (266 of 292)	**Recording issues.** Technical issues led to recording not stored or recording could not be analyzed.
**Study 2A**		**Speaking Multiple Exchanges**	**Writing Multiple Exchanges**	**Speaking Single Exchange**	**Writing Single Exchange**			
	Total Individuals Who Started Survey					435 people		
	Participants Who Started Conversation	118	126	88	102	434 people	99.8% (434 of 435)	**Pre-pairing attrition.** Could not participate because: unable to be matched with a partner (odd # of participants in a session), not fluent in English, or voluntarily left prior to the conversation (personal reasons).
	Pairs Who Satisfy Criteria for Analysis	54	49	39	45	187 pairs (374 people)	86.2% (374 of 434)	**Pair-level exclusions.** Excluded from analysis due to

								a lack of adequately strong disagreement with the partner.
	Pairs Who Finished Conversation & Post-Survey	53	48	39	45	185 pairs (370 people)	98.9% (370 of 374)	**Post-treatment attrition**. Left after starting the conversation or had technical difficulties with their equipment (e.g., Skype) that prevented completing the conversation.
	Recorded Conversations Included in Language Analysis	51	48	38	44	181 pairs (362 people)	97.8% (362 of 370)	**Recording issues.** Technical issues led to recording not stored or recording could not be analyzed.
**Study 2B**		**Speaking Long**	**Writing Long**	**Speaking Short**	**Writing Short**			
	Total Individuals Who Started Survey					482 people		
	Participants Who Started Conversation	112	114	118	114	458 people	95.0% (458 of 482)	**Pre-pairing attrition.** Could not participate because: unable to be matched with a partner (odd # of participants in a session), not fluent in English, or voluntarily left prior to conversation (personal reasons).
	Pairs Who Satisfy Criteria forAnalysis	51	51	52	51	205 pairs (410 people)	89.5% (410 of 458)	**Pair-level exclusions.** Excluded from analysis due to lack of adequately strong disagreement with the partner.
	Pairs Who Finished Conversation & Post-Survey	51	51	52	51	205 pairs (410 people)	100% (410 of 410)	**Post-treatment attrition**. Left after starting the conversation or had technical difficulties with their equipment (e.g., Skype) that prevented completing the conversation.
	Recorded Conversations Included in Language Analysis	50	50	48	50	198 pairs (396 people)	96.6% (396 of 410)	**Recording issues.** Technical issues led to recording not stored or recording could not be analyzed.
**Study 2C**		**Speaking Asychronous**	**Writing Asynchronous**	**Speaking Synchronous**	**Writing Synchronous**			
	Total Individuals Who Started Survey					489 people		
	Participants Who Started Conversation	128	128	114	102	472 people	96.5% (472 of 489)	**Pre-pairing attrition.** Could not participate because: unable to be matched with a partner (odd # of participants in a session), not fluent in English, or voluntarily left prior to the conversation (personal reasons).
	Pairs Who Satisfy Criteria for Analysis	54	56	50	47	207 pairs (414 people)	87.7% (414 of 472)	**Pair-level exclusions.** Excluded from analysis due to lack of adequately strong disagreement with partner.
	Pairs Who Finished Conversation & Post-Survey	54	56	50	47	207 pairs (414 people)	100% (414 of 414)	**Post-treatment attrition**. Left after starting the conversation or had technical difficulties with their equipment (e.g., Skype) that prevented completing the conversation.

	Recorded Conversations Included in Language Analysis	51	45	46	45	187 pairs (374 people)	90.3% (374 of 414)	**Recording issues.** Technical issues led to recording not stored or recording could not be analyzed.
**Study 3 - UC Berkeley Sample**		**Speaking**		**Writing**				
	Number of Participants Who Had at Least 1 Conversation	21.2*		16.8*		38 people		
	Participants Who Did a Pre-survey	15		16		31 people	81.6% (31 of 38)	**Pre-survey attrition.** Participants who did not complete a pre-survey due to late arrival.
	Participants Who Did a Pre-Survey and at Least 1 Post-Survey	11		14		25 people	80.6% (25 of 31)	**Post-survey attrition**. Participants who left before completing a post-survey or forgot to complete a post-survey.
	Conversations with at Least 1 Post-Survey	36		39		75 conversations (136 post-surveys)		(61 conversations had 2 post-surveys each, 14 conversations had 1 post-survey each.)
	Conversations Where Both Partners Did Post-survey	23		38		61 conversations (122 post-surveys)	81.3% (61 of 75)	Each conversation has 2 post-surveys, one from each partner.
	Conversations Where Both Partners Did Post-Survey and Pre-Survey	12		29		41 conversations (82 post-surveys)	67.2% (41 of 61)	Dataset without any incomplete data. All conversations include pre-survey and post-survey from both parties.
**Study 3 - MNSU Sample**		**Speaking**		**Writing**				
	Number of Participants Who Had at Least 1 Conversation	16		16		32 people		
	Participants Who Did a Pre-survey	16		16		32 people	100% (32 of 32)	**Pre-survey attrition.** Participants who did not complete a pre-survey due to late arrival.
	Participants Who Did a Pre-Survey and at Least 1 Post-Survey	16		16		32 people	100% (32 of 32)	**Post-survey attrition**. Participants who left before completing a post-survey or forgot to complete a post-survey.
	Conversations with at Least 1 Post-Survey	38		38		76 conversations (147 post-surveys)		(71 conversations had 2 post-surveys each, 5 conversations had 1 post-survey each.)
	Conversations Where Both Partners Did Post-survey	34		37		71 conversations (142 post-surveys)	93.4% (71 of 76)	Each conversation has 2 post-surveys, one from each partner.

	Conversations Where Both Partners Did Post-Survey and Pre-Survey	34	37	71 conversations (142 post-surveys)	100% (71 of 71)	Dataset without any incomplete data. All conversations include pre-survey and post-survey from both parties.
**Study 3 - ASU Sample**		**Speaking**	**Writing**			
	Number of Participants Who Had at Least 1 Conversation	13	21	34 people		
	Participants Who Did a Pre-survey	12	21	33 people	97.1% (33 of 34)	**Pre-survey attrition.** Participants who did not complete a pre-survey due to late arrival.
	Participants Who Did a Pre-Survey and at Least 1 Post-Survey	12	21	33 people	100% (33 of 33)	**Post-survey attrition**. Participants who left before completing a post-survey or forgot to complete a post-survey.
	Conversations with at Least 1 Post-Survey	33	38	71 conversations (142 post-surveys)		(71 conversations had 2 post-surveys each.)
	Conversations Where Both Partners Did Post-survey	33	38	71 conversations (142 post-surveys)	100% (71 of 71)	Each conversation has 2 post-surveys, one from each partner.
	Conversations Where Both Partners Did Post-Survey and Pre-Survey	29	38	67 conversations (134 post-surveys)	94.4% (67 of 71)	Dataset without any incomplete data. All conversations include pre-survey and post-survey from both parties.

*Notes. Because all participants in Study 3 had multiple conversations, we report not only participant-level attrition but also conversation-level (and post-survey level) attrition. In the Study 3 UC Berkeley sample, one participant had five conversations (1 spoken, 4 written) which is why we report them as being 0.2 in the speaking condition and 0.8 in the writing condition. No other participants changed experimental conditions to our knowledge, and this participant did not complete a pre-survey so they are not included in the main analyses.

Supplementary Information P - Topic Descriptions Used in Studies 1-3

In Studies 1 and 2A, participants were asked to discuss one of three possible topics: potential changes to the drinking age, use of genetically modified food, and implementing an admissions quota. Supplementary Table S21 shows the three topics selected from a broader set of 13 topics that were tested in a pilot survey (Participants = 191; 71.2% Berkeley affiliation; 65.4% Female; 34.6% Male; 5.24% Conservative; 72.8% Liberal; 35.1% College degree) collecting opinions from students on campus on each topic. For the full pilot survey and data, see OSF. Each topic was rated on a scale from 1 (Strongly Disagree) to 7 (Strongly Agree). We selected the three topics to use in the main study because they had relatively high variances in opinions and bimodal agreement scores, increasing the chance that we could pair dyads to strongly disagree with each other about at least one topic.

Supplementary Table S21. Mean and standard deviation scores for agreement on topics in the pilot study to select conversation topics for Studies 1 and 2A. Selected topics are shown in bold. Topics are ordered from highest standard deviation to lowest.

**Topic**	**Mean Agreement Score (1-7)**	**Standard Deviation of Agreement Score**
Sanders V Clinton	3.10	1.81
**Drinking age**	**3.59**	**1.78**
Vegetarian cafe	2.81	1.62
**Admissions quota**	**2.84**	**1.57**
**Use of GMOs**	**3.79**	**1.56**
Animal testing	3.76	1.54
Oakland gentrification	3.32	1.49
Hillary Clinton	4.12	1.38
Greek life	3.48	1.37
Increased tuition	2.24	1.34
Standardized testing	3.26	1.29

Euthenasia	4.92	1.13
Reduce homelessness	5.06	1.06

Note. We intended to select the topics with the greatest variance in agreement, but deemed the Sanders vs. Clinton and vegetarian cafe topics to be less relevant to our sample and thus chose to exclude them from use.

In Studies 2B and 2C, participants were asked to discuss three additional topics - allowing controversial speakers on campus, making slavery reparations, and legalizing drugs. Below shows the three topics selected from a pilot survey (N = 101; 90.1% Berkeley affiliation; 78.2% Female; 20.8% Male; 6.93% Conservative; 68.3% Liberal; 28.7% College degree) that collected opinions from students on campus about 14 topics in total. For the full pilot survey and data, see OSF. We selected the three topics to use in the main study because they had high variances in opinions and bimodal agreement scores, increasing the chance that we could pair dyads to strongly disagree with each other about at least one topic.

Supplementary Table S22. Mean and standard deviation scores for agreement on topics in the pilot study to select conversation topics for Studies 2B and 2C. Selected topics are shown in bold. Topics are ordered from highest standard deviation to lowest.

**Topic**	**Mean Agreement Score (1-7)**	**Standard Deviation of Agreement Score**
**Legalising all drugs**	**3.25**	**1.62**
**Controversial speakers on campus**	**3.72**	**1.59**
**Slavery reparations**	**3.48**	**1.53**
Women register selective service	3.47	1.52
Adverts using retouched photos	4.62	1.49
Athletes get priority class enrollment	3.03	1.49
Greek life at Berkeley	3.54	1.47
Universal basic income	3.73	1.46
Animal testing	4.13	1.44
Legalising cognitive drugs	2.89	1.43
Standardized test	3.5	1.4

Gentrification of Oakland	3.25	1.28
Military UAVs	3.46	1.27
Right to die law	4.8	1.09

In Study 3, we consulted with the local Bridge organizers at each location to choose a single topic for discussion, based on what they believed their students would find controversial. Those topics are reported below. For each topic, we strove to provide a relatively objective summary of each side of the debate to enrich participants' conversations.

The text that participants read regarding each of the topics from Studies 1, 2 and 3 are below.

<u>Topics used in Studies 1 and 2a</u>

Drinking Age Topic: "There has been a recent push for the legal drinking age to be lowered from 21 to 17. Proponents of lowering the legal drinking age to 17 argue that the even younger age of 16 is when many Europeans are considered old enough to drink beer and wine. They believe 17 year olds are old enough to have the autonomy to choose whether or not they would like to consume alcohol. Opponents argue that lowering the drinking age would be detrimental because at 17, the brain is not fully developed, nor is decision-making ability. Therefore, 17 year olds may be more likely to engage in dangerous activities as a result of drinking, such as drunk driving. In addition, because the brain is not fully developed until 25, they believe drinking at 17 could inhibit proper brain development and lead to health issues later on."

- "Do you oppose or support changing the legal drinking age from 21 to 17?" (-3 = completely oppose, -2, -1, 1, 2, 3 = completely support)
- "For example, imagine that UC Berkeley could change the legal drinking age from 21 to 17. (Currently the legal drinking age is 21). Do you think that UC Berkeley should

change the drinking age to 17 years old on campus?” (yes / no)

- “How strongly do you feel about your stance?” (0 = not strongly at all, 1 = somewhat strongly, 2 = very strongly)

GMO Topic: “GMOs, or genetically modified organisms, have been frequently debated throughout the years, particularly in regard to genetically modified food. Proponents of GMOs argue that genetic engineering might help in feeding the growing population. Opponents argue that almost no research has been conducted on the health consequences of genetic engineering and there is plenty of reason to believe that genetically modifying food can result in devastating health consequences, from severe allergies to cancer.”

- “Do you oppose or support genetically modified food?” (-3 = completely oppose, -2, -1, 1, 2, 3 = completely support)
- “For example, imagine that UC Berkeley is deciding whether or not to use GMO-produce in their cafeterias around campus. (Imagine that currently they do NOT carry any GMO-produce). They could decide to start using some GMO-produce, which will save them some money, or to continue using no GMO-produce. Do you think UC Berkeley should switch to using some GMO-produce in their cafeterias?” (yes / no)
- “How strongly do you feel about your stance?” (0 = not strongly at all, 1 = somewhat strongly, 2 = very strongly)

Race Quota Topic: “According to UC Berkeley, this is the breakdown of the undergraduate student body, by race, in 2013:

https://diversity.berkeley.edu/sites/default/files/diversity-snapshot-web-final.pdf

Some have argued for a racial quota for admissions in order to promote diversity. Proponents of a racial quota argue that this practice would increase diversity and advance populations that have been historically underrepresented in higher education. Opponents argue that this practice could be a form of discrimination in and of itself, as it reduces reliance on merit and limits possibilities for those in racial populations who have historically been well represented in higher education."

- "Do you oppose or support a racial quota for admissions at UC Berkeley?" (-3 = completely oppose, -2, -1, 1, 2, 3 = completely support)
- "Specifically, imagine that UC Berkeley is deciding whether or not to use racial quotas in their admission systems. (Imagine that currently there are no racial quotas). They could decide to use no quotas at all or implement some racial quotas in their decisions. Should UC Berkeley switch to making some racial quota-driven decisions for their admissions process?" (yes / no)
- "How strongly do you feel about your stance?" (0 = not strongly at all, 1 = somewhat strongly, 2 = very strongly)

Topics used in Studies 2b and 2c

Controversial Speaker Topic: "In 2017, UC Berkeley expected multiple visits by controversial speakers such as Milo Yiannopoulos and Ann Coulter. These planned visits resulted in protests and widespread debate over who should be allowed to speak on campus. **Proponents** of allowing extremely controversial speakers to speak on campus say that everyone has a right to free speech, regardless of how controversial the viewpoint. **Opponents** of allowing extremely controversial speakers to speak on campus say that some viewpoints should be considered hate speech, which can encourage violence and cause fear

and psychological damage."

- "Do you oppose or support allowing extremely controversial speakers to speak on campus?" (-3 = completely oppose, -2, -1, 1, 2, 3 = completely support)
- "How strongly do you feel about your stance?" (0 = not strongly at all, 1 = somewhat strongly, 2 = very strongly)

Reparations Topic: "There are significant discrepancies between average infant-mortality rates, life expectancy, unemployment rates, and income between Black American and White American populations in the United States. Some have suggested that these discrepancies are remnants of slavery (the "legacy of slavery"), and the government has considered making reparations, or providing some sort of compensation (e.g., financial or structural) to descendants of slaves. **Proponents** of requiring government reparations for slavery say that, while slavery may have been abolished a long time ago, the effects of slavery and racism still exist and the government has a moral obligation to try to repair the continuing effects of slavery. **Opponents** of requiring government reparations for slavery say that financial reparations are expensive, and many Americans would have to pay more in taxes, despite having nothing to do with slavery themselves. They argue that the reparations also wouldn't solve a lot of the issues facing Black Americans today."

- "Do you oppose or support requiring government reparations for slavery?" (-3 = completely oppose, -2, -1, 1, 2, 3 = completely support)
- "How strongly do you feel about your stance?" (0 = not strongly at all, 1 = somewhat strongly, 2 = very strongly)

Drug Legalization Topic: "In California, there are legal drugs from each of the 5 drug

classes (e.g., alcohol, marijuana, nicotine). But many other drugs from these classes are still illegal (e.g., cocaine, heroin, LSD). Some people have suggested that all drugs should be decriminalized so that, instead of facing jail-time, drug users would be instead sent to medical professionals for treatment. For example, Portugal legalized drugs in 2001 and since then, Portugal's drug-related HIV infections and overdose death rate have declined. **Proponents** of legalizing all drugs in the U.S. say that illegal drugs are problematic because it promotes trafficking, violence, and drug abuse. If someone overdoses, others don't call for help for fear of getting in trouble. They also argue that a lot of government money is spent on law enforcement and incarceration when it could be spent on social programs to prevent drug use and rehabilitation. **Opponents** of legalizing all drugs in the U.S. say that legalization will increase levels of drug abuse and addiction. They argue that, while the current laws may not be entirely effective in eliminating issues from illegal drug use, these issues could be much worse if drugs were legalized."

- "Do you oppose or support legalizing drugs in the U.S.?" (-3 = completely oppose, -2, -1, 1, 2, 3 = completely support)
- "How strongly do you feel about your stance?" (0 = not strongly at all, 1 = somewhat strongly, 2 = very strongly)

Topics used in Study 3 (varied by each participant location)

Participants in Study 3 received one topic to discuss for each location. Participants at University of California, Berkeley ("UC Berekeley") discussed same sex marriage, participants at Minnesota State University, Mankato ("MNSU") discussed gun control laws, and participants at Arizona State University ("ASU") discussed the US-Mexico wall.

Same-Sex Marriage Topic (UC Berkeley): "In June 2018, the Supreme Court ruled that Christian baker Jack Phillips cannot be forced to bake a cake for a same-sex wedding.

Supporters of this ruling have stated that private business owners like Philips should have the right to refuse to provide service for any clients that he does not want to serve. Opponents of the ruling have stated that refusing to serve anyone on the basis of their membership in a social group (e.g., sexual orientation, race, religion, or gender) constitutes discrimination."

- To what extent do you oppose or support private business owners having the right to refuse service for the clients on the basis of their social membership (e.g., sexual orientation, race, religion, gender)? (-3 = completely oppose, -2, -1, 1, 2, 3 = completely support)
- How strongly do you feel about your stance? (0 = not strongly at all, 1 = somewhat strongly, 2 = very strongly)
- How much do you feel like you know about this topic? (1 = not much at all, 5 = a great deal)

Gun Control Topic (MNSU): "The United States has the highest total and per capita number of guns in the world. The United States also has more mass shootings than any other country, prompting widespread debate over whether or not the United States should adopt stronger gun control laws. Proponents of stronger gun control laws state that the Second Amendment was intended for militias; that gun violence would be reduced; that gun restrictions have always existed; and that a majority of Americans, including gun owners, support new gun restrictions. They state that the government can reduce shooting deaths by imposing common sense restrictions on gun access that do not violate the Second Amendment. Opponents of stronger gun control laws state that the Second Amendment protects an individual's right to own guns; that guns are needed for self-defense from threats ranging from local criminals to foreign invaders; and that gun ownership deters crime rather than causes more crime. They

state that placing strict regulations on gun ownership will only benefit criminals by disarming those who could otherwise defend themselves and save others.”

- To what extent do you oppose or support adopting stronger gun control laws in the United States? (-3 = completely oppose, -2, -1, 1, 2, 3 = completely support)
- How strongly do you feel about your stance? (0 = not strongly at all, 1 = somewhat strongly, 2 = very strongly)
- How much do you feel like you know about this topic? (1 = not much at all, 5 = a great deal)

US-Mexico Border Topic (ASU): “Proponents of expanding the wall state that expanding the wall is a good investment, as it will decrease the number of illegal immigrants. They believe the wall will help protect and strengthen America. For example, it may reduce crime and reduce illegal immigrants being hired for American jobs. They believe a country is only as strong as its borders. Opponents of expanding the wall state that expanding the wall may not significantly reduce the number of illegal immigrants crossing the border (for example, because there are other routes into the U.S., such as tunnels, boat smuggling, and truck smuggling). They suggest that the wall is unlikely to fix problems (like reducing crime and reducing illegal immigrants being hired for American jobs). Some also believe that building the wall may be primarily lucrative for large contracting companies.”

- To what extent do you personally oppose or support substantially expanding the U.S.-Mexico border wall? (-3 = completely oppose, -2, -1, 1, 2, 3 = completely support)
- How strongly do you feel about your stance? (0 = not strongly at all, 1 = somewhat strongly, 2 = very strongly)

- How much do you feel like you know about this topic? (1 = not much at all, 5 = a great deal)

Supplementary Information Q - Protocol Details for Studies 1-3

Below, we provide each experimental script in its original form with only slight modifications for readability. These scripts were written by an experimenter and used when training research assistants on how to conduct the studies. Typically, italicized text in the scripts reflects instructions for research assistants (e.g., regarding the procedure they should follow) whereas non-italicized text reflects words that research assistants would read aloud to participants. Each research assistant was given a hard copy of the script and told to follow it exactly when they ran participants in the study. The scripts contain several abbreviations, terms, and acronyms that would be familiar to our research assistants, including: SONA (the system hosting our participant pools), RA (research assistant), Xlab (the Experimental Social Science laboratory at UC Berkeley), and Qualtrics (the platform on which we design and conduct surveys). We relabeled any experimental conditions to match the final labels used in the paper.

Study 1 Script

Participants will be recruited from SONA system. They should be fluent English speakers. XLab employees (paid version) or RA (credit version) will check them in, give participant number nametags, and assign them computers. Make sure you have the name tags filled out with participant numbers before the session.

As participants are walking in, speaking RA: Welcome! When you sit down, please don't start the survey on the screen until I give you further instructions.

(Once everyone is seated)

Hello! Welcome to the Political Topics Study! Before we start, is anyone here not fluent in English? *(If they are not fluent English speakers, they cannot participate and should not be paid a show up fee)*

Great! We'll start now, but before we do, please put away your cell phones and give me your full attention. During this study, it is very important that you do not do anything on your computers until you are told to - please do not close any of the windows unless you are told.

Today, you are going to participate in a study about people's opinions about different topics. The study is divided into 3 parts. In Part 1, you'll read about a few topics and give your opinions on them. In Part 2, we will match you with another participant and assign you to one of the topics from the first part of the study and you will engage with a partner in a conversation about your assigned topic. We will be recording these conversations. Finally, in Part 3, you will take a survey about your experiences.

Now, please review and sign the consent forms [audio/video conditions: and media release forms] on your desk - let me know if you have any questions. [audio/video conditions: You can only be in the study if you initial all of the boxes in the media release form]. Raise your hand when you are done with the forms, and I will collect them. If you wish to keep a copy of the consent form, let me know at the end of the study.

Collect all forms. Make sure the consent forms are signed and the media release forms are signed and initialed - must have initials for each row to participate.

Okay, you may now start the survey on your computer. You will put your assigned participant number-- **the number we put on your nametag - NOT the computer number** -- into the first question. **Please read the questions carefully, as you will be assigned to discuss your answer to one of the topics with a partner.** After you finish the final question on the survey, please hit the final arrow button to make sure your response is submitted and raise your hand so I know you are all done.

(Speaking RA should walk around making sure everyone is completing the survey)

Awesome, it looks like everyone is done. Before we move on, I want to make sure everyone hit "next" to complete their survey. Now, minimize the screen. You should now be looking at the desktop with desktop icons on the left side. Is everyone there? *(Speaking RA walk around and make sure they are all at the same place)*

(The RA in charge of matching should refresh Qualtrics response page and make sure everyone's response shows up. After confirmed, Speaking RA:)

Now, we need to quickly review the answers you have given and match you with a partner. While you wait, we have another survey for you to take. Please click on the bookmark on

your computer titled “EXP 2” and work on that while we match you. Please press the arrow button once you’ve reached the end to ensure your response is recorded.

(Speaking RA should walk around and make sure that everyone is completing the correct survey while matching RA matches the pairs).

(While matching RA is pairing, speaking RA, after they finish making sure everyone is on the right survey, should keep checking in on the most recent pair made, and go write the pairs as they are made in the chart on the whiteboard. While writing the pairs, speaking RA should also check if there are mistakes, e.g. one participant number appears twice)

NOTE: Once the pairs are all up on the board and double checked that no duplicates exist/ all numbers included, if the participants are still on the personality survey, just say “Okay, please take the next 15 seconds to finish up your survey. If you haven’t completed your last question, that is okay. Just put “N/A” and hit next until you’re finished. If a participant was not matched, they should receive the $5 from XLab staff (or take down their name and tell them you will allocate 0.5 credits later).”

Now it’s time to start the second part of the study. As you can see, we’ve placed all of your numbers here on the board. First, note where you are located on the board. Does anyone not see their number?

Now, since the conversation with your partner will take place over Skype, you will now switch computers where you will be logged into your Skype account. Please take note of your new computer number. It is written directly to the right of your participant number on the whiteboard. The computers all have labels, starting with X01 right here. Now, please collect your things, including your notesheet, and go to the computer you’ve been assigned to.

(Help participants find computers. Once the participants have settled)

Okay, the topic in the rightmost box of your row *(point to show)* is the topic you will be debating with your partner. **As a reminder, here is a copy of the specific question that you will be debating.** ***Matching RA: Hand out <u>question prompts</u>, according to assigned topic. Then, hand out instruction sheets.***

You and your partner have been matched because you have different opinions on this topic. Your job today is to get your partner to understand your position and why you hold it. Think of this conversation as a debate. This debate will be held in a structured manner. *Point to whiteboard debate rules.* You will start by delivering 3-minute statements to each other. If you are at computers 1-10 *(gesture towards participants)* you will be delivering your

3-minute statement first, and I will refer to you as Person A. If you are at computers 11-20 *(gesture towards participants)* you will be delivering your 3-minute statement second, and I will refer to you as Person B. Then, you will have 3 minutes to discuss the topic in a back and forth manner. You have also been given some instruction sheets you can refer to if you forget the conversation instructions or are confused.

3 minutes can feel like a long time to deliver a statement, but we do expect you to take the full three minutes. So you have a chance to prepare, there is a note sheet on your desk. Please take the next two minutes to collect your thoughts about what you want to say in your 3-minute statement, using your note sheet as needed. Go ahead and start now, and I'll let you know when time is up.

(After 2 minutes)

Your time is up. Now open up Google Chrome from the taskbar at the bottom of the computer screen. When you open it, you should already be logged into a Skype account. Do not contact your partner yet.

We will be recording these conversations, so we will quickly walk around to start the screen recordings. *(RAs should walk around and quickly click Start Recording on all computers).*

WRITING CONDITION:

> **Since we are recording the screen, please make sure your cursor doesn't block any of the text - move it off to the side of the screen whenever possible.** Great, now, Person A, you will have three minutes to type your first statement to your partner. I will tell you when your time is up. Person B, please sit and wait quietly until your partner sends you their statement, and ***do not*** **start typing until Person A's three minutes are up**. As a reminder, please only discuss your assigned topic. Person A, go ahead. *(15 second warning, After 3 minutes have passed)* Please finish your statement and send it to your partner. *(Make sure everyone sends the statements)* Person B, take a minute to read the statement and let me know when you are done reading by raising your hand *(After about one minute)* Okay, now, Person B, you have three minutes to type your statement to your partner. I will tell you when your time is up. Person A, please sit and wait until your partner sends you their statement. Person B, go ahead. *(15 second warning, After 3 minutes have passed)* Please finish your statement and send it to your partner.

(Once everyone has sent the statements) Person A, you now have a minute to read the statement - raise your hand when you are done reading. *(After about one minute)* Okay, now you both have 3 minutes to have a back-and-forth debate about your topic. You do not have to come to an agreement. I will tell you when your time is up. *(After 3 minutes)* Please finish your conversation.

SPEAKING CONDITION:

Do not put your headphones on yet, but Person A, go ahead and click on the phone call button to contact your partner - **do not click the video call button**. Person B, accept the call by clicking the phone call button when you receive it - **do not click the video call button**. If you cannot contact your partner, raise your hand and I will help you. *(Both RAs must walk around to make sure each pair has gotten in contact with their partner. **Make sure video cameras are off)***

PLEASE MOVE YOUR CURSOR OFF TO THE SIDE OF THE SCREEN. Great, now, Person A, you will have three minutes to deliver your first statement to your partner. I will tell you when your time is up. Try to talk for the full 3 minutes -- if you run out of things to say, please just sit quietly and wait for Person B's turn. **Person B, please do not start talking until after the three minutes are up and I tell you that you can start.** As a reminder, please only talk about your assigned topic. To test the sound, go ahead and put on your headphones and just say hello - do not start making your statement yet. *Make sure everyone can hear each other.* Person A, go ahead and start your 3-minute statement. *(After 3 minutes have passed)* Please finish up your statement. *(Once everyone has finished the statements)* Okay, now, Person B, you have three minutes to deliver your statement to your partner. I will tell you when your time is up. Go ahead. *(After 3 minutes have passed)* Please finish your statement.

(Once everyone has sent the statements) Okay, now you both have 3 minutes to have a back-and-forth debate about your topic. You do not have to come to an agreement. I will tell you when your time is up. *(After 3 minutes)* Please finish your conversation.

VIDEO-CHATTING CONDITION:

During the conversation, please make sure your face stays in the video so your partner can see you - do not move off to the side of the screen. Do not put your

headphones on yet, but Person A, go ahead and click on the video call button to contact your partner. Person B, accept the call when you receive it by clicking the video call button. If you cannot contact your partner, raise your hand and I will help you. *(Both RAs must walk around to make sure each pair has gotten in contact with their partner.* ***Make sure video cameras are on)***

PLEASE MOVE YOUR CURSOR OFF TO THE SIDE OF THE SCREEN. Great, now, Person A, you will have three minutes to deliver your first statement to your partner. I will tell you when your time is up. Try to talk for the full 3 minutes -- if you run out of things to say, please just sit quietly and wait for Person B's turn. **Person B, please do not start talking until after the three minutes are up and I tell you that you can start.** As a reminder, please only talk about your assigned topic. To test the sound, go ahead and put on your headphones and just say hello - do not start making your statement yet. *Make sure everyone can hear each other.* Person A, go ahead and start your 3-minute statement. *(After 3 minutes have passed)* Please finish up your statement. *(Once everyone has finished the statements)* Okay, now, Person B, you have three minutes to deliver your statement to your partner. I will tell you when your time is up. Go ahead. *(After 3 minutes have passed)* Please finish your statement.

(Once everyone has sent the statements) Okay, now you both have 3 minutes to have a back-and-forth debate about your topic. You do not have to come to an agreement. I will tell you when your time is up. *(After 3 minutes)* Please finish your conversation.

We are now just going to end the screen recordings before moving on. *(RAs should walk around and quickly press on the keyboard shortcut 'F9' on all computers.)*

Finally, minimize the windows until you get to the desktop, and please click on the desktop shortcut titled "EXP 3." Again, you will use the participant number on your name tag to fill out the first question. When you are finished, make sure to hit the arrow button to finish. When you are done, please raise your hand. **Please do not exit out of the screen before I**

have checked for completion. If you'd like to be debriefed, there are sheets at the table in the back.

Study 2A Script

Participants will be recruited from the SONA system. They should be native English speakers and strangers. XLab employees will check them in and assign them computers. Make sure you have the name tags filled out and ready to go to give to the XLab employee.

As participants are walking in: Welcome! ______ will check you in and give you a computer. When you sit down, please don't start the survey on the screen until I give you further instructions.

(Once everyone is seated)

Hello! Welcome to the Political Topics Study! Before we start, is anyone here not fluent in English? *(If they are not fluent English speakers, they cannot participate and should not be paid a show up fee)*

Great! We'll start now, but before we do, please put away your cell phones and give me your full attention.

Today, you are going to participate in a study about people's opinions about different topics. The study is divided into 3 parts. In Part 1, you'll read about a few topics and give your opinions on them. In Part 2, we will match you with another participant and assign you to one of the topics from the first part of the study and you will engage with a partner in a conversation about your assigned topic. In Part 3, you will take a survey about your experiences.

Now, please use the computer in front of you to fill out the first survey. You will put your assigned participant number-- the number we put on your nametag-- into the first question. After you finish, please hit the arrow button to make sure your response is submitted and raise your hand so I know you are all done.

(Both RAs should walk around making sure everyone is completing the survey)

Awesome, it looks like everyone is done. Before we move on, I want to make sure everyone hits "next" to complete their survey, and exits out of that screen. You should now be looking

at a blank black screen with 3 Firefox icons on the left side. Is everyone there? *(Walk around and make sure they are all at the same place)*

Cool. We need to quickly review the answers you have given and match you with a partner. While you wait, we have another survey for you to take. Please click on the bookmark on your computer titled "2 PT Personality" and work on that while we match you. Please press the arrow button once you've reached the end to ensure your response is recorded.

(One RA should walk around and make sure that everyone is completing the correct survey while the other RA matches the pairs).

(While one RA is pairing, the other RA, after they finish making sure everyone is on the right survey, should keep checking in on the most recent pair made, and go write the pairs on the board as they are made in the chart on the whiteboard. The "texting" RA should grab the correct instructions to be handed out (asynch/synch).

NOTE: Once the pairs are all up on the board, if the participants are still on the personality survey, just say "Okay, please take the next 15 seconds to finish up your survey. If you haven't completed your last question, that is okay. Just put "N/A" and hit next until you're finished"

Now it's time to start the second part of the study. As you can see, we've placed all of your numbers here on the board. First, note where you are located on the board. The topic in the rightmost box of your row (point to show) will be the topic you are discussing with a partner.

There is a note sheet on your desk. Please take the next two minutes to collect your thoughts about what you want to say about your topic, using your note sheet as needed. Also, don't forget to write your pair number, participant number, and topic on the note sheet. Go ahead and start now, I'll let you know when time is up.

(After 2 minutes)

Your two minutes have passed. Now, for those of you on the top half of the board, you'll be going to the back with ________ (RA). Go ahead and collect your things and follow her to the back.

***<u>SPEAKING CONDITIONS</u>* (for the RA who takes participants to the back room).**

Okay, in the next step you and your partner will be having a conversation about the topic we've assigned you. The room you will be in corresponds with the pair number on your note

sheet. If you're confused, you will also have instructions sheets in your assigned room, and of course, you can always ask me for help!

You and your partner have been matched because you have different opinions on a topic. **Your job today is to get your partner to understand your position and why you hold it.**

<u>Multiple Exchange (Speaking) Condition</u>

Think of this conversation as a debate. This debate should be held in a conversational, back and forth manner.

Does anyone have any questions? *(Answer questions).* Great! I can take you guys to your rooms now. Who is in Pair 1?

Does one of you in each pair have a timer on your phone? *(Wait for them to get it out)* Great! Set the timer for 6 minutes and keep talking about your topic until the timer goes off. When the timer is done, please open your door to let me know that you are done. As a reminder, please only talk about the topic you were assigned.

Bring participant to their respective rooms-- stagger out the rooms. First, take participants to Room 1 and let them start. Then, go back and get participants that go to Room 2, and do the same, and continue until you get to room 5.

Okay, you should be about set to start, since you already had time to collect your thoughts. Because we might need to refer back to your conversation, we will be recording it. These recordings are for research purposes only and your name will never be attached to the conversation.

Start Recorder - say: "This is participant numbers X & X" (read the name tags), pair number X (based on room number), session X . When you are done "First, can I have you please speak your participant number into the recorder? (let them both speak). Ok, let's start the timer now. Great, go ahead and start…" *Leave room.*

Wait for participants to finish. Stop the recorders and collect them.Tell them to go out to the main room once you have.

<u>Single Exchange (Speaking) Condition:</u>

Think of this conversation as a debate. This debate should be held in a structured manner, where you will each prepare a single statement and deliver it to your partner.

Does anyone have any questions? *(Answer questions).*

Do either of you have a timer on your phone? *(Wait for them to get it out)* Great! You will take turns speaking. The person closest to the door will speak first, and the other will follow. Use the timer to make sure each of you speaks for exactly 3 minutes. When the 6 total minutes have passed, please open your door to let me know that you are done. As a reminder, please only talk about the topic you were assigned.

Bring participants to their respective rooms-- stagger out the rooms. First, take participants to Room 1 and let them start. Then, go back and get participants that go to Room 2, and do the same, and continue until you get to room 5.

Okay, you guys should be about set to start, since you already had time to collect your thoughts. Because we might need to refer back to your conversation, we will be recording it. These recordings are for research purposes only and your name will never be attached to the conversation.

Start Recorder - say: "This is participant numbers X & X" (read the name tags), pair number X (based on room number), session X . When you are done "First, can I have you please speak your participant number into the recorder? (let them both speak). "Ok, let's start the timer now. Great, go ahead and start…" *Leave room.*

Wait for participants to finish. Stop the recorders and collect them. Also collect their note sheets and pens. Tell them to go out to the main room once you have.

***WRITING CONDITIONS** (for the RA who stays in the front room with participants)*

(Once the talking participants have filtered out) For those of you in the bottom half of the board, you'll be staying right here. First, I'd like you to find your partner by looking at their name tag and wave so you know who you'll be speaking with. You can find your partner because they will be the person in the same row as you. *(point).*

(once everyone has waved) Does anyone NOT know who their partner is? *(show them if they raise their hand).*

Awesome. Please take note of the computer you are at. It will be located directly to the right of your participant number on the board. The computers all have labels, starting with X01 right here. Now, please collect your things and go to the computer you've been assigned to.

(Once the participants have settled) Awesome, now open up the Chrome tab. It should already be open in the bottom bar on the computer screen, and when you open it you should see a Gmail inbox.

Okay, in the next step you and your partner will be having a conversation about the topic we've assigned you. Here are some instruction sheets you can refer to if you forget what I say, or are confused. *(RA hands out Instruction Sheet).* You and your partner have been matched because you have different opinions on a topic. **Your job today is to get your partner to understand your position and why you hold it.**

***Multiple Exchange (Writing) Condition*:**

Think of this conversation as a debate. This debate should be held in a conversational, back and forth manner. It will occur over G-Chat. You are already signed into the Gmail account you will be using for the conversation. The conversation box in which you and your partner will chat should already be open in the bottom right corner of the screen. Just say hi to your partner and introduce yourself. If you cannot contact your partner, raise your hand and I will help you. I'm going to walk around and make sure each pair has said hello *(RA must walk around computers 1-5 to make sure each pair has gotten in contact with their partner. EVERYONE MUST SAY HI TO THEIR PARTNER BEFORE CONTINUING.)*

Great. Now you're going to have a debate with your partner about the topic that you've been assigned. As a reminder, please only talk about the topic you were assigned.

Does anyone have any questions before we begin? *(Answer questions if necessary)*

Great! Please start now! *(Start timer for 6 minutes)*

RA should walk around to make sure everything is going smoothly. Once 6 minutes have passed, ask the participants to finish their conversation. Once they finish, if the talking participants aren't close to done yet, just give the texting participants instructions for the final survey of the study.

Single Exchange (Writing) Condition:

Think of this conversation as a debate. This debate will be held in a structured manner, where you will each prepare a single statement and deliver it to your partner. You will have about 4 ½ minutes to make a statement to send to your partner. You can continue writing until I say stop, and then you must email your statement to your partner. The email of your partner is located on the post it on your desk. Please send your statement to that email with your participant number from your nametag in the subject line. To help you keep track of the time, I will let you know when you have 1 minute left to write. Remember your job is to explain your position to your partner and get them to understand your position. As a reminder, please only talk about the topic you were assigned.

Does anyone have any questions before we begin? *(Answer questions if necessary)*

Great! Please start now!

RA should walk around to make sure everything is going smoothly. At 3 ½ minutes, tell participants they have 1 minute left to write. Once 4 minutes have passed, tell the participants they must finish and email their position to their partner. Ask if anyone hasn't received their partner's email. Once that is figured out, give them about another 2 minutes to read their partner's position. Once they finish, if the talking participants aren't close to done yet, give the texting participants instructions for the final survey of the study.

Final statement to all participants (can be staggered if time lag for talking)

Now, you will complete the final survey. Please click on the bookmark titled "3 PT Post Survey." Again, you will use the participant number on your name tag to fill out the first question. When you are finished, make sure to hit the arrow button to finish. When you are done, please raise your hand and I will come check for completion and give you permission to go get paid. Please do not exit out of the screen before I have checked for completion. . If you'd like to be debriefed, there are sheets at the table in the back.

Study 2B Script

Participants will be recruited from SONA system. They should be fluent English speakers. XLab employees will check them in, give participant number nametags, and assign them computers.

As participants are walking in, speaking RA: Welcome! When you sit down, please don't start the survey on the screen or talk to other participants until I give you further instructions.

(Once everyone is seated) Hello! Welcome to the Political Topics Study! Before we start, is anyone here not fluent in English? *(If they are not fluent English speakers, they cannot participate and should not be paid a show up fee)*

Great! We'll start now, but before we do, please put away your cell phones and give me your full attention. During this study, it is very important that you do not do anything on your computers until you are told to - please do not close any of the windows unless you are told.

Today, you are going to participate in a study about people's opinions about different topics. The study is divided into 3 parts. In Part 1, you'll read about a few topics and give your opinions on them. In Part 2, we will match you with another participant and assign you to one of the topics from the first part of the study and you will engage with a partner in a conversation about your assigned topic. We will be recording these conversations. Finally, in Part 3, you will take a survey about your experiences.

Now, please review and sign the consent forms [audio condition: and media release forms] on your desk - let me know if you have any questions. [audio condition: You can only be in the study if you initial all of the boxes in the media release form]. Raise your hand when you are done with the forms, and we will collect them. If you wish to keep a copy of the consent form, let me know at the end of the study. *Both RAs should collect all forms. Make sure the consent forms are signed and the media release forms are signed and initialed - must have initials for each row to participate.*

Okay, you may now start the survey on your computer. You will put your assigned participant number-- **the number we put on your nametag - NOT the computer number** -- into the first question. **Please read the questions carefully, as you will be assigned to discuss your answer to one of the topics with a partner.** After you finish the final question on the survey, please hit the final arrow button to make sure your response is submitted and raise your hand so I know you are all done. *(Speaking RA should walk around making sure everyone is completing the survey. Matching RA should open the Qualtrics survey)*

Awesome, it looks like everyone is done. Now, minimize the screen. You should now be looking at the desktop with desktop icons on the left side. Is everyone there? *(Speaking RA walk around and make sure they are all at the same place. The RA in charge of matching should refresh Qualtrics response page and make sure everyone's response shows up.)*

Now, we need to quickly review the answers you have given and match you with a partner. While you wait, we have another survey for you to take. Please click on the bookmark on your computer titled "EXP 2" and work on that while we match you. Please press the arrow

button once you've reached the end to ensure your response is recorded. *(Speaking RA should walk around and make sure that everyone is completing the correct survey while matching RA matches the pairs).* ***Matching:*** *Must be a distance of 3 scale points (-3, -2, -1, 1, 2, 3) so -3's can go with 1's, 2's, 3's, -2's can go with 2's, 3's, -1's can go with 3's*

NOTE: Once the pairs are all up on the board and double checked that no duplicates exist/ all numbers included, if the participants are still on the personality survey, just say "Okay, please take the next 15 seconds to finish up your survey. If you haven't completed your last question, that is okay. Just put "N/A" and hit next until you're finished. If a participant was not matched, they should receive the $5 from XLab staff."

Now it's time to start the second part of the study. As you can see, we've placed all of your numbers here on the board. First, note where you are located on the board. Does anyone not see their number?

Now, since the conversation with your partner will take place over Skype, you will now switch computers where you will be logged into your Skype account. Please take note of your new computer number. It is written directly to the right of your participant number on the whiteboard. The computers all have labels, starting with X01 right here. Now, please collect your things, including your notesheet, and go to the computer you've been assigned to. *(Help participants find computers)*

Okay, the topic in the rightmost box of your row *(point to show)* is the topic you will be debating with your partner. **As a reminder, here is a copy of the specific question that you will be debating.** ***Matching/Speaking RAs: Hand out <u>question prompts</u>, according to assigned topic. Then, hand out instruction sheets.***

You and your partner have been matched because you have different opinions on this topic. Your job today is to get your partner to understand your position and why you hold it. Think of this conversation as a debate. [**writing condition**: You will be **typing** with your partner over Skype's chat feature; **speaking condition**: you will be talking to your partner over a Skype phone call]. You will discuss your topic with your partner for [6 minutes / 12 minutes]

So you have a chance to prepare, there is a note sheet on your desk. Please take the next **<u>three</u>** minutes to collect your thoughts about what you want to say. Use your note-sheet to list as many arguments as you can think of for your topic. Go ahead and start now, and I'll let you know when time is up.

(After 3 minutes) Your time is up. Now open up Google Chrome from the taskbar at the bottom of the computer screen. When you open it, you should already be logged into a Skype account. Do not contact your partner yet.

We will be recording these conversations, so we will quickly walk around to start the screen recordings. *(RAs should walk around and quickly click Start Recording on all computers).*

WRITING CONDITION:

> Since we are recording the screen, please make sure your cursor doesn't block any of the text - move it off to the side of the screen whenever possible. **Great, you will now have [6 minutes / 12 minutes] to have a back-and-forth debate about your topic**. If you run out of things to say about your assigned topic, you may use the time to get to know each other. Please keep the conversation going. You may go ahead. *(15 second warning)* Please finish your conversation.

SPEAKING CONDITION:

> **Do not put your headphones on yet**, but if you're at computers 1-10, go ahead and click on the phone call button to contact your partner - **do not click the video call button**. A permission window may pop up in the left corner - go ahead and hit allow. If you're at computers 11-20, accept the call by clicking the phone call button when you receive it - **do not click the video call button**. If you cannot contact your partner, raise your hand and I will help you. *(Both RAs must walk around to make sure each pair has gotten in contact with their partner.* ***Make sure video cameras are off)***
>
> To test the sound, go ahead and put on your headphones and just say hello. *Make sure everyone can hear each other.* **Great, you will now have [6 minutes / 12 minutes] have a back-and-forth debate about your topic**. If you run out of things to say about your assigned topic, you may use the time to get to know each other. Please keep the conversation going. You may go ahead. *(After 6 or 12 minutes)* Please finish your conversation.

We are now just going to end the screen recordings before moving on. *(RAs should walk around and quickly press on the keyboard shortcut 'F9' on all computers.)*

Finally, minimize the windows until you get to the desktop, and please click on the desktop shortcut titled "EXP 3." Again, you will use the participant number on your name tag to fill

out the first question. When you are finished, make sure to hit the arrow button to finish. When you are done, please raise your hand.* **Please do not exit out of the screen before I have checked for completion.** If you'd like to be debriefed, there are sheets at the table in the back.

Study 2C Script

Participants will be recruited from SONA system. They should be fluent English speakers. XLab employees will check them in, give participant number nametags, and assign them computers.

As participants are walking in, speaking RA:

Welcome! When you sit down, please don't start the survey on the screen or talk to other participants until I give you further instructions. You may start reviewing the consent forms on your desk.

Once everyone is seated:

Hello! Welcome to the Political Topics Study! Before we start, is anyone here not fluent in English?

If they are not fluent English speakers, they cannot participate and should not be paid a show up fee

Great! We'll start now, but before we do, please put away your cell phones and give me your full attention. During this study, it is very important that you do not do anything on your computers until you are told to.

Today, you are going to participate in a study about people's opinions about different topics. The study is divided into 3 parts. In Part 1, you'll read about a few topics and give your opinions on them. In Part 2, we will match you with another participant and assign you to one of the topics from the first part of the study and you will engage with a partner in a conversation about your assigned topic. Finally, in Part 3, you will take a survey about your experiences.

Now, please review and sign the consent forms [audio conditions: and media release forms] on your desk - let me know if you have any questions. [audio conditions: You can only be in the study if you initial all of the boxes in the media release form]. Raise your hand when you

are done with the forms, and we will collect them. If you wish to keep a copy of the consent form, let me know at the end of the study.

Both RAs should collect all forms. Make sure the consent forms are signed and the media release forms are signed and initialed - must have initials for each row to participate.

You may now start the survey on your computer. You will put your assigned participant number-- **the number we put on your nametag - NOT the computer number** -- into the first question. **Please read the questions carefully, as you will be assigned to discuss your answer to one of the topics with a partner. You may use the background information handouts on your desk for more information about the topics.** After you finish the final question on the survey, please hit the final arrow button to make sure your response is submitted and raise your hand so I know you are all done.

RAs should walk around making sure everyone is completing the survey and prepare to download Qualtrics survey data

Awesome, it looks like everyone is done. Now, close the window. You should now be looking at the desktop with desktop icons on the left side. Is everyone there?

Speaking RA walk around and make sure they are all at the same place. The RA in charge of matching should refresh Qualtrics response page and make sure everyone's response has been recorded.

MATCHING

Now, we need to quickly review the answers you have given and match you with a partner. While you wait, we have another survey for you to take. Please click on the bookmark on your computer titled "EXP 2" and work on that while we match you. Please press the arrow button once you've reached the end to ensure your response is recorded.

Both RAs should separately work to match participants - the first RA to match all participants will write the pairs on the board. ***Matching:*** *Must be a distance of 3 scale points (-3, -2, -1, 1, 2, 3) so -3's can go with 1's, 2's, 3's, -2's can go with 2's, 3's, -1's can go with 3's*

NOTE: Once the pairs are all up on the board and double checked that no duplicates exist/ all numbers included, if the participants are still on the personality survey, just say "Okay,

please take the next 15 seconds to finish up your survey. If you haven't completed your last question, that is okay. Just put "N/A" and hit next until you're finished. If a participant was not matched, they should receive the $5 from XLab staff (walk them back to the bench, explain that they weren't matched but they can sign up again, and get their name for the XLab staff).

Now it's time to start the second part of the study. As you can see, we've placed all of your numbers here on the board. First, note where you are located on the board. Does anyone *not* see their number?

Now, since the conversation with your partner will take place over Skype, you will now switch computers where you will be logged into your Skype account. Please take note of your new computer number. It is written directly to the right of your participant number on the whiteboard. The computers all have labels, starting with X01 right here. As you move to your new computer number, please find your partner first and introduce yourself - your partner's participant number is written in the same row as yours. Now, please collect your things, including your background information sheet, note sheet and pen, introduce yourself to your partner and go to the computer you've been assigned to.

Help participants find computers

Okay, the topic in the rightmost box of your row *(point to show)* is the topic you will be debating with your partner. As a reminder, the specific question you will be debating is on the background information handout. We will now quickly collect the background info for the topics you were not assigned to debate and give you some instructions sheets.

Collect background info for topics they weren't assigned to. Make sure they know what topic they are assigned to. Then, hand out ***instruction sheets*** *according to the study condition.*

You and your partner have been matched because you have different opinions on this topic. Your job today is to get your partner to understand your position and why you hold it. Think of this conversation as a debate.

Synchronous Writing condition: You will be **typing** with your partner over Skype's chat feature;

Asynchronous Writing condition: You will be taking turns **typing** statements to your partner over Skype's chat feature

Synchronous Speaking condition: You will be talking to your partner over a Skype phone call.

Asynchronous Speaking condition: You will be taking turns sending voice messages to your partner over Skype chat.

You will discuss your topic with your partner for a total of 12 minutes.

So you have a chance to prepare, there is a note sheet on your desk. Please take the next three minutes to collect your thoughts about what you want to say. Use your note-sheet and background information handout to list as many arguments as you can think of for your topic. Go ahead and start now, and I'll let you know when time is up. *Make sure everyone has a pen and note sheet after the switch.*

CONVERSATION

(After 3 minutes) Your time is up. Now open up Skype from the taskbar at the bottom of the computer screen. When you open it, you should already be logged into a Skype account and connected with another account. Do not contact your partner yet. Is everyone there?

SYNCHRONOUS WRITING CONDITION:

> Great, you will now have 12 minutes to have a back-and-forth debate about your topic over Skype chat. To communicate with each other, just type your messages in the Skype chat box that says, "Type a message here." Please keep the conversation going and only discuss your assigned topic. Does anyone have questions? Start by just typing "hi" to make sure you're connected -- did anyone not get a response? Okay, you may go ahead. *1 minute warning and then at 12 minutes say:* Please finish your conversation.

ASYNCHRONOUS WRITING CONDITION:

> Great, you will now have 12 minutes to have a debate about your topic over Skype chat. This debate will occur in a structured manner: Please take note of the instructions handout. if you are at computers 1-10, you will be "Person A." If you are at computers 11-20, you will be "Person B." Person A will have one minute to type a statement in the Skype chat box that says, "Type a message here." After one minute, you must send your statement to Person B. Person B will have one minute to read the

statement. Then Person B will have one minute to type a statement back and Person A will have one minute to read it. You may finish reading the statement before the one-minute is up, but please do not start typing your next statement - you can use the extra time to think about what you want to say next. We will then repeat this two more times. Please only discuss your assigned topic. Does anyone have any questions? Start by just typing “hi” to make sure you’re connected -- did anyone not get a response?

Read 3x: (text in parentheses only needs to be read for the first time or until participants understand)

Person A, you now have one minute to type a statement - go ahead. (Do not send the message until your one minute is up.) After 1 minute: Person A, please finish your sentence and send your statement to your partner. Person B, you have one minute to read it. (You may not need the full minute to read the statement, so just sit and wait quietly if you finish reading early - do not start typing your next statement, but you may think about what you want to say next.) After 1 minute: Person B, you now have one minute to type a statement - go ahead. (Do not send the message until your one minute is up.) After 1 minute: Person B, please finish your sentence and send your statement to your partner. Person A, you have one minute to read it. (Again, you may not need the full minute to read the statement, so just sit and wait quietly if you finish reading early - do not start typing your next statement, but you may think about what you want to say next.)

After third time: Person A, you have one minute to read it… Do not send any more messages to each other!

SYNCHRONOUS SPEAKING CONDITION:

Do not put your headphones on yet, but if you’re at computers 1-10, go ahead and click on the phone call button to contact your partner - **do not click the video call button**. A permission window will pop up in the left corner - go ahead and hit allow. If you’re at computers 11-20, accept the call by clicking the phone call button when you receive it - **do not click the video call button**. If you cannot contact your partner, raise your hand and I will help you. We are going to record these conversations, so we will also quickly walk around to start the recordings. *Both RAs must walk around to make sure each pair has gotten in contact with their partner and* ***start screen recordings. Make sure video cameras are off***.

To test the sound, go ahead and put on your headphones and just say hi. *Make sure everyone can hear each other.* Great, you will now have 12 minutes to have a back-and-forth debate about your topic. Please keep the conversation going and only discuss your assigned topic. Does anyone have questions? You may go ahead. *1 minute warning and then at 12 minutes say:* Please finish your conversation. We are going to quickly walk around and end the recordings. End recordings.

ASYNCHRONOUS SPEAKING CONDITION:

Great, you will now have 12 minutes to have a debate about your topic over Skype chat. This debate will occur in a structured manner: Please take note of the instructions handout. if you are at computers 1-10, you will be “Person A.” If you are at computers 11-20, you will be “Person B.” Person A will have one minute to record a voice statement by clicking the icon to the left of the Skype chat box - it looks like a video camera in a message bubble and if you hover over it with your mouse it says, “Video Message.” Does anyone not see this? The webcams are not enabled, so the video will just be a black screen. After one minute, you must send your voice statement to Person B. Person B will have one minute to listen to the statement. Then Person B will have one minute to record a voice statement to send back and Person A will have one minute to listen to it. We will then repeat this two more times. Please only discuss your assigned topic. Does anyone have any questions? One more note, after you listen to your partner’s voice statement, please close the screen by clicking the X on the *left* corner of the window. If you click the X on the right, you will close Skype.

Test: Read through quickly once and then slowly, step-by-step as participants actually do it.

Don’t do this yet, but to test the sound, I will ask you to put on your headphones, click the video message icon, click record only once and wait for the button to turn red, once the button is red you’ll just say “Hi”, click the record button again to end the recording, and then click the send button. Ok, so go ahead and put on your headphones (make sure the microphone is directly in front of your mouth but not too close), click the video message icon, click record only once and wait for the button to turn red, once the button is red just say Hi, then end the recording and send it. Listen to your partner’s message to make sure you can hear them. Did this not work for anyone?

Read 3x:

Person A, you now have one minute to record a voice statement - go ahead and click the "video message feature" and *click the record button*. After 1 minute: Person A, please finish your last sentence, *stop the recording and send* your voice statement to your partner. Person B, you have one minute to listen to it - when you're done click the X on the left corner of the window so I know you're done. After 1 minute: Person B, you now have one minute to record a voice statement - go ahead and click the video message feature and *click the record button*. After 1 minute: Person B, please finish your last sentence, *stop the recording and send* your voice statement to your partner. Person A, you have one minute to listen to it - when you're done click the X on the left corner of the window so I know you're done.

POST SURVEY

Finally, minimize the window to get to the desktop, and please click on the desktop shortcut titled "EXP 3." Again, you will use the participant number on your name tag to fill out the first question. When you are finished, make sure to hit the arrow button to finish. When you are done, please raise your hand.* Please do not exit out of the screen before I have checked for completion. If you'd like to be debriefed, there are sheets at the table in the back.

Study 3 Script

At all sites, the experimenters and research assistants followed this procedure: Upon arrival, participants are handed a nametag with a participant number and an information handout, including the condition to which they have been assigned (writing or speaking). If in the writing condition, their handout should also have the Skype ID login and password. Participants must wear the nametag visibly. If in the writing condition, participants will log into Skype using their assigned account to make sure it's working properly. Participants are directed to sit in one of two groups to which they have been assigned: Writing or Speaking. Once participants are seated with the proper equipment and all writing condition participants are signed into their Skype accounts, we introduced the event:

<u>At University of California, Berkeley ("UC Berkeley"), participants were told:</u>

"Welcome to our Political Speed Dating event, hosted by BridgeUSA and the UC Berkeley Mind and Person Perception Lab. Our goal for the evening is to give you the opportunity to interact with multiple people who might have different opinions than you do. Each of the conversations will be one-on-one and we've assigned you to a partner. As you've noticed, we've put you into two different groups. This group over here will start by having Skype conversations, and the other group will talk in person. But at some point you'll get the chance to switch groups. The reason we are asking you to interact in different ways is to conduct some research about how this might influence your interactions."

<u>At Minnesota State University, Mankato ("MNSU"), participants were told:</u>

"Welcome to our Political Conversations event, hosted by BridgeMNSU and the UC Berkeley Mind and Person Perception Lab. Our goal for the evening is to give you the opportunity to interact with multiple people who might have different opinions than you do. Each of the conversations will be one-on-one and we've assigned you to a partner for each round. As you've noticed, we've put you into two different groups. This group over here will have Skype conversations, and the other group will talk in person. The reason we are asking you to interact in different ways is to conduct some research about how this might influence your interactions."

<u>At Arizona State University ("ASU"), participants were told:</u>

"Welcome to our Political Conversations event, hosted by BridgeASU and the UC Berkeley Mind and Person Perception Lab. Our goal for the evening is to give you the opportunity to interact with multiple people who might have different opinions than you do. Each of the conversations will be one-on-one and we've assigned you to a partner for each round. As

you've noticed, we've put you into two different groups. This group over here will have Skype conversations, and the other group will talk over skype/in person. The reason we are asking you to interact in different ways is to conduct some research about how this might influence your interactions."

At all sites: Once participants were seated, they opened their laptops and followed the URL on the board to complete a consent form and pre-survey. Specifically, participants were told: "Before any conversations begin, please open up your laptop and follow the URL written on the whiteboard to complete a consent form and a short pre-survey about your political opinions. You will be asked to enter your participant number - the number from your nametag into the first question. Every survey that you complete will ask about your participant number, so please do not take off your sticker throughout the event. Let us know if you did not receive a number. You may refer to the background information sheet we handed out if you'd like more information about the topic."

When everyone was done with the pre-survey, participants were told to start the first conversation.

At UC Berkeley, participants were specifically told:
"Great, it looks like everyone is done! The person sitting across from you is the person with whom you'll be talking. You will now have an 8 minute conversation about the political topic with this person. If you are on [this side of the room], you will have typed conversations over Skype. If you are on [this side of the room], you will simply talk to each other. Please start your conversation with your partner now. We'll give you a minute warning."

At MNSU, participants were told:

"Great, it looks like everyone is done! The person sitting across from you is the person with whom you'll be talking. You will now have an 8 minute conversation about the political topic with this person. If you are on [this side of the room], you will have typed conversations over Skype chat. Please exchange Skype account information and connect with each other via Skype chat. Just type "hello" to make sure it's working then wait for further instruction. Raise your hand if you need help. If you are on [this side of the room], you will simply talk to each other. Please start your conversation with your partner now. As a reminder, please only discuss your assigned topic. You have 8 minutes, and we'll give you a 1-minute warning."

At ASU, participants were told:

"Great, it looks like everyone is done! The person sitting across from you is the person with whom you'll be talking. You will now have a 6 [12] minute conversation about the political topic with this person. If you are on [this side of the room], you will have text conversations over Skype chat. Please exchange Skype account information and connect with each other via Skype chat. Just text "hello" to make sure it's working then wait for further instruction. Raise your hand if you need help. If you are on [this side of the room], you will simply talk to each other (over skype, so please exchange skype account information and everyone on the right side of the table should call their partner, and everyone on the left side of the table should accept the call). Please start your conversation with your partner now. As a reminder, please only discuss your assigned topic. You have 6 [12] minutes, and we'll give you a 1-minute warning."

At all sites: After each conversation, participants completed a short post-survey (write URL on board).

At all sites, participants were then told:

"Your time is up! Please follow this URL on the whiteboard to answer a few quick questions about your conversation. In the first two questions, you will enter your participant number and your partner's participant number. Remember, these questions are completely confidential and will never be shared with your partner, so please be honest! [Wait until everyone is done with the survey: Raise your hand if you're still working on the survey.] Okay, now if you're seated on the inside of the circle, you will rotate your chairs to the right so you have a new conversation partner."

At all sites, research assistants followed these instructions: For the second conversation, there is no need to give participants more preparation time (just provide enough time for participants to connect with their partner on Skype). After at least four conversations (as many as time allows), hand out the debriefing statement.

The research assistants running the study were also tasked with assigning participants to a partner based on where they sat (and making sure participants weren't instead selecting their conversation partners), as well as making sure people's name-tags (with their participant numbers) were visible to their partner. If one participant took significantly longer to complete a survey than the rest, the research assistants were told to request that the participant move on without finishing the survey. Research assistants were told that all pairs should be speaking or writing during the same time period.

Note that all participants could see their conversation partner, even when they were conversing via text using the laptops in front of them.

Supplementary Information R - Dependent Measures Across Studies

Below we report the primary dependent measures collected in Studies 1-4, the Annotation Study (reported in Supplementary Information G), and the Study 6 (see details in supplementary Information R and S). For each index, items were presented in randomized order to participants.

Perceived conflict. For Studies 1, 2, 4 and the Lay Theory Study, we asked four items to measure perceived conflict (Study 1: $\alpha = .76$; Study 2A: $\alpha = .79$; Study 2B: $\alpha = .80$; Study 2C: $\alpha = .78$; Study 4: $\alpha = .86$; Lay Theory Study: $\alpha = .92$). We created this scale by drawing from Jehn's (1995) scale of intragroup relationship conflict scale but adapting it for dyadic disagreements. Specifically, we asked participants about "how your conversation with your partner went today?". For each item, participants responded on a seven-point Likert scale from (0 = no conflict at all, 6 = a great deal of conflict).

1. How contentious was the conversation with your partner?
2. How emotional was the conversation with your partner?
3. How heated was the conversation with your partner? (Excluded from Lay Theory Study)
4. How much conflict did you feel during your conversation with your partner?

In Study 3, where we partnered with BridgeUSA, perceived conflict was assessed with a single question, "How much conflict did you feel during your conversation?" on a seven-point Likert scale from 0 (No conflict at all) to 6 (A great deal of conflict) at Minnesota State University and Arizona State University. The question was adapted slightly at UC Berkeley where we asked participants, "How much conflict did you feel during your conversation with the other person?" on the same seven-point Likert scale. In Study 4, where we collected ratings from observers, we asked the four questions above, but referring to "the conversation between Person A and Person B" instead of "the conversation with your

partner." In the Annotation Study, where we also collected ratings from observers, perceived conflict was assessed with a single question, "How much conflict occurred between the pair in Exchange [x]?" on a seven-point Likert scale from 0 (No conflict) to 6 (A lot of conflict). In the Lay Theory Study, which measured predictions, the items were changed to the future tense (e.g., "How contentious will the conversation with your partner be?"), and participants made predictions for each of the three conversation mediums ("During the video/phone / written conversation…").

In Table S23, we report the correlation across Studies 1 and 2 for the four perceived conflict items. All items show strong positive correlations with one another.

Supplementary Table S23. Correlation matrix of perceived conflict items used in Studies 1 and 2.

	Contentious	Emotional	Heated
Emotional	.496		
Heated	.490	.601	
Conflict	.414	.438	.697

Understanding (perceived and actual). For Studies 1-3 and the Lay Theory Study, we used two items to measure perceived understanding (Study 1: α = .67; Study 2A: α = .68; Study 2B: α = .69; Study 2C: α = .73; Study 3: α = .78; Lay Theory Study: α = .88). For each item, participants responded on a seven-point Likert scale from 0 (Did not understand at all) to 6 (Understood extremely well).

1. To what extent do you think your partner understood your position?

2. To what extent do you think you understood your partner's position?

In Study 3, the term 'opinion' replaced 'position' in the scale items. In Study 4, where we collected ratings from observers, understanding was assessed with a single question,

“How well did Person A and Person B understand each other?”. In the Annotation Study, where we also collected ratings from observers, understanding was assessed with a single question, “How much understanding occurred between the pair in Exchange [x]?” on a seven-point Likert scale from 0 (No understanding) to 6 (A lot of understanding). In the Lay Theory Study, which measured predictions, the items were changed to the future tense (e.g., “To what extent do you think you will understand your partner’s position?”) and participants made predictions for each of the three conversation mediums (“By the end of the video/phone / written conversation…”).

Finally, Study 3 also collected a measure of *actual understanding*: “If you had to guess, to what extent do you think your conversation partner opposes or supports [the topic]?” on the same response scale that the participant had reported for themselves (seven-point Likert scale from “Completely opposes” to “Completely supports”).

Perceived humanisation. For Studies 1, 2 and 4, we used five items from (Haslam & Reicher, 2006) humanisation scale (Study 1: $\alpha = .81$; Study 2A: $\alpha = .85$; Study 2B: $\alpha = .82$; Study 2C: $\alpha = .85$; Study 4: $\alpha = .90$). In Study 3, the Annotation Study, and the Lay Theory Study perceived humanisation was not assessed. We asked participants to “please indicate the degree to which you agree with the following statements about your partner compared to the average person, using the scale below.” For each item, participants responded on a seven-point Likert scale from -3 (much less than average) to 3 (much more than average).

1. My partner is refined and cultured.
2. My partner is impulsive and lacking self-restraint.
3. My partner is rational and logical.
4. My partner is unsophisticated.
5. My partner is like a fully developed adult rather than a child.

In Study 4, where we collected ratings from observers, we asked the five questions above for “Person A” and “Person B” in the conversation, separately. Table S24 shows the correlation across Studies 1 and 2 for the five perceived humanisation items; all items in the scale correlated significantly with one another in the expected directions.

Supplementary Table S24. Correlation matrix of perceived humanisation items used in Studies 1 and 2.

	Refined	Impulsive	Rational	Unsophisticated
Impulsive	-0.369			
Rational	0.662	-0.414		
Unsophisticated	-0.479	0.545	-0.476	
Developed Adult	0.610	-0.403	0.629	-0.498

Perceived competence. For Studies 1, 2, 4 and the Lay Theory Study, to assess participants’ *perceived competence* of their partners, we measured five items (Study 1: α = .94; Study 2A: α = .95; Study 2B: α = .92; Study 2C: α = .93; Study 4: α = .95; Lay Theory Study: α = .95). For each item, participants responded on a seven-point Likert scale from 0 (not at all) to 6 (very). Competence was not measured in the Annotation Study.

1. Overall, how competent do you consider your partner to be after your conversation?

2. Overall, how thoughtful do you consider your partner to be after your conversation?

3. Overall, how logical are your partner's beliefs (based on your conversation)?

4. Overall, how rational are your partner's beliefs (based on your conversation)?

5. Overall, how well-reasoned are your partner's beliefs (based on your conversation)?

In Study 3, where we partnered with BridgeUSA, perceived competence was assessed with a single question at Minnesota State University and Arizona State University, "During the conversation, how reasonable did your conversation partner's opinion seem?" on a seven-point Likert scale from 0 (No very reasonable) to 6 (Extremely reasonable). At the UC Berkeley site in Study 3, we asked a slight variation, "Overall, how competent do you consider the other person to be, after your conversation?" on a seven-point Likert scale from 0 (No at all competent) to 6 (Extremely competent). In Study 4, where we collected ratings from observers, we asked the five questions above for "Person A" and "Person B" in the conversation, separately. In the Lay Theory Study, which measured predictions, the items were changed to the future tense (e.g., "How competent will you consider your partner to be?") and participants made predictions for each of the three conversation mediums ("By the end of the video/phone / written conversation… "). Table S25 shows the correlation across Studies 1 and 2 for the five perceived competence items; all items in the scale correlated significantly and positively with one another.

Supplementary Table S25. Correlation matrix of perceived competence items used in Studies 1 and 2.

	Competence	Thoughtful	Logical	Rational
Thoughtful	0.752			
Logical	0.738	0.699		
Rational	0.705	0.666	0.850	
Well-reasoned	0.716	0.690	0.816	0.807

Attitudes. We examined changes in participants' attitudes in Studies 1-3 by taking the difference between post-conversation attitudes on the topic of discussion (reported in the

post-survey) and pre-conversation attitudes (reported in the pre-survey). Then, we reversed half the scores so that at all times, a positive score means moving in the direction of the partner's opinions, and a negative score indicates moving away from the partner's opinions. The specific attitude measures are reported in Supplementary Note 15.

Liking. In Studies 1 and 2, to assess how much participants liked each other after the conversation, we asked: "Overall, how much do you like your partner" on a seven-point Likert scale from 0 (Do not like at all) to 6 (Like a great deal). In Study 3, where we partnered with BridgeUSA, liking was assessed with a single question, "During the conversation, how much did you like your conversation partner?" on a seven-point Likert scale from 0 (Do not like at all) to 6 (Like a great deal) at Minnesota State University and Arizona State University. At UC Berkeley for Study 3, we asked "Overall, how much do you like the other person?" on the same seven-point Likert scale. In Study 4, we asked observers to rate both Person A and Person B with a single question, "Overall, how much do you like Person [x]?" on a seven-point Likert scale from 0 (Do not like at all) to 6 (Like a great deal). In the Annotation Study, where we collected ratings from observers, liking was assessed with a single question, "How much do Person A and Person B seem to like each other in Exchange [x]?" on a seven-point Likert scale from 0 (Don't like each other at all) to 6 (Like each other a lot). In the Lay Theory Study, we asked participants a single-item question, "By the end of the video/phone / written conversation, how much will you like your partner?" on a seven-point Likert scale from 0 (Not at all) to 6 (Very).

Responsiveness. To assess how responsive participants were to one another in Studies 1 and 2, we asked "How responsive was your partner toward you during the conversation (that is, how much did he/she seem to understand, value, and support your opinion)?" on a

seven-point Likert scale from 0 (Not at all responsive) to 6 (Very responsive). In Study 4, we amended the scale for the perspective of an observer and asked, "How responsive Person A and Person B seem towards each other (That is, how much do they seem to understand, value, and support each other's opinions)?" on a seven-point Likert scale from 0 (Not at all responsive) to 6 (Very responsive). Responsiveness was not assessed in Study 3, the Lay Theory Study, or the Annotation Study.

Perceived common ground. To assess how much participants believed they found common ground in Studies 1 and 2, we asked: "How much did you and your partner find common ground (i.e. areas of agreement) in the conversation?" on a seven-point Likert scale from 0 (No common ground) to 6 (A lot of common ground). In Study 4, we amended the scale for the perspective of an observer and asked, "How much did Person A and Person B find common ground (i.e. areas of agreement) in the conversation?" on a seven-point Likert scale from 0 (No common ground) to 6 (A lot of common ground). Common ground was not assessed in Study 3 or the Annotation Study. In the Lay Theory Study, we asked, "During the video / phone / written conversation, how much will you and your partner find common ground (i.e., areas of agreement) in the conversation?" on a seven-point Likert scale from 0 (No common ground) to 6 (A lot of common ground).

Enjoyment. To assess how much participants enjoyed the conversation in Studies 1 and 2, we asked: "How much did you enjoy the conversation with your partner?" on a seven-point Likert scale from 0 (Did not enjoy at all) to 6 (Very much enjoyed). In Study 4, we amended the scale for the perspective of an observer and asked, "How much did Person A and Person B enjoy the conversation?" on a seven-point Likert scale from 0 (Did not enjoy at all) to 6 (Very much enjoyed). In Study 3, we asked participants at UC Berkeley "How much

did you enjoy the conversation?” on a seven-point Likert scale from 0 (Did not enjoy at all) to 6 (Very much enjoyed). Enjoyment was not assessed at Minnesota State University or Arizona State University in Study 3 or the Annotation Study. In the Lay Theory Study, we asked, “During the video/phone / written conversation, how much will you enjoy the conversation with your partner?” on a seven-point Likert scale from 0 (Did not enjoy at all) to 6 (Very much enjoyed).

Perceived agreement. In Studies 1 and 2, to assess how much participants agreed with each other after the conversation, we asked, ‘At the end of the conversation, how much do you now agree or disagree with your partner’s views?’ on a five-point Likert scale from 1 (I strongly disagree) to 5 (I strongly agree). In Study 3, we asked, “How much [do you now agree/did you agree] with your conversation partner’s opinions?” at Minnesota State University and Arizona State University, and “How much did you agree with the other person?” at UC Berkeley on a seven-point Likert scale from 0 (Complete disagreement) to 6 (Complete agreement). In Study 4, we amended the scale for the perspective of an observer and asked “By the end of the conversation, how much did Person A and Person B agree with each other?” on a five-point Likert scale from 1 (They strongly disagreed) to 5 (They strongly agreed). In the Annotation Study, agreement was assessed with a single question, “How much agreement occurred between the pair in Exchange [x]?” on a seven-point Likert scale from 1 (Complete disagreement) to 7 (Complete agreement). In the Lay Theory Study, we asked two items to examine predicted agreement ($\alpha = .92$): “By the end of the conversation, how much will you agree or disagree with your partner’s views?” and “By the end of the conversation, how much will your partner agree or disagree with your views?” on a seven-point Likert scale from 0 (Will strongly disagree) to 6 (Will strongly agree).

In addition, we also assessed how much participants thought they agreed with each other before the conversation in Studies 1 and 2. We asked, “At the beginning of the conversation, how much did you agree or disagree with your partner's views?” on a five-point Likert scale from 1 (I strongly disagreed) to 5 (I strongly agreed).

Perceived influence. In addition to changes in attitudes towards the topic from pre- to post- conversation, we also asked participants in Study 3 at UC Berkeley [and at Minnesota State and Arizona State University] after each conversation, “To what extent do you think the other person [your conversation partner] influenced your own opinion?” on a seven-point Likert scale from 1 (Did not influence at all) to 7 (Extremely influenced).

Manipulation checks. In Studies 1, 2 and 4, we asked a series of manipulation check questions. No manipulation check questions were asked in Study 3. In Study 1, we asked participants, “How did you communicate with your partner today?”. Participants selected one of the following responses: “We typed in a Skype Chat”, “We talked via a Skype call (No Video)”, or “We talked via a Skype Video call”. In Study 2A, we asked participants, “How did you communicate with your partner today?”, where they selected one of the following response options: “I typed on a computer” or “I talked (in person)”. We also asked participants in Study 2A, “ Did you communicate in a structured or a conversational manner?”, where participants selected one of the following response options: “I communicated in a structured manner: both my partner and I gave one long response” or “I communicated in a conversational manner: both my partner and I went back and forth”. In Study 2B, we asked participants, “How did you communicate with your partner today?”, where participants selected one of the following response options: “We typed in a Skype Chat”, “We talked via a Skype Phone Call”, or “Other”. In Study 2C, we asked participants,

“Please read all options below before selecting the one that best describes how you communicated with your partner today:”, where participants selected one of the following response options: “We typed in a Skype Chat without following any particular conversation structure”, “We typed one minute statements and sent them to each other via Skype chat”, “We talked via a Skype Phone Call”, “We recorded voice statements and sent them to each other via Skype Chat”, or “Other”. In Study 4, we asked, “What did you do in this study?”, where participants selected one of the following response options: “I read the transcription of Person A and Person B's original (spoken) statements.”, “I listened to the spoken version of Person A and Person B's original (written) statements.”, “I read Person A and Person B's original (written) statements.”, or “I listened to Person A and Person B's original (spoken) statements.”. In Study 4, we also asked, “Which topic did Person A and Person B discuss in their conversation?” where participants selected one of the following response options: “Lowering the drinking age”, “GMOs”, or “Enforcing admissions quotas”.

Demographic questions. In Studies 1, 2, and 4, the following demographic questions were collected at the end of the post-survey:

1. “What is your gender?” where participants selected one of the following response options: “Male”, “Female”, “Other (please enter below)”.
2. “What is your highest level of education completed?” where participants selected one of the following response options: “High School”, First year of college”, “Second year of college”, “Third year of college”, “Fourth year of college”, “Bachelors”, “Masters”, “Doctorate”.
3. “What is your age?”
4. “Are you currently affiliated with UC Berkeley (and if so, in what capacity)?”, where participants selected one of the following response options: ”Not

affiliated with UC Berkeley", "I am staff at UC Berkeley", "I am an undergraduate student at UC Berkeley (or will be an undergraduate student

5. soon)", "I am a graduate student at UC Berkeley", "I am faculty at UC Berkeley, Other (please enter a response)".
6. "Choose one or more races that you consider yourself to be", where participants selected one of the following response options:
7. "White", "Black or African American", "American Indian or Alaska Native", "Asian" "Native Hawaiian or Pacific Islander", "Other".
8. "What is your political orientation?" on a five-point Likert scale from 1 (Very conservative) to 5 (Very liberal).
9. "How would you describe your fluency in English?" on a five-point Likert scale from 1 (Not fluent at all) to 5 (Very fluent).

For Study 3, we collected demographics data at the end of the pre-survey, substituting UC Berkeley with either ASU or MNSU accordingly in the question on institution affiliation. We also asked participants "How would you define your political party affiliation (if any)?" and "Why did you decide to come to the event today?". We did not ask participants about their English fluency for study 3.

For the Lay theory study, we asked participants the same demographic questions as for Studies 1, 2 and 4, except from English fluency and institution affiliation.

Personality questions. In Studies 1 and 2, we measured the Big-5 44-item survey (John & Srivastava, 1999) in a separate survey conducted after the pre-survey while dyads were being matched for their conversation. This survey specifically measures five personality factors: openness to experience, conscientiousness, extraversion, agreeableness, and neuroticism (emotional stability).

Below, we report three more scales we measured only in the Lay Theory Study.

Predicted discomfort. We measured predicted discomfort using two items (α = .85). For each item, participants responded on a seven-point Likert scale from 0 (very uncomfortable) to 6 (very comfortable).

1. During the video / phone / written conversation, how uncomfortable or comfortable will you feel?
2. During the video / phone / written conversation, how uncomfortable or comfortable will your partner feel?

Predicted effort. We measured predicted effort using two items (α = .77). For each item, participants responded on a seven-point Likert scale from -3 (very hard) to 3 (very easy).

1. During the video / phone / written conversation, how hard or easy will it be to convey your true intent?
2. During the video / phone / written conversation, how hard or easy will it be for your partner to convey their true intent?

Predicted awkwardness. We measured predicted awkwardness by asking, "During the video / phone / written conversation how awkward will the conversation be?" on a seven-point Likert scale from 0 (Not at all) to 6 (Very).

Supplementary Information S - Transcription and Reading Guides Used in Study 4

For Study 4, we transcribed the spoken conversations and had voice actors read aloud the written conversations. For transcription, we used a reputable transcription service (temi.com) to create a first version of the transcript, which was manually checked by at least two research assistants. The research assistants followed the guidelines below to create transcriptions that would be adequately readable for the Study 4 participants. For voice actors, we recruited two male and two female research assistants to speak aloud the written conversations. We gender-matched the voice actor (using the actor's stated gender) to the original participant's self-reported gender. The voice actors recorded in a recording studio, two at a time (in male/male, male/female, and female/female sessions) so that they could feel as though they were having a real conversation, making the experience more natural.

To conduct the NLP analysis, we used participants' original writing or the transcripts that were created by temi.com and checked by two research assistants, but removed the "Person A" and "Person B" demarcations as well as any context words in brackets that research assistants had added for the Study 3 readers ("[Inaudible]"; "[Experimenter says time is up]"; "[Name redacted]"; "[Timer goes off]", e.g., "I put a 2 [on the scale]").

—-------------

<u>TRANSCRIPTION GUIDE</u>

In general, we want to stay true to what the participants originally said. However, we also want these transcripts to be readable so that other participants can evaluate them. The instructions below clarify what should be transcribed verbatim and what should be fixed. If you run into something in a transcript that isn't clear, do not make up rules. Always try to be consistent with what the communicator was trying to express.

- **Do not include sound check** (e.g., “Hi, can you hear me?”) but you can include pleasantries exchanged after confirming the sound works (e.g., “How are you?”)
- **Make sure you are typing the correct words**
  - If it doesn’t make sense, you may not be hearing the right words
  - Listen carefully and turn the volume up
  - Use context, use Google!
  - If you really can’t understand, write “[inaudible]”
- **Make sure you label Person A and Person B correctly**
  - Each change in participant should be a new line.
  - Person A should support the topic, Person B should oppose
  - A and B should always alternate, even if there is a long pause.
    - The only exception is when the Experimenter says time is up. At that point, write in the transcript: “[Experimenter says time is up]”
  - Always be very careful: Sometimes labels get switched halfway through the transcript. Check to be sure they are consistent.
- **Fix spelling errors and typos**
  - For example, “alright” should be “all right”.
- **Fix grammar *mistakes***
  - Add periods and commas in run-on sentences between complete thoughts.
  - Fix subject/verb agreement: If they say “some is” change to “some are” - this is usually not how the person would write.
  - Fix capitalization (e.g., acronyms should be capitalized)
    - § US should be changed to U.S.
    - § Capitalize “God” (e.g., “oh my God”).
  - Fix sentence fragments (e.g., “Okay cool” to “Okay, cool.” or “Okay. Cool.”) -

this includes “Oh, cool.”

- Change “gonna”, “sorta”, “kinda”, “dunno” to “going to”, “sort of”, “kind of”, “don’t know” - this isn’t intentional/ just how people speak.
- Use “because” instead of cuz or ‘cause.
- Use double dashes to indicate a change in thought (e.g., “I support legalizing drugs because I -- do you know if the researcher means all drugs?”) but do not indicate unintentional stutters or struggles to find the right phrases (it has to be a complete change in thought).
- Don’t put commas after “And,” “So,” or “But” when starting a sentence (e.g., “And I think that…”) because they are more like continuations of run-on sentences than transition words.
    - Keep poor grammar if it’s really what the person intended or how the person would probably write -- for example, if the person used “less” instead of “fewer”.
- Capitalize White and Black when referring to race.

- **Take out filler words or unintentional utterances that do not change the meaning of the sentence:** uh, um, meaningless use of “like”, excessive use of “so”...
    - “You know?” can be a filler word - take it out when participants say “you know?” without waiting for a response or use it excessively.
    - “I mean” is another example. Keep it in only if it seems meaningful and like the speaker is trying to clarify their meaning.
        - Keep in instances of “I think” as it’s too difficult to determine when this is meaningful. Remove if so frequent (I think I think) that it could be a stutter or tic.
    - “I don’t know” and “I guess” should be taken out if used excessively or if the

person uses them as a filler (to fill space) and not to convey meaning.

        - Does “I guess” indicate real uncertainty? If not, do not include it in the transcript.

    - Sometimes “uh” might be meaningful if used to denote uncertainty about what the other person said.
    - Take out “I feel like” if it’s excessive and doesn’t contribute to the meaning.
    - Remove repeated words and stutters unless they were intentional.
    - Remove verbal mistakes: sometimes participants speak quickly and say the wrong word before correcting it. In such a case, only include the correct word (e.g., “I think that racism should be acceptable -- should be unacceptable” should just be written as “I think that racism should be unacceptable”).
    - Note: sometimes word repetitions are purposeful! “No no no” could be a stutter or it could be an emphasized no. Try to determine the speaker’s intentions. If intentional, leave in repetitions with a comma in between: “no, no, no”

- **Include backchannels**
    - Use consistent spelling: “mm-hmm”, “hmm”, “mm”, “uh-huh”, “yeah”, “ah”, “Ooh”.
    - Insert only between complete thoughts (do not interrupt the other person’s sentence as it makes it hard to read).
    - Follow with a period.
    - Do not insert excessive back-channels interrupting the other’s statement. Just insert the *last* back-channel at the end of the other’s statement.
        - For example, in the transcript below all of the “uh-huh”s here should be removed except for the last one:

Person B: Um. To be honest, this -- when the whole Milo thing happened –

Person A: Uh-huh.

Person B: -- last sem -- or two semesters ago, it was really traumatic. So that's -- I just don't even have any energy to debate --

Person A: Uh-huh.

Person B: -- about --

Person A: Uh-huh.

Person B: -- things like this --

Person A: Uh-huh.

Person B: -- because my positionality as a black woman is really unique and different.

Person A: Uh-huh.

Person B: So, debating my experiences is pointless.

Person A: Uh-huh. I think that...

If the back-channeling isn't consistently the same word (e.g., it isn't always "uh-huh"), use the last back-channel the person said.

- **Do not include interruptions unless they are purposeful** (e.g., the person is trying to cut in). If they just accidentally started talking at the same time, don't include - it comes across as a purposeful interruption when reading it (but most interruptions aren't purposeful)
    - **Example of purposeful interruption for formatting example:**

      Person A: But, do they have to? Why should they have to work harder than everyone else when they have been working harder for hundreds of years, when this country was built on their backs? Our infrastructure and our wealth

came from plantation workers. That's why our country is so prosperous. Because Black people did that work.

Person B: Yes, what happened in the past sucks. Obviously no one would ever say that was good, but it happened in the past, and it sucks and --

Person A: And so what?

Person B: I think --

Person A: Tough luck?

Person B: Yes. Life just isn't fair sometimes, and sometimes you have to work hard because of things that happened, and it sucks, but you just kind of have to do it.

- **Add supplemental words to clarify meaning in brackets only when the sentence wouldn't make sense without it** (e.g., "I put a 2 because I strongly support it." should be "I put a 2 [on the scale] because I strongly support it.)
    - Some Berkeley-specific references may need clarification. Non-UC Berkeley participants should be able to understand the transcript.
    - If you feel like you need to add more than a couple words or re-write the entire sentence, it may be best to leave the sentence as-is (we don't want to change it too much from the original). Try your best to see if there's a way to make it clear without changing much. Ask others for help!
- **Quoting:** When someone is quoting something that they or someone else might say in a sentence, put it in quotations (""). Use double quotes. This isn't always an obvious set-up using "like" or "say". Sometimes it is denoted by the change in a person's voice or unusual sentence structure (e.g., I think there are some that are just, "All right, that's not cool.")
    - Capitalize the first word in the quote and put a comma before the quote

starts

- **Include when experimenter says time is up as "[Experimenter says time is up]".** If the experimenter does not interrupt the conversation, just include this line at the end of the transcription.
    - This should be on its own line and in bold font.
    - Long vs. Short:
        - Long: [Experimenter says time is up] at the end of the dialogue
            - 1 per transcript
        - Short: [Experimenter says time is up] at the end of the dialogue
            - 1 per transcript
    - Single exchanges vs. multiple exchanges:
        - Single exchanges: [Timer goes off] after each single exchange
            - 2 per transcript
            - If the person didn't talk for the full 3 minutes or had a dialogue instead, okay to leave out
        - Multiple exchanges: [Timer goes off] at the end of multiple exchange
            - 1 per transcript
    - Audio, Video, Text:
        - Audio/Video: [Experimenter says time is up] after each single exchange and at the end of multiple exchange
            - 3 per transcript
    - Berkeley Bridge:
    - Synchronous vs Asynchronous:
- **Do not record nonverbals** (e.g., "[laughs]")
- **Do not add ellipses** (...)

- **Do not add exclamation marks**
- **Do not include the RA talking** and do not to transcribe background participants
- **Include even irrelevant parts of the conversation** in the transcript
- **Redact the participants' names**. If the participant says their name, in the place where their name was said, write: [name redacted].

Include curse words. Write the full word into the transcript (e.g., "Damn").

<u>READING ALOUD GUIDE</u>

For each conversation, you will do at least two (but possibly more) readings together with your conversation partner. Record all of the readings that you do.

- The first reading is a practice reading. For this reading, focus on ensuring that you speak each word correctly and that you understand what you are reading. If you identify any phrases or statements that are challenging to speak (e.g., that translate awkwardly into spoken language), please make a note for the experimenters. If the writers use acronyms (e.g., omg, lol, rofl, fml), please just speak aloud each letter in the acronym.
- For your second reading, focus on speaking the correct words while also imbuing your words with the thoughts, emotions, and substance that the original writer felt. For this reading, please try to imagine that you are the person who wrote the words. We want you to read the words as if you were actually coming up with the lines naturally off the top of your head, as in a real conversation, rather than reading from a written conversation.
- If needed, you can do more readings together. Continue until all words have been spoken correctly and with the appropriate tone that mirrors what you think the original writer meant to convey. You can give feedback to each other as you do the

readings.

- After you believe you are done with readings, listen to the final reading in its entirety to make sure it sounds OK. If there are issues with the recording (e.g., background noise, static), please re-do the reading.

Supplementary Information T - Materials for Study 6

Please imagine that you are going to participate in another study RIGHT NOW, along with another Prolific participant. First, you and the other person will each complete an online survey in which you report your opinions about three different issues. These issues were pre-selected because they are highly contentious.

You can read the three issues below.

ISSUE 1: DO YOU SUPPORT OR OPPOSE LOWERING THE LEGAL DRINKING AGE IN THE U.S.?

Proponents of lowering the legal drinking age argue that in many other countries (e.g., in Europe), minors are typically considered old enough to drink beer and wine. They believe minors are old enough to have the autonomy to choose whether or not they would like to consume alcohol.

Opponents of lowering the legal drinking age argue that lowering the drinking age would be detrimental because the brain is not fully developed in minors, nor is decision-making ability. Therefore, minors may be more likely to engage in dangerous activities as a result of drinking, such as drunk driving. In addition, because the brain is not fully developed until 25, drinking at younger ages could inhibit proper brain development and lead to health issues later on.

ISSUE 2: DO YOU SUPPORT OR OPPOSE THE USE OF GMOS IN RESTAURANT AND GROCERY FOOD?

GMOs, or genetically modified organisms, have been frequently debated throughout the years, particularly in regard to genetically modified food.

Proponents of GMOs argue that genetic engineering might help in feeding the growing population.

Opponents of GMOs argue that almost no research has been conducted on the health consequences of genetic engineering and there is plenty of reason to believe that genetically modifying food can result in devastating health consequences, from severe allergies to cancer.

ISSUE 3: DO YOU SUPPORT OR OPPOSE THE USE OF A RACE QUOTA IN EMPLOYMENT DECISIONS?

Proponents of a racial quota argue that this practice would increase diversity and advance populations that have been historically underrepresented in higher education.

Opponents of a racial quota argue that this practice could be a form of discrimination in and of itself, as it reduces reliance on merit and limits possibilities for those in racial populations who have historically been better represented in the workforce.

Imagine that after reading about each issue, you then report how much you support or oppose the topic on a 6-point Likert scale from -3 (completely oppose) to 3 (completely support). You also report how strongly you feel about your position on a 3-point Likert scale with the following options: 0 (not at all strongly), 1 (somewhat strongly), or 2 (extremely strongly).

Next, imagine that you are matched with a conversation partner on Prolific who STRONGLY DISAGREES with you on one of the issues.

In other words, your conversation partner feels the opposite that you feel. If you oppose, they support; or if you support, they oppose. Both of you also reported feeling relatively strongly about your opinions.

Imagine that you have never met your conversation partner before.

Imagine you will have a conversation with your partner (who disagrees with you) for the next 10 minutes. After the conversation, you and your partner will evaluate your experiences and each other in a survey.

There are three different conditions in this study.
Video-Chatting Condition: If you are assigned to this condition, you and your partner will have your conversation by speaking to each other via a video call.

Speaking Condition: If you are assigned to this condition, you and your partner will have your conversation by speaking to each other via a phone call (without video).

Writing Condition: If you are assigned to this condition, you and your partner will have your conversation by writing to each other via an online, text-chat platform.

For each condition, please assume that you will be able to freely access the software needed to have the video call, phone call, or text-chat without downloading anything onto your computer. In addition, please assume that neither you or your partner will be able to see or save each other's contact information. Finally, please assume that you are currently in a place where you would be able to video-chat, talk, or text-chat with your partner for 10 minutes.

Now, imagine that you are assigned to have a [VIDEO/PHONE/WRITTEN] call with your conversation partner, who strongly disagrees with you.

Remember that you should assume that you'll be able to freely access the video-chat platform, your contact information will not be saved or shared with the other person, and that you will talk to each other for 10 minutes.

Participants report on predictions of conflict (0 = no conflict at all - 6 = a lot of conflict), enjoyment (0 = Will not enjoy at all - 6 = Will very much enjoy), discomfort (0 = very uncomfortable - 6 = Very comfortable), effort (0 = Not at all - 6 = Very), awkwareness (0 = Not at all - 6 = Very), common ground (0 = Not common ground - 6 = a lot of common ground), liking of partner (0 = Not at all - 6 = Very), understanding (0 = Will not understand at all - 6 = Will understand extremely well), partner competence (0 = Not at all - 6 = Very) and attitude congruence (0 = Will strongly disagree - 6 = Will strongly agree).

Before you begin your conversation, imagine that the experimenter gives you a choice between the three different conditions:

Video-Chatting Condition: You and your partner will have your conversation by speaking to each other in a **video call**.

Speaking Condition: You and your partner will have your conversation by speaking to each other in a **phone call (no video)**.

Writing Condition: You and your partner will have your conversation via by writing to each other in a **text chat**.

If you could choose, which of the three conditions would you *want* to be assigned to? Please rank the following three conditions in order from your MOST preferred (#1, at the top) to your LEAST preferred (#3, at the bottom).

[ ] Video-Chatting Condition (Video Call)

[ ] Speaking Condition (Phone Call)

[ ] Writing Condition (Text Chat)

Why did you rank the three conditions the way you did? Please write at least two sentences explaining your reasons.

[open text box]

SUPPLEMENTARY REFERENCES

1. University of California, Berkeley, Office of the Vice Chancellor for Equity & Inclusion. Diversity snapshot: Fall 2013 (University of California, Berkeley, 2013); https://diversity.berkeley.edu/sites/default/files/diversity-snapshot-web-final.pdf
2. Imai, K., Keele, L., & Tingley, D. A general approach to causal mediation analysis. *Psychological methods*, 15(4), 309. (2010).
3. Danescu-Niculescu-Mizil, C., Sudhof, M., Jurafsky, D., Leskovec, J., & Potts, C. A computational approach to politeness with application to social factors. arXiv preprint arXiv:1306.6078. (2013).
4. McFee, B., Raffel, C., Liang, D., Ellis, D. P., McVicar, M., Battenberg, E., & Nieto, O. librosa: Audio and music signal analysis in python. *SciPy*, 2015, 18-24. (2015).
5. Boersma, P., & Weenink, D. Praat: doing phonetics by computer. 2009. *Computer program available at http://www. praat. Org*. (2005).
6. Mauch, M., & Dixon, S. pYIN: A fundamental frequency estimator using probabilistic threshold distributions. In *2014 ieee international conference on acoustics, speech and signal processing (icassp)* (pp. 659-663). IEEE. (2014).
7. Ellis, D. P. Beat tracking by dynamic programming. Journal of New Music Research, 36(1), 51-60. (2007).
8. Smith, J. O., Spectral Audio Signal Processing. In *Center for Computer Research in Music and Acoustics (CCRMA)*, Stanford Universityhttps://ccrma.stanford.edu/~jos/sasp/Sinusoidal_Peak_Interpolation.html. (2022).
9. Gouyon, F., Pachet, F., & Delerue, O. On the use of zero-crossing rate for an application of classification of percussive sounds. In *Proceedings of the COST G-6 conference on Digital Audio Effects (DAFX-00)*, Verona, Italy (Vol. 5, p. 16). (2000).

10. Klapuri, A., & Davy, M. (Eds.). Signal processing methods for music transcription. (2007).

11. Jiang, D. N., Lu, L., Zhang, H. J., Tao, J. H., & Cai, L. H. Music type classification by spectral contrast feature. In *Proceedings. IEEE international conference on multimedia and expo* (Vol. 1, pp. 113-116). IEEE. (2002).
