# Supplementary material for: Spoken disagreement is more constructive than written disagreement
Source: Nat Commun. 2026 Apr 27;17:5792. doi: 10.1038/s41467-026-71669-5 (PMC13332060; doi:10.1038/s41467-026-71669-5)
Supplement: Supplementary file 2 — Reporting Summary [file 41467_2026_71669_MOESM2_ESM.pdf]

nature portfolio

Corresponding author(s): Burint Bevis

Last updated by author(s): Mar 12, 2026

Reporting Summary

Nature Portfolio wishes to improve the reproducibility of the work that we publish. This form provides structure for consistency and transparency in reporting. For further information on Nature Portfolio policies, see our Editorial Policies and the Editorial Policy Checklist.

Statistics

For all statistical analyses, confirm that the following items are present in the figure legend, table legend, main text, or Methods section.

n/a	Confirmed	
☐	☒	The exact sample size (*n*) for each experimental group/condition, given as a discrete number and unit of measurement
☐	☒	A statement on whether measurements were taken from distinct samples or whether the same sample was measured repeatedly
☐	☒	The statistical test(s) used AND whether they are one- or two-sided<br>*Only common tests should be described solely by name; describe more complex techniques in the Methods section.*
☐	☒	A description of all covariates tested
☐	☒	A description of any assumptions or corrections, such as tests of normality and adjustment for multiple comparisons
☐	☒	A full description of the statistical parameters including central tendency (e.g. means) or other basic estimates (e.g. regression coefficient) AND variation (e.g. standard deviation) or associated estimates of uncertainty (e.g. confidence intervals)
☐	☒	For null hypothesis testing, the test statistic (e.g. *F*, *t*, *r*) with confidence intervals, effect sizes, degrees of freedom and *P* value noted<br>*Give P values as exact values whenever suitable.*
☒	☐	For Bayesian analysis, information on the choice of priors and Markov chain Monte Carlo settings
☐	☒	For hierarchical and complex designs, identification of the appropriate level for tests and full reporting of outcomes
☐	☒	Estimates of effect sizes (e.g. Cohen's *d*, Pearson's *r*), indicating how they were calculated

Our web collection on statistics for biologists contains articles on many of the points above.

Software and code

Policy information about availability of computer code

Data collection	All survery data were collected via Qualtrics and are available at: https://doi.org/10.17605/OSF.IO/C3ZGY Conversation data was recorded using skype, and audio data was transcribed by research assistants
Data analysis	Data analyses were conducted in R and can be found at: https://doi.org/10.17605/OSF.IO/C3ZGY

For manuscripts utilizing custom algorithms or software that are central to the research but not yet described in published literature, software must be made available to editors and reviewers. We strongly encourage code deposition in a community repository (e.g. GitHub). See the Nature Portfolio guidelines for submitting code & software for further information.

Data

Policy information about availability of data

All manuscripts must include a data availability statement. This statement should provide the following information, where applicable:

- Accession codes, unique identifiers, or web links for publicly available datasets
- A description of any restrictions on data availability
- For clinical datasets or third party data, please ensure that the statement adheres to our policy

Anonymized data are available at: https://doi.org/10.17605/OSF.IO/C3ZGY

Research involving human participants, their data, or biological material

Policy information about studies with human participants or human data. See also policy information about sex, gender (identity/presentation), and sexual orientation and race, ethnicity and racism.

Reporting on sex and gender	Gender was tested as a potential moderator and the results reported in Supplementary Information E
Reporting on race, ethnicity, or other socially relevant groupings	We did not report any findings on race or ethnicity, but political ideology was tested as a potential moderator. The results are reported in Supplementary Information E
Population characteristics	Demographic data including gender, age, political orientation were collected at the end of each study via Qualtrics.
Recruitment	Studies 1 & 2: participants recruited from the lab participant pool of UC Berekeley. In Study 3, participants were recruited across
Ethics oversight	Ethics approval letters can be found at: https://doi.org/10.17605/OSF.IO/C3ZGY

Note that full information on the approval of the study protocol must also be provided in the manuscript.

Field-specific reporting

Please select the one below that is the best fit for your research. If you are not sure, read the appropriate sections before making your selection.

☐ Life sciences ☒ Behavioural & social sciences ☐ Ecological, evolutionary & environmental sciences

For a reference copy of the document with all sections, see nature.com/documents/nr-reporting-summary-flat.pdf

Behavioural & social sciences study design

All studies must disclose on these points even when the disclosure is negative.

Study description	All studies were quantitative, between-subject experiments. In Studies 1, 2 and 3, participants engaged in a conversation with a conversation partner on a topic either through written
Research sample	We aimed for approx. 50 participants in each experimental condition.
Sampling strategy	1,576 participants and 1,432 observers were recruited across eight nine studies
Data collection	Written conversations were downloaded directly and spoken conversations recorded and transcribed. All participants provided ratings on a range of conversation outcomes and impressions
Timing	Study 1: April - May 2018; Study 2A: Sept - Oct 2017; Study 2B: July - Sept 2018; Study 2C: Feb - May 2019; Study 3: Jan - March 2019; Study 4: March 2018; Lay theory: Feb
Data exclusions	Supplementary Table S1 lists exclusion criteria and number of participants excluded at different stages of Studies 1, 2 and 3. We report pre-treatment exclusions for all studies.
Non-participation	Those who began the conversation but did not finish the study were excluded. We report post-treatment attrition in supplementatry Table S1. Where it was not possible to save the
Randomization	Participants were randomly assigned to experimental conditions
