# Supplementary material for: Spoken disagreement is more constructive than written disagreement
Source: Nat Commun. 2026 Apr 27;17:5792. doi: 10.1038/s41467-026-71669-5 (PMC13332060; doi:10.1038/s41467-026-71669-5)
Supplement: Supplementary file 3 — Peer Review file [file 41467_2026_71669_MOESM3_ESM.pdf]

nature portfolio

Peer Review File

Spoken Disagreement Is More Constructive Than Written Disagreement

Corresponding Author: Mr Burint Bevis

This file contains all reviewer reports in order by version, followed by all author rebuttals in order by version.

Version 0:

Reviewer comments:

Reviewer #1

(Remarks to the Author)
Key Results:
This paper presents convincing evidence that spoken conversations are more effective than written conversations in constructive disagreement, eliciting higher understand, lower experienced conflict, more favorable impressions of the other speaker and more "attitude alignment" compared to written conversations. However, people currently do not realize this and incorrectly believe that spoken conversations will be less useful than written ones in constructive disagreement. The authors present results from a number of different studies to measure which aspects of conversations make these conversations more or less successful. They also collected information for two of their studies on personalities, age and gender to see how these influenced their results but found nothing significant.

Validity:
The validity of the methods used to test whether speaking or writing was more successful in conversations is described in very good detail and all seems correctly done. Note that not all results (e.g. perceived understanding and conflict) did prove statistically significant for these tests.

Significance:
Data and methodology: Your assessment of the validity of the approach, the quality of the data, and the quality of representation. We ask reviewers to assess all data, including those provided as
supplementary information. If any aspect of the data is outside the scope of your expertise, please note this in your report or in the comments to the editor. We may, on a case-by-case basis, ask reviewers to check code provided by the authors (see this Nature editorial for more information).

The data collection was done very well and used many participants for many different studies. In Study 4 the authors examined how a msg was "consumed" due to the speaker's "vocal tone" (pitch) or "pacing" (speaking rate) which they compare with how the message was produced – which words were selected. They recruited new participants to read and listen to the earlier data produced and rate them. The transcribed the spoken data so that the written and spoken versions could be compared. The participants then rated the conversations measuring perceived competence, humanisation, and liking. The then used a politeness R package to identify text features from what was said -- 39 theory-based features that isolate structural and stylistic aspects of linguistic content.

This seems like a rather limited way of identifying what makes speech more successful. Why not simply use other packages which can identify aspects of speech such as pitch, intensity, speaking rate, jitter, shimmer, Harmonics to Noise Ratio, what intonational phrase is being produced? These can easily be extracted Praat, OpenSmile, parselmouth or Scikit-learn. These would be much more useful in identifying major differences within the speech data – which speech is more successful and why?

Analytical approach:

The statistical tests all seem well done.

Suggested improvements: Your suggestions for additional experiments or data that could help strengthen the work and

make it suitable for publication in the journal. Suggestions should be limited to the present scope of the manuscript; that is, they should only include what can be reasonably addressed in a revision and exclude what would significantly change the scope of the work. The editor will assess all the suggestions received and provide additional guidance to the authors.

I am not sure the authors would be able to perform the different forms of speech analysis suggested above for this paper, although if they do have connections with persons who do, including student researchers, it could add an important addition to a paper focused on the difference between speech and text.

Clarity and context:
This seems good.

References:
They seem fine for what is presented in the paper. However there are many other references the authors could use to increase their interest in speech processing.

(Remarks on code availability)
It seems fine.

Reviewer #2

(Remarks to the Author)
The paper under review examines whether the medium of a conversation influences the outcomes of the conversation between disagreeing conversation partners. A series of experiments shows that spoken conversations increase perceived understanding and reduce perceived conflict compared to written conversations. Nonetheless, people prefer written conversations over spoken conversations.

In my evaluation, the paper has potential to make an innovative and generative contribution to the literature. The research question is significant and not easy to study due to the challenges of conducting live conversation experiments. Although recent research has begun to study conversations between disagreeing conversation partners experimentally, the current research experimentally manipulates conversational features that go beyond prior work. I appreciate the clever designs to keep various factors of conversations constant to identify what is driving (or not driving) the effects. Another strength of the paper is the multi-method approach, combining lab and field experiments as well as an online prediction study and NLP analyses. The data and code are made easily available.

Nonetheless, the paper has several important limitations that undermine the scientific value of the paper in its current form.

1. Lack of power analyses and interpretability of null effects

A significant contribution of the paper is the clever manipulation of conversational features. Unfortunately, this contribution is undermined because the current analyses do not reveal whether the null (interaction) effects of these manipulations are meaningful. The paper does not include power analyses, so that readers cannot distinguish whether the null effects indicate that the actual effects are likely null or whether the studies were underpowered.

This is particularly relevant for the interaction effects in Studies 2A-C. The authors write on p. 20: “Further, none of the conversational structures significantly interacted with the effect of
conversation medium on perceived understanding or conflict, suggesting that although speaking may tend to be a more interactive and synchronous medium than writing, manipulating those aspects was not enough to reduce the effect of medium on perceived understanding or conflict.”

A non-significant interaction cannot be interpreted as meaningful without power analyses (or other statistical methods such as Bayesian statistics). To address this point, the authors could conduct sensitivity power analyses to describe what effect they could identify with sufficient power and be clear for what kind of effects they were underpowered. Due to the complexity of their design, the authors will need to assume several parameters to conduct power analysis. I’d recommend running sensitivity power analysis that use several estimates from the studies they conducted as input, such as the sample size, means, standard deviations and correlations between variables relevant for the data analysis. Using these estimates, they can simulate what effects they were able to find with 95% power.

2. Lack of accounting for interdependency in analysis strategy

Due to the interactivity, the analyses are more complicated than standard experimental analyses. Therefore, I would suggest that the authors spent significantly more space on explaining their analytical choices. I have the following questions

First, how does the analysis in Study 1 account for the fact that treatment assignment was at the session level? My intuition is that this should result in less power than treatment assignment at the dyad level. Yet, the analysis strategy does not account for assignment at the session level.

Second, how does the analysis in Study 3 account for the fact that treatments involved multiple conversations? The authors write they cluster standard errors at the individual level. But do they also account for the dependency between partners of the

same conversation?

3. No causal evidence for the role of conversational receptiveness.

The paper emphasizes the role of conversational receptiveness for driving the effects. For example, the authors write on p. 44-45: “Our experiments also demonstrate the power of linguistic choices in the perception of conflict. At least one of the reasons why communicators experienced less conflict, and felt greater understanding, when speaking than writing is because they used more receptive language. The increase in conversational receptiveness can be specifically attributed to greater use of subjectivity phrases and fewer uses of negative emotion phrases in speech than writing. Notably, we also found that conversational receptiveness is more effective in writing than speech.”

Because the authors did not demonstrate the causal effects of conversational receptiveness, I see this a significant overstatement. An experiment that manipulates of conversational receptiveness to test its main effect and/or interaction effect with medium would be fascinating. But unless the authors provide such evidence, the language on the role of conversational receptiveness would need to be toned down significantly.

4. Confounds in Study 3

Study 3 – which is described as a field experiment that allows generalizing beyond lab experiments – features several design decisions that complicate the interpretation of the findings. First, Study 3 featured multiple conversations. As a result, responses to the second – fifth conversations cannot simply be ascribed to differences in conversation medium but are also the product of the different conversations participants had in prior rounds. This could be addressed be restricting the analysis to only the first round.

Second, an important distinction between the spoken and the written conversation in Study 3 is that the written conversation is very unnatural. Because participants sat across each other, speaking is much more natural than chatting. This confound cannot be addressed without additional data collection. It is reassuring that the results are consistent across the conversations. But the boost that a paper should get for a field replications is limited by this design feature.

Third, another confound is that that spoken and written conversations differed in length (although only at the ASU site) is. One may argue that the lack of significant interactions in Study 2b means that length does not matter. However, as I write in my first point, I am not convinced by that result.

Fourth, some participants had four and some had five conversations in Study 3. It is unclear why the experience was not identical across all participants. This could be addressed by adding an explanation and testing for differences across conditions. Furthermore, it could be cleanly addressed by restricting the analysis to the first round of conversations.

5. Unclear conceptualization of constructive disagreement.

The authors define (or operationalize?) constructive disagreement as perceived understanding and conflict. However, it is unclear whether this definition is widely used or deviates from other definitions/operationalizations in the literature.

Furthermore, in the abstract and parts of the introduction, the conceptual differences between perceived understanding and perceived conflict and two other outcomes (impressions of conversation partner and attitude alignment) are unclear. The paper could be improved by clarifying that these are different outcomes. In fact, attitude alignment is fundamentally different from constructive disagreement because it implies a reduction in disagreement.

The paper could be further improved by conceptually distinguishing conflict from disagreement. Conflict is sometimes used as a synonym for disagreement but the items the authors use suggest that conflict is meant in a more emotional sense.

6. Differences between lay study and experiments.

The lay study is a nice addition to show that the experimental findings are surprising. Ideally, these studies would be as similar as possible to allow clean comparisons. However, there are at least two important differences between the lay study and experimental findings. First, the lay study uses a within-subjects manipulation whereas the experiments featuring actual conversations use between-subjects manipulations. Second, the sampling populations are quite different. These limitations should be acknowledged as potential alternative explanations for the observed differences.

7. Lack of clarity which studies and analyses were preregistered.

The paper is unclear about which studies are preregistered. Statements such as “predetermined exclusion criteria” in Study 1 are confusing. Readers may understand this as “preregistered” but (i) Study 1 was not preregistered and (ii) the exclusion criteria were not described in the preregistration. This could be improved by specifying in the text which studies were preregistered and how the studies deviated from the preregistration.

8. Unavailability of OSF materials through some links

The links in the data and code availability sections does not provide access to the OSF project. However, the link in the transparency statement does provide access. This could be improved by providing access through all links.

Thank you for the opportunity to review this paper. I wish the authors all the best and hope my feedback is helpful to improve the paper.

Jan G. Voelkel

(Remarks on code availability)
The link https://osf.io/c3zgy/ (which is also listed in the data and code availability sections) does not provide access to the OSF project. However, the link in the transparency statement does provide access.

I looked up the code for some analyses decisions which informed my review but I did not try to reproduce the findings.

Reviewer #3

(Remarks to the Author)
The present paper investigates the effect of written versus spoken conversation on constructive agreement (less conflict and more understanding) in several experiments. The main finding is that spoken conversation leads to more constructive agreement. This is mediated by 'receptiveness' of the language. However, receptiveness has a stronger effect on constructive agreement in written than spoken conversations. In contrast to these findings, lay theory suggest that written conversations will be more constructive than spoken ones.
In my view, this paper addresses an important research question that is of interest to a broad audience and relatively novel. The authors present a large amount of evidence from several experiments, supporting their main claim. In addition, they report on several interesting extra experiments that deepen understanding and relevance regarding the found effects. I have several questions and remarks on the paper, which I list below, but most are relatively minor and can be easily addressed by the authors I think. There are also three somewhat larger issues that I believe do need to be addressed before publication is warranted.

Major remarks
1. I believe the introduction of the paper could benefit from including some other relevant studies. First, specifically two papers from Roos et al. (2020a&b) appear very relevant to the present study. These papers also investigate differences between online (written) and face-to-face discussions about controversial topics. Apart from some methodological differences (e.g., groups of 3/4) the studies' outcome measures are also somewhat different from the present study, but they do appear related and very relevant (i.e., responsiveness, ambiguity, disinhibition, polarization, and solidarity). I would suggest the authors discuss these papers and indicate what their study adds to the insights of those results (and potentially other papers from the same research group). Somewhat less relevant is a paper by Sillars & Zorn (2020) showing that negative email is often interpreted more negatively than it was intended, which also goes in a similar direction as the present results. I may not be aware of all relevant studies in the field, so the authors may additionally want to do a more thorough literature search. Second, the discrepancy the authors report between lay theory and research findings may be related to other findings in psychology, such as the finding in Kruger et al. (2005; already cited by the authors but not in this context). This study showed that we think we can communicate better over email than we actually can, which may be related to the present finding that we think our textual communication is more constructive than our verbal communication, while this is not the case.
2. I also have several comments and questions related to the variable of 'receptiveness'. This term was slightly confusing to me at first because I expected it to be more about interactional responsiveness (as Roos et al., 2020a use the term responsiveness), that is, whether the communicators orient towards each other and appear to listen to each other in their speech. This may also relate to the use of interjections for example, which are very relevant interactional devices in conversation (see e.g., Dingemanse, 2024). It appears that the 'receptiveness' variable does not fully capture these aspects that may also be very relevant (acknowledgements and agreements appear to come closest, but they may be more explicit, judging from the examples). It is interesting in this regard that, in study 4, transcribed speech leads to more conflict and less understanding. Looking at the transcript instructions, precisely these interjections may have been taken out in the written versions. Such responsiveness may be more expected to occur in conversations with more interactivity. Therefore, it may be interesting to analyze study 2A to see if there is more receptiveness in the interactive condition. Last, it is interesting that, as seen from Figure 5, not all presumed elements of receptiveness actually go in the expected direction. Especially more ambiguity (Adverb. Limiter) is found in speaking (which was incidentally also found by Roos et al., 2020a) whereas this is seen as negative for receptiveness. This begs the question whether receptiveness is the right construct to focus on in the present study, or whether it makes more sense to focus on more specific constructs or variables to see how they can explain the difference between spoken and written communication, including perhaps something that is more related to 'responsiveness'. At least I would like to see the authors discuss these issues in the General Discussion.
3. I do not understand the description of the mediation analysis (7.2.4). It is stated that the direct and total effects are significant, but the authors should be interested in the *indirect* effect, which would be the (part of the) effect of medium on understanding that is mediated by receptiveness. They do not mention any indirect effects. As it is stated now, it seems the total effect is .35 and the direct effect (not mediated by Receptiveness) is .31. That would imply that the indirect effect is very small (.04) and unlikely to be significant. It would be important to check exactly which effects are found here.

Minor remarks
1. At the start of the Introduction (page 3) it is not immediately clear which type of media are the main focus of the paper (namely, text vs. speech). It would be helpful to spell that out early.
2. I missed some methodological details that I believe would help the reader if they were present in the main text (and not

only in an appendix):
- Study 1, page 8: it is unclear that pair members are strangers to each other
- Study 2A: What is the structure of the conversations here: is there interaction throughout the conversation or is there again allocated speaking time as in study 1?
- Study 2A: (page 15, first line) “both people prepared their statements at the same time”. It is not clear what this exactly means in speech and writing. I assume that in writing, people type their response at the same time, whereas in speech they think about what they want to say at the same time (but say it after each other)? In the latter case it is still possible for the second speaker to react to the first, which makes the two situations not really equivalent.
- Study 2C (3.3.2): the structure is not totally clear to me here: “in round one, Person A (randomly assigned) was given one minute to speak or write, while Person B had one minute to listen or read”. I suppose this is sequential (but the word while is confusing here)? If it is, doesn’t that mean that the conversation time in this condition is actually 12 minutes, because it includes 6 minutes of ‘waiting time’?
- Study 3, page 23: “recordings were attempted at one location but the background noise was too disruptive”. How was this for the participants during the study? Could they hear each other well and were they not distracted by the background conversations?
3. Paragraph 4.2.2: It seems that the interaction effect between attitude distance and medium is only performed for agreeing dyads. I wonder why it was not performed for the whole sample (agreeing and disagreeing dyads, which can be seen as a continuum), because the variance of attitude distance would be much larger in that case.
4. Page 27: The effect on attitude shifts is very small and only marginally significant. What does that mean for the relevance of the findings in general? Wouldn’t one expect that constructive disagreement is more effective in bringing conversation partners closer together in terms of their opinion? I think this would be relevant to discuss in the General Discussion (even if it was just measured in one study, it could be suggested for future research).
5. The authors use the term ‘linguistic content’ not only for the actual semantic content of what is said, but also for how (formal, polite, etc.) something is said. It might be helpful for some readers to clarify this early on that the authors also include this aspect under ‘linguistic content’.
6. Page 40. Even though the effect of medium on receptiveness is still significant when controlling for word count, sentiment, and politeness, the effect does appear to be smaller than without these controls. It may be interesting to analyze whether any of these control variables mediate the effect to see if they can still partly explain the effect (though not fully as this analysis suggests).
7. Study 5: Effect of preference. It might be relevant to discuss the possibility that preference is related to other factors sch as awkwardness and effort, especially when talking to a stranger. Since there is no comparison with a non-conflict situation, it is also not clear whether this effect is related to the conflict or not.
8. Page 45: Discussion of moderating effect of medium: An alternative explanation here might be that precisely BECAUSE receptiveness is relatively infrequent in written conversation, it is more meaningful when it does occur, and therefore the effect is larger.
9. Page 45: Discussion of Study 1: no difference between video chat and spoken conversation: This finding may also be related to the fact that video chat is different from face-to-face conversation since it does not allow for eye contact for example.
10. Page 46: Discussion of production and consumption effects: I think it should be noted here that the finding that “the same words were interpreted differently when heard versus read” only holds for originally spoken materials. As mentioned above, this effect may be related to the transcription instructions since some words (i.e., interjections) that may be important were removed.
11. Page 49: The discussion here about participants’ engagement may also be relevant for experiment 5 (at least the preference measure). It is likely that the participants that were asked to imagine being part of another experiment had different goals and engagement: they were likely less (intrinsically) motivated to solve the conflict, so the extra effort they believed the speech condition to entail may not have been worth it for them.
12. Paragraph 9.3: Limitations: Another limitation that could be mentioned is that all studies (as far as I can tell) involved conversations between strangers, not friends, where these effects may be different (this is especially imaginable for the preference effect in study 5, but may be the case for other results as well).

Textual remarks
- I am not a native speaker of English, but I had not heard of the word ‘vitriol’ before (page 3), and had to look it up. The authors may consider using another word here or explain what it means.
- Page 4: ‘speech can accurately convey an experience or intention even without semantic content’, would be nice for the reader to know how this information can then be conveyed (e.g., by prosody?).
- Page 37: The description of Figure 5 (“The more a feature is used in writing than speaking, the higher it is on the y-axis”) appears to be opposite to the actual figure.

References
- Dingemanse, M. (2024). Interjections at the Heart of Language. Annual Review of Linguistics, 10(1), 257-277.
- Roos, C. A., Koudenburg, N., & Postmes, T. (2020a). Online social regulation: When everyday diplomatic skills for harmonious disagreement break down. Journal of Computer-Mediated Communication, 25(6), 382-401.
- Roos, C. A., Postmes, T., & Koudenburg, N. (2020b). The microdynamics of social regulation: Comparing the navigation of disagreements in text-based online and face-to-face discussions. Group Processes & Intergroup Relations, 23(6), 902-917.
- Sillars, A., & Zorn, T. E. (2021). Hypernegative interpretation of negatively perceived email at work. Management Communication Quarterly, 35(2), 171-200.

(Remarks on code availability)

Version 1:

Reviewer comments:

Reviewer #1

(Remarks to the Author)
The authors have taken my suggestions to include audio data in their research as well as text and included their findings in the paper. Their results are quite interesting and add to the overall importance of the paper

(Remarks on code availability)
I did not assess the code.

Reviewer #2

(Remarks to the Author)
The revised paper could make an innovative and generative contribution to the literature. The central finding – that speaking, compared to writing, increases perceived understanding and reduces perceived conflict – is compelling and nicely demonstrated across several studies. The contrast to the lay theory study adds further value, and the limitations of this comparison are appropriately acknowledged. That alone would make for a strong and interesting paper.

However, the paper also emphasizes other claims that are less convincingly supported. In particular, the concerns I raised in the previous round around interpretations of non-significant results, causal interpretations of correlational findings, and transparency in analysis decisions still appear to persist. I believe the paper would be stronger if it were shorter, focused more narrowly on the central finding (the effect of medium on perceived understanding and conflict), and more rigorous in evaluating and communicating the evidentiary basis for its claims.

1. Interpretation of non-significant effects as evidence for the absence of an effect.

The addition of sensitivity power analyses for Studies 2A–C is welcome.

However, the manuscript still draws overly strong conclusions from non-significant results. For example: “Further, none of the conversational structures had a statistically significant interaction with the effect of the conversation medium on perceived understanding or conflict, suggesting the effect of medium is not entirely due to speech being more interactive (2A), producing more words (2B), or being more synchronous (2C) than writing. However, these results must be interpreted with caution as we were not sufficiently powered to detect small interaction effects.”

This statement is not supported by the sensitivity power analyses as reported. First, it is difficult to compare the power analyses and the findings reported in the text because the power analyses use f^2 and R^2 metrics, while the main text discusses beta coefficients. In my experience, interaction effects accounting for 6%–7% of the variance would require very large interaction effects. To meaningfully speak to what effects can be ruled out, power analyses should estimate the minimum detectable beta coefficients with 95% power. This could be done by running simulations using the parameters of the study and then varying the effect size. It would be appreciated if the code for the power analyses could be shared.

Based on the provided information, I am not convinced Studies 2A-C were powered to detect even interaction effects that would cut the effect of medium in half. Without providing further evidence, conclusions about the moderating roles of synchronicity, duration, and interactivity seem premature.

In other sections of the paper, non-significant effects are presenting without interpretation and corresponding sensitivity power analyses. For example, in the paragraph starting with the sentence: “Participants had multiple conversations in this study, so we tested whether the treatment effect varied over time.” Test statistics are sometimes omitted altogether (e.g., “We also find no moderation of the treatment effect across the different locations”).

All non-significant effects need to be put in context so that the reader can distinguish between informative null effects and underpowered tests from which little can be inferred.

2. Interpretation of correlational findings as evidence for causal relationships.

Causal language is still used when discussing findings that are correlational, especially regarding receptiveness. For example:

“the (b) path confirms that conversational receptiveness increases perceived understanding.”

“Our experiments show that spoken media (e.g., using the phone, video-chatting, in-person conversation) induce greater use of receptive language than written media (e.g., text-messaging, emailing), but also that when used in speech, receptive language is less effective.”

The causal role of receptiveness is not tested in the paper, as it is not experimentally manipulated. I raised this point in my previous review, and while the authors acknowledged the concern—writing in their response that they had softened causal claims—this change does not seem to be fully reflected in the manuscript. The discussion still implies a direct causal relationship.

It is also worth noting that receptiveness only mediates approximately 10% of the total effect in the correlational mediation analysis.

3. Transparency

The paper includes many analyses, but it is unclear which were preregistered. The authors note:

"To reduce researcher degrees of freedom even in studies that were not preregistered, we apply consistent analyses across studies…"

However, analysis strategies appear less consistent than described. For example, Study 3 includes agreeing dyads, whereas other studies exclude them. The results are reported separately for disagreeing and agreeing dyads, and then again in a "prediction accuracy" section, which appears to pool both. Additionally, in the earlier analyses, a control variable (whether participants knew each other) is included, but it is not included in the prediction accuracy analyses.

I do not assume any ill intent, but these inconsistencies make it difficult to evaluate the robustness of the findings.

The transparency statement indicates that all preregistrations are posted on OSF. However, I was only able to access a preregistration for the lay theory study via the provided link.

4. Attrition

Table S2 is helpful and appreciated. That said, there are at times large deviations from random assignment that raise concerns about differential attrition. For example:

In Study 1, 38% of video-chat participants versus 13% of speaking participants were excluded due to "pair-level exclusion."

In Study 1, 15% of speaking participants versus 0% in writing were excluded due to recording issues.

In Study 3 (Berkeley), 36% of dyads were assigned to speaking and 64% to writing.

5. Inconsistencies/errors

The authors use different thresholds for interpretating p-values.
o On page 13: a p-value of .043 is described as marginally significant.
o On page 14: The same result is described as non-significant: "We found no significant difference between the two speaking conditions - audiovisual or audio-only - which was somewhat surprising, given the additional visual information available in one group."
o On page 49, two p-values of .071 and .042 are evidence of slightly more understanding without a statement about significance.

On page 20, effects are described as being in a consistent direction although the effect for Study 2A is in the opposite direction: "The effects on perceived conflict were in a consistent direction but not always statistically significant. Average perceived conflict was lower when speaking (2A: M = 1.31, SD = 1.11; 2B: M = 0.54, SD = 1.15; 2C: M = 0.58, SD = 1.03) than writing (2A: M = 1.22, SD = 1.21; 2B: M = 0.67, SD = 1.25; 2C: M = 1.05, SD = 1.30)."

On page 33, the interaction with admission quotas vs. drinking age was not tested even though that will have the largest effect size.

Thanks for the opportunity to read and review this manuscript. I hope these comments are helpful in strengthening the paper.

(Remarks on code availability)

Reviewer #3

(Remarks to the Author)
I would like to thank the authors for their effort to change the manuscript and respond to my comments from the first round. I am happy with many of the changes in the manuscript and convinced by most of the arguments in the response letter. I have one remaining larger issue (point 1 below) that I believe the authors should handle more carefully, namely the incorporation of the earlier studies that I mentioned in my first review. Next to that, I have a few other more minor suggestions that I believe would improve the paper even more.

1. I appreciate that the authors cite the Roos et al. (2020a, 2020b) studies in the revised version of their manuscript. However, they do this very briefly in the Introduction and I do not agree with the statement made there about these studies, namely that they 'do not capture conversational behavior' (page 4). The (spoken and written) conversations in these studies were actually recorded and were coded by human coders for conversational behavior, namely responsiveness and ambiguity. I agree with the authors that there are other differences with the present manuscript (as they mention in their response letter), such as the smaller number of participants and the explicit creation/measurement of disagreement, which may be mentioned in the introduction (although I do not agree that their outcome measures such as perceived solidarity and polarization can be equated with 'fun and clarity'). Furthermore, I believe the studies deserve some further discussion in either the Introduction or (perhaps preferably) the Discussion, given that some of the outcomes appear quite related to those of the present study, even if perhaps not the exact same measures and concepts are used. I think it would be helpful for communication across different disciplines to acknowledge and discuss research from other disciplines despite difficulties related to different terminology. Although I respect and understand the authors decision to focus on receptiveness in this study, links can be made with concepts from the Roos et al. studies. As a first example, Roos et al. focus on ambiguity (ambivalence, disclaimers, and hedges) as one conversational behavior. Judging from the descriptions of features of receptive language, this may relate to subjectivity, hedges, and adverb limiters (which are defined as 'adverbs that increase ambiguity'). Two of these are found in the present study to be more frequent in speech than text, which is what Roos et al. find for ambiguity as well. Second, outcome measures such as perceived consensus, perceived solidarity, and perceived polarization appear related to the concept of conflict in the present study, which makes it relevant to discuss the similarities in results between the studies.
2. For studies 1 and 2, in the text only the results for the two main outcome variables are described, but in the figures all outcome measures are displayed, which is a bit confusing. Maybe I missed it, but it would be helpful if the authors explicitly state somewhere what they report in the main text (and whether/where the results for other variables are described in an Appendix).
3. On page 12, it is stated that: "Then, they were given three more minutes to jointly discuss, for a total of up to nine minutes." This is a bit unclear, did they discuss for 3 or 9 minutes or minimally 3 and maximally 9 (was it up to them)? Later it is stated that conversations in this study were 9 minutes.
4. The exact speech conditions in study 2 are not always clear. In 2A the speech condition was 'in person', so does that mean face-to-face, without a video connection? And in 2B/2C it was 'using the Skype platform', so does that mean videoconferencing or Skype calling (without video)?
5. There are some minor inconsistencies in the reporting of the results (e.g., no figure for study 3 and means/SDs for some results but not others).
6. It might be interesting for methodological reasons to know if the prediction accuracy of the other's position ('actual understanding') correlates with your measure of perceived understanding in study 3.
7. The greater use of adverb limiters in speaking actually goes against the expectation given that more adverb limiters are related to less receptivity. This counterintuitive finding is not acknowledged nor explained or discussed in the text anywhere. I would appreciate a short discussion of this somewhere.
8. The 'other language measures' (page 46) are not defined and word count was already discussed above so it is unclear why this is repeated here.
9. In the first paragraph of page 52, the authors could again add the potential explanation that receptivity has a larger effect in text because it is more rare there.
10. At the end of page 54, the authors state that "communicators who used more receptive language tended to report greater understanding, less conflict, and higher impressions of each other after their conversations". If I understand it correctly, this result comes from the mediation analysis, however, the details of this analysis were not so explicit. Was this indeed investigated at the individual level: individuals who used more receptive language reported greater understanding? In that case, might it also be interesting to investigate the effect on the other: do the interlocutors of individuals who use more receptive language report greater understanding as well?
11. Figure 2: "The variables are ordered in this plot based on effect size.": I first misunderstood this to mean absolute effect size, maybe this could be changed to: from most negative to most positive effect?
12. In Appendix T some causal explanations appear overstated: "whether aspects of speech influenced our dependent variables" and "role of speech rate in enhancing understanding".

(Remarks on code availability)

Version 2:

Reviewer comments:

Reviewer #2

(Remarks to the Author)
The revised paper makes an innovative and generative contribution to the literature. I appreciate that the authors thoroughly engaged with the reviews. I find the most recent submission significantly improved and I generally support acceptance. However, there remain some issues for which the revisions are suboptimal, and I recommend addressing these prior to publication.

1. Sensitivity (not post hoc) power analyses versus confidence intervals?

There appears to be confusion about my prior comment about interpreting null effects. The authors added confidence

intervals. While confidence intervals are useful to report, they are not a substitute for sensitivity power analyses, which, in my opinion, would improve the paper if featured more prominently in the main text.

Sensitivity power analyses help determine which effect sizes can be ruled out given the design. These differ from post-hoc power analyses; my original comments did not advocate for post-hoc power.

To illustrate the stakes: the detectable interaction effects in Studies 2A–C were extremely large (βs: 1.80, 1.10, 1.25), especially relative to the largest observed main effect (β = .53). Thus, these studies were fundamentally ill-powered to detect realistic interaction effects.

The paper should explicitly state that Studies 2A–C primarily serve a stimuli-sampling function—demonstrating robustness across contexts. However, the absence of statistically significant interactions cannot be interpreted as evidence of null effects. Many readers may mistakenly make this inference, so the manuscript should proactively prevent misinterpretation.

To be clear, I am not arguing the authors should have powered the studies to detect realistic interactions; as Cohen (1988) notes, this is often infeasible. My objection is solely to be transparent that statistical power was insufficient to detect interaction effects.

I was confused by the author's statement in the memo: "In fact, it was not always possible for the minimum detectable interaction effects to be simulated with 95% power. The maximum power we could simulate interaction effects for across each study were (2A: 96%, 2B: 73%, 2C: 89%)."

This statement is unclear, as there is always a minimum detectable effect size associated with any desired power level—even if that effect is very large. It would be helpful for the authors to clarify what constraints prevented them from identifying the minimum detectable effect size.

2. Interpretation of Findings in Study 4

The interpretation currently collapses across outcomes, whereas Figure 4 indicates distinct patterns for perceived understanding vs. perceived conflict. The authors summarize the findings as: "the current study finds an effect of the production medium whereby observers perceive higher understanding and less conflict between communicators who spoke than wrote to one another" and "In particular, spoken statements that were heard by observers (i.e., in the "produced-by-speaking and consumed-by-listening" condition) were deemed to create the most understanding and least conflict."

But looking at Figure 4, the data appear to support different conclusions for the two dependent variables. To accurately represent the results, the manuscript should interpret simple effects, not only main effects and interactions.
• The production medium has a clear effect on perceived understanding; words produced by speaking result in more perceived understanding than words produced by writing. This effect is stronger when the words are produced by listening.
• In contrast, there is no clear overall effect of production medium on perceived conflict. Instead, ONLY when words are produced by speaking AND consumed by listening, there is a reduction in perceived conflict.

Minor points
• Page 16: "Of that set, 1 person (0.2%) could not participate in the study because either they could not be matched with a partner within their session or were not fluent enough in English." The exclusion rationale is ambiguous ("either… or…"). I'd recommend clarifying which applies.
• Page 18: "Of those who participated, 46 (9.5%) were removed from the analysis due to our predetermined exclusion criteria." I'd recommend that the word "predetermined" is cut because the study was not preregistered.
• Page 20: "Our final sample was 414 individuals." The numbers don't match. 489 – 17 – 54 = 418 (not 414).
• Page 52: "Overall, these lay theory data suggest that people expect a disagreement will be more constructive (e.g., lead to more understanding and less conflict) in a written conversation than in a spoken conversation." This is a slightly inaccurate characterization as the difference between speaking condition and writing condition was not statistically significant for perceived understanding.

Thank you for the opportunity to review this revised submission. I believe the paper will make a valuable contribution. I wish the authors all the best as they finalize the manuscript.

(Remarks on code availability)

Reviewer #3

(Remarks to the Author)
The authors have addressed all my concerns satisfactorily.

(Remarks on code availability)

Open Access This Peer Review File is licensed under a Creative Commons Attribution 4.0 International License, which permits use, sharing, adaptation, distribution and reproduction in any medium or format, as long as you give appropriate credit to the original author(s) and the source, provide a link to the Creative Commons license, and indicate if changes were made.
In cases where reviewers are anonymous, credit should be given to 'Anonymous Referee' and the source.
The images or other third party material in this Peer Review File are included in the article's Creative Commons license, unless indicated otherwise in a credit line to the material. If material is not included in the article's Creative Commons license and your intended use is not permitted by statutory regulation or exceeds the permitted use, you will need to obtain permission directly from the copyright holder.
To view a copy of this license, visit https://creativecommons.org/licenses/by/4.0/

REVIEWER COMMENTS

Reviewer #1 (Remarks to the Author):

Thank you for your positive evaluation and constructive comments. We describe how we have addressed the substantive concerns below.

Key Results:
This paper presents convincing evidence that spoken conversations are more effective than written conversations in constructive disagreement, eliciting higher understand, lower experienced conflict, more favorable impressions of the other speaker and more “attitude alignment” compared to written conversations. However, people currently do not realize this and incorrectly believe that spoken conversations will be less useful than written ones in constructive disagreement. The authors present results from a number of different studies to measure which aspects of conversations make these conversations more or less successful. They also collected information for two of their studies on personalities, age and gender to see how these influenced their results but found nothing significant.

Validity:
The validity of the methods used to test whether speaking or writing was more successful in conversations is described in very good detail and all seems correctly done. Note that not all results (e.g. perceived understanding and conflict) did prove statistically significant for these tests.

Significance:

Data and methodology:
The data collection was done very well and used many participants for many different studies. In Study 4 the authors examined how a msg was “consumed” due to the speaker’s “vocal tone” (pitch) or “pacing” (speaking rate) which they compare with how the message was produced – which words were selected. They recruited new participants to read and listen to the earlier data produced and rate them. The transcribed the spoken data so that the written and spoken versions could be compared. The participants then rated the conversations measuring perceived competence, humanisation, and liking. The then used a politeness R package to identify text features from what was said -- 39 theory-based features that isolate structural and stylistic aspects of linguistic content.

This seems like a rather limited way of identifying what makes speech more successful. Why not simply use other packages which can identify aspects of speech such as pitch, intensity, speaking rate, jitter, shimmer, Harmonics to Noise Ratio, what intonational phrase is being produced? These can easily be extracted Praat, OpenSmile, parselmouth or Scikit-learn. These would be much more useful in identifying major differences within the speech data – which speech is more successful and why?

We thank you for this suggestion. The reason we originally chose not to analyze the voice recordings is that our primary research question regards the consequences of spoken versus written mediums, and such analyses cannot be conducted in the written medium. In other words, they cannot provide insight regarding differences between speaking and writing. This is one reason why we relied on text-based linguistic features (e.g., receptiveness), which allow us to compare common features across mediums.

However, we appreciate your broader point that a lot more can be learned from participants' speech, and your ideas for analyzing it are well-taken. Following your recommendations, we used a Python-based approach using the Librosa package (McFee et al., 2015) to extract speech rate, loudness variability, pitch variation, speech crispness, speech brightness, frequency spread, and speech clarity (full definitions provided in Appendix T). We also attempted to extract additional features such as jitter, shimmer, and harmonics-to-noise ratio (HNR) using Praat, but these measures were highly sensitive to background noise and microphone inconsistencies, leading to unreliable extractions across datasets. Unlike Librosa's spectral and temporal audio features, which could be robustly applied to all recordings, jitter and shimmer calculations require precise, high-quality pitch tracking, making them prone to errors in conversational speech. Given these limitations, we prioritized features that could be reliably extracted across all studies to ensure consistency in our analyses.

Our findings (reported in Supplementary Table S25 in Appendix T) show that speech rate is positively correlated with perceived understanding, competence, and enjoyment, and negatively correlated with perceived conflict, suggesting that speech rate should be explored further in future studies.

Of course, these results must be interpreted with great caution. They are correlational, not causal. For example, simply speaking faster is unlikely to cause an increase in understanding; it could be that the pairs who speak more quickly together are doing so as a result of having better understanding. Moreover, the most useful analysis of audio features must also consider how they interact with

linguistic features. For example, emphasis may certainly change the meaning of certain words, but how this affects receptiveness and constructive disagreement depends on what words are being emphasized. In summary, we believe the question of how speech features influence conflict outcomes is a complex and important question that deserves a more thorough treatment in a follow-up paper.

McFee, B., Raffel, C., Liang, D., Ellis, D. P., McVicar, M., Battenberg, E., & Nieto, O. librosa: Audio and music signal analysis in python. SciPy, 2015, 18-24. (2015).

Analytical approach:

The statistical tests all seem well done.

We appreciate the kind words!

Suggested improvements:

I am not sure the authors would be able to perform the different forms of speech analysis suggested above for this paper, although if they do have connections with persons who do, including student researchers, it could add an important addition to a paper focused on the difference between speech and text.

We are grateful that your suggestion has provided us with a useful in-house learning experience. Luckily, we were able to learn how to conduct the analyses ourselves.

Clarity and context:
This seems good.

References:
They seem fine for what is presented in the paper. However there are many other references the authors could use to increase their interest in speech processing.

We appreciate the suggestion but our investigation above has led us to believe that speech processing should be the focus of a follow-up paper, rather than adding to the current paper, which is squarely focused on the decision of medium. Because speech processing is impossible in one experimental condition (i.e., writing), it cannot help our understanding of the difference between conditions. Still, we have added some citations regarding speech processing (Boersma & Weenink, 2005; Ellis, 2007; Gouyon et al., 2000; Klapuri & Davy, 2007;

McFee et al., 2015; Mauch & Dixon, 2014; Smith, 2022) to Appendix T, wherein we conduct the analyses. That being said, if there are specific papers you have in mind, we would be happy to read them and add them to our literature review.

References:

Boersma, P., & Weenink, D. Praat: doing phonetics by computer. 2009. Computer program available at http://www. praat. Org. (2005).

Ellis, D. P. Beat tracking by dynamic programming. Journal of New Music Research, 36(1), 51-60. (2007).

Gouyon, F., Pachet, F., & Delerue, O. On the use of zero-crossing rate for an application of classification of percussive sounds. In Proceedings of the COST G-6 conference on Digital Audio Effects (DAFX-00), Verona, Italy (Vol. 5, p. 16). (2000).

Klapuri, A., & Davy, M. (Eds.). Signal processing methods for music transcription. (2007).

Mauch, M., & Dixon, S. pYIN: A fundamental frequency estimator using probabilistic threshold distributions. In 2014 ieee international conference on acoustics, speech and signal processing (icassp) (pp. 659-663). IEEE. (2014).

McFee, B., Raffel, C., Liang, D., Ellis, D. P., McVicar, M., Battenberg, E., & Nieto, O. librosa: Audio and music signal analysis in python. SciPy, 2015, 18-24. (2015).

Smith, J. O., Spectral Audio Signal Processing. In Center for Computer Research in Music and Acoustics (CCRMA), Stanford University. https://ccrma.stanford.edu/~jos/sasp/Sinusoidal_Peak_Interpolation.html. (2022).

Jiang, D. N., Lu, L., Zhang, H. J., Tao, J. H., & Cai, L. H. Music type classification by spectral contrast feature. In Proceedings. IEEE international conference on multimedia and expo (Vol. 1, pp. 113-116). IEEE. (2002).

Reviewer #1 (Remarks on code availability):

It seems fine.

Reviewer #2 (Remarks to the Author):

The paper under review examines whether the medium of a conversation influences the outcomes of the conversation between disagreeing conversation partners. A series of experiments shows that spoken conversations increase perceived understanding and reduce perceived conflict compared to written conversations. Nonetheless, people prefer written conversations over spoken conversations.

In my evaluation, the paper has potential to make an innovative and generative contribution to the literature. The research question is significant and not easy to study due to the challenges of conducting live conversation experiments. Although recent research has begun to study conversations between disagreeing conversation partners experimentally, the current research experimentally manipulates conversational features that go beyond prior work. I appreciate the clever designs to keep various factors of conversations constant to identify what is driving (or not driving) the effects. Another strength of the paper is the multi-method approach, combining lab and field experiments as well as an online prediction study and NLP analyses. The data and code are made easily available.

Thank you for your positive evaluation and constructive comments. We describe how we have addressed the substantive concerns below.

Nonetheless, the paper has several important limitations that undermine the scientific value of the paper in its current form.

1. Lack of power analyses and interpretability of null effects

A significant contribution of the paper is the clever manipulation of conversational features. Unfortunately, this contribution is undermined because the current analyses do not reveal whether the null (interaction) effects of these manipulations are meaningful. The paper does not include power analyses, so that readers cannot distinguish whether the null effects indicate that the actual effects are likely null or whether the studies were underpowered.

This is particularly relevant for the interaction effects in Studies 2A-C. The authors write on p. 20: “Further, none of the conversational structures significantly interacted with the effect of
conversation medium on perceived understanding or conflict, suggesting that although speaking may tend to be a more interactive and synchronous medium than writing,

manipulating those aspects was not enough to reduce the effect of medium on perceived understanding or conflict."

A non-significant interaction cannot be interpreted as meaningful without power analyses (or other statistical methods such as Bayesian statistics). To address this point, the authors could conduct sensitivity power analyses to describe what effect they could identify with sufficient power and be clear for what kind of effects they were underpowered. Due to the complexity of their design, the authors will need to assume several parameters to conduct power analysis. I'd recommend running sensitivity power analysis that use several estimates from the studies they conducted as input, such as the sample size, means, standard deviations and correlations between variables relevant for the data analysis. Using these estimates, they can simulate what effects they were able to find with 95% power.

Thank you for your suggestion. Following your recommendation, we performed sensitivity power analyses to estimate the smallest interaction effects that could be reliably detected at 95% power (α=0.05) in Studies 2A, 2B, and 2C. The results indicate that our studies were sufficiently powered to detect moderate interaction effects but may have been underpowered to detect small interaction effects (2A: f^2 = .072; 2B: f^2 = .065; 2C: f^2 = .064). This suggests that it is unlikely we missed any strong interactions, but we cannot rule out the presence of smaller, undetected interaction effects. We have now clarified this limitation in the manuscript.

Additionally, we conducted a power analysis for the main effects of the communication medium that we observed on perceived understanding and perceived conflict in Study 1. Our analysis indicates that our studies had high power (≥ 0.99) to detect the effect of medium on perceived understanding (a large effect in our studies). For the effect of medium on perceived conflict (a smaller effect in our studies), power was somewhat lower (ranging from 0.837 in Study 2A to 0.876 in Study 2C). This reinforces confidence in the observed main effects while acknowledging limitations in detecting smaller effects, particularly of medium on perceived conflict.

Thank you again for this helpful suggestion.

2. Lack of accounting for interdependency in analysis strategy

Due to the interactivity, the analyses are more complicated than standard experimental analyses. Therefore, I would suggest that the authors spent significantly more space on explaining their analytical choices. I have the following questions

First, how does the analysis in Study 1 account for the fact that treatment assignment was at the session level? My intuition is that this should result in less power than treatment assignment at the dyad level. Yet, the analysis strategy does not account for assignment at the session level.

Second, how does the analysis in Study 3 account for the fact that treatments involved multiple conversations? The authors write they cluster standard errors at the individual level. But do they also account for the dependency between partners of the same conversation?

Both of these concerns are valid statistical issues that we can solve in the same way, using multiple levels of clustered standard errors. For Studies 1 and 2, we cluster at the session and dyad level. For Study 3, we cluster at the person and dyad level. In Study 3, we additionally control (using fixed effects) for site and round if the data are pooled and control for round only if the data are at the site level. We have adjusted our regression modelling function to use the multiwayvcov package, which handles multiple levels of clustering throughout. None of our results change substantively, but we are glad to make this improvement to the paper regardless.

3. No causal evidence for the role of conversational receptiveness.

The paper emphasizes the role of conversational receptiveness for driving the effects. For example, the authors write on p. 44-45: “Our experiments also demonstrate the power of linguistic choices in the perception of conflict. At least one of the reasons why communicators experienced less conflict, and felt greater understanding, when speaking than writing is because they used more receptive language. The increase in conversational receptiveness can be specifically attributed to greater use of subjectivity phrases and fewer uses of negative emotion phrases in speech than writing. Notably, we also found that conversational receptiveness is more effective in writing than speech.”

Because the authors did not demonstrate the causal effects of conversational receptiveness, I see this a significant overstatement. An experiment that manipulates of conversational receptiveness to test its main effect and/or interaction effect with medium

would be fascinating. But unless the authors provide such evidence, the language on the role of conversational receptiveness would need to be toned down significantly.

Thank you for this concern, and we agree that our original wording overstated the causal role of conversational receptiveness. In response, we have revised the language throughout the manuscript to adopt a more measured tone. For example, in our discussion in the Language Analyses section, we now write: "Speaking fosters greater conversational receptiveness than writing, and particularly enhances linguistic features like subjectivity phrases and adverb limiters while reducing negative emotion. Receptive language also mediates, at least partially, the effect of medium on constructive disagreement outcomes (e.g., perceived understanding, conflict)." In our General Discussion, we write: "Our experiments suggest that linguistic choices likely play a role in shaping perceptions of conflict. One possible reason why communicators reported experiencing less conflict and greater understanding when speaking than writing is that they tended to use more receptive language." Specifically, we have softened claims about causality and now describe conversational receptiveness as a potential factor in the observed effects, rather than as a direct driver of the observed effects.

4. Confounds in Study 3

Study 3 – which is described as a field experiment that allows generalizing beyond lab experiments – features several design decisions that complicate the interpretation of the findings. First, Study 3 featured multiple conversations. As a result, responses to the second – fifth conversations cannot simply be ascribed to differences in conversation medium but are also the product of the different conversations participants had in prior rounds. This could be addressed be restricting the analysis to only the first round.

We took your suggestion to include an analysis restricted to the first round of conversations. However, it is important to note that a power analysis of Study 3 limited only to the Round 1 data suggests that we are underpowered to identify an effect of medium on perceived understanding (power = .628) and perceived conflict (power = .482) with just the Round 1 data. Thus, any results need to be interpreted with caution.

The results from this analysis showed non-significant effects on perceived understanding (standardized $\beta = .69$, $SE = .39$, $t(23) = 1.74$, $p = .095$) and perceived conflict ($\beta = -.33$, $SE = .46$, $t(23) = 0.73$, $p = .474$). The direction of results and effect sizes are consistent with those from the analysis of all rounds of data. Because we cannot know whether these results support the robustness of our

conclusions, we include them only in Appendix H. We would also like to note that each participant completed all their conversations in only one medium. So if there are systematic differences in previous conversations, that itself could be attributed to the randomly assigned condition. Therefore, if there is some bleed-over across sequential conversations, this is not necessarily a confound of the treatment effect.

Second, an important distinction between the spoken and the written conversation in Study 3 is that the written conversation is very unnatural. Because participants sat across each other, speaking is much more natural than chatting. This confound cannot be addressed without additional data collection. It is reassuring that the results are consistent across the conversations. But the boost that a paper should get for a field replications is limited by this design feature.

We were extremely conscious of the trade-off between external and internal validity while designing Study 3, and we deliberately chose to sacrifice some external validity (as you note, it is not typical for two people to be chatting via a text-based medium while sitting side-by-side, although this does sometimes happen) in order to increase our internal validity (having pairs converse in the exact same physical setting in both conditions rules out many possible alternative explanations that could be driving differences between speaking and writing, e.g., anonymity). Overall, we believe the boost to our internal validity was worth the sacrifice to external validity.

Still, to address your concern, we now explicitly note two limitations of the study in the paper. First, we note that Study 3 could be considered a "framed field experiment" (as defined by Harrison & List, 2004) because participants knew they were in a study. Second, we acknowledge that our design choices may have sacrificed some external validity, particularly in the writing condition. We write: "We designed this procedure to enhance our internal validity (for example, ensuring participants would see each other regardless of whether they were typing on a laptop to their partner or speaking above the laptop with them) but note it may also have reduced external validity (e.g., potentially making the experience feel less natural, perhaps particularly in the writing condition)." We do not see how differences in typicality (or naturality) would create the effects we observed, but hope you will appreciate that we mention the potential confound in the paper regardless so that readers can draw their own conclusions about it.

Third, another confound is that spoken and written conversations differed in length (although only at the ASU site) is. One may argue that the lack of significant interactions

in Study 2b means that length does not matter. However, as I write in my first point, I am not convinced by that result.

We deliberately gave participants twice as much time to write as to speak at the ASU site in order to provide a conservative test of our hypothesis. Although this confound was working against our hypothesis, we still observed the same effect of conversation medium at the ASU site. We now provide our analyses broken down by site, since there were slight modifications in our paradigm for each site (see Appendix H). These analyses show consistent, statistically significant effects of medium at each site individually. We hope that these results will help to reassure you that our effects are robust to conversation length, at least for the lengths we tested.

Regarding the broader question of the role of conversation length in conversation experiences, we have now conducted some additional analyses. First, and not surprisingly, there are significantly more words in the spoken than written conversations across Studies 1 and 2 (Supplementary Table S19). Second, correlational analyses using pooled data from Studies 1 and 2 show a small positive correlation between word count and perceived understanding ($r = .151$, $p < .001$) and an even smaller but not statistically significant negative correlation between word count and perceived conflict ($r = -.028$, $p = .295$). These associations can also be observed in the regression results in Supplementary Table S20. Finally, our results are robust with and without word count as controls across Studies 1 and 2 (as reported in the Language Analysis section in the manuscript). Note that we cannot conduct these analyses for Study 3, because we were not able to collect recordings for most conversations across the sites.

Overall, these analyses suggest that conversation length could be relevant for outcomes like understanding and conflict, but the effect of medium appears to be robust to the conversation length, at least in the contexts we tested.

Fourth, some participants had four and some had five conversations in Study 3. It is unclear why the experience was not identical across all participants. This could be addressed by adding an explanation and testing for differences across conditions. Furthermore, it could be cleanly addressed by restricting the analysis to the first round of conversations.

Given that we used a field setting in Study 3, we did not have perfect control of when participants arrived or left the conversation events. Some people stayed for the entire event (about one hour), and others left. Thus, there was variance in the number of conversations participants had (ranging from one to seven

conversations), but a one-way ANOVA revealed no significant differences in the number of conversations across medium in Study 3 ($F(1,86)=.54$, $p=.46$). We now report the distribution of the number of conversations per participant in Appendix H for additional transparency.

We account for potential differences in the number of conversations in several ways. First, as described earlier, we conducted a subset analysis using only the first conversation. The results are consistent with the pooled analyses of Studies 1 and 2, though the findings were not statistically significant, likely due to reduced statistical power. These results are now reported in Appendix H. Second, we tested whether the conversation number moderated any effects of the conversation medium. We did not observe any statistically significant interactions, which could suggest that the number does not influence the effect of medium or that we were underpowered to detect it. At the least, we can find no evidence that conversation number significantly influenced the effect of medium.

5. Unclear conceptualization of constructive disagreement.

The authors define (or operationalize?) constructive disagreement as perceived understanding and conflict. However, it is unclear whether this definition is widely used or deviates from other definitions/operationalizations in the literature.

Furthermore, in the abstract and parts of the introduction, the conceptual differences between perceived understanding and perceived conflict and two other outcomes (impressions of conversation partner and attitude alignment) are unclear. The paper could be improved by clarifying that these are different outcomes. In fact, attitude alignment is fundamentally different from constructive disagreement because it implies a reduction in disagreement.

The paper could be further improved by conceptually distinguishing conflict from disagreement. Conflict is sometimes used as a synonym for disagreement but the items the authors use suggest that conflict is meant in a more emotional sense.

We appreciate this feedback, which has helped us clarify our conceptualization of constructive disagreement on pages 6-7. We propose that constructive disagreements promote understanding - a key goal in exchanging diverse perspectives – while also minimizing perceptions of conflict. Conflict scholars draw clear boundaries between mere disagreement (you think X, I think Y) and perceived conflict, which involves emotions, antagonism, and hostility. We explain the idea of the conflict spiral, which demonstrates how disagreement can

lead to conflict (Andersson & Pearson, 1999; Brett et al., 1998; Weingart et al., 2015).

We focus on these two outcomes of perceived understanding and conflict because they are particularly proximal to the conversation itself, whereas other outcomes we measured seem to be more relevant to downstream consequences (e.g., attitude alignment and impressions of the conversation partner). The other outcomes we measured tend to be correlated with each other in our data and in past research as well. We think it is important to have both conflict and understanding as joint outcomes in our main DV because, as we explain in the paper, you can address one at the expense of the other in unhelpful ways. That is, you can avoid interacting altogether, which minimizes conflict but cannot produce understanding. Likewise, understanding that comes at the expense of intense conflict can be counterproductive.

6. Differences between lay study and experiments.

The lay study is a nice addition to show that the experimental findings are surprising. Ideally, these studies would be as similar as possible to allow clean comparisons. However, there are at least two important differences between the lay study and experimental findings. First, the lay study uses a within-subjects manipulation whereas the experiments featuring actual conversations use between-subjects manipulations. Second, the sampling populations are quite different. These limitations should be acknowledged as potential alternative explanations for the observed differences.

We agree with you that both of these limitations should be mentioned in the manuscript, and we have now done so (pp. 50-51). We can also explain why we selected a within-subjects design for the lay study. We reasoned that the within-subjects framing is analogous to someone prospectively choosing the medium by which to conduct a conversation, whereas retrospective assessment is rarely done with two similar conversations in different mediums (akin to a between-subjects design). Thus, the within-subjects design seemed more appropriate for answering our research question in the lay study.

7. Lack of clarity which studies and analyses were preregistered.

The paper is unclear about which studies are preregistered. Statements such as "predetermined exclusion criteria" in Study 1 are confusing. Readers may understand this as "preregistered" but (i) Study 1 was not preregistered and (ii) the exclusion criteria were not described in the preregistration. This could be improved by specifying in the

text which studies were preregistered and how the studies deviated from the preregistration.

Thank you for noting this. Study 1 was not preregistered, but we used the same analysis strategy (e.g., data exclusions, regression models, outcome measures) for all of our lab experiments, so this limits our researcher degrees of freedom, which is why we described it as "predetermined exclusion criteria" in the manuscript. However, we can now see that this phrase is misleading. We have updated the language in the paper to make it clearer which studies were preregistered. Specifically, we report this information in our Open Science Disclosure as well as on page 9 in the manuscript and Appendix A.

The links in the data and code availability sections does not provide access to the OSF project. However, the link in the transparency statement does provide access. This could be improved by providing access through all links.

Thank you for highlighting this. The links have now been amended in the main and supplementary information documents accordingly. This is the URL for the current link (which we will make public as soon as the manuscript is accepted for publication): http://bit.ly/3uN0TnB

Thank you for the opportunity to review this paper. I wish the authors all the best and hope my feedback is helpful to improve the paper.

Jan G. Voelkel

Reviewer #2 (Remarks on code availability):

The link https://osf.io/c3zgy/ (which is also listed in the data and code availability sections) does not provide access to the OSF project. However, the link in the transparency statement does provide access.

I looked up the code for some analyses decisions which informed my review but I did not try to reproduce the findings.

Please see our previous comment to # 8 above. All links have now been updated, thank you for double-checking!

Reviewer #3 (Remarks to the Author):

The present paper investigates the effect of written versus spoken conversation on constructive agreement (less conflict and more understanding) in several experiments. The main finding is that spoken conversation leads to more constructive agreement. This is mediated by 'receptiveness' of the language. However, receptiveness has a stronger effect on constructive agreement in written than spoken conversations. In contrast to these findings, lay theory suggest that written conversations will be more constructive than spoken ones.

In my view, this paper addresses an important research question that is of interest to a broad audience and relatively novel. The authors present a large amount of evidence from several experiments, supporting their main claim. In addition, they report on several interesting extra experiments that deepen understanding and relevance regarding the found effects. I have several questions and remarks on the paper, which I list below, but most are relatively minor and can be easily addressed by the authors I think. There are also three somewhat larger issues that I believe do need to be addressed before publication is warranted.

Thank you for your kind words and keen attention to the research! We are happy to make revisions based on your remarks, as detailed below.

Major remarks

1. I believe the introduction of the paper could benefit from including some other relevant studies. First, specifically two papers from Roos et al. (2020a&b) appear very relevant to the present study. These papers also investigate differences between online (written) and face-to-face discussions about controversial topics. Apart from some methodological differences (e.g., groups of 3/4) the studies' outcome measures are also somewhat different from the present study, but they do appear related and very relevant (i.e., responsiveness, ambiguity, disinhibition, polarization, and solidarity). I would suggest the authors discuss these papers and indicate what their study adds to the insights of those results (and potentially other papers from the same research group). Somewhat less relevant is a paper by Sillars & Zorn (2020) showing that negative email is often interpreted more negatively than it was intended, which also goes in a similar direction as the present results. I may not be aware of all relevant studies in the field, so the authors may additionally want to do a more thorough literature search.

Thank you for your close focus on our contribution. We have integrated the literature that you recommended into the paper, and we believe our theoretical contribution is now much clearer and stronger.

In particular, we think there are at least three ways in which we substantively move beyond prior research, including the Roos et al (2020a, b) and Sillars & Zorn (2020) papers. First, we are studying contexts in which we either explicitly measure disagreement between conversation partners or create disagreement by deliberately pairing together individuals who have different opinions. By examining contexts in which there is disagreement, we can study potential conflict. Many other papers have not examined disagreement contexts. In Roos et al (2020a, *JCMC*), the authors used confederates to provoke a group that all had the same position. In Roos et al (2020b, *GPIR*), it was not clear whether participants disagreed with each other, as issue positions were not measured either before or after the conversation. Thus, the question of how communication medium influences disagreement outcomes, specifically, was not tested in either paper. (We also note that Roos et al (2020 a,b) focused on more short-term outcomes about the conversation itself (e.g., fun, clarity) rather than the relational outcomes we examine (e.g., understanding, conflict).)

Second, we think our work is empirically stronger than prior research. For example, we are reasonably well-powered to detect effects in real-time conversations, with a pooled sample of 1,576 conversation partners. In contrast, Roos et al (2020a and b) had only 204 participants *combined* between both papers. Relatedly, we think our experimental designs are cleaner. For example, both of the Roos et al papers examined contexts in which participants had many conversations within groups, and there were multiple people per group. This changes how people interpret disagreement, whether they speak up or stay silent, and how they build trust. The papers also did not measure order or agreement effects (and the sample sizes are too small to test such effects anyways). In contrast, we assign participants into pairs to test our hypotheses, providing a more direct test of the effect of medium.

Finally, we collect actual behavior - the conversations themselves. This allows us to conduct more sophisticated language analyses, getting inside the “black box” of the conversations themselves and testing a new mechanism for the potential effect of medium - conversational receptiveness. Earlier work typically did not record the conversations and could not test such questions.

Second, the discrepancy the authors report between lay theory and research findings may be related to other findings in psychology, such as the finding in Kruger et al. (2005; already cited by the authors but not in this context). This study showed that we think we can communicate better over email than we actually can, which may be related to the present finding that we think our textual communication is more constructive than our verbal communication, while this is not the case.

We appreciate this insightful connection that you made between our work and Kruger et al (2005). Indeed, there are several papers now indicating that people may mispredict the consequences of their communication choices. For example, Schroeder and Epley (2015) found that the communication medium changed impressions of communicators but the communicators themselves did not anticipate its impact. Kumar and Epley (2021) further found that people mispredict how awkward phone (vs. email) conversations with old friends will be, which can lead them to prefer email even though phone conversations facilitate greater connection. We extend this body of work by demonstrating a case whereby people mistakenly believe textual communication is more constructive than verbal communication. We now mention this literature in our paper.

2. I also have several comments and questions related to the variable of 'receptiveness'. This term was slightly confusing to me at first because I expected it to be more about interactional responsiveness (as Roos et al., 2020a use the term responsiveness), that is, whether the communicators orient towards each other and appear to listen to each other in their speech.

Thank you for pointing out the similarities in terms. We are familiar with both constructs, but chose to focus on receptiveness for several reasons. Receptiveness was developed specifically for conflict and can be measured directly from transcripts using an algorithm. It has been validated via several large-sample pre-registered experiments with real conversations (Yeomans et al., 2020; Minson et al., 2024; Tormala & Hussein, 2021).

Although responsiveness has a deeper history in the literature, it was developed mostly for intimate relationships (Reis & Clark, 2013; Reis & Gable, 2015) rather than conflict (especially intra-group political conflict, like we study in the current paper). It is not well-understood how to assess it from behavioral data: We know of one paper which found linguistic markers of responsiveness (Huang et al., 2017), but those were friendly conversations, and the behavioral feature involved, follow-up questions, does not predict successful conflict management because

many follow-up questions that demonstrate listening are still hostile and argumentative. For these reasons, although we agree that responsiveness is a relevant construct, we prefer to stay focused on receptiveness in our paper.

This may also relate to the use of interjections for example, which are very relevant interactional devices in conversation (see e.g., Dingemanse, 2024). It appears that the 'receptiveness' variable does not fully capture these aspects that may also be very relevant (acknowledgements and agreements appear to come closest, but they may be more explicit, judging from the examples).

We agree it is interesting to examine various conversational behaviors beyond the overall receptiveness score. But because conversation has an essentially infinite number of degrees of freedom, some focus is necessary. We decided to focus on receptiveness because it has an established theoretical and empirical grounding in the conflict management literature. Dingemanse (2024) reviews a body of work on interjections from linguistics, which has a different standard for evidence - they do not quantitatively estimate any effects (i.e., presenting either correlational or causal evidence), but instead provide narrative arguments about stylized examples. This also means they do not provide a reproducible definition of what an interjection is. In their review, they note that there are many types of interjections, both helpful and unhelpful. And the meaning of an interjection depends on the context (e.g., "yes" could be an answer to a question, a backchannel, an apology acceptance, an acknowledgement of common ground, and so on). They also do not discuss interjections in relation to conflict or disagreement, which is the focal context for this paper. If you have a specific prediction you would like us to test, we would be happy to conduct those analyses

It is interesting in this regard that, in study 4, transcribed speech leads to more conflict and less understanding. Looking at the transcript instructions, precisely these interjections may have been taken out in the written versions. Such responsiveness may be more expected to occur in conversations with more interactivity. Therefore, it may be interesting to analyze study 2A to see if there is more receptiveness in the interactive condition.

Your intuition is correct. We did indeed find more receptiveness in the interactive conditions than the non-interactive ones in Study 2A, in both Speaking ($B = 0.364$, $SE = 0.1461$, $t(88) = 2.492$, $p = 0.015$) and Writing ($B = 0.5727$, $SE = 0.1488$ $t(91) = 3.849$, $p < .001$). (Note that we did not include this result in the main paper due to

length constraints, but we are happy to add it if you think it is important to the story.)

Last, it is interesting that, as seen from Figure 5, not all presumed elements of receptiveness actually go in the expected direction. Especially more ambiguity (Adverb. Limiter) is found in speaking (which was incidentally also found by Roos et al., 2020a) whereas this is seen as negative for receptiveness.

Limiters have primarily been tied to minimizing requests in the politeness literature (Voigt et al., 2017; Minson et al., 2025), which is why we examine them. We reviewed the Roos paper and did not find any mention of limiters. You are correct that limiters do not seem to follow the same pattern as other conversational behaviors, which may suggest interesting theoretical developments. We do examine two more traditionally accepted markers of uncertainty (Hedges and Subjectivity) that play a prominent role in receptiveness, which are discussed extensively in the language analyses.

This begs the question whether receptiveness is the right construct to focus on in the present study, or whether it makes more sense to focus on more specific constructs or variables to see how they can explain the difference between spoken and written communication, including perhaps something that is more related to 'responsiveness'. At least I would like to see the authors discuss these issues in the General Discussion.

We discussed at length earlier why we chose to focus on receptiveness instead of responsiveness. We appreciate that you encouraged us to defend our use of this construct more rigorously.

References:

Reis, H. T., & Clark, M. S. (2013). Responsiveness. In J. A. Simpson & L. Campbell (Eds.), The Oxford handbook of close relationships (pp. 400–423). Oxford University Press.

Reis, H. T., & Gable, S. L. (2015). Responsiveness. *Current Opinion in Psychology*, *1*, 67-71.

Huang, K., Yeomans, M., Brooks, A. W., Minson, J., & Gino, F. (2017). It doesn't hurt to ask: Question-asking increases liking. *Journal of personality and social psychology*, *113*(3), 430.

Voigt, R., Camp, N. P., Prabhakaran, V., Hamilton, W. L., Hetey, R. C., Griffiths, C. M., ... & Eberhardt, J. L. (2017). Language from police body camera footage shows racial disparities in officer respect. *Proceedings of the National Academy of Sciences*, *114*(25), 6521-6526.

Minson, J.A., Yeomans, M., Collins, H.K., Dorison, C.A. & Gino, F. (2025). Conversational receptiveness transmits between parties and bridges ideological conflict. *Journal of Personality and Social Psychology.*

3. I do not understand the description of the mediation analysis (7.2.4). It is stated that the direct and total effects are significant, but the authors should be interested in the *indirect* effect, which would be the (part of the) effect of medium on understanding that is mediated by receptiveness. They do not mention any indirect effects. As it is stated now, it seems the total effect is .35 and the direct effect (not mediated by Receptiveness) is .31. That would imply that the indirect effect is very small (.04) and unlikely to be significant. It would be important to check exactly which effects are found here.

We appreciate your suggestion to clarify the mediation analysis. You are correct that the indirect effect is around .04; we now explicitly report the average causal mediation effect (ACME) using bootstrapping with 20,000 simulations on p. 44 which shows the indirect effect to be .035 (95% CI: [0.0097, 0.060], p = .0042). Although perhaps not large, it is indeed statistically significant and we believe worth reporting. We hope these additions clarify the role of receptiveness in mediating the effect of conversation medium on perceived understanding.

Minor remarks
1. At the start of the Introduction (page 3) it is not immediately clear which type of media are the main focus of the paper (namely, text vs. speech). It would be helpful to spell that out early.

Good point - we have now made this point more explicit by adding the sentence, “Specifically, we focus our comparisons on two ubiquitous conversation mediums - spoken and written” at the top of page 4.

2. I missed some methodological details that I believe would help the reader if they were present in the main text (and not only in an appendix):
- Study 1, page 8: it is unclear that pair members are strangers to each other

We have clarified in the main text that participants were recruited individually and paired based on disagreement. The intention during pairing was to make sure each dyad was of strangers. We believe that most participants did not already know their partner because our laboratory participant pool contains over 10,000 people, and, in other studies which used the same participant pool, fewer than 5% of random pairs reported knowing each other.

We also collected some empirical data about whether pairs knew each other in Study 2C. Specifically, we asked some of our participants (in sessions 28 - 38) whether they already knew their partner or not; 6 of the 134 (5%) indicated they did. Because we drew from a similar population pool in other studies (1, 2A, and 2B all came from the same university subject pool), these results suggest the grand majority of our participants did not already know each other.

- Study 2A: What is the structure of the conversations here: is there interaction throughout the conversation or is there again allocated speaking time as in study 1?

We have added a clarification that in the multiple exchanges condition, each pair had 6 minutes. In the single exchange condition, each pair also had 6 minutes, but each speaker had exactly 3 minutes to speak. A timer was used to make sure the speakers did not overrun.

- Study 2A: (page 15, first line) "both people prepared their statements at the same time". It is not clear what this exactly means in speech and writing. I assume that in writing, people type their response at the same time, whereas in speech they think about what they want to say at the same time (but say it after each other)? In the latter case it is still possible for the second speaker to react to the first, which makes the two situations not really equivalent.

In both the speaking and writing conditions, participants each had time to prepare statements in advance, without knowing what the other person would say. One person was randomly assigned to deliver their statement first (in speaking or writing), and the other person followed. You are correct that the second person in the speaking condition could conceivably modify their statement in reaction to the first person, possibly making the speaking condition slightly more interactive than the writing condition. We tried to control differences between the conditions as much as possible while still making them true to life - it would have been impossible to have both people deliver their speeches at the same time, whereas written interactions often do occur simultaneously in real life. Thus, we chose to prioritize slightly more external validity in the design of this

study. However, we acknowledge your point and now note in the paper that it is “possible the second person deviated from their prepared remarks after listening to or reading their partner’s opinions.”

- Study 2C (3.3.2): the structure is not totally clear to me here: “in round one, Person A (randomly assigned) was given one minute to speak or write, while Person B had one minute to listen or read”. I suppose this is sequential (but the word while is confusing here)? If it is, doesn’t that mean that the conversation time in this condition is actually 12 minutes, because it includes 6 minutes of ‘waiting time’?

Thank you for pointing out this ambiguity - we have revised “while” to read “and then” to make sure the temporal order is clear. We state that there are 12 minutes of total interaction time in all conditions.

- Study 3, page 23: “recordings were attempted at one location but the background noise was too disruptive”. How was this for the participants during the study? Could they hear each other well and were they not distracted by the background conversations?

Humans are remarkably good at focusing their attention on a voice in a crowd, and participants did not have trouble hearing each other (although it is impossible to rule out momentary distractions). For what it’s worth, the experiments were all conducted in the same room, so the external distractions should be similar across conditions. However, machines do not have the ability to pick out distinct voices, especially when limited to a single non-directional microphone, which is why the recordings were unsuitable for transcription.

3. Paragraph 4.2.2: It seems that the interaction effect between attitude distance and medium is only performed for agreeing dyads. I wonder why it was not performed for the whole sample (agreeing and disagreeing dyads, which can be seen as a continuum), because the variance of attitude distance would be much larger in that case.

Sorry, we were not clear in our write-up. For those interaction tests, we do in fact use the entire sample, for exactly the reasons you lay out here. We have clarified this in the manuscript.

4. Page 27: The effect on attitude shifts is very small and only marginally significant. What does that mean for the relevance of the findings in general? Wouldn’t one expect that constructive disagreement is more effective in bringing conversation partners closer together in terms of their opinion? I think this would be relevant to discuss in the

General Discussion (even if it was just measured in one study, it could be suggested for future research).

It suggests that although spoken conversations improve understanding and reduce conflict, they do not strongly or consistently change people's attitudes. This is unsurprising to us as the topics discussed by the students, and indeed in polarized societies, are important to people and may be part of their long-held views. Therefore, those views are unlikely to be strongly influenced by conversations in the short term. However, even if attitude change is limited, the fact that spoken dialogue fosters greater perceived understanding, reduces conflict, and improves interpersonal outcomes is still meaningful. Changes in attitudes could be more likely to occur over time as a consequence of greater constructive disagreement in specific conversations. We have added a note about this in the General Discussion, as you suggested.

5. The authors use the term 'linguistic content' not only for the actual semantic content of what is said, but also for how (formal, polite, etc.) something is said. It might be helpful for some readers to clarify this early on that the authors also include this aspect under 'linguistic content'.

Thank you for pointing out this confusion. "Linguistic content" is a phrase we used twice in the paper, colloquially. We meant to refer only to semantic content (i.e., the words and verbal utterances being used), rather than auditory features like tone of voice, when we used the phrase. Formality and politeness are primarily properties of language, so this would be included in "linguistic content". We have now more clearly defined "linguistic content" in the paper and hopefully it is much easier to read fluently now.

6. Page 40. Even though the effect of medium on receptiveness is still significant when controlling for word count, sentiment, and politeness, the effect does appear to be smaller than without these controls. It may be interesting to analyze whether any of these control variables mediate the effect to see if they can still partly explain the effect (though not fully as this analysis suggests).

This is an interesting hypothesis, but we have been very cautious to not develop overly complex mediation models using variables within the conversation. In our main model, this is less of a problem because the conversation finishes before any outcome measures are collected. However, all those other text variables are somewhat correlated with receptiveness, and are also interspersed in time. For example, if I am receptive in the first half of a conversation, you might speak

more, or more politely, in the second half. So which variable is mediating which? A much more fine-grained analysis of these time series issues is required to take any of those kinds of analyses seriously. We think that is beyond the scope of this paper (in fact, those methods may not exist with any reliability, that we are aware of).

7. Study 5: Effect of preference. It might be relevant to discuss the possibility that preference is related to other factors sch as awkwardness and effort, especially when talking to a stranger. Since there is no comparison with a non-conflict situation, it is also not clear whether this effect is related to the conflict or not.

We acknowledge that we did not test people's preferences or predictions regarding non-conflict situations - this is an interesting idea! This paper focuses on disagreement and perceived conflict, not because it is necessarily distinct from other situations, but because the relational stakes are high, and understanding these situations is important in its own right. We have added an acknowledgment of this limitation in the discussion of the lay study description.

We have relegated the results from our other measured factors to Appendix Q for brevity (this paper is already quite long). You are right that effort is, in fact, an important predictor of which medium people tend to choose. However, because these are correlations and multiple variables change across medium, we can't really disentangle them here in a clean way.

8. Page 45: Discussion of moderating effect of medium: An alternative explanation here might be that precisely BECAUSE receptiveness is relatively infrequent in written conversation, it is more meaningful when it does occur, and therefore the effect is larger.

This is a great point that we have acknowledged in the discussion section of the language analysis. Specifically, we state that "there are many possibilities for why conversational receptiveness might be more effective in written communication, including lower usage rate of receptive language. That is, conversational receptiveness may be more effective when used sparingly."

9. Page 45: Discussion of Study 1: no difference between video chat and spoken conversation: This finding may also be related to the fact that video chat is different from face-to-face conversation since it does not allow for eye contact for example.

This is perhaps true - though anecdotally, we have found that people can still look distracted on Zoom. We have revised the paper to be clear about describing that

condition as video chat, rather than face-to-face, and touch on the comparison in the discussion of Study 1.

10. Page 46: Discussion of production and consumption effects: I think it should be noted here that the finding that "the same words were interpreted differently when heard versus read" only holds for originally spoken materials. As mentioned above, this effect may be related to the transcription instructions since some words (i.e., interjections) that may be important were removed.

Good point! We have softened the claim here. However, we also note that in Study 4, we only used data from the single exchange condition, where interaction was minimized, precisely to get around this interjection issue. So we think this is less of a concern for interpreting the final results.

11. Page 49: The discussion here about participants' engagement may also be relevant for experiment 5 (at least the preference measure). It is likely that the participants that were asked to imagine being part of another experiment had different goals and engagement: they were likely less (intrinsically) motivated to solve the conflict, so the extra effort they believed the speech condition to entail may not have been worth it for them.

This is a good point. We've added it to the caveats for interpreting the results of the lay theory study, in line with some of Reviewer #2's suggested caveats above.

12. Paragraph 9.3: Limitations: Another limitation that could be mentioned is that all studies (as far as I can tell) involved conversations between strangers, not friends, where these effects may be different (this is especially imaginable for the preference effect in study 5, but may be the case for other results as well).

This is a good point. We have now added some language in limitation 2 of the discussion section to explicitly consider the relational context of the disagreement.

Textual remarks
- I am not a native speaker of English, but I had not heard of the word 'vitriol' before (page 3), and had to look it up. The authors may consider using another word here or explain what it means.

Thank you for alerting us that the word was confusing to you. We have removed it from the paper.

- Page 4: ‘speech can accurately convey an experience or intention even without semantic content’, would be nice for the reader to know how this information can then be conveyed (e.g., by prosody?).

Yes, presumably speech is conveying information via prosody beyond the semantic content. We have now added several citations to relevant work that should help explain this to interested readers, and we mention other sources of information in the manuscript now like prosody.

- Page 37: The description of Figure 5 (“The more a feature is used in writing than speaking, the higher it is on the y-axis”) appears to be opposite to the actual figure.

Thank you for the catch! This has now been fixed.

References
- Dingemanse, M. (2024). Interjections at the Heart of Language. Annual Review of Linguistics, 10(1), 257-277.
- Roos, C. A., Koudenburg, N., & Postmes, T. (2020a). Online social regulation: When everyday diplomatic skills for harmonious disagreement break down. Journal of Computer-Mediated Communication, 25(6), 382-401.
- Roos, C. A., Postmes, T., & Koudenburg, N. (2020b). The microdynamics of social regulation: Comparing the navigation of disagreements in text-based online and face-to-face discussions. Group Processes & Intergroup Relations, 23(6), 902-917.
- Sillars, A., & Zorn, T. E. (2021). Hypernegative interpretation of negatively perceived email at work. Management Communication Quarterly, 35(2), 171-200.

Thank you for these helpful references! We have added the two Roos et al papers to our main text now.

REVIEWER COMMENTS

Reviewer #1:

The authors have taken my suggestions to include audio data in their research as well as text and included their findings in the paper. Their results are quite interesting and add to the overall importance of the paper.

Thank you so much for your earlier audio data suggestions. We think that incorporating the auditory analyses have made this paper more interesting, and appreciate your engagement with our work.

Reviewer #2:

We appreciate how thoroughly you reviewed our paper for a second time! You have been instrumental in correcting several of our missteps, which we believe we have now addressed. Please see our responses below and in the paper. We hope we have managed to fix any remaining issues.

The revised paper could make an innovative and generative contribution to the literature. The central finding – that speaking, compared to writing, increases perceived understanding and reduces perceived conflict – is compelling and nicely demonstrated across several studies. The contrast to the lay theory study adds further value, and the limitations of this comparison are appropriately acknowledged. That alone would make for a strong and interesting paper.

Thank you for the positive words.

However, the paper also emphasizes other claims that are less convincingly supported. In particular, the concerns I raised in the previous round around interpretations of non-significant results, causal interpretations of correlational findings, and transparency in analysis decisions still appear to persist. I believe the paper would be stronger if it were shorter, focused more narrowly on the central finding (the effect of medium on perceived understanding and conflict), and more rigorous in evaluating and communicating the evidentiary basis for its claims.

We were hoping to write a shorter paper, but the paper has grown along with the review process. In the latest version, we tried to focus the reader more closely on the key results and streamline the less central results. We also tried our utmost to take care with our language so as not to overinterpret our results.

1. Interpretation of non-significant effects as evidence for the absence of an effect.

The addition of sensitivity power analyses for Studies 2A–C is welcome.

However, the manuscript still draws overly strong conclusions from non-significant results. For example: “Further, none of the conversational structures had a statistically significant interaction

with the effect of the conversation medium on perceived understanding or conflict, suggesting the effect of medium is not entirely due to speech being more interactive (2A), producing more words (2B), or being more synchronous (2C) than writing. However, these results must be interpreted with caution as we were not sufficiently powered to detect small interaction effects."

This statement is not supported by the sensitivity power analyses as reported. First, it is difficult to compare the power analyses and the findings reported in the text because the power analyses use f^2 and R^2 metrics, while the main text discusses beta coefficients. In my experience, interaction effects accounting for 6%–7% of the variance would require very large interaction effects. To meaningfully speak to what effects can be ruled out, power analyses should estimate the minimum detectable beta coefficients with 95% power. This could be done by running simulations using the parameters of the study and then varying the effect size. It would be appreciated if the code for the power analyses could be shared.

Based on the provided information, I am not convinced Studies 2A-C were powered to detect even interaction effects that would cut the effect of medium in half. Without providing further evidence, conclusions about the moderating roles of synchronicity, duration, and interactivity seem premature.

We agree with you that conclusions regarding any interaction effects (or, in this case, lack thereof) are premature in Studies 2A, 2B, and 2C. We have tried to address your concerns in three ways.

First, we conducted the simulation-based power analyses you recommended for Studies 2A, 2B, and 2C. In each simulation, we held the sample size and predictors constant, and generated new outcome values (e.g., for perceived understanding) by varying the coefficient (β) of the interaction term from −2.0 to +2.0 in increments of 0.05. For each value of β, we ran 1,000 simulations and estimated power as the proportion of simulations in which the interaction term reached statistical significance ($p < .05$), based on repeated sampling of synthetic outcomes.

We find that all three studies were underpowered to detect statistically significant interaction effects. The minimum detectable interaction effects were higher (βs for 2A: 1.80, 2B: 1.10, 2C: 1.25) than our observed interaction effects (βs for 2A: .03, 2B: .14, 2C: .24). In fact, it was not always possible for the minimum detectable interaction effects to be simulated with 95% power. The maximum power we could simulate interaction effects for across each study were (2A: 96%, 2B: 73%, 2C: 89%). As Cohen (1988, Chapter 2.4) states - "for most behavioral science research (although admitting of many exceptions), power values as large as .90-.99 would demand sample sizes so large as to exceed an investigator's resources. Even when, with much effort or at much cost, these large n's can be attained, they are probably inefficient, given the nature of statistical inference and the sociology of science."

Consistent with these results, the rate at which we can reject the null hypotheses for the interaction terms in our studies was low (2A: 5.7%, 2B: 10.9%, 2C: 23.3%), especially compared to the rate at which we can reject the null hypotheses for the main effects of

medium (e.g., in Study 2A our analysis suggests we had 99% power to detect the effect of medium). This discrepancy reflects the greater difficulty in detecting interaction terms due to smaller effect sizes and larger standard errors. The code (power_simulations.R) for these analyses are posted on OSF (https://osf.io/k73nq). These power analyses are now reported in Appendix F.

Second, because observed power provides little information beyond what is already conveyed by the p-value - "You can directly convert your p-value to the observed power, regardless of your sample size or effect size" (Lakens, 2022), we decided that rather than reporting post-hoc power analyses in the main text of the paper (which several sources including Lakens 2022 and Zumbo & Hubley 1998 recommend against doing), we would instead provide confidence intervals for all results. Confidence intervals offer a more continuous and interpretable measure of uncertainty and have been recommended as a more informative alternative to post-hoc power analysis (e.g., Wilkinson, 1999). We have amended the manuscript accordingly to include 95% confidence intervals for each result. Incidentally, this also helps keep the manuscript more concise.

Third, based on our additional analyses and your recommendations, we updated the writing in the paper so as not to draw conclusions from any statistically non-significant results. Regarding Studies 2A-C specifically: a) we shortened the introduction of these studies to focus primarily on the conversation medium manipulation (and less on the conversation structure manipulations); b) in the results section, we now explicitly report that the effect of medium on perceived conflict is statistically non-significant in Studies 2A and 2B; and c) we removed any mention of conversation structure in the discussion of these studies. We also removed speculation about any statistically non-significant effects in Studies 2A-C entirely from the General Discussion of the paper.

Cohen, J. (1988). Statistical power analysis for the behavioral sciences (Edition 2). Hillsdale, NJ. Erlbaum Publishing.

Lakens, D. (2022). Sample size justification. *Collabra Psychology, 8*(1), 33267. https://doi.org/10.1525/collabra.33267

Wilkinson, L. (1999). Statistical methods in psychology journals: Guidelines and explanations. *American Psychologist*, *54*(8), 594–604. https://doi.org/10.1037/0003-066X.54.8.594

Zumbo, B. D., & Hubley, A. M. (1998). A note on misconceptions concerning prospective and retrospective power. *Journal of the Royal Statistical Society: Series D (The Statistician), 47*(2), 385–388. https://doi.org/10.1111/1467-9884.00139

In other sections of the paper, non-significant effects are presenting without interpretation and corresponding sensitivity power analyses. For example, in the paragraph starting with the sentence: "Participants had multiple conversations in this study, so we tested whether the treatment effect varied over time."

Thank you for encouraging us to be more thorough. We now report test statistics for every finding in the main text. Ancillary findings were moved entirely to the Appendix. As discussed in our prior response, we also now include 95% CIs in our regression models to help with interpretation of the statistically non-significant results.

Regarding the test of the treatment effect over time, we have moved this analysis to Appendix H to streamline the paper. We now write: "We did not find statistically significant associations between conversation round and perceived understanding (standardized β = .04, SE = .06, t(352) = 0.78, p = .435, 95% CI [-.07, .15]), or interaction between round and medium on understanding (standardized β = -.01, SE = .07, t(352) = -0.08, p = .939, 95% CI [-.13, .12])." (We also realized this analysis could be conducted on both the initially agreeing and disagreeing dyads, which increased our sample size, so the statistics have changed but the conclusions are substantively the same.)

Test statistics are sometimes omitted altogether (e.g., "We also find no moderation of the treatment effect across the different locations").

We updated this analysis and have added the appropriate statistical tests into the main text. We now write: "To assess whether the effect of conversation medium on perceived understanding and conflict varied by campus site (UC Berkeley, ASU and MNSU), we conducted ANOVAswith the interaction term of conversation medium × campus site. The interaction effects were not statistically significant for perceived understanding, F(2, 97) = 0.94, p = .395, or for perceived conflict, F(2, 97) = 0.41, p = .667." We also visualize the results by campus site in Figure 3. We separately report the results for each campus site in Appendix H.

All non-significant effects need to be put in context so that the reader can distinguish between informative null effects and underpowered tests from which little can be inferred.

We had originally removed some of the statistical tests for non-sig results to streamline the paper (it is already very long, as you note) but, given your concern here, we have added these statistical tests back into the main text to help readers put those results into context. We now report all statistical tests in the text even when the result was not statistically significant. We also relegated some results to the Appendix to save space.

2. Interpretation of correlational findings as evidence for causal relationships.

Causal language is still used when discussing findings that are correlational, especially regarding receptiveness.

Your comment has opened our eyes to how frequently we use causal language even without meaning to do so! We fully concur that receptiveness was not experimentally manipulated and therefore cannot have a causal effect. (Note that sometimes when we referred to an "effect", we meant an "association" rather than a casual effect, but we now see that language was not clear enough.) We have now removed any suggestion that language has a casual impact on the post-survey measures.

For example:

“the (b) path confirms that conversational receptiveness increases perceived understanding.”

We now write: “the *(b)* path shows that conversational receptiveness is associated with greater perceived understanding.” In subsequent sections like the moderation analysis section, the Language Analyses discussion, and our General Discussion, we made several similar such edits to the language. To provide a few examples, instead of writing that “speaking fosters greater conversational receptiveness”, we now write, “people engage in greater conversational receptiveness when speaking than writing.” Instead of writing that “we test whether the effects of conversational receptiveness on constructive disagreement are mediated by medium,” we now write, “we test whether any associations between conversational receptiveness and constructive disagreement outcomes are moderated by medium.” And so on.

“Our experiments show that spoken media (e.g., using the phone, video-chatting, in-person conversation) induce greater use of receptive language than written media (e.g., text-messaging, emailing), but also that when used in speech, receptive language is less effective.”

We have removed this sentence to streamline the paper. We now write: “We further test exactly which linguistic features are associated with different conversation media and constructive outcomes.” We modified other paragraphs in the General Discussion in similar ways.

The causal role of receptiveness is not tested in the paper, as it is not experimentally manipulated. I raised this point in my previous review, and while the authors acknowledged the concern—writing in their response that they had softened causal claims—this change does not seem to be fully reflected in the manuscript. The discussion still implies a direct causal relationship.

We believe we have now addressed your concerns about casual language more thoroughly and consistently throughout the entire manuscript. Thank you for drawing our attention to this.

It is also worth noting that receptiveness only mediates approximately 10% of the total effect in the correlational mediation analysis.

We have added your caveat about partial mediation to the discussion of that section - we agree it is important to note that our mediation analysis only accounts for a fraction of the total effect.

3. Transparency

The paper includes many analyses, but it is unclear which were preregistered. The authors note:

“To reduce researcher degrees of freedom even in studies that were not preregistered, we apply consistent analyses across studies…”

However, analysis strategies appear less consistent than described. For example, Study 3 includes agreeing dyads, whereas other studies exclude them. The results are reported separately for disagreeing and agreeing dyads, and then again in a “prediction accuracy” section, which appears to pool both. Additionally, in the earlier analyses, a control variable (whether participants knew each other) is included, but it is not included in the prediction accuracy analyses.

I do not assume any ill intent, but these inconsistencies make it difficult to evaluate the robustness of the findings.

We tried to be as consistent as possible in our analyses across studies. However, it is important to note that a key feature of Studies 1 and 2 was the deliberate pairing of participants with opposing attitudes, so the majority of dyads disagree rather than agree. For Study 3, we intentionally did not pair participants based on opposing attitudes, meaning that we had a larger, more robust sample size of agreeing dyads. That’s why we treat the data slightly differently in Study 3. To conceptually replicate prior results, we first analyze the disagreeing dyads and then analyze the agreeing dyads. Note that after controlling for initial attitude differences in Study 3 across all participants in this study (regardless of their initial attitudes), the primary effects of medium remain statistically significant on perceived understanding ($\beta = .59$, $SE = .10$, $t(352) = 6.07$, $p < .001$, 95% CI [.40, .78]) and perceived conflict ($\beta = -.32$, $SE = .10$, $t(352) = -3.25$, $p = .001$, 95% CI [-0.51, -.12]). We also found that the effects of medium on perceived understanding ($\beta = .39$, $SE = .06$, $t(1478) = 6.78$, $p < .001$, 95% CI [.28, .50]), and perceived conflict ($\beta = -.20$, $SE = .06$, $t(1478) = 3.15$, $p = .002$, 95% CI [-0.32, -.08]) were still significant in Studies 1-2 after controlling for initial attitude differences. We have edited the description of our analysis strategy in the paper to explain it more clearly.

Regarding the control variable in Study 3, this was the only study in which we measured (and preregistered controlling for) whether participants knew each other. This difference is an accommodation for the recruitment strategy in the field sample, which had to be less precise than in the lab samples. That’s why we control for it in a robustness analysis, but the results are the same regardless of whether the variable is included in the model. This is also true in the prediction accuracy analysis for the ASU site (p 32), where we found that the effect of medium on predicting partner’s attitude was statistically significant with ($\beta = -.54$, $SE = .15$, $t(138) = -3.62$, $p < .001$, 95% CI [-.83, -.25]) and without controlling for participants’ prior knowledge of each other ($\beta = -.54$, $SE = .15$, $t(139) = -3.67$, $p < .001$, 95% CI [-.83, -.25]).

The transparency statement indicates that all preregistrations are posted on OSF. However, I was only able to access a preregistration for the lay theory study via the provided link.

We posted the preregistration links in our first appendix (Appendix A), but we see from your comments that we did not make them adequately accessible. To make the

preregistrations as accessible as possible, we did the following:

1. **We posted the public link to the preregistration in the Methods section for the relevant study in the main text. Specifically, for each study, we added a section called "Experiment Design" to report the design, technology platforms used if any, and the preregistration link. Studies that were did not preregistered we specifically report in that section (i.e., "This experiment was not preregistered.") We hope that including these "Experiment Design" sections will additionally help readers to follow our complex experimental designs and procedures.**
2. **We posted PDF versions of all preregistrations on OSF.**
3. **We describe our deviations from preregistrations in Appendix A. These data were collected over several years during which our science has evolved its views on preregistration. There was some vagueness in our original preregistrations which we now regret. We have tried to resolve this vagueness by prioritizing transparency above all else, and consistency across studies, so as to minimize researcher degrees of freedom wherever possible. Consistency is important because as has been mentioned, this paper has gotten quite long already during the review process. We believe the consistent analytical approach we have here best satisfies the many goals of science (including clarity, informativeness, and parsimony). Perfect adherence to pre-registration is not sufficient for good science by itself, and in any case our initial pre-registrations are too vague to be perfectly binding contracts (as might be expected in a registered report, for example). Instead we take our preregistrations as contemporaneous documents of our thinking, and have shared them in full so that readers can judge for themselves whether our estimated effects are credible. We should also point out that the deviations you mention are all too small to have any meaningful effect on the substantive conclusions of the paper.**

4. Attrition

Table S2 is helpful and appreciated. That said, there are at times large deviations from random assignment that raise concerns about differential attrition. For example:

In Study 1, 38% of video-chat participants versus 13% of speaking participants were excluded due to "pair-level exclusion."

We reviewed the raw data again and confirmed you are correct about the differences between condition exclusions in Study 1. We have investigated this issue in three ways (A, B, and C below). In Part A, we provide a detailed analysis of why attrition differs. In Part B, we show that when we relax our exclusion criteria, our main effects are robust. In Part C, we conduct balance checks confirming that random assignment was consistent across other pre-treatment variables. Combined, we believe this is a thorough analysis that indicates our estimated effects are due to treatment effects rather than to selection.

A. Attrition Analyses.

First, we conducted a more thorough analysis of attrition at each step in our experimental procedure (shown below in Table A1). As you can see in Table A1, participants were

relatively evenly distributed by condition in our original log sheet. About the same number completed the pre-survey and post-survey. The differential attrition by condition emerges at our analysis exclusions. We used two criteria to select "strongly disagreeing" participants: 1) participants needed to each report an attitude strength greater than 0 (non-neutrality requirement) and 2) participants needed to have a stance difference of 3 or more from their partner on the attitude scale (stance difference requirement). There was differential attrition by condition for only the stance difference requirement, both in the video-chat condition (67% of participants retained for analysis) and writing condition (75% retained) compared to the speaking condition (92% retained). Another way to report this difference is that, among all 396 participants from whom we have data, the video-chatting pairs (M=3.17, SD=1.63) and writing pairs (M=3.38, SD=1.52) showed relatively weaker stance differences than the speaking pairs (M=4.03, SD=1.15; video-chatting vs. speaking, $t(256.79) = 5.07$, $p < .001$, and writing vs. speaking, $t(236.53) = 3.87$, $p < .001$). As a result, there were fewer disagreeing dyads in the video-chatting condition (45 dyads, 90 participants), than the speaking (54 dyads, 108 participants) or writing (47 dyads, 94 participants) conditions. Note that because we started with slight sample size differences between conditions, after removing non-disagreeing dyads, the remaining sample does not show an imbalance in sample size across conditions, $\chi^2(2, n = 292) = 1.84$, $p = .399$.

Table A1.

Number of Participants at Each Point

Experiment Condition	Number Assigned in Log Sheet	Number Who Completed Pre-Survey	Number Who Completed Post-Survey	Number Who Were "Non-Neutral" (Attitude Strength > 0)	Number with Sig. Stance Difference (> 3)	Number Who "Strongly Disagreed" (Non-neutral and Sig. Stance Diff.)
Video-Chat	151	150	144	130	96	90
Speaking	134	133	124	114	114	108
Writing	136	135	128	122	96	94
**Total**	**421**	**418**	**396**	**366**	**306**	**292**

Percentage of Participants at Each Point

Experiment Condition	Completed Pre-Survey (Out of Total)	Completed Post-Survey (Out of Pre-Survey Respondents)	Non-Neutral (Out of Post-Survey Respondents)	Sig. Stance Difference (Out of Post-Survey Respondents)	Strongly Disagreeing (Out of Post-Survey Respondents)
Video-Chat	99%	96%	90%	67%	63%
Speaking	99%	93%	92%	92%	87%
Writing	99%	95%	95%	75%	73%
**Total**	**99%**	**95%**	**92%**	**77%**	**74%**

Overall, we conclude from this analysis that the differential attrition by condition is primarily due to differences in the stance distance for the video-chat and writing condition participants compared to the speaking condition participants. Because participants reported their pre-survey attitudes prior to learning about who their partner was and prior to learning their assignment to medium condition, it is impossible that the condition differences could be due to participants changing their behavior in response to their condition. Although we cannot fully rule out the possibility that pairs' assignment to condition could have somehow caused the different stance distances, we believe this difference is incidental (see also Part C).

B. Robustness Analyses.

When we conduct our analyses with all 396 participants for whom we have data, where there is no differential attrition by condition, we find that perceived understanding is higher in the speaking (i.e., video-chat and audio conditions) than in the writing condition, β = .40, SE = .06, $t(392) = 6.73$, $p < .001$, and perceived conflict is lower, β = -.27, SE = .10, $t(392) = -2.75$, $p = .006$. This result is also robust when controlling for stance differences between pairs (understanding: β = .42, SE = .06, $t(391) = 6.88$, $p < .001$; conflict: β = -.28, SE = .10, $t(391) = -2.74$, $p = .006$). Compared to the results without agreeing pairs (currently reported in the manuscript main text), we can see that the effects are similar (for understanding: standardized β = .48, SE = .07, $t(288) = 7.04$, $p < .001$; for conflict: β = -.41, SE = .09, $t(288) = -4.39$, $p < .001$). See these comparisons visualized in Figure B1 (perceived understanding) and Figure B2 (perceived conflict).

Figure B1.

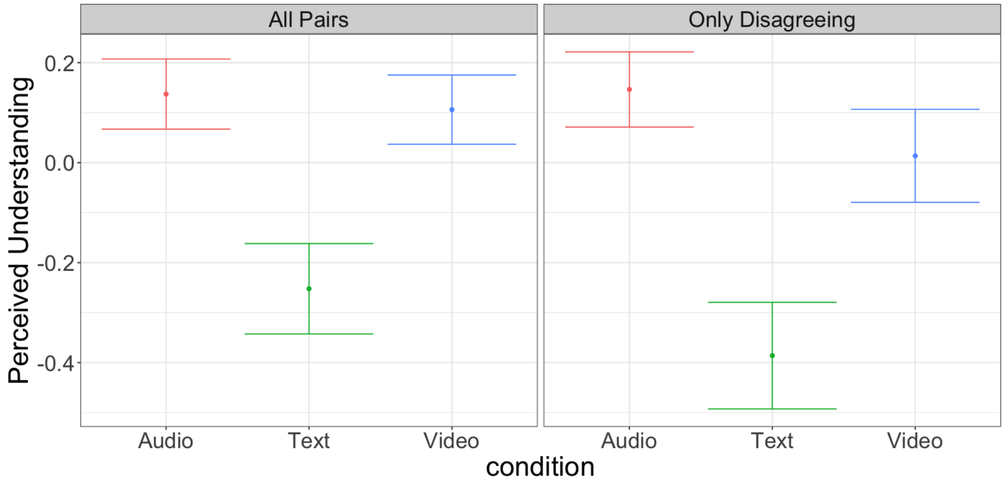

Figure B2.

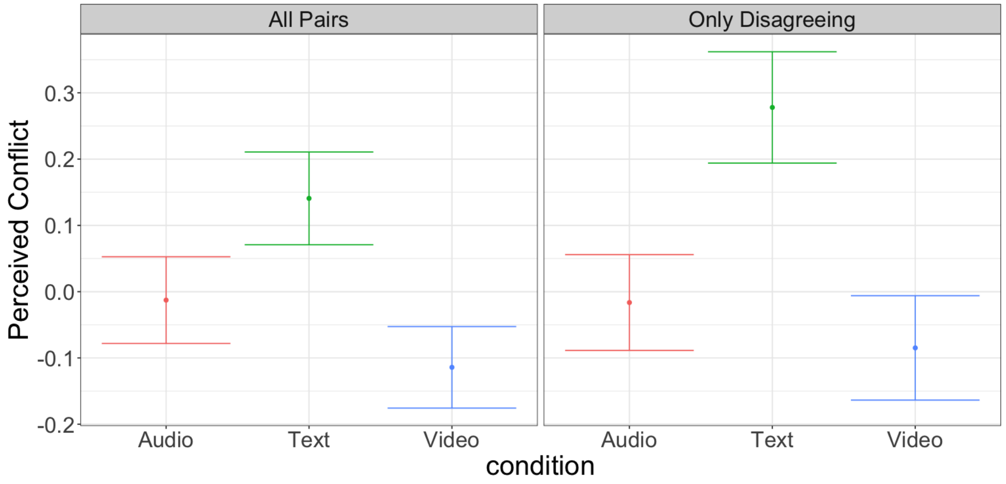

C. Balance Check.

Even though we think the stance distance was incidentally different between conditions, we examined other observable variables to make sure there were no other differences between conditions. Including all participants who completed the post-survey (n = 396), we found no other statistically significant differences on the following variables: topic assignment ($\chi^2(4) = 5.54$, p = .236), gender ($\chi^2(4) = 4.08$, p = .394), age (F(2, 393) = 2.26, p = .105), race ($\chi^2(30) = 28.85$, p = .525), or political ideology ($\chi^2(8) = 8.43$, p = .393). This is reassuring, as it suggests there are no demographic differences across conditions.

To summarize the key conclusions from the above analyses, we believe the differential attrition was incidental and our results are robust across different subsets of the data. We also don't have this issue in Studies 2 or 3, which show the same results as Study 1.

In Study 1, 15% of speaking participants versus 0% in writing were excluded due to recording issues.

This difference is only relevant for our analyses of the conversation recordings (i.e., the Language Analyses). For outcomes that do not use language variables, we do not exclude anyone due to recording issues. We generally have more technical issues with recordings in the speaking conditions than writing conditions across studies. We don't think this should meaningfully change the interpretation of results because technical issues would have occurred quasi-randomly across participants. To confirm this, we conducted balance checks, estimating whether any other variables predicted recording issues in the speaking condition. For participants in the speaking condition, we conducted a logistic regression analysis with technical issues as the dependent variable (binary) and topic assignment, gender, age, and political ideology as the independent variables. We found none of the variables were related to technical issues (ps > .123) apart from one variable: the topic of conversation. Specifically, there were slightly more recording issues among participants discussing the drinking age (vs. the other two topics; Odds Ratio = 3.1 p = .046). Note that this is a small, unpredicted effect that becomes non-significant with a standard correction for multiple comparisons (Bonferroni-adjusted p = .78), suggesting that the result may be

spurious.

In Study 3 (Berkeley), 36% of dyads were assigned to speaking and 64% to writing.

We did the same type of analysis described for Study 1: First, we identify where attrition occurs (A), then we conduct robustness analysis (B), and finally we run a balance check by condition (C).

Our additional analyses led us to realize that we could be clearer in the paper in explaining how many participants are included in each analysis. We now provide clearer sample size information in the paper. We also realized that some of our analyses could include additional participants (e.g., those who did not complete the pre-survey), so we have updated some of the analyses to maximize statistical power when possible. None of the results substantively changed, but some became a little stronger when including all possible participants in the analysis.

A. Attrition Analyses.

Tables A2-3 summarize the attrition in the Berkeley site, A4-5 summarize attrition in the MNSU site, and A6-7 summarize attrition in the ASU site. We now incorporate this information into the table in Supplemental Material Appendix B.

To review the results in the Tables, the most issues with attrition occurred at the Berkeley site (i.e., 24 missing post-surveys out of 160 possible, and 7 missing pre-surveys out of 38 participants); in contrast, there were only a few issues at MNSU (i.e., 7 missing post-surveys out of 154 possible, and no missing pre-surveys out of 32 participants) and almost no issues at ASU (i.e., no missing post-surveys out of 142, only 1 missing pre-survey out of 34 participants). Given that the Berkeley study was conducted first (in Jan 2019), then MNSU (Feb 2019), and ASU (March 2019), we think the improved response rates are due to improving our experimental paradigm after the Berkeley data collection effort.

Further examining just Tables A2-3 (Berkeley), there are two key areas of differential attrition: fewer speakers than writers completed pre-surveys (71% vs. 95%, respectively) and fewer speakers than writers completed post-surveys (76% vs. 94%, respectively). Although we can't know for certain why this happened, we suspect some speaking participants may not have realized they were supposed to be using the laptops in front of them to complete surveys (because speaking in itself does not require the use of a laptop, as compared to writing).

Also - please note that in the Study 3 UC Berkeley sample, one participant participated in both conditions, switching midway through the data collection - this is why two columns of the table include fractions in the participant counts. In Table S2 in our supplement, we also note this with an asterisk. No other participants switched conditions (participants were not supposed to be switching from their assigned role). This person also did not complete a pre-survey, so they are not in the main analyses reported in the paper.

Table A2. Participant Attrition in Bridge Berkeley Study

Number of Participants at Each Point

Experiment Condition	Num of Participants Who Had at Least 1 Conversation	Participants Who Did a Pre-survey	Participants Who Did at Least 1 Post-Survey	Participants Who Did a Pre-Survey and at Least 1 Post-Survey
Speaking	21.2	15	16.2	11
Writing	16.8	16	15.8	14
**Total**	**38***	**31**	**32**	**25**

Percentage of Participants at Each Point

Experiment Condition	Pre-Survey Completers (Out of Number Who Had Convo)	Post-Survey Completers (Out of Number Who Had Convo)	Pre and Post-Survey Completers (Out of Number Who Had Convo)
Speaking	71%	76%	52%
Writing	95%	94%	83%
**Total**	**82%**	**84%**	**66%**

Note: *One participant had one spoken conversation and the other four written conversations.

Table A3. Conversation Attrition in Bridge Berkeley Study

Number of Conversations at Each Point

Experiment Condition	Conversations Recorded in Log Sheet	Conversations Where Both Partners Did Post-survey	Conversations Where Only 1 Partner Did Post-survey	Conversations Where Neither Partner Did Post-survey	Conversations with at Least 1 Post-Survey	Conversations Where Both Partners Did Post-Survey and Pre-Survey
Speaking	41	23	13	5	36	12
Writing	39	38	1	0	39	29
**Total**	**80**	**61**	**14**	**5**	**75**	**41**

Percentage of Conversations at Each Point

Experiment Condition	Convos with 2 post-surveys (out of total convos)	Convos with 1 post-survey (out of total convos)	Convos with 0 post-surveys (out of total convos)	Convos with at least 1 post-survey (out of total convos)	Convos with 2 post-surveys and 2 pre-surveys (out of total convos)
Speaking	56%	32%	12%	88%	29%
Writing	97%	3%	0%	100%	74%
**Total**	**76%**	**18%**	**6%**	**94%**	**51%**

Table A4. Participant Attrition in Bridge MNSU Study

Number of Participants at Each Point

Experiment Condition	Num of Participants Who Had at Least 1 Conversation	Participants Who Did a Pre-survey	Participants Who Did at Least 1 Post-Survey	Participants Who Did a Pre-Survey and at Least 1 Post-Survey
Speaking	16	16	16	16
Writing	16	16	16	16
**Total**	**32**	**32**	**32**	**32**

Percentage of Participants at Each Point

Experiment Condition	Pre-Survey Completers (Out of Number Who Had Convo)	Post-Survey Completers (Out of Number Who Had Convo)	Pre and Post-Survey Completers (Out of Number Who Had Convo)
Speaking	100%	100%	100%

Writing	100%	100%	100%
**Total**	**100%**	**100%**	**100%**

Table A5. Conversation Attrition in Bridge MNSU Study

**Number of Conversations at Each Point**						
**Experiment Condition**	**Conversations Recorded in Log Sheet**	**Conversations Where Both Partners Did Post-survey**	**Conversations Where Only 1 Partner Did Post-survey**	**Conversations Where Neither Partner Did Post-survey**	**Conversations with at Least 1 Post-Survey**	**Conversations Where Both Partners Did Post-Survey and Pre-Survey**
Speaking	39	34	4	1	38	34
Writing	38	37	1	0	38	37
**Total**	**77**	**71**	**5**	**1**	**76**	**71**

**Percentage of Conversations at Each Point**						
**Experiment Condition**		**Convos with 2 post-surveys (out of total convos)**	**Convos with 1 post-survey (out of total convos)**	**Convos with 0 post-surveys (out of total convos)**	**Convos with at least 1 post-survey (out of total convos)**	**Convos with 2 post-surveys and 2 pre-surveys (out of total convos)**
Speaking		87%	10%	3%	97%	87%
Writing		97%	3%	0%	100%	97%
**Total**		**92%**	**6%**	**1%**	**99%**	**92%**

Table A6. Participant Attrition in Bridge ASU Study

**Number of Participants at Each Point**				
**Experiment Condition**	**Num of Participants Who Had at Least 1 Conversation**	**Participants Who Did a Pre-survey**	**Participants Who Did at Least 1 Post-Survey**	**Participants Who Did a Pre-Survey and at Least 1 Post-Survey**
Speaking	13	12	13	12
Writing	21	21	21	21
**Total**	**34**	**33**	**34**	**33**

**Percentage of Participants at Each Point**				
**Experiment Condition**		**Pre-Survey Completers (Out of Number Who Had Convo)**	**Post-Survey Completers (Out of Number Who Had Convo)**	**Pre and Post-Survey Completers (Out of Number Who Had Convo)**
Speaking		92%	100%	92%
Writing		100%	100%	100%
**Total**		**97%**	**100%**	**97%**

Table A7. Conversation Attrition in Bridge ASU Study

**Number of Conversations at Each Point**						
**Experiment Condition**	**Conversations Recorded in Log Sheet**	**Conversations Where Both Partners Did Post-survey**	**Conversations Where Only 1 Partner Did Post-survey**	**Conversations Where Neither Partner Did Post-survey**	**Conversations with at Least 1 Post-Survey**	**Conversations Where Both Partners Did Post-Survey and Pre-Survey**
Speaking	33	33	0	0	33	29
Writing	38	38	0	0	38	38
**Total**	**71**	**71**	**0**	**0**	**71**	**67**

Percentage of Conversations at Each Point

Experiment Condition	Convos with 2 post-surveys (out of total convos)	Convos with 1 post-survey (out of total convos)	Convos with 0 post-surveys (out of total convos)	Convos with at least 1 post-survey (out of total convos)	Convos with 2 post-surveys and 2 pre-surveys (out of total convos)
Speaking	100%	0%	0%	100%	88%
Writing	100%	0%	0%	100%	100%
**Total**	**100%**	**0%**	**0%**	**100%**	**94%**

B. Robustness Analysis.

Given the differential attrition in the Berkeley Bridge data, we conducted two robustness analyses. First, we re-ran the analyses excluding all of the Berkeley data, separately for the disagreeing and agreeing pairs. Without Berkeley data, for disagreeing pairs (n = 84 post-surveys), participants who spoke (vs. wrote) reported more understanding (standardized β = .91, SE = .17, t(80) = 5.38, p < .001, 95 % CI [.58, 1.24]) and non-significantly less conflict (standardized β = -.43, SE = .21, t(80) = -2.05, p = .043, 95% CI [-.83, .02]). These results are similar to those that include the Berkeley data reported in the paper (n = 104 post-surveys; understanding: standardized β = .97, SE = .16, t(99) = 6.26, p < .001, 95% CI [.66, 1.27]; conflict: standardized β = -.49, SE = .19, t(99) = -2.53, p = .013, 95% CI [-.86, -.11]). Without Berkeley data for the agreeing pairs (n = 192 post-surveys), participants who spoke (vs. wrote) again reported more understanding (standardized β = .49, SE = .14, t(188) = 3.55, p < .001, 95 % CI [.22, .75]) and non-significantly less conflict (standardized β = -.20, SE = .14, t(188) = -1.47, p = .144, 95% CI [-.47, .07]). These reports are similar to those that include the Berkeley data reported in the paper with n = 254 post-surveys: understanding (standardized β = .45, SE = .12, t(249) = 3.65, p < .001, 95% CI [.21, .69]) and conflict (standardized β = -.26, SE = .11, t(249) = -2.28, p = .023, 95% CI [-.48, -.04]).

Second, we re-ran the analysis to include all of the data we have across all three sites (even for participants who completed a post-survey but not a pre-survey or whose partner did not complete a post-survey; n = 425 post-surveys total). Note that this analysis combines the disagreeing pairs, agreeing pairs, and those pairs for whom initial disagreement or agreement could not be determined because one or both of them did not complete a pre-survey. With all the available post-survey data, we found that perceived understanding was still higher (standardized β = .62, SE = .10, t(420) = 6.54, p < .001, 95% CI [.44, .81]), and perceived conflict lower (standardized β = -.44, SE = .10, t(420) = -4.33, p < .001, 95% CI [-.63, -.24]) in the speaking than writing conditions.

All of these analyses are very consistent. We now report all of them in the Supplemental Materials Appendix H.

C. Balance Checks.

We tested in the Berkeley Bridge data whether the speakers differed from the writers on any observable features. Unfortunately, we already had differential attrition at pre-survey completion. But among those that did the pre-survey (15 speakers, 16 writers), the speakers (vs. writers) did not report different ages, t(54) = –1.80, p = .078, political orientations,

$\chi^2(3) = 6.78$, $p = .079$, pre-attitudes on the topic of conversation, $t(101.38) = 0.08$, $p = .932$, strength of opinion, $t(105.58) = 1.97$, $p = .052$, or knowledge of the topic, $t(98.34) = 0.34$, $p = .732$. However, participants' reported race, $\chi^2(4) = 30.19$, $p < .001$, and gender, $\chi^2(2) = 7.06$, $p = .029$, differed by medium. Specifically, there were disproportionately more participants identifying as White in the writing vs. speaking conditions (47.3% vs. 17.5%) but more participants identifying as Asian in the speaking vs. writing conditions (55.6% vs. 25.7%) and relatively more males in the writing vs. speaking conditions (59.5% vs. 39.7%). Although we are not certain why these differences exist, we don't have any reason to believe that differences in race and gender themselves – without any corresponding differences in attitudes related to the topic, which we don't see – would influence our results. We now report these in Appendix B.

5. Inconsistencies/errors

The authors use different thresholds for interpretating p-values.
o On page 13: a p-value of .043 is described as marginally significant.
o On page 14: The same result is described as non-significant: "We found no significant difference between the two speaking conditions - audiovisual or audio-only - which was somewhat surprising, given the additional visual information available in one group."

We apologize for the oversights you noted. In the discussion of this study (Study 1), we meant to highlight that video-chatting did not lead to *more* understanding compared to speaking with video-off, but you are correct that in fact it actually led to *less* understanding. Given that this result was not predicted, and runs in the opposite direction of our lay intuition, we do not want to overinterpret it in the discussion. We now describe the p=.043 effect in the Study 1 Results section: "[Perceived understanding] was, unexpectedly, higher in the speaking than video-chatting condition." In the Study 1 Discussion section, we now write: "We did not observe any improvement in understanding, or reduction in conflict, among pairs who video-chatted compared with those who spoke together without video. On the contrary, pairs reported slightly less understanding when video-chatting than speaking off-video. Given the additional visual information available when video-chatting, this result could be surprising but must be interpreted with caution. It is possible that the video quality was not optimal; moreover, video conversations are probably less nuanced than face-to-face, in-person conversations, which Study 1 did not test (but see Study 2A)."

o On page 49, two p-values of .071 and .042 are evidence of slightly more understanding without a statement about significance.

We were trying to interpret these results in tandem but we now separately describe them to be more accurate: "...participants expected writing to produce more understanding ($M = 4.22$, $SD = 0.30$) than video-chatting ($M = 4.04$, $SD = 0.29$; standardized $\beta = .13$, $SE = .06$, $t(402) = 2.04$, $p = .042$) although not statistically significantly more than speaking ($M = 4.06$, $SD = 0.29$; standardized $\beta = .11$, $SE = .06$, $t(402) = 1.81$, $p = .071$)."

On page 20, effects are described as being in a consistent direction although the effect for Study

2A is in the opposite direction: “The effects on perceived conflict were in a consistent direction but not always statistically significant. Average perceived conflict was lower when speaking (2A: M = 1.31, SD = 1.11; 2B: M = 0.54, SD = 1.15; 2C: M = 0.58, SD = 1.03) than writing (2A: M = 1.22, SD = 1.21; 2B: M = 0.67, SD = 1.25; 2C: M = 1.05, SD = 1.30).”

Thank you for catching this error. We now write that perceived conflict was statistically significantly lower when speaking than writing only in Study 2C, and not statistically different by condition in Studies 2A-B.

On page 33, the interaction with admission quotas vs. drinking age was not tested even though that will have the largest effect size.

Given that we did not anticipate or preregister an interaction by topic and have no theoretical reason to expect one, we did not conduct this analysis.

Thanks for the opportunity to read and review this manuscript. I hope these comments are helpful in strengthening the paper.

Yes indeed, we are particularly impressed by your thoroughness and attention to detail! We tried our utmost to address your comments with similar thoroughness.

Reviewer #3:

I would like to thank the authors for their effort to change the manuscript and respond to my comments from the first round. I am happy with many of the changes in the manuscript and convinced by most of the arguments in the response letter.

Thank you!

I have one remaining larger issue (point 1 below) that I believe the authors should handle more carefully, namely the incorporation of the earlier studies that I mentioned in my first review. Next to that, I have a few other more minor suggestions that I believe would improve the paper even more.

1. I appreciate that the authors cite the Roos et al. (2020a, 2020b) studies in the revised version of their manuscript. However, they do this very briefly in the Introduction and I do not agree with the statement made there about these studies, namely that they ‘do not capture conversational behavior’ (page 4). The (spoken and written) conversations in these studies were actually recorded and were coded by human coders for conversational behavior, namely responsiveness and ambiguity. I agree with the authors that there are other differences with the present manuscript (as they mention in their response letter), such as the smaller number of participants and the explicit creation/measurement of disagreement, which may be mentioned in the introduction (although I do not agree that their outcome measures such as perceived solidarity and polarization can be equated with ‘fun and clarity’). Furthermore, I believe the studies deserve some further discussion in either the Introduction or (perhaps preferably) the Discussion, given that some of the outcomes appear quite related to those of the present study,

even if perhaps not the exact same measures and concepts are used. I think it would be helpful for communication across different disciplines to acknowledge and discuss research from other disciplines despite difficulties related to different terminology. Although I respect and understand the authors decision to focus on receptiveness in this study, links can be made with concepts from the Roos et al. studies. As a first example, Roos et al. focus on ambiguity (ambivalence, disclaimers, and hedges) as one conversational behavior. Judging from the descriptions of features of receptive language, this may relate to subjectivity, hedges, and adverb limiters (which are defined as 'adverbs that increase ambiguity'). Two of these are found in the present study to be more frequent in speech than text, which is what Roos et al. find for ambiguity as well. Second, outcome measures such as perceived consensus, perceived solidarity, and perceived polarization appear related to the concept of conflict in the present study, which makes it relevant to discuss the similarities in results between the studies.

Thank you for providing a deeper review of the consistencies between the current research and the Roos et al papers.

We apologize for characterizing the Roos et al papers as not capturing conversational behavior; we meant to convey that there are critical differences between holistic human annotations (i.e., the method used in the Roos et al papers) and algorithmic analysis (i.e., the method we use) of conversation language. Annotations are not as reliable (humans will give different answers, and disagree with each other), reproducible (it is impossible to recreate the process of human judgment exactly), internally valid (annotators are not blind to condition), or interpretable (you cannot know the mechanism - i.e. what specific actions a person took that lead to a particular evaluation). In the introduction, we have now modified the sentence accordingly: "Little research has studied the combined effects of medium on conversation, particularly in disagreement conversations (for exceptions, see Roos, Koudenburg, et al., 2020; Roos, Postmes, et al., 2020)."

We think a nuanced comparison between our own results and those of the Roos et al papers is outside the scope of this manuscript for at least three reasons. First, we are trying to keep the paper to a reasonable length. Reviewer 2 has asked us to write a shorter, more focused paper that prioritizes our most well-powered and pre-registered analyses and we agree that this is the best approach for a general interest journal, because it focuses on the most important contributions to science. Second, although the Roos et al papers present interesting and generative findings, they have not been highly cited (54 total citations across both papers according to Google Scholar at the time of writing this) and have some methodological weaknesses (e.g., their small sample sizes and other issues that are non-exhaustive mentioned in the prior response letter), so we don't want to focus overly much on them. Third, the Roos et al papers are less similar to our own than you might think at first glance. They use different approaches for measuring behavior and generating conflict, and also have some different results (e.g., in the video-chat vs. text conditions). Thus, a deeper comparison between the papers would necessitate addressing these differences and reviewing their methodological weaknesses, which we don't think is necessary or productive.

That said, we agree there are interesting consistencies between the papers. You inspired us

to discuss our analysis of linguistic features in the General Discussion and relate it to the Roos et al papers: "We further test exactly which linguistic features are associated with different conversation media and constructive outcomes, for example finding that spoken conversations use more subjective language and hedges than written conversations (consistent with prior research that speech is more ambiguous than writing; Roos, Koudenberg, et al., 2020)." We believe this sentence, along with the revised sentence in the Introduction, provide adequate reference to the Roos et al papers, and will hopefully pique readers' interest in the work. (Incidentally, while revising our General Discussion, we also streamlined it to shorten the paper length and cited a few more seminal missing papers.) Overall, we appreciate that you have encouraged us to think more deeply about how our work fits within the existing literature including its similarities and differences with the Roos et al papers.

2. For studies 1 and 2, in the text only the results for the two main outcome variables are described, but in the figures all outcome measures are displayed, which is a bit confusing. Maybe I missed it, but it would be helpful if the authors explicitly state somewhere what they report in the main text (and whether/where the results for other variables are described in an Appendix).

Thank you for noticing this. We tried to be clearer by demarcating the "post-conversation measures" in each study's result section, and describing our additional measures in this section. We also edited our description of the figure to explicitly report that it contains multiple measures, i.e., "Figure 1 plots the effects of conversation medium on perceived understanding, conflict, and other outcome measures (see Appendix G for the pooled analysis of all outcome measures across Studies 1-3)."

3. On page 12, it is stated that: "Then, they were given three more minutes to jointly discuss, for a total of up to nine minutes." This is a bit unclear, did they discuss for 3 or 9 minutes or minimally 3 and maximally 9 (was it up to them)? Later it is stated that conversations in this study were 9 minutes.

The experimenters provide up to 9 minutes for the conversations. We now write: "Each participant first took up to three minutes to make their initial position clear (i.e., six minutes total). Then, they were given three more minutes to jointly discuss, for a total of up to nine minutes." We hope it is clear – participants had 3 minutes each (6 minutes total) and then another 3 minutes (making 9 minutes total).

4. The exact speech conditions in study 2 are not always clear. In 2A the speech condition was 'in person', so does that mean face-to-face, without a video connection? And in 2B/2C it was 'using the Skype platform', so does that mean videoconferencing or Skype calling (without video)?

Yes, that is correct. Participants in Study 2A were face-to-face in person (in the spoken condition) whereas on Skype for the video-chatting and audio-only conditions in Studies 2B and 2C. To make this design choice clearer in all studies, we added this information into a separate "Experiment Design" section. We hope our Methods sections are now easier to follow and understand.

5. There are some minor inconsistencies in the reporting of the results (e.g., no figure for study 3 and means/SDs for some results but not others).

Thank you for pointing this out. We liked your idea to add a figure for Study 3, which we have now done. We have added Figure 3 showing differences in perceived understanding and conflict by site. We also now report statistics for all results.

6. It might be interesting for methodological reasons to know if the prediction accuracy of the other's position ('actual understanding') correlates with your measure of perceived understanding in study 3.

Good idea! We ran your suggested analysis for the relevant item in the understanding index: "To what extent do you think you understood your partner's opinions?" and found a relationship between prediction accuracy and the reported understanding on this item (standardized $\beta = -.21$, SE= .11, $t(140) = -2.04$, $p = .043$, 95% CI [-.42, .01]).

7. The greater use of adverb limiters in speaking actually goes against the expectation given that more adverb limiters are related to less receptivity. This counterintuitive finding is not acknowledged nor explained or discussed in the text anywhere. I would appreciate a short discussion of this somewhere.

Thank you for pointing this out. You are right that the effect of limiters on receptiveness is negative, even if the use of limiters is more common in speaking than writing. This suggests that the greater use of adverb limiters in speech actually detracts from, rather than contributes to, the effect of medium on receptiveness. In our paper, we now focus on describing the conversation features that positively contribute to the effect of medium on receptiveness (e.g., the greater use of subjectivity and agreement language), although we kept the results for all features in Figure 5. Note that we do not expect all conversation features to be equally affected, or even necessarily affected in the same direction, by medium. An analogy would be if a business reports a net increase in sales, it does not mean every single product had an increase in sales. Some might go up, whereas others might go down. It's the same with receptiveness - because it has many component features, our algorithm can identify which specific features are positively associated with changes in receptiveness (something that is not possible with holistic human annotations, for example).

8. The 'other language measures' (page 46) are not defined and word count was already discussed above so it is unclear why this is repeated here.

We included that section to incorporate controls for related language metrics, specifically positive sentiment and politeness (as well as word count). We have renamed the section title to try to make our intent clearer.

9. In the first paragraph of page 52, the authors could again add the potential explanation that receptivity has a larger effect in text because it is more rare there.

Nice idea. We added this sentence: "Perhaps one reason receptive language has more impact in writing is because it is used more rarely."

10. At the end of page 54, the authors state that "communicators who used more receptive language tended to report greater understanding, less conflict, and higher impressions of each other after their conversations". If I understand it correctly, this result comes from the mediation analysis, however, the details of this analysis were not so explicit. Was this indeed investigated at the individual level: individuals who used more receptive language reported greater understanding? In that case, might it also be interesting to investigate the effect on the other: do the interlocutors of individuals who use more receptive language report greater understanding as well?

We clarified our mediation model. The (b) path shows that each person's receptive language use is positively associated with their partner's perceived understanding.

To decompose this relationship further for you, we separately tested how each of the two items in the "perceived understanding" scale (i.e., each person's belief that they understood their partner's position and that their partner understood their position) correlated with one's own receptive language and one's partner's receptive language. Participants' own receptive language was positively associated with their own belief they understood their partner (standardized: $\beta = 0.11$, SE = 0.02, $t(1388) = 4.57$, $p < .001$, 95% CI [0.06, 0.16]), their own belief their partner understood them (standardized: $\beta = 0.11$, SE = 0.02, $t(1388) = 4.57$, $p < .001$, 95% CI [0.06, 0.16]), their partner's belief they understood the participant (standardized: $\beta = 0.17$, SE = 0.02, $t(1388) = 7.42$, $p < .001$, 95% CI [0.13, 0.22]), and their partner's belief the participant understood them (standardized: $\beta = 0.11$, SE = 0.03, $t(1388) = 4.35$, $p < .001$, 95% CI [0.06, 0.16]). Further, the receptiveness of each person's language was positively correlated with the receptiveness of their partner's language ($r = .29$, $p < .001$).

We agree these associations are quite interesting, and are not surprised to observe them because in another paper that is currently under revision at a different journal, we find that receptiveness is "contagious" in conversation between speakers. We prefer to save a deeper discussion of this for our other paper, where we can address this question more comprehensively (we are also conscious about the length of this current manuscript, and want to focus on the central results).

11. Figure 2: "The variables are ordered in this plot based on effect size.": I first misunderstood this to mean absolute effect size, maybe this could be changed to: from most negative to most positive effect?

We updated the language per your suggestion. Thank you.

12. In Appendix T some causal explanations appear overstated: "whether aspects of speech influenced our dependent variables" and "role of speech rate in enhancing understanding".

See our response to R2 #2, whereby we have removed any inappropriate casual language

from the main text of the manuscript. We appreciate that you noticed that Appendix T also contained some of this language, which we have now edited to remove.

Thank you again to Reviewer 3 for continuing to engage with our paper and helping to strengthen it.

REVIEWERS' COMMENTS

Reviewer #2 (Remarks to the Author):

The revised paper makes an innovative and generative contribution to the literature. I appreciate that the authors thoroughly engaged with the reviews. I find the most recent submission significantly improved and I generally support acceptance. However, there remain some issues for which the revisions are suboptimal, and I recommend addressing these prior to publication.

1. Sensitivity (not post hoc) power analyses versus confidence intervals?

There appears to be confusion about my prior comment about interpreting null effects. The authors added confidence intervals. While confidence intervals are useful to report, they are not a substitute for sensitivity power analyses, which, in my opinion, would improve the paper if featured more prominently in the main text.

Sensitivity power analyses help determine which effect sizes can be ruled out given the design. These differ from post-hoc power analyses; my original comments did not advocate for post-hoc power.

To illustrate the stakes: the detectable interaction effects in Studies 2A–C were extremely large (βs: 1.80, 1.10, 1.25), especially relative to the largest observed main effect ($\beta = .53$). Thus, these studies were fundamentally ill-powered to detect realistic interaction effects.

The paper should explicitly state that Studies 2A–C primarily serve a stimuli-sampling function—demonstrating robustness across contexts. However, the absence of statistically significant interactions cannot be interpreted as evidence of null effects. Many readers may mistakenly make this inference, so the manuscript should proactively prevent misinterpretation.

To be clear, I am not arguing the authors should have powered the studies to detect realistic interactions; as Cohen (1988) notes, this is often infeasible. My objection is solely to be transparent that statistical power was insufficient to detect interaction effects.

Thank you for your careful clarification regarding sensitivity power analyses and the interpretation of non-significant interaction effects. We have revised the manuscript to directly address these concerns.

The manuscript now explicitly states that the absence of statistically significant interactions should not be interpreted as evidence that the effect of medium is invariant to changes in conversation structure. Instead, we clarify that Studies 2A–C had limited sensitivity to detect the estimated interaction effects and have added the minimum detectable effect sizes in the main text. Consistent with your suggestion, we now frame Studies 2A–C as stimulus-sampling exercises that demonstrate the robustness of the main effect of medium across conversational contexts, rather than as informative tests of moderation.

I was confused by the author's statement in the memo: "In fact, it was not always possible for the minimum detectable interaction effects to be simulated with 95% power. The maximum

power we could simulate interaction effects for across each study were (2A: 96%, 2B: 73%, 2C: 89%).”

This statement is unclear, as there is always a minimum detectable effect size associated with any desired power level—even if that effect is very large. It would be helpful for the authors to clarify what constraints prevented them from identifying the minimum detectable effect size.

We apologize for not explaining this clearly enough in the letter. We note that because sample size was held fixed in the sensitivity analysis simulations, 95% power was not reached in Studies 2B and 2C within the examined range of interaction effect sizes (β = −2 to +2). This reflects the limited sensitivity of these study designs, rather than a limitation of the simulation approach. We hope our revisions address your concerns and improve the transparency and interpretability of the findings.

2. Interpretation of Findings in Study 4

The interpretation currently collapses across outcomes, whereas Figure 4 indicates distinct patterns for perceived understanding vs. perceived conflict. The authors summarize the findings as: “the current study finds an effect of the production medium whereby observers perceive higher understanding and less conflict between communicators who spoke than wrote to one another” and “In particular, spoken statements that were heard by observers (i.e., in the “produced-by-speaking and consumed-by-listening” condition) were deemed to create the most understanding and least conflict.”

But looking at Figure 4, the data appear to support different conclusions for the two dependent variables. To accurately represent the results, the manuscript should interpret simple effects, not only main effects and interactions.

• The production medium has a clear effect on perceived understanding; words produced by speaking result in more perceived understanding than words produced by writing. This effect is stronger when the words are produced by listening.
• In contrast, there is no clear overall effect of production medium on perceived conflict. Instead, ONLY when words are produced by speaking AND consumed by listening, there is a reduction in perceived conflict.

Thank you for encouraging us to decompose the interaction effects using simple effects to describe the patterns more thoroughly. We now do this in the manuscript.

Minor points
• Page 16: “Of that set, 1 person (0.2%) could not participate in the study because either they could not be matched with a partner within their session or were not fluent enough in English.” The exclusion rationale is ambiguous (“either… or…”). I’d recommend clarifying which applies.

Thank you for noting this ambiguity. We have clarified the exclusion rationale. The participant was excluded because they could not be matched with a partner within their session, and the reference to English fluency has been removed.

• Page 18: “Of those who participated, 46 (9.5%) were removed from the analysis due to our predetermined exclusion criteria.” I’d recommend that the word “predetermined” is cut because the study was not preregistered.

We have revised the manuscript to remove the word “predetermined” from this sentence on page 18, as the study was not preregistered.

• Page 20: “Our final sample was 414 individuals.” The numbers don’t match. 489 – 17 – 54 = 418 (not 414).

Thank you for catching this discrepancy. We identified that the correct number of excluded participants is 58 rather than 54, which yields a final sample size of 414. We have corrected this number in the manuscript accordingly.

• Page 52: “Overall, these lay theory data suggest that people expect a disagreement will be more constructive (e.g., lead to more understanding and less conflict) in a written conversation than in a spoken conversation.” This is a slightly inaccurate characterization as the difference between speaking condition and writing condition was not statistically significant for perceived understanding.

Thank you for catching this, we have revised the sentence to focus on the statistically significant differences between speaking and writing in the prediction data, like predicted conflict and enjoyment.

Thank you for the opportunity to review this revised submission. I believe the paper will make a valuable contribution. I wish the authors all the best as they finalize the manuscript.

Thank you so much for your careful review and suggested revisions throughout this process.

Reviewer #3 (Remarks to the Author):

The authors have addressed all my concerns satisfactorily.

Thank you for your help in improving our manuscript!
